# Submonomer synthesis of peptoids containing *trans*-inducing *N*-imino- and *N*-alkylamino-glycines†

**DOI:** 10.1039/d1sc00717c

**Published:** 2021-05-10

**Authors:** Carolynn M. Davern, Brandon D. Lowe, Adam Rosfi, Elon A. Ison, Caroline Proulx

**Affiliations:** Department of Chemistry, North Carolina State University Raleigh NC 27695-8204 USA cproulx@ncsu.edu

## Abstract

The use of hydrazones as a new type of submonomer in peptoid synthesis is described, giving access to peptoid monomers that are structure-inducing. A wide range of hydrazones were found to readily react with α-bromoamides in routine solid phase peptoid submonomer synthesis. Conditions to promote a one-pot cleavage of the peptoid from the resin and reduction to the corresponding *N*-alkylamino side chains were also identified, and both the *N*-imino- and *N*-alkylamino glycine residues were found to favor the *trans*-amide bond geometry by NMR, X-ray crystallography, and computational analyses.

## Abstract 2

The use of hydrazones as a new type of submonomer in peptoid synthesis is described, giving access to peptoid monomers that are structure-inducing.

## Graphical abstract

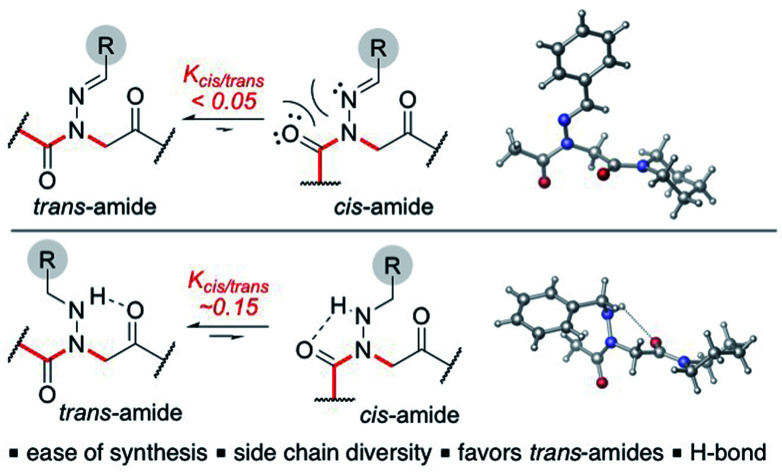

## Introduction

**Fig. 1:**
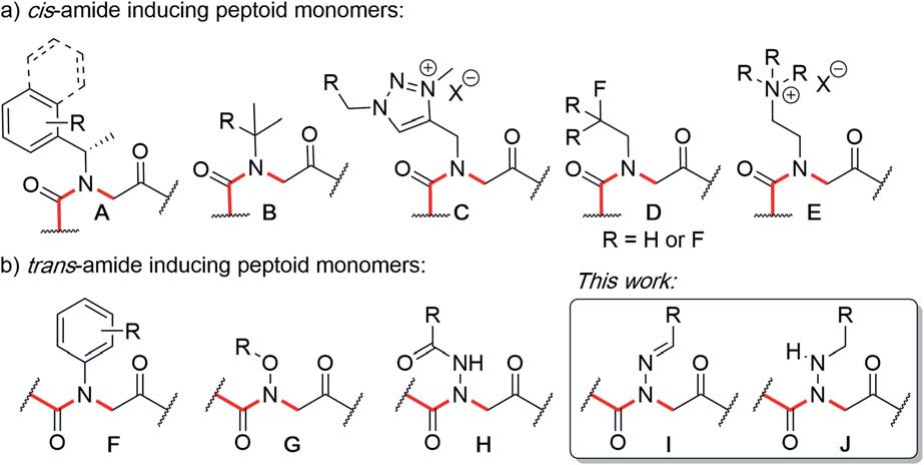
*N*-Substituted glycine (peptoid) monomers that favor (a) the *cis* or (b) *trans*-amide bond geometry.

Peptoids are *N*-substituted glycine oligomers that have been widely pursued to mimic both peptides and synthetic polymers due to their increased stability, ease of synthesis, and large side chain diversity.^1^ Compared to peptides, peptoids maintain the same glycine backbone; however, the side chain is located on the nitrogen atom, resulting in tertiary amides devoid of stereocenters and hydrogen bond donors (NH). To counter the resulting increase in backbone flexibility, a variety of peptoid side chains have been developed that induce either the *cis*- or *trans*-amide geometry through steric and electronic effects. For example, *N*-substituted glycines with α-chiral benzylic,^2^*tert*-butyl/α-*gem*-dimethylated,^3^ triazolium,^4^ fluorinated,^5^ and cationic alkyl ammonium^6^ side chains have all been found to favor the *cis*-amide bond configuration (Fig. 1a). Comparatively fewer options exist to reinforce *trans*-amide bonds, which include *N*-(aryl)-,^2*c-e*,7^*N*-(alkoxy)-,^8^*N*-(hydroxyl)-,^9^ and *N*-(acyl hydrazide)^10^ glycines (Fig. 1b). With this existing set of conformationally-restricted peptoid monomers, several secondary structures such as helices, ribbons, loops, turns, and sheet-like structures have been accessed,^1^ making peptoids a versatile, structurally tunable platform to design folded structures. However, poor water solubility and limited functional group diversity plague many of the existing structure-inducing peptoid monomers, and there remains a need to continue expanding the toolbox of *trans*-amide inducing peptoid monomers.

**Scheme 1:**
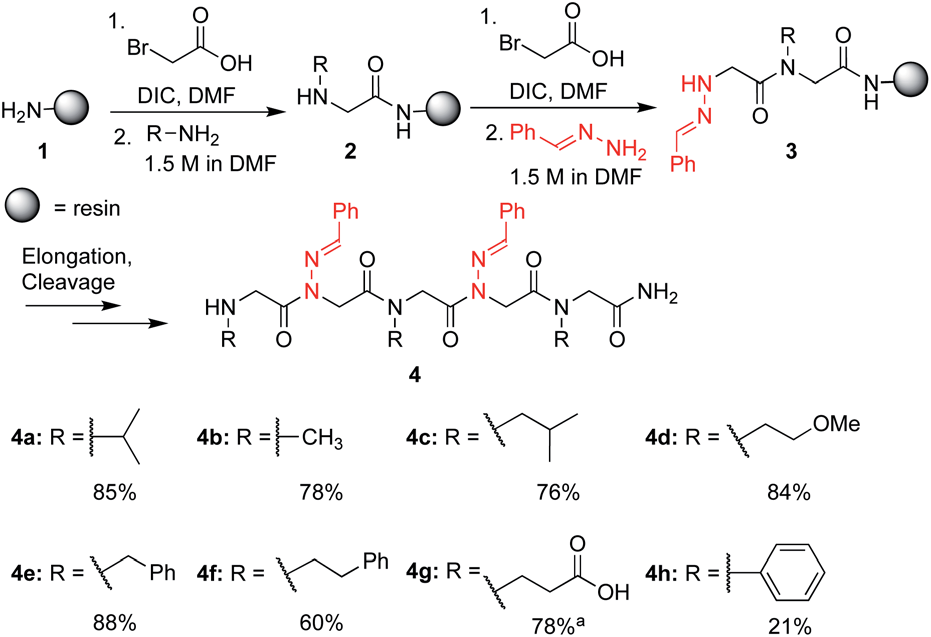
Crude purities of peptoid pentamers **4a–h**. LCMS purity of the crude peptoid at 214 nm. Unless otherwise noted, peptoids were cleaved from the resin using 95 : 5 TFA : H_2_O for 10 minutes. ^a^ Cleavage was performed using 95 : 5 TFA : H_2_O for 2 h.

In solid-phase submonomer peptoid synthesis, cycles of (1) acylation with activated bromoacetic acid and (2) S_N_2 displacement with primary amines are repeated until the desired peptoid length is achieved (Scheme 1).^11^ Optimization is often required to install structure-inducing residues, which tend to be sterically hindered and less nucleophilic. This includes aniline nucleophiles used in the synthesis of *N*-aryl glycines, the most widely utilized *trans*-inducing peptoid monomers to date. To improve the synthesis of peptoids possessing multiple *N*-aryl glycines, a method relying on the addition of silver perchlorate during the displacement step with electron-poor anilines was recently developed.^12^ However, given the importance of both side chain diversity and secondary structure rigidification in peptidomimicry, novel *trans*-amide inducing peptoid residues that are amenable to rapid automated library synthesis using standard procedures would be beneficial. Moreover, the discovery of peptoid monomers that can reinforce side chain-to-side chain intermolecular interactions (*e.g*. π stacking, hydrogen-bonding) while also restricting backbone dihedral angles could be impactful in nanomaterials applications.^13^ Hydrazide submonomers have been explored to expand side chain diversity, introduce hydrogen bond donors/acceptors in the side chain, and reinforce *trans*-amide bond conformation (Fig. 1b, **H**). However, certain substituents were found to promote undesired intramolecular cyclizations, leading to peptoid truncations.^10^

**Fig. 2:**
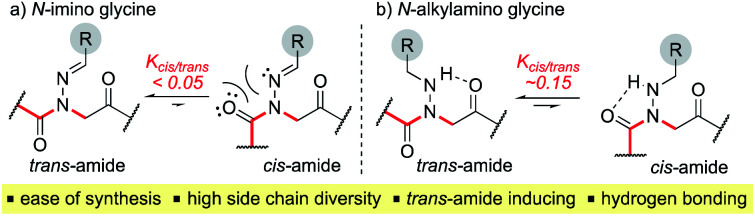
Structure and properties of (a) *N*-imino glycines and (b) *N*-alkylamino glycines.

Here, we introduce the use of hydrazones as submonomers in solid phase peptoid synthesis that can afford both *N*-imino and *N*-alkylamino glycine residues (Fig. 2), depending on the conditions used to cleave the peptoids from the solid support. Such monomers have shown promise in medicinal chemistry platforms when incorporated into peptides, and exhibited sufficient stability for biological testing.^14^ However, peptides and peptidomimetics containing multiple different *N*-imino glycines could not be accessed with previous methods, which relied on the use of *tert*-butyl carbazate as a submonomer and diversification of the *N*-amino glycine after resin cleavage.^14^ Hydrazones are readily available *via* condensation of hydrazine with aldehydes and ketones, providing easy access to a wide array of side chain diversity. We demonstrate for the first time that they are readily incorporated during submonomer peptoid synthesis, affording *N*-imino glycine- or *N*-alkylamino glycine-containing peptoids following cleavage from the resin with trifluoroacetic acid (TFA) and various scavengers. We further demonstrate that both *N*-imino glycines and *N*-alkylamino glycines strongly induce *trans*-amide bonds, with introduction of a new side chain – backbone hydrogen bond (Fig. 2) and changes in the side chain dihedral angles (*χ*) upon reduction.

## Results and discussion

### Solid phase submonomer synthesis and resin cleavage

To validate the use of hydrazones as submonomers and verify compatibility with other peptoid monomers, sandwich sequences^15^ were first synthesized, alternating between benzaldehyde hydrazone as a representative example and a set of eight other primary amines (Scheme 1). We included *N*-phenyl glycine in our study in attempts to afford all-*trans* peptoid oligomer **4h**. In all cases, the displacement step was performed by treating the resin-bound bromoacetylated peptoid with a 1.5 M solution of amine or hydrazone in DMF for 1 h, which are standard conditions for solid phase submonomer peptoid synthesis. Most analogs were obtained in high conversions, demonstrating the ease of using benzaldehyde hydrazone as a submonomer. Notably, there was no evidence of *trans*-imination reactions upon repeated treatment with excess primary amines.‡‡*Trans*-imination would lead to the free *N*-amino glycine which would presumably give rise to side products upon further bromoacetylation/displacement reaction cycles. However, using standard procedures, **4h** was only detected in ∼20% conversion by LCMS analysis. While it is possible that the *N*-terminal *N*-aryl glycine oxidized,^12,16^ the corresponding α-oxo aldehyde side product was not observed and we attributed the low crude purity to difficulties in the bromoacetylation of non-nucleophilic *N*-aryl glycine residues.^7*a*,12,16*c*^ This demonstrates that *N*-imino glycines are easier to incorporate than commonly employed *trans*-inducing *N*-aryl glycine peptoid monomers.

*N*-Imino glycines were found to undergo undesired acid-catalyzed hydrolysis during cleavage from the resin using 95 : 5 TFA : H_2_O, giving unsubstituted *N*-amino glycine residues. However, in the case of benzaldehyde hydrazone, this side reaction was only prevalent when the *N*-imino glycine residue was at the *N*-terminus; acetylation or embedding this residue within a peptoid resulted in little to no side chain hydrolysis for peptoids **4a–h** (ESI†). Reduction of the C=N bond was also observed when common carbocation scavengers [*e.g.* triisopropylsilane (TIPS) and triethylsilane (TES)] were added to the cleavage cocktail. Hydrazone reduction during resin cleavage could provide rapid access to valuable peptoid monomers with a new hydrogen bond donor site (NH), eliminating the need for subsequent treatment with sodium cyanoborohydride. We hypothesized that both *N*-imino- and *N*-alkylamino glycine-containing peptoids could be obtained cleanly contingent on the cleavage cocktail used. Thus, careful examination of different resin cleavage conditions was undertaken using tripeptoid **5** as a model compound (Table 1). Using a standard cleavage cocktail of 95 : 2.5 : 2.5 TFA : H_2_O : TIPS resulted in a mixture of products and low crude purity after 2 h, and should therefore be avoided (Table 1, entries 1 and 2). However, cleavage of the resin-bound peptoid using 95 : 5 TFA : H_2_O in the absence of TIPS provided compound **6** as the major product (Table 1, entries 3 and 4), and using 95 : 5 TFA : TES without water afforded the reduced peptoid **7** in good crude purities (Table 1, entries 5 and 6).

**Table 1:** Optimization of peptoid cleavage from the resin

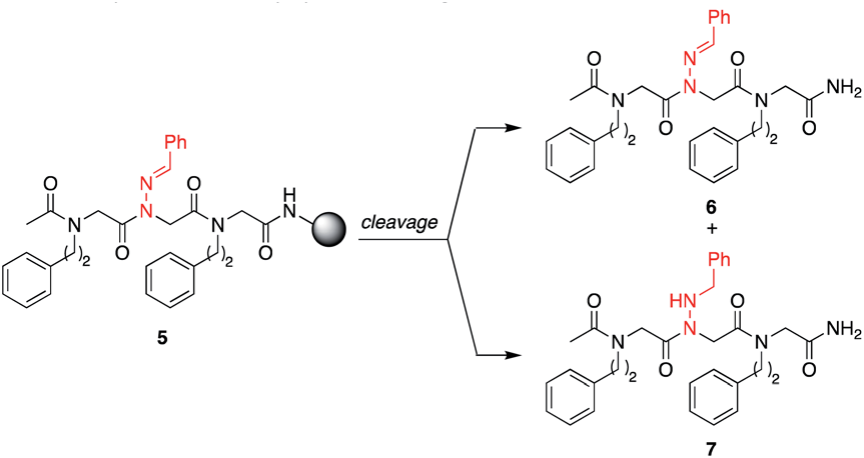				
Entry	Cleavage cocktail	Time	Crude purity^a^ (%)	**6** : **7** ratio
1	TFA : H_2_O : TIPS 95 : 2.5 : 2.5	10 min	60	67 : 33
2	TFA : H_2_O : TIPS 95 : 2.5 : 2.5	2 h	26	34 : 66
3	**TFA : H**_**2**_ **O 95 : 5**	**10 min**	**75**	**100 : 0**
4	TFA : H_2_O 95 : 5	2 h	66	100 : 0
5	**TFA : TES 95 : 5**	**10 min**	**89**	**2 : 98**
6	**TFA : TES 95 : 5**	**2 h**	**76**	**0 : 100**
7	TFA : TIPS 90 : 10	2 h	61	5 : 95
8	TFA : DCM: TIPS 45 : 50 : 5	10 min	83	96 : 4
9	TFA : DCM: TIPS 45 : 50 : 5	2 h	58	57 : 43
10	**TFA : phenol 95: 5** b	**2 h**	**69**	**100 : 0**

a LCMS purity of the crude peptoid mixture at 214 nm (*i.e*., % area corresponding to the sum of **6** + **7**).

b The peptoid was precipitated in cold Et_2_O before LCMS analysis.

We note that although TIPS efficiently reduced the benzaldehyde-derived hydrazone side chain to give **7** after 2 h (Table 1, entry 7), using TES instead afforded a higher crude purity overall with better selectivity for peptoid **7**. Diluting a 90 : 10 TFA : TIPS cleavage cocktail two-fold with dichloromethane yielded 96 : 4 and ∼1 : 1 mixtures of peptoid **6** and **7** after 10 minutes and 2 hours, respectively (Table 1, entries 8 and 9). To prevent both hydrolysis and reduction of the *N*-imino glycine residue, another cleavage solution using phenol as a carbocation scavenger (95 : 5 TFA: phenol) was also tried, which afforded **6** in comparable crude purities after Et_2_O precipitation as the 2 h 95 : 5 TFA: water cleavage (Table 1, entry 10). Overall, the excellent selectivity and good crude purities observed using 95 : 5 TFA : H_2_O and 95 : 5 TFA : TES to give **6** and **7**, respectively, encouraged us to pursue longer sequences with more diverse *N*-imino glycine residues.

**Fig. 3:**
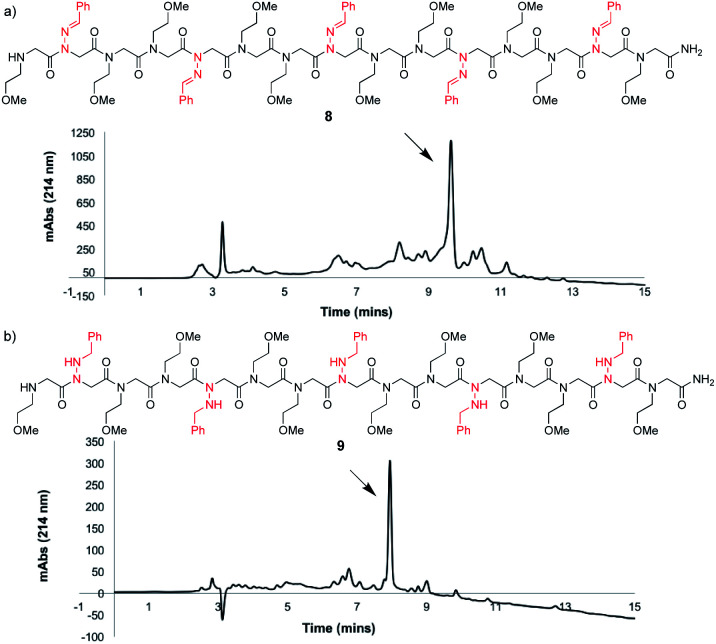
LCMS traces before purification at 214 nm of an oligomer containing five *N*-imino glycine residues derived from benzaldehyde hydrazone (a) cleaved using 95 : 5 TFA : H_2_O for 10 minutes (unreduced) and (b) cleaved with 95 : 5 TFA : TES for 2 h followed by treatment of the crude peptoid with a fresh solution for another 2 h (reduced).

To study the utility of benzaldehyde hydrazone as a submonomer in the synthesis of a longer oligomer, a 15mer sequence was synthesized that contains five *N*-imino glycine residues separated by two *N*-(2-methoxyethyl)glycines (*N*me) to increase water solubility (Fig. 3a)*.* Peptoid **8** was synthesized using an automated peptide synthesizer, giving the desired product in 63% crude purity after resin cleavage with 95 : 5 TFA : H_2_O for 10 minutes. Gratifyingly, reduction of all five *N*-imino glycine residues was also possible by using a 95 : 5 TFA : TES cleavage cocktail for a total of 4 h (Fig. 3b), giving peptoid **9** as the major product. Installing five *N*-imino glycines in a row was also found to be possible, albeit affording the desired peptoid in lower crude purity (ESI†). In that case, the major side product appears to be a deletion sequence lacking only one *N*-imino glycine residue.

### Increasing side chain diversity in *N*-imino- and *N*-alkylamino glycine peptoids

**Scheme 2:**
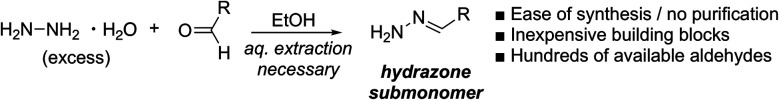
Hydrazone submonomer synthesis.

To increase the diversity of side chains, we next evaluated the synthesis and use of twelve different aliphatic and (hetero)aromatic hydrazone submonomers. In the literature, hydrazones are typically synthesized by reacting excess hydrazine monohydrate with an aldehyde in ethanol, and are used immediately in their crude form after an aqueous extraction (Scheme 2).^17^ This simple one-step procedure should allow the synthesis of hundreds of new peptoid submonomers from common and often inexpensive building blocks. Despite the large excess of amine typically used in solid phase submonomer peptoid synthesis (1–2 M solutions, 15–30-fold excess), we found that the procedure to make the hydrazone submonomers was critical for reproducible results. To access more water soluble and volatile hydrazones (*e.g.* isobutyraldehyde hydrazone), we briefly explored methods that bypassed aqueous extractions.^18^ However, after troubleshooting, the use of excess hydrazine monohydrate followed by an aqueous work-up proved essential for more consistent results in peptoid synthesis (ESI†).§§Even when the hydrazone is crystalline, it is more effective to carry out an extraction rather than simply rinse the crystals with water. Thus, all hydrazones in this study were ultimately synthesized using this method (Scheme 2), and were used immediately afterwards as freshly prepared solutions in DMF.

**Scheme 3:**
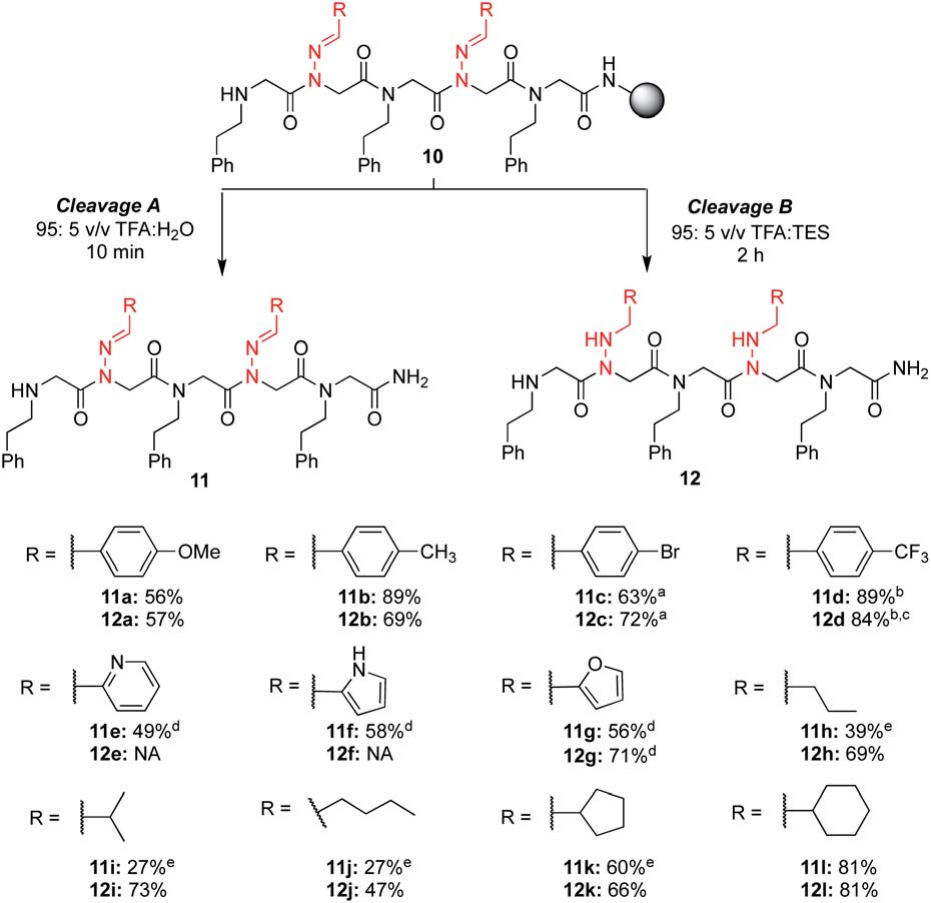
Crude purities of peptoid pentamers **11a–l** and **12a–l** at 214 nm. ^a^ Hydrazone displacements were performed in a heated sonicator bath. ^b^ Hydrazone displacements were performed with a 3 M solution for 2 h in a heated sonicator bath. ^c^ Crude purity after a second treatment with a fresh solution of 95 : 5 TFA : TES for 2 h. ^d^ A 5 minute chloroacetylation was performed instead of a 25 min bromoacetylation, and the hydrazone displacement was performed in a heated sonicator bath with a 1.5 M hydrazone solution made using 1 M KI in DMF. ^e^ The major side product corresponds to hydrolysis of one or more hydrazone side chains.

With these different hydrazone submonomers in hand, we synthesized pentamers **11–12a–l** (Scheme 3) to test their efficiency in solid phase submonomer peptoid synthesis. Here, we chose to use *N*-(2-phenylethyl)glycine (Npe) as a spacer to facilitate HPLC analyses of analogs containing *N*-imino glycines with non-aromatic side chains. While installation of electron-rich benzaldehyde hydrazones proceeded with no deletion side product, the use of *p*-Br and *p*-CF_3_-benzaldehyde hydrazones as submonomers required optimization of the displacement reaction, presumably due to the reduced nucleophilicity imparted by the electron-withdrawing groups. Ultimately, increasing the concentration of the hydrazone solution to 3 M and placing the resin in a heated sonicator bath (60 °C) for 2 h provided pentamer **11d** in good crude purity. Cleavage of this peptoid from the resin using 95 : 5 TFA : TES provided reduced **12d** in 74% crude purity, with an additional peak corresponding to the parent peptoid with only one reduced hydrazone (11%). After redissolving the crude peptoid in a fresh solution of 95 : 5 TFA : TES and stirring for an additional 2 h, full conversion to pentamer **12d** was detected in 84% crude purity. Displacement with *p*-bromo benzaldehyde hydrazone was also performed in a heated sonicator bath for improved yields.

Analogs **11e–g**, containing heterocyclic moieties (*e.g.* pyridine, pyrrole (unprotected), furan), were synthesized using chloroacetic acid to avoid irreversible alkylation of the side chain, as previously reported.^19^ When using these conditions for the acylation step, addition of potassium iodide in the hydrazone submonomer solution was found to be necessary to give the more reactive iodoacetamide after *in situ* halogen exchange (ESI†). With the exception of anilines, other amines are able to displace the chloride,^19^ and the submonomer solution of phenylethylamine was thus prepared without KI. From these results, we established that hydrazones exhibit similar (low) nucleophilicity compared to anilines. Yet, the high yield of most hydrazone-containing peptoids using standard procedures indicates that *N*-imino glycines are generally easier to install than *N*-aryl glycines (*e.g.* peptoids **4a–g***vs.***4h**, *vide supra*). In the process of this optimization, we began to wonder if an undesired *N*-alkylation could also occur at the imino nitrogen when using bromoacetic acid, lowering the overall crude purities in all of our hydrazone-containing peptoids. However, when we compared the two different monomer addition cycles with several other sequences lacking heterocycles, we did not observe an increase in yields using chloroacetic acid (data not shown). This suggests that alkylation of the imino nitrogen by bromoacetic acid is unlikely to be leading to significant side product formation.

No reduction of the imine functional group in heterocyclic-containing analogs **11e** and **11f** was observed using the 95 : 5 TFA : TES cleavage conditions over two hours. Conversely, *N*-imino glycines with aliphatic side chains readily provided reduced peptoids **12h-l** under these conditions, and attempts to obtain the parent compounds **11h-j** in the absence of TES resulted in lower conversions due to rapid imine hydrolysis. It is worth noting that in these cases we observed hydrolysis side products even using a 95 : 5 TFA : phenol cleavage cocktail, demonstrating that rapid hydrolysis could occur in the LCMS vial with these analogs when dissolved in a MeCN : H_2_O mixture. In comparison, peptoids with *N*-imino glycines possessing cyclopentane (**11k**) and cyclohexane (**11l**) side chains were obtained as the major product when the LCMS analysis was performed directly after resin cleavage with 95 : 5 TFA : H_2_O for 10 min.

### Hydrolytic stability of *N*-imino glycine-containing peptoids

**Fig. 4:**
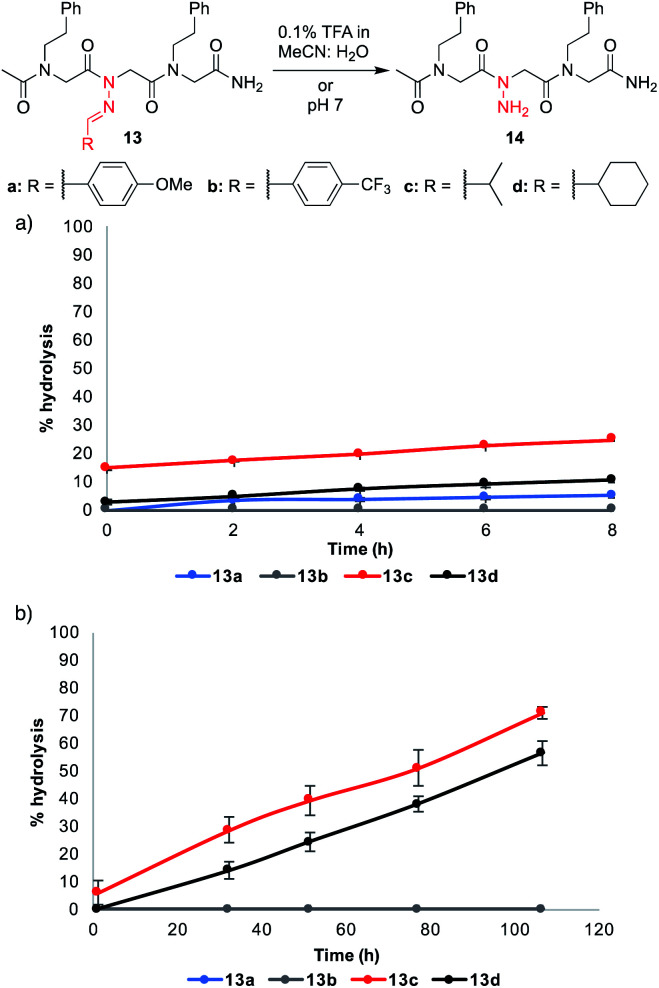
Stability studies in (a) 0.1% TFA MeCN : H_2_O and (b) pH 7. % Hydrolysis was determined by integrating the LCMS peak areas for **13** and **14** at 214 nm. The blue curve overlays with the gray curve and is not visible.

Despite their improved stability relative to **11h–j**, certain other *N*-imino glycine-containing peptoids were found to undergo hydrolysis to varying degrees over time. To get additional insight about the relative stability of these peptoids, we monitored select peptoid trimers (**13a–d**) in 0.1% formic acid (FA) in 1 : 1 MeCN : H_2_O for 8 hours and in phosphate buffer pH 7 for several days (Fig. 4).¶¶Small amounts of MeCN were added to the peptoid solution in phosphate buffer pH 7 (∼15% v/v) for solubility. These conditions were chosen to mimic (1) the mobile phase that would be needed for reverse-phase HPLC purifications and (2) the mild aqueous conditions that would be necessary to conduct biological assays. Aliphatic hydrazones in analogs **13c–d** underwent hydrolysis to a greater extent under both conditions, with ≥50% hydrolysis observed after 4 days in phosphate buffer (pH 7). On the other hand, aromatic hydrazones remained stable both in the presence of 0.1% FA over 8 h and in neutral aqueous buffer over days. Moreover, addition of 1 mM Lys or Cys did not lead to or increase decomposition rates for **13a–d** (ESI†). Consistent with the literature, we conclude that *N*-imino glycine-containing peptoids possessing aromatic side chains should be stable enough for biological analysis at neutral pH.^14^ In the course of our studies, we discovered that acetylation of the *N*-terminus increased the stability of peptoids relative to their unacetylated counterparts (ESI†). Moreover, dissolving the peptoids in 1 : 1 MeCN : H_2_O containing 0.1% trifluoroacetic acid instead of formic acid led to slightly faster hydrolysis and the appearance of a side product over several days, likely arising from an acetyl group transfer onto the *N-*amino glycine (ESI†). Hence, care should be applied when purifying *N*-imino glycine-containing peptoids by preparative HPLC containing 0.1% acid in the mobile phase, and fractions should be immediately frozen and lyophilized. Peptoids comprising of *N*-imino glycines with aliphatic side chains can be cleaved from the resin with 95 : 5 TFA : TES v/v to access more stable (reduced) derivatives.

### Synthesis of a peptoid with multiple different *N*-imino- and *N*-alkylamino glycines

**Fig. 5:**
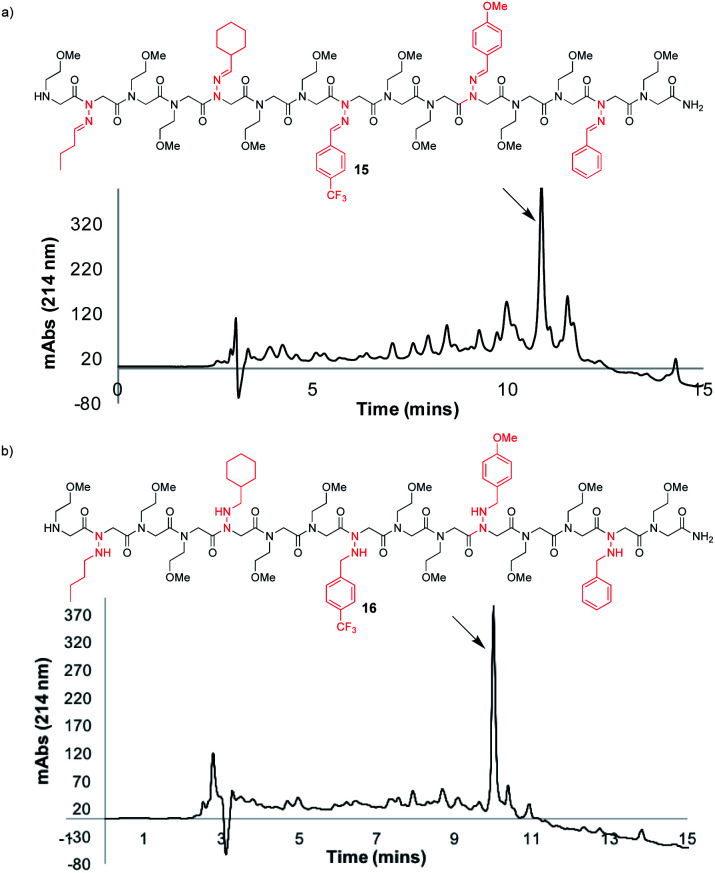
LCMS traces before purification at 214 nm of a peptoid 15mer containing five different *N*-imino glycine residues, cleaved from solid support using (a) TFA : DCM: TIPS 45 : 50 : 5 for 10 minutes, and (b) 95 : 5 TFA : TES for 2 h, followed by a second treatment with a fresh solution of 95 : 5 TFA : TES for 2 h.

To further demonstrate the versatility of hydrazone submonomers in solid phase peptoid synthesis, we synthesized a peptoid possessing five different *N-*imino glycines by automated solid phase peptoid synthesis (Fig. 5). All hydrazone submonomers were freshly prepared as 1.5 M solutions in DMF with 1 h displacement times, with the exception of 4-CF_3_-benzaldehyde hydrazone (3 M, 4 h). The synthesis was paused at the 7mer and 13mer stages, and resumed after preparing fresh solutions of the hydrazones needed to complete the 15mer. Unfortunately, initial attempts to obtain peptoid **15** using 95% TFA: 5% H_2_O during resin cleavage were unsuccessful. Upon further investigation using hexapeptoids with two different *N*-imino glycines as model compounds, we discovered that peptoids containing multiple different *N*-imino glycines could undergo rapid hydrazone exchange under these conditions, resulting in mixtures of products with scrambled *N*-imino glycine compositions (ESI†). We hypothesized that a cleavage cocktail that did not contain water would inhibit this undesired hydrazone exchange. As previously demonstrated, 95% TFA : 5% phenol could also be utilized to afford *N*-imino glycine-containing peptoids (Table 1, entry 10). Ultimately, we also found that exposing the resin to TFA : CH_2_Cl_2_ : TIPS 45 : 50 : 5 for 10 minutes afforded **15** in 52% crude purity (Fig. 5a). Since TIPS is less efficient as a reducing agent compared to TES, a shorter cleavage time coupled with a 2-fold dilution of the cleavage cocktail with CH_2_Cl_2_ was able to provide **15** without reduction of *N*-imino glycines. Gratifyingly, hydrazone exchange was not observed upon dilution of the crude peptoids with 0.1% FA in 1 : 1 H_2_O : MeCN or phosphate buffer (pH 7). Cleavage from the resin with 95 : 5 TFA : TES, gave the desired product (**16**) with all five imines reduced in high crude purity (Fig. 5b), confirming both the utility of hydrazone submonomers in peptoid synthesis and the generality of the TFA : TES cleavage conditions for reducing multiple different hydrazones.

### Conformational analysis of *N*-imino- and *N*-alkylamino glycine monomers

#### X-ray crystallography

**Scheme 4:**
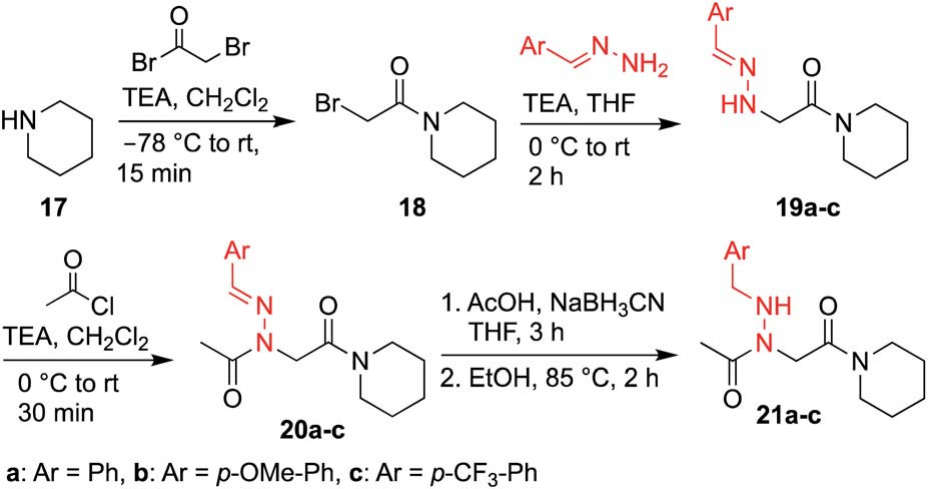
Synthesis of peptoid monomers **20** and **21**.

While hydrazone submonomers offer advantages in terms of side chain diversity and hydrogen bonding capabilities, they also exhibit important structure-inducing properties. Heteroatom substitution on the backbone nitrogen in both peptoids^8–10^ and peptides^20^ has been shown to induce the *trans*-amide bond geometry, presumably due to electronic repulsion between the lone pairs of the side chain heteroatom and backbone carbonyl group of the adjacent residue when *ω* = 0°. To confirm this trend, model compounds **20a–c** and **21a–c** were synthesized in solution using similar submonomer peptoid synthesis methods^9^ (Scheme 4), and their conformational properties were studied using NMR spectroscopy and X-ray crystallography.

**Fig. 6:**
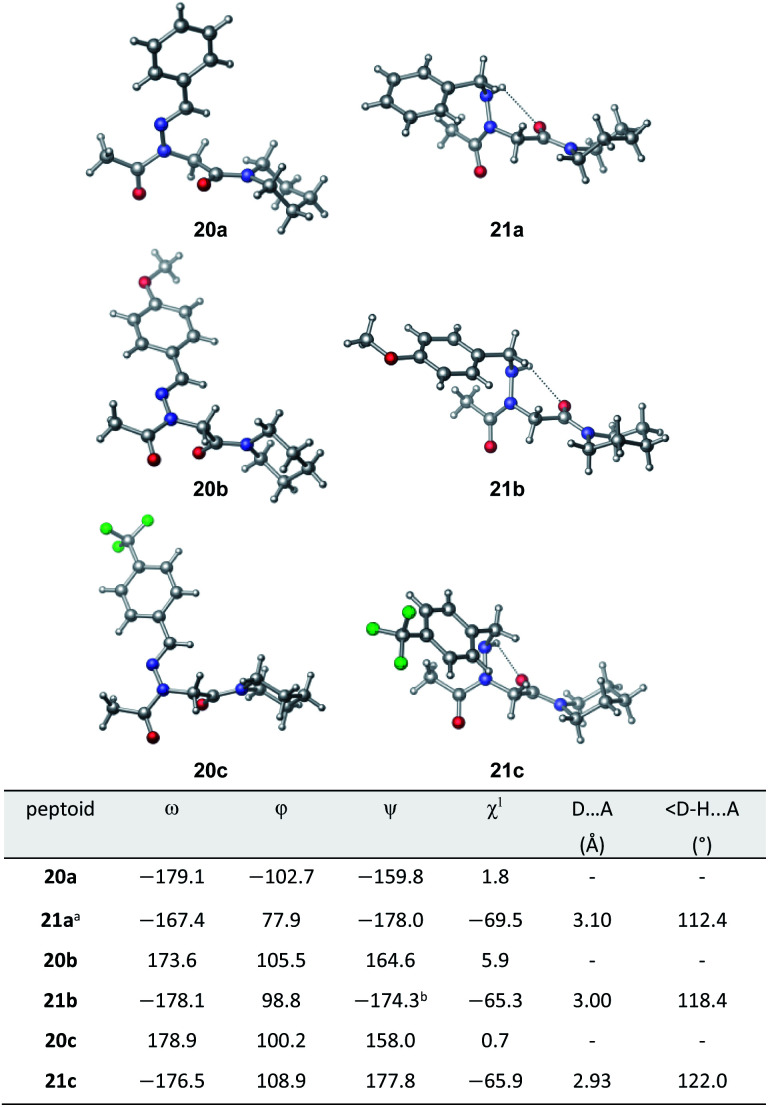
X-ray structures of **20** (left) and **21** (right).^a^ A second structure with opposite dihedral angles was present in the unit cell. ^b^ Disorder is present in the piperidinyl region. In the minor conformation, *ψ* = 150.6.

Vapor diffusion of hexanes into ethyl acetate solutions of **20a–c** and **21a–c** provided crystals of all compounds, which adopted *trans*-amide bond configurations in the solid state in all cases (Fig. 6). Reduction of the C=N double bond in **20** to give **21** caused most notable changes in the side chain chi (*χ*^1^) dihedral angle, which pivoted from an average of 2.8° to −66.9°. In contrast, all backbone dihedral angles remained within 30°. Moreover, for **21a–c**, distances of 2.93–3.10 Å between the side chain nitrogen (D) and carbonyl group (A) of the same residue with D–H⋯A angles of 112.4–122.0° revealed the presence of new intramolecular hydrogen bonds between the side chain NH and the *C*-terminal amide carbonyl.^21^ In contrast, no hydrogen bond was observed in *N*-acyl hydrazide monomers (Fig. 1b, type **H**), where the side chain chi (*χ*^1^) dihedral angle was closer to 90°.^10*a*^

#### NMR analysis

**Scheme 5:**
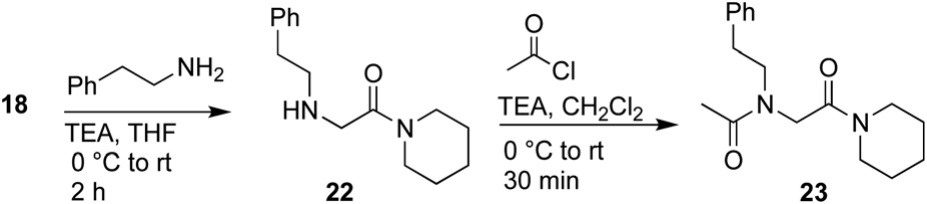
Synthesis of control monomer **23**.

**Fig. 7:**
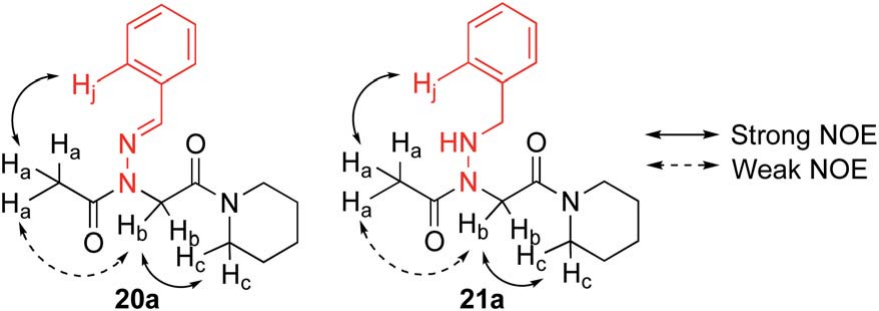
Observed NOE interactions in monomers **20a** and **21a**.

To study the conformational properties of *N*-imino- and *N*-alkylamino glycines in solution, NMR analyses were also conducted. Compound **23**, which lacks a nitrogen atom in the side chain but maintains the phenyl ring two atoms away from the backbone nitrogen, was synthesized as a control for conformational analysis in solution (Scheme 5). ^1^H NMR analyses in CDCl_3_, CD_3_CN, and CD_3_OD revealed a single major conformational isomer for **20a** and two conformational isomers for **21a** and **23** (ESI†). NOESY spectra were acquired for **20a** and **21a** as representative examples, and provided evidence for a *trans*-amide geometry in solution in both cases (Fig. 7), in accordance with what was observed by X-ray crystallography. Specifically, at 10 mM in CD_3_OD, NOE cross-peaks were present between the acetyl group (CH_3_) and the side chain phenyl ring for both **20a** and **21a** (ESI†), which is consistent with *trans*-amide configurations.^9^ However, a weak cross-peak was also observed between the acetyl group (H_a_) and the backbone methylene protons (H_b_) in both cases. While this is typically unexpected for the *trans*-amide isomer,^7*a*^ such weak NOEs have also been observed in at least one other *trans*-inducing peptoid monomer.^9^ Compared to the NOE cross peak between the backbone methylene protons (H_b_) and the piperidinyl protons (H_c_), which are *cis* to one another, integration of the acetyl/backbone methylene cross-peak was found to be 13-fold and 8-fold less intense for **20a** and **21a**, respectively.||||Similar analyses were conducted in ref. 9. The weak NOE observable between the acetyl CH_3_ and the backbone methylene is consistent with the atomic distance of 4.32–4.37 Å between the two protons detected by X-ray crystallography, which lies near the limit for NOE signal detection.

*K*_*cis*/*trans*_ values were determined for **20a**, **21a** and **23** in CDCl_3_, CD_3_CN, and CD_3_OD (Table 2). While the presence of a minor conformational isomer was not detected by ^1^H NMR for *N*-imino glycine **20a** (*K*_*cis*/*trans*_ < 0.05), in the case of **21a**, the equilibrium favored the *trans* isomer in all three solvents (*K*_*cis*/*trans*_ 0.13–0.17). ^1^H NMRs collected over a range of temperatures between −35 °C and 45 °C in CD_3_OD did not lead to the appearance of a second peak in the acetyl region for **20a**, or cause the two peaks to coalesce in the case of **21a** (ESI†). Control peptoid **23** was also found to favor the *trans* isomer in all three solvents, albeit to a lesser extent. When comparing *K*_*cis*/*trans*_ values with other peptoid residues that favor the *trans*-amide bond geometry, *N*-imino glycine derivative **20a** appears to (1) induce the *trans*-amide isomer more strongly than *N*-aryl glycines,^2*d*,7*a*^ and (2) exhibit similar behavior to *N*-alkoxy glycines, where no minor isomers are detected by ^1^H NMR.^8,9^ However, *N*-alkylamino glycine derivative **21a** is slightly less *trans*-amide inducing compared to *N*-aryl glycines.

**Table 2:** Average *K*_*cis*/*trans*_ values in compounds **20a**, **21a**, and **23**

	^1^H-NMR (CDCl_3_)		^1^H-NMR (CD_3_CN)		^1^H-NMR (CD_3_OD)	
	*K*_*cis*/*trans*_ a	Δ*G*_*cis*/*trans*_ (kcal mol^−1^)	*K*_*cis*/*trans*_ a	Δ*G*_*cis*/*trans*_ (kcal mol^−1^)	*K*_*cis*/*trans*_ a	Δ*G*_*cis*/*trans*_ (kcal mol^−1^)
**20a** b	<0.05	—	<0.05	—	<0.05	—
**21a**	0.13 ± 0.01	1.22 ± 0.06	0.17 ± 0.01^c^	1.05 ± 0.05	0.16 ± 0.01	1.07± 0.05
**23**	0.26 ± 0.05^d^	0.80 ± 0.11	0.72 ± 0.03^d^	0.19 ± 0.02	0.44 ± 0.01^e^	0.48 ± 0.02

a Average *K*_*cis*/*trans*_ and Δ*G*_*cis*/*trans*_ values were calculated using values from 4 different concentrations in each solvent (ESI, Tables S11 and S12).

b No additional peaks for a minor conformer was detected for **20a** in any solvent or concentration tested.

c For peptoid **21a** in CD_3_CN, the solvent peak has the same ppm shift as the acetyl group; *cis*/*trans* ratios were measured using the backbone methylene peaks only.

d Shoulder begins to appear that may represent different rotamers, but are not fully resolved; *cis*/*trans* ratios were measured using the backbone methylene peaks only.

e Shoulder begins to appear that may represent different rotamers, but are not fully resolved; *cis*/*trans* ratios were measured using the acetyl peaks only.

In addition to favoring the *trans-*amide bond, reduced analog **21a** has the potential for inter- and intramolecular hydrogen bonding, implicating the side chain hydrogen-bond donor (NH). To probe this unique hydrogen-bonding capability, we monitored the NH chemical shift as a function of concentration, and conducted variable temperature (VT) experiments. Little variation was observed in the NH chemical shift signal for **21a** in CDCl_3_ (Δ*δ* = 0.01) over a concentration range of 1–100 mM. This suggests that there is intramolecular hydrogen bonding in solution,^7*b*^ similar to what was observed in the solid state (ESI, Fig. S26†). Moreover, VT NMR between 25–55 °C in DMSO-d_6_ resulted in a chemical shift/temperature coefficient of −0.59 ppb K^−1^, a value that is consistent with solvent shielding (ESI, Fig. S27†).^22^ Because most peptoid monomers lack hydrogen bond donors, these *N*-alkylamino glycine monomers should exhibit unique properties that could be complementary to *N*-alkoxy glycines^8^ as hydrogen-bond acceptors. Notably, this *trans-*inducing monomer pair differ by a single nitrogen to oxygen atom substitution, which may promote productive side chain packing interactions.

#### Computational studies

**Fig. 8:**
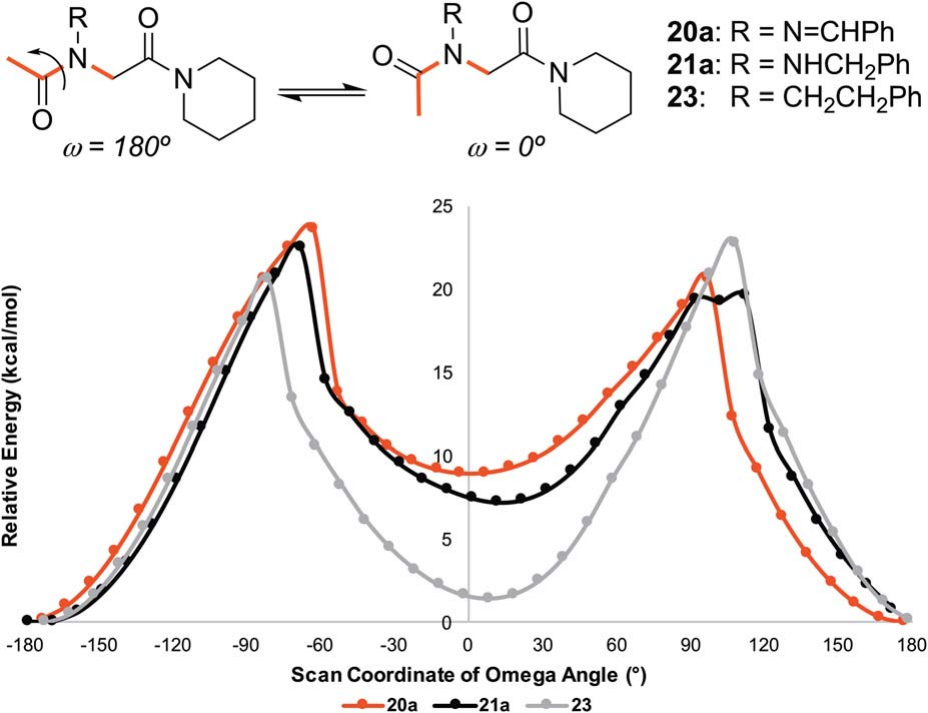
Relative energy for relaxed potential energy scan of optimized structures about the *ω* dihedral angle for monomers **20a**, **21a**, and **23** reported at the B3LYP/6-31G(d,p)^25^ level of theory. See ESI† for more computational details.

Theoretical calculations performed using the Gaussian 16 ^23^ implementation of B3LYP^24^ density functional theory confirmed the *trans*-inducing capabilities of *N*-imino- and *N*-alkylamino glycine residues. The structures of **20a** and **21a** were generated using the coordinates from their respective X-ray crystal structures, and **23** by editing the crystal structure of **20a** in GaussView. Relaxed potential energy scans were performed about the *ω* dihedral angle (Fig. 8). Energy minima were optimized at the same level of theory but also included the D3 version of Grimme's empirical dispersion correction.^26^ Energies were calculated at the same level of theory with the 6-311+G(2d,p)^27^ basis set and included solvation corrections in acetonitrile *via* the PCM method (Table 3).^28^

**Table 3:** Calculated^a^ energy differences between *cis*- and *trans*-isomers

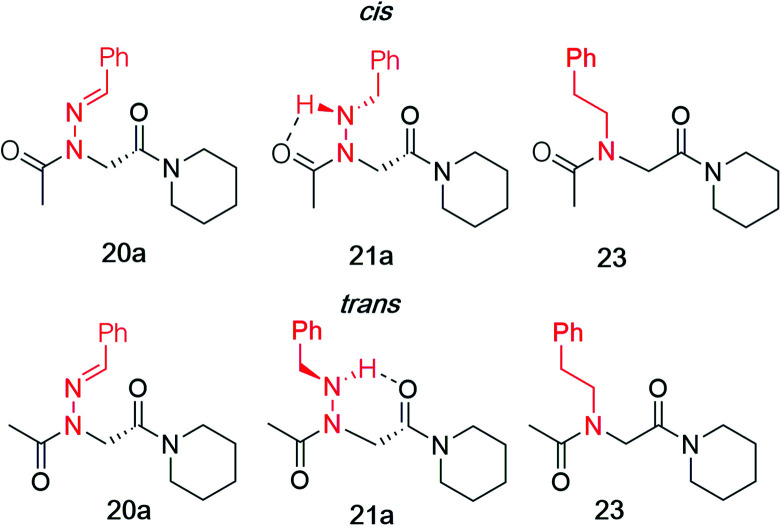			
Molecule	*ω* Dihedral angle (°)		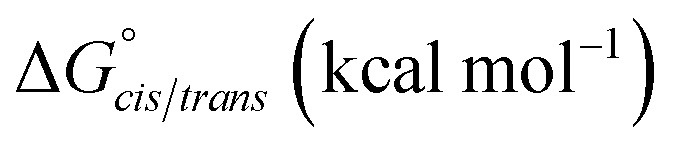
	*cis*	*trans*	
**20a**	−7.61	179	5.6
**21a**	−4.94	178	2.9
**23**	−10.6	174	0.67

a Energy calculations for each isomer were performed using B3LYP-D3/6-311+G(2d,p)^27^ level of theory with the PCM solvation corrections method (in acetonitrile) as implemented in Gaussian 16. A potential energy surface scan for rotation around the C–N–N–H dihedral angle was performed for the *cis* conformer of **21a**, which identified a second minima with hydrogen bonding to the acetyl carbonyl. See computational methods in ESI for more details.

**Fig. 9:**
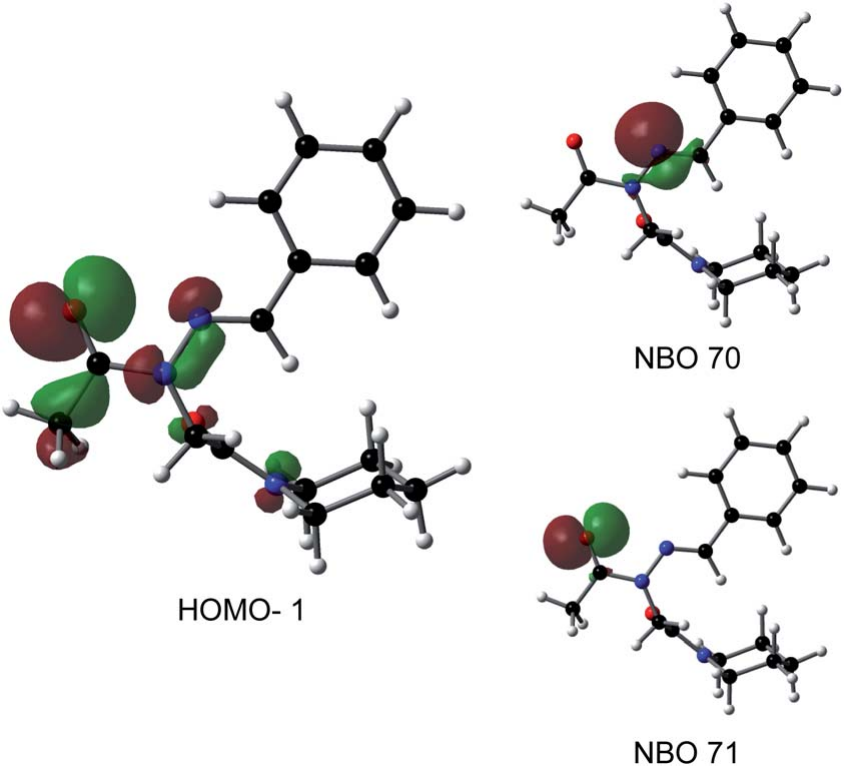
Molecular and natural bonding orbitals (NBOs) for **20a**. Orbitals are shown at 0.045 isocontours.

Consistent with experimental data, preference for the *trans* isomer follows the order **20a***>***21a** > **23**. The trends in free energies, and consequently, the preference for the *trans* isomer, can be explained by electron–electron repulsion between the lone pair on the *N*-imino nitrogen and the acetyl oxygen in **20a**. This can be observed in HOMO-1 (Fig. 9). NBOs 70 and 71 show that these orbitals originate from a lone pair on the imino nitrogen and acetyl oxygen respectively. When the nitrogen atom is removed in **23**, electron–electron repulsion is reduced and there is only a small preference for the *trans* isomer. This results in 
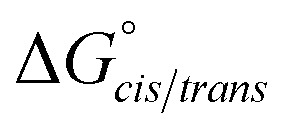
 of 5.6 kcal mol^−1^ for **20a** and 0.67 kcal mol^−1^ for **23** respectively. In **21a**, the presence of a hydrogen bond between the acetyl oxygen and the amino N–H bond (N–H⋯O = 2.24 Å) reduces the energetic difference between the *cis* and *trans* isomers. Thus, 
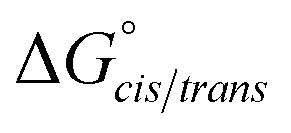
 for **21a** is smaller (2.9 kcal mol^−1^) compared to **20a** (5.6 kcal mol^−1^). Taken together, these results support that *N*-imino glycines and *N*-alkylamino glycines have a preference for the *trans*-amide configuration, which should allow for secondary structure stabilization in longer oligomers.

## Conclusions

In sum, we report here the use of hydrazones as new submonomers for solid phase peptoid synthesis, providing easy access to both *N*-imino- and *N*-alkylamino glycine-containing peptoids. A wide array of aliphatic and (hetero)aromatic side chains can be easily accessed using various hydrazone submonomers, pre-synthesized from simple condensation reactions with hydrazine and aldehydes. The *N*-imino glycine-containing peptoids can be cleaved from the solid support using standard TFA/H_2_O conditions when there is only one *N*-imino glycine or when all the *N*-imino glycines in the sequence are the same, and with TFA/TIPS/CH_2_Cl_2_ for 10 minutes or TFA/phenol when the peptoid contains multiple different *N*-imino glycines. This is essential to prevent undesired hydrazone exchange during resin cleavage. Alternatively, resin-bound *N*-imino glycine-containing peptoids can undergo a one-pot reduction/cleavage reaction using TFA/TES to afford the *N*-alkylamino glycine-containing peptoid. Both residues were found to exhibit a preference for the *trans*-amide isomer, increasing the toolbox of structure-inducing monomers for future peptoid design and applications. Moreover, the hydrogen-bonding capacity of *N*-alkylamino glycines could be beneficial in biological and materials applications, with potential to rigidify secondary structures and intermolecular interactions.

## Author contributions

C. P. and C. D. conceived and planned the experiments; C. P., C. D., and E. A. I. conceived and planned the computational studies; synthesis and conformational analysis by X-ray crystallography and NMR spectroscopy was carried out by C. D., B. L., and A.R. with input from C. P.; computational studies were performed by E. A. I., C. D., and B. L. with training and input from E. A. I.; all authors provided input on the manuscript.

## Conflicts of interest

There are no conflicts to declare.

## Supplementary Material

SC-012-D1SC00717C-s001

SC-012-D1SC00717C-s002

SC-012-D1SC00717C-s003
