# Supplementary material for: Submonomer synthesis of peptoids containing trans-inducing N-imino- and N-alkylamino-glycines
Source: Chem Sci. 2021 May 10;12(24):8401–10. doi: 10.1039/d1sc00717c (PMC8221195; doi:10.1039/d1sc00717c)
Supplement: SC-012-D1SC00717C-s002 [file SC-012-D1SC00717C-s002.pdf]

Electronic Supplementary Material (ESI) for Chemical Science.
This journal is © The Royal Society of Chemistry 2021

Davern, C.M.; Lowe, B.D.; Rosfi, A.; Ison, E.A.; Proulx, C.

SUPPORTING INFORMATION

Submonomer Synthesis of Peptoids Containing *trans*-Inducing *N*-Imino and *N*-Alkylamino-glycines

Carolynn M. Davern, Brandon D. Lowe, Adam Rosfi, Elon A. Ison, and Caroline Proulx*

Department of Chemistry, North Carolina State University, Raleigh, NC 27695-8204, USA

e-mail: cproulx@ncsu.edu

Table of Contents of the Supporting Information

Title, authors and address S1

Table of Contents S2

A. General Methods

General and Reagents S5

B. Experimental Procedures and Characterization Data

1. Manual solid phase submonomer peptoid synthesis S6
2. Automated solid phase submonomer peptoid synthesis S6
3. Cleavage test of resin-bound peptoids S7
4. Hydrazone submonomer synthesis S7-11
   Figure S1. Test tripeptoid made using hydrazone without aqueous workup S9
   Figure S2. Test tripeptoid made using hydrazone with aqueous workup S10
   Table S1. Attempted optimization of *in situ* hydrazone synthesis S11
5. Hydrolysis of *N*-imino glycine residues is more prevalent when at the *N*-terminus S11-13
   Figure S3. Hydrolysis of *N*-terminal *N*-imino glycine residue S12
   Figure S4. No hydrolysis of embedded *N*-imino glycine residue S13
6. Comparing chloroacetic acid *vs* bromoacetic acid S14-16
   Table S2. Nucleophilicity of benzaldehyde hydrazone in peptoid trimer synthesis S14
   Figure S5. Test tripeptoid using bromoacetic acid/DIC S14
   Figure S6. Test tripeptoid using chloroacetic acid/DIC but no KI in displacement S15
   Figure S7. Test tripeptoid using chloroacetic acid/DIC and KI in displacement S16
7. Optimization of hydrazone displacements S17
   Table S3. Optimization of the synthesis of pentamer peptoid **11c,d,i/12c,d,i** S17
8. Stability studies of tripeptoids **13a-d** and **S13a-d** S18-21
   Figure S8. % hydrolysis of **13a-d** and **S13a-d** in pH 7 buffer S18
   Figure S9. % hydrolysis of **13a-d** in 0.1% TFA vs. 0.1% FA S19
   Figure S10. Decomposition of **13c** over 8 hours S20
   Figure S11. % hydrolysis of **13a-d** in presence of Cys or Lys S21
9. Hydrazone exchange using hexapeptoid model compounds S22-28
   Table S4. Ratios of exchange products in hexapeptoids **S16-18** S22
   Figure S12. Representative hexapeptoid with Cleavage III S23-24
   Figure S13. Representative hexapeptoid with Cleavage VIII S25
   Figure S14. Representative hexapeptoid with Cleavage X S26
   Figure S15. Representative hexapeptoid **S16** stability to pH 7 and 0.1% FA S27
   Figure S16. Representative hexapeptoid **S18** stability to pH 7 and 0.1% FA S28
10. Peptoid characterization data S29-34
    Table S5. Characterization data for peptoids **4a-4h** S29
    Table S6. Characterization data for peptoids **8, 9, S19-22** S30
    Table S7. Characterization data for peptoids **11a-11l, 12a-12l** S31
    Table S8. Characterization data for peptoids **13a-13d** S32
    Table S9. Characterization data for peptoids **S16-18** S33
    Table S10. Characterization data for peptoids **15** and **16** S34
11. Representative protocols for synthesis of model peptoids in solution (**18-23**) S35-38

12. Variable solvent and concentration NMRs for **20a, 21a,** and **23** S39-43
Figure S17. ^{1}H NMR (600 MHz, $CDCl_3$) at variable concentration of **20a** S39
Figure S18. ^{1}H NMR (600 MHz, CD_3OD) at variable concentration of **20a** S39
Figure S19. ^{1}H NMR (600 MHz, CD_3CN) at variable concentration of **20a** S40
Figure S20. ^{1}H NMR (600 MHz, $CDCl_3$) at variable concentration of **21a** S40
Figure S21. ^{1}H NMR (600 MHz, CD_3OD) at variable concentration of **21a** S41
Figure S22. ^{1}H NMR (600 MHz, CD_3CN) at variable concentration of **21a** S41
Figure S23. ^{1}H NMR (600 MHz, $CDCl_3$) at variable concentration of **23** S42
Figure S24. ^{1}H NMR (600 MHz, CD_3OD) at variable concentration of **23** S42
Figure S25. ^{1}H NMR (600 MHz, CD_3CN) at variable concentration of **23** S43
13. Analysis of peptoid **21a** S44
Figure S26. NMR chemical shift of the NH proton of the side chain of **21a** S44
Figure S27. Temperature coefficient of **21a** S44
14. $K_{cis/trans}$ values S45-46
Table S11. $K_{cis/trans}$ values of **20a, 21a,** and **23** in variable solvents/concentrations S45
Table S12. $K_{cis/trans}$ values of **20a-c, 21a-c,** and **23** at 100 mM in $CDCl_3$ S46
15. Variable temperature NMRs for peptoids **20a** and **21a** S47-53
Figure S28a. Variable temperature ^{1}H NMRs for peptoid **20a** (CD_3OD, H_a) S47
Figure S28b. Variable temperature ^{1}H NMRs for peptoid **20a** (CD_3OD, H_b) S48
Figure S29a. Variable temperature ^{1}H NMRs for peptoid **21a** (CD_3OD, H_a) S49
Figure S29b. Variable temperature ^{1}H NMRs for peptoid **21a** (CD_3OD, H_b) S50
Figure S29c. Variable temperature ^{1}H NMRs for peptoid **21a** (CD_3CN, H_b) S51
Figure S29d. Variable temperature ^{1}H NMRs for peptoid **21a** (DMSO-d6, H_a) S52
Figure S29e. Variable temperature ^{1}H NMRs for peptoid **21a** (DMSO-d6, H_b) S53
16. NOESY NMRs of **20a, 21a,** and **23** S54-56
Table S13. NOESY parameters S54
Figure S30. 2D NOESY NMR of peptoid **20a** S54
Figure S31. 2D NOESY NMR of peptoid **21a** S55
Figure S32. 2D NOESY NMR of peptoid **23** S56
17. Computational studies S57
Figure S33. Scan about the χ dihedral angle of the *cis* conformer of **21a** S57

C. X-ray crystallographic data

Figure S34. X-ray crystal structures of peptoids **20a-c** and **21a-c** S58
Table S14. Dihedral angles of peptoids **20a-c** and **21a-c** S59
Table S15. Hydrogen bond geometry (Å) for compound **21a-c** S59
Table S16. Crystal data and structure refinement of peptoids **20a-c** and **21a-c** S60

D. NMR Characterization Spectra

^{1}H and ^{13}C NMRs of peptoids **19a-c, 20a-c, 21a-c, 21,** and **23** in $CDCl_3$ S61-69

E. LC-MS Chromatograms

Characterization of peptoids **4a-4h** S70-74
Characterization of cleavage results of peptoid **5** with various cleavages to give **6** or **7** S75-84
Characterization of peptoids **S16-18** with Cleavage VIII S85-86

Manual and automated synthesis of oligomers with 5 hydrazones (**8, 9, S19-22**) S87-92
Characterization of crude peptoids **11a-l** and **12a-l** S93-104
Characterization for stability studies performed on tripeptoids **13a-d** S105-108
Characterization of peptoid **15** and **16** S109

F. References S110

A. General Methods

General: Polystyrene Rink Amide resin (0.78 mmol/g or 0.61 mmol/g) was purchased from Protein Technology, Inc™. Manual solid-phase submonomer peptoid synthesis was performed in disposable filter cartridges with 20µM PE frit filters and caps from Applied Separations (cat # 2413 for 3 mL filter cartridges) with gentle agitation on a Thermo Fisher vortex mixer equipped with a microplate tray. Solution draining and washing of the resin was accomplished by connecting the filter columns to a water aspirator vacuum *via* a waste trap. For heated reactions, the cartridge caps were wrapped in Teflon tape and parafilm and placed in a Fisher Scientific Ultrasonic Bath 9.5L (Model # 15337425). Analytical LCMS analyses were performed using Agilent Technologies 1260 Infinity II series LCMS Single Quad instrument with ESI ion-source and positive mode ionization, equipped with either a 5 µM, 150 x 4.6 mm C18 Vydac column purchased from Mac-Mod Analytical, Inc. (Column 1, cat # 218TP5415) or a 5 µM, 150 x 4.6 mm C18 Luna column purchased from Phenomenex Analytical, Inc. (Column 2, cat # 00F-4252-E0). A flow rate of 0.5 mL/min and varying gradients of CH_3CN [0.1% trifluoroacetic acid (TFA)] in water (0.1% TFA) over 12 minutes (total run time = 22 minutes) were used for all LCMS analyses. Automated peptoid synthesis was done using a Biotage® Syro Wave™ peptide synthesizer in 10 mL reactors with PTFE frits.

Solution phase reactions done with dry solvents were performed in oven-dried glassware under argon sealed with rubber septa and were stirred with Teflon-coated magnetic stir bars. Thin layer chromatography (TLC) was performed using Silicycle silica gel 60 F-254 precoated plates (0.25 mm) and visualized using ultraviolet light (UV) and/or submersion in aqueous ninhydrin or $KMnO_4$ staining solutions. Samples were purified using a Biotage® Isolera One, employing polypropylene cartridges preloaded with silica gel (25 micron) and were eluted with UV detection (254, 280 nm). Nuclear magnetic resonance (NMR) spectra (^{1}H, ^{13}C) were recorded on a 600 or 700 MHz Bruker spectrometer at 25 ºC unless otherwise specified. Chemical shifts are expressed in parts per million (ppm, δ scale) and are referenced to residual protium in the NMR solvent ($CHCl_3$, δ 7.26, MeOH, δ 3.31, or MeCN, δ 1.94). NMR data is presented by listing chemical shift, multiplicity (s = singlet, d = doublet, t = triplet, q = quartet, m = multiplet, br = broad), coupling constant in Hertz, and integration. Chemical shifts for ^{13}C NMR spectra are recorded in parts per million (ppm, δ scale) and are referenced to the central peak of deuterochloroform (δ 77.16). All spectra were obtained with complete proton decoupling. Infrared (IR) spectra were collected on a Thermo Scientific Nicolet iS5 FTIR instrument using attenuated total reflectance (ATR) mode and signals are reported in reciprocal centimeters (cm^{-1}). Melting points were obtained on a Mettler Toledo MP50 One Click Melting Point System.

Reagents: Triisopropylsilane (TIPS), trifluoroacetic acid (TFA), bromoacetic acid, and diisopropylcarbodiimide (DIC) were purchased from Chem Impex Int'l, Inc. *N,N*-diisopropylethylamine (DIEA), triethylamine (TEA), triethylsilane (TES), piperidine, acetyl chloride, 4-fluorobenzaldehyde, 4-chlorobenzaldehyde, 4-bromobenzaldehyde, *p*-anisaldehyde, isobutyraldehyde, 4-(trifluoromethyl)benzaldehyde, *p*-tolualdehyde, 2-methoxyethylamine, methylamine (40% in water), isobutylamine, benzylamine, aniline, 2-pyrrolecarboxaldehyde, isobutyraldehyde, butyraldehyde, chloroacetic acid, potassium iodide, and benzaldehyde were purchased from Sigma Aldrich. Bromoacetyl bromide, phenol, isopropylamine, 2-phenylethylamine, β-alanine *tert*-butyl ester hydrochloride, formic acid, and $NaBH_3CN$ were purchased from Alfa Aesar (Thermo Fisher Scientific). 2-furaldehdye, cyclopentanecarboxaldehyde, cyclohexanecarboxaldehyde, and hydroxylamine hydrochloride were purchased from Acros Organics. All chemicals were used as received without further purification.

B. Experimental Procedures and Characterization Data

1. Manual Solid Phase Submonomer Peptoid Synthesis[1]: All reactions are gently agitated on a vortex mixer equipped with a microplate tray for the designated amount of time. For the first cycle, 100 mg of Rink Amide resin (0.061-0.078 mmol) was added to a 3 mL solid phase peptide synthesis (SPPS) cartridge and swelled with DMF (1 mL) for 20 minutes. The DMF was drained, and the resin was treated with 20% piperidine in DMF solution (v/v) for Fmoc deprotection (1 mL, 2.02 mmol, 5 minutes) followed by another 15-minute incubation with fresh reagent (1 mL, 2.02 mmol). The solution was drained and the resin was washed with DMF (5 x 1 mL). Each subsequent monomer addition cycle consisted of 1) a bromoacetylation reaction and 2) a displacement step. The bromoacetylation reaction was performed by addition of a 0.6 M bromoacetic acid or chloroacetic acid solution[a] in DMF (1 mL, 0.6 mmol) and 86 µL of *N,N*-diisopropylcarbodiimide (DIC, 0.56 mmol) for 25 minutes followed by the same wash cycle as above with DMF (5 x 1 mL). Displacements were performed with 1.5 M solution of amine or hydrazone in DMF (1 mL, 1.5 mmol) for 1 hour at room temperature, unless otherwise noted.[b] This process was repeated until the desired length of peptoid was achieved. When the peptoid was finished or at a stopping point for the day, the resin was washed with CH_2Cl_2, left to dry, and stored in the fridge (if stored for a prolonged period) or in a fume hood at room temperature (if stored overnight).

[a] Chloroacetic acid was used with hydrazone submonomers possessing heterocylic side chains.
[b]When *p*-CF_3-benzaldehyde hydrazone **d** is used, the reaction time is lengthened to two hours, the concentration of the hydrazone is increased to 3.0 M, and the reaction is performed in a heated sonicator bath (60°C). When *p*-Br-benzaldehyde hydrazone **c** is used, the reaction is performed in a heated sonicator bath (60°C). When heterocyclic hydrazones **e**, **f**, or **g** are used, the reaction is performed using 1.5 M hydrazone in 1.0 M KI in DMF instead of neat DMF.

<u>Note:</u> Resin-bound peptoid should be stored after a displacement reaction and should not be stored at the dimer stage where on-resin cyclization to a diketopiperazine side product may occur.

2. Automated Solid Phase Submonomer Peptoid Synthesis: Protocol on the Biotage® Syro Wave™ Peptide Synthesizer includes similar synthesis procedures as manual synthesis. For the first cycle, 200 mg of Rink Amide resin (0.122-0.156 mmol) was swollen in DMF (3.0 mL, reaction time 30 minutes: vortex 20 s, break time 1 min, empty 45 s) prior to Fmoc deprotection by treatment with 1) 40% piperidine in DMF (v/v) (3.3 mL, reaction time 3 minutes: vortex 10 s, break time 1 min, empty 45 s) followed by 2) 40% piperidine in DMF and neat DMF (1.7 mL each, reaction time 12 minutes: vortex 10 s, break time 1 min, empty 45 s). The resin was washed with DMF (5 x 3.1 mL, reaction time 2 minutes: vortex 20 s, break time 1 min, empty 50 s). Each subsequent monomer addition cycle consisted of 1) a bromoacetylation reaction and 2) a displacement step. The bromoacetylation reaction was performed by addition of a 0.6 M bromoacetic acid solution in DMF (3.2 mL, 1.92 mmol) and a 1:1 v/v DIC: DMF solution (700 µL, 2.28 mmol) (reaction time 30 minutes: vortex 20 s, break time 1 min, empty 30 s), followed by the same wash cycle as above with DMF (6 x 3.1 mL). Displacements were performed with 1.5 M amine or hydrazone solution in DMF[a] (3.2 mL, 4.8 mmol) (reaction time 60 minutes: vortex 20 s, break time 1 min, empty 30 s), followed by another wash cycle with DMF (5 x 3.1 mL).

[a]When *p*-CF_3-benzaldehyde hydrazone **d** is used, the reaction time is lengthened to four hours and the concentration of the hydrazone is increased to 3.0 M.

3. Cleavage test of resin-bound peptoids: 200 µL of a cleavage cocktail was added to a small amount of resin in a 1 mL SPPS cartridge. Solutions must be used the same day they are made, especially those containing TES or TIPS as they lose their efficacy as reducing agents if not used right away. The cleavage solution was collected by filtering the resin through the disposable fritted cartridge into a 20 mL scintillation glass vial. The resin was washed with CH_2Cl_2 (3 x 500µL) into the same vial. The filtrate was evaporated to dryness using a Biotage® V10 evaporator and the crude peptoid was typically dissolved in 1:1 v/v MeCN: H_2O (1 mL) for analysis on the LCMS. The exact cleavage cocktail and cleavage times varied (see Table 1 in the article main text).

Cleavage I: 95/2.5/2.5 v/v/v TFA/TIPS/H_2O, 10 min.
Cleavage II: 95/2.5/2.5 v/v/v TFA/TIPS/H_2O, 2 h.
Cleavage III: 95/5 v/v TFA/H_2O, 10 min.
Cleavage IV: 95/5 v/v TFA/H_2O, 2 h.
Cleavage V: 95/5 v/v TFA/TES, 10 min.
Cleavage VI: 95/5 v/v TFA/TES, 2 h. Treating the crude peptoid with a fresh solution for an additional 2 h may be required for reduction of the *N*-imino glycine residues.
Cleavage VII: 90/10 v/v TFA/TIPS, 2 h.
Cleavage VIII: 45/5/50 v/v/v TFA/TIPS/DCM, 10 min.
Cleavage IX: 45/5/50 v/v/v TFA/TIPS/DCM, 2 h.
Cleavage X: 95/5 v/w TFA/phenol, 2 h. After evaporating filtrate to dryness, a cold ether precipitation is required to remove excess phenol before making the LCMS samples.

Note: If the N-terminal residue in an oligomer is an N-imino glycine residue, the hydrazone side chain is more susceptible to hydrolysis. Acetylation can be done on a small sample prior to test cleavage for analysis. Add 1 mL of 10% acetic anhydride in DMF per 100 mg resin for 40 minutes. Drain and wash the resin with DMF (5 x 1 mL) and DCM (5 x 1 mL) prior to the test cleavage.

4. **Hydrazone submonomer synthesis.** *The procedures for making the hydrazone submonomers are critical for reproducible results in submonomer peptoid synthesis. The hydrazones should be made immediately before use for best results.*

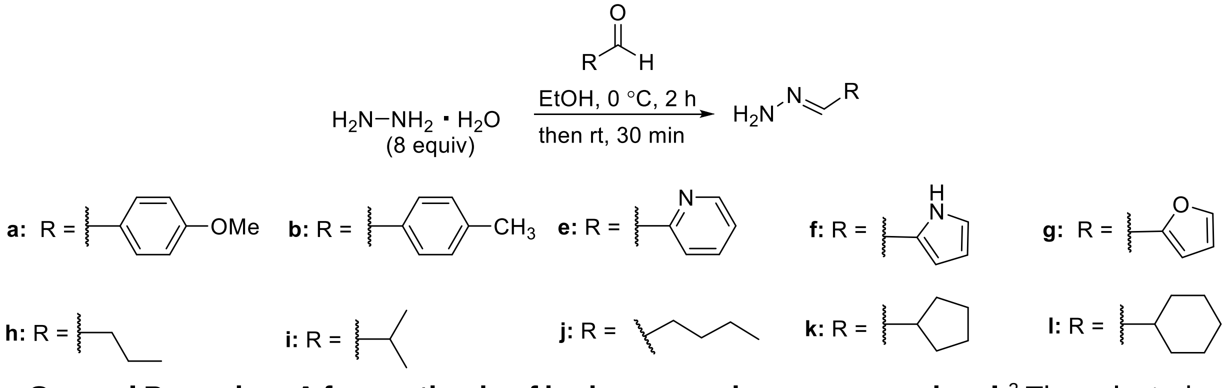

General Procedure A for synthesis of hydrazone submonomers a-b, e-l:[2] The selected aldehyde (1.0 equiv, 15 mmol) was dissolved in EtOH (4 mL) and cooled to 0 °C before being added dropwise to a solution of hydrazine monohydrate (8.0 equiv, 120 mmol, 5.6 mL) in EtOH (6 mL) at 0 °C. The reaction was stirred for 1.5 h at 0 °C before removing the ice bath and leaving the reaction stirring for an additional 30 minutes. The reaction mixture was transferred to a 250 mL separatory funnel and diluted with 75 mL CH_2Cl_2. The organic layer was washed sequentially with saturated brine (2 x 75 mL) and 5% $NaHCO_3$ (2 x 75

mL).[a] The organic layer was dried with Na_2SO_4 and the solvent was removed under vacuum. The samples were placed on high vacuum for two hours (hydrazones **a**, **b**, and **e**) or 20 minutes (hydrazones **f-l**), depending on their predicted boiling points.

[a]The number of washes was reduced to 1x each when making aliphatic hydrazones (**h-l**).

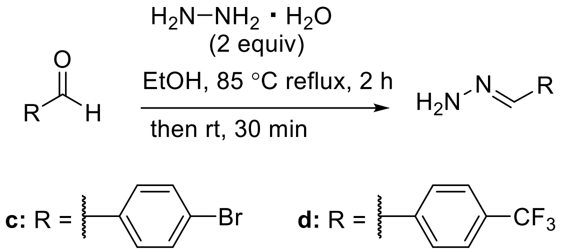

General Procedure B for synthesis of hydrazone submonomers c and d:[3] The selected aldehyde (1.0 equiv, 10 mmol) was dissolved in EtOH (10 mL). Hydrazine monohydrate (2.0 equiv, 20 mmol, 1 mL) was added in a single addition to the aldehyde solution, and the reaction mixture was heated to reflux at 85 °C for 1.5 h. The reaction mixture was cooled to room temperature and stirred for another 30 minutes before being transferred to a 250 mL separatory funnel and diluted with 75 mL of CH_2Cl_2. The organic layer was washed sequentially with saturated brine (2 x 75 mL) and 5% $NaHCO_3$ (2 x 75 mL). The organic layer was dried with Na_2SO_4 and the solvent was removed under vacuum. The samples were placed on high vacuum for two hours.

Note 1: *We found that the aqueous workup was very important. p-Bromobenzaldehyde hydrazone* ***c*** *crystallizes upon cooling to room temperature. However, bypassing the aqueous extraction and simply rinsing the crystals with H_2O causes a large side product (m/z = 630.5) to appear when the hydrazone is used in the synthesis of a tripeptoid sandwich sequence (see Figures S1 and S2).*

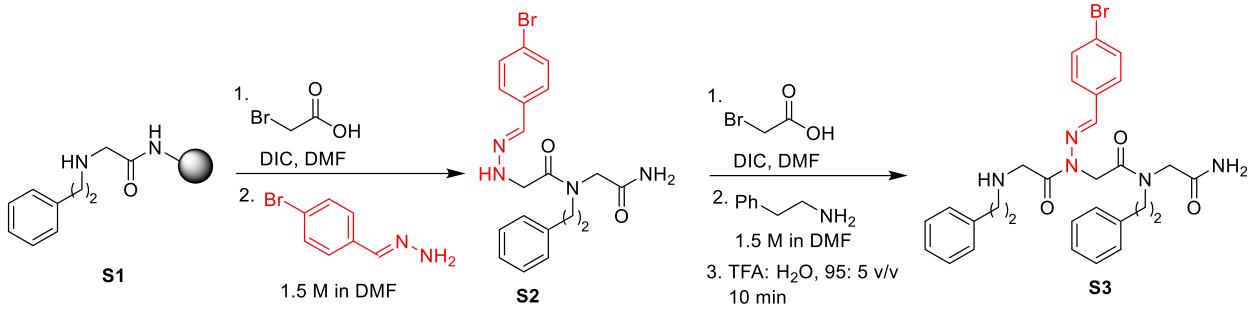

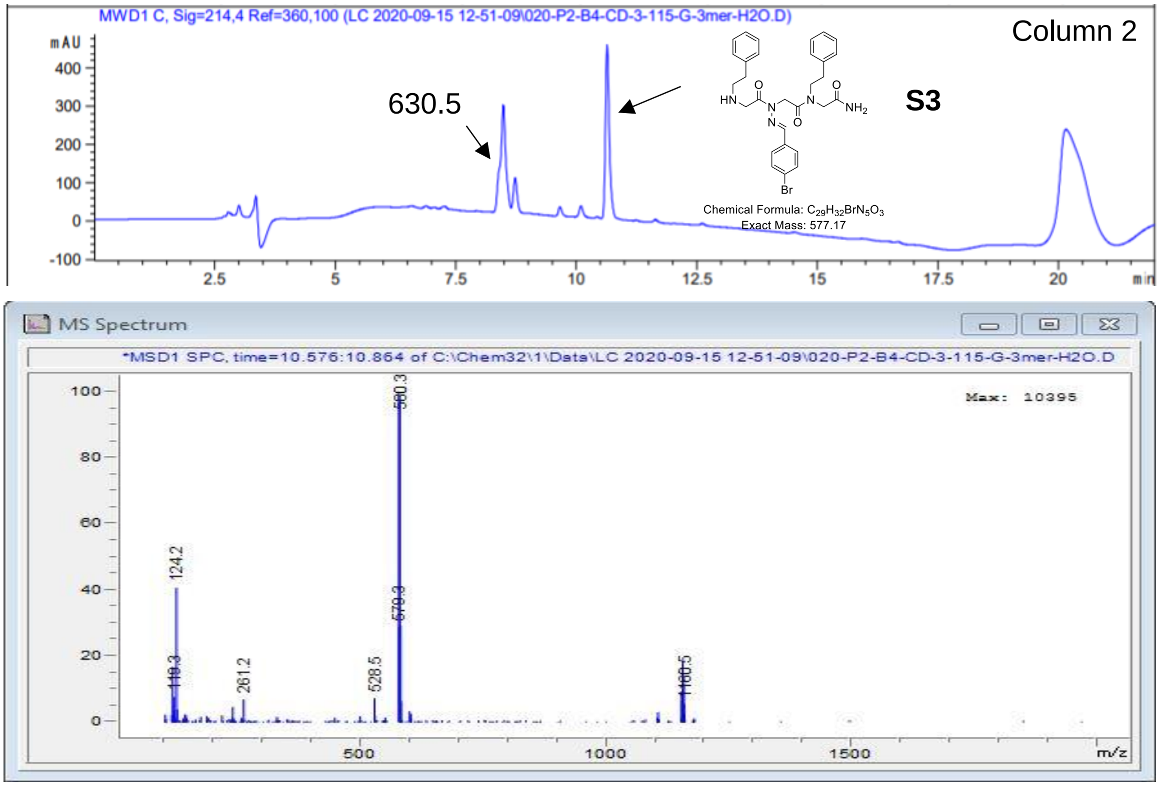

Figure S1. LCMS chromatogram at 214 nm of a test tripeptoid synthesized by using *p*-Br-benzaldehyde hydrazone crystals washed with water (no aqueous extraction).

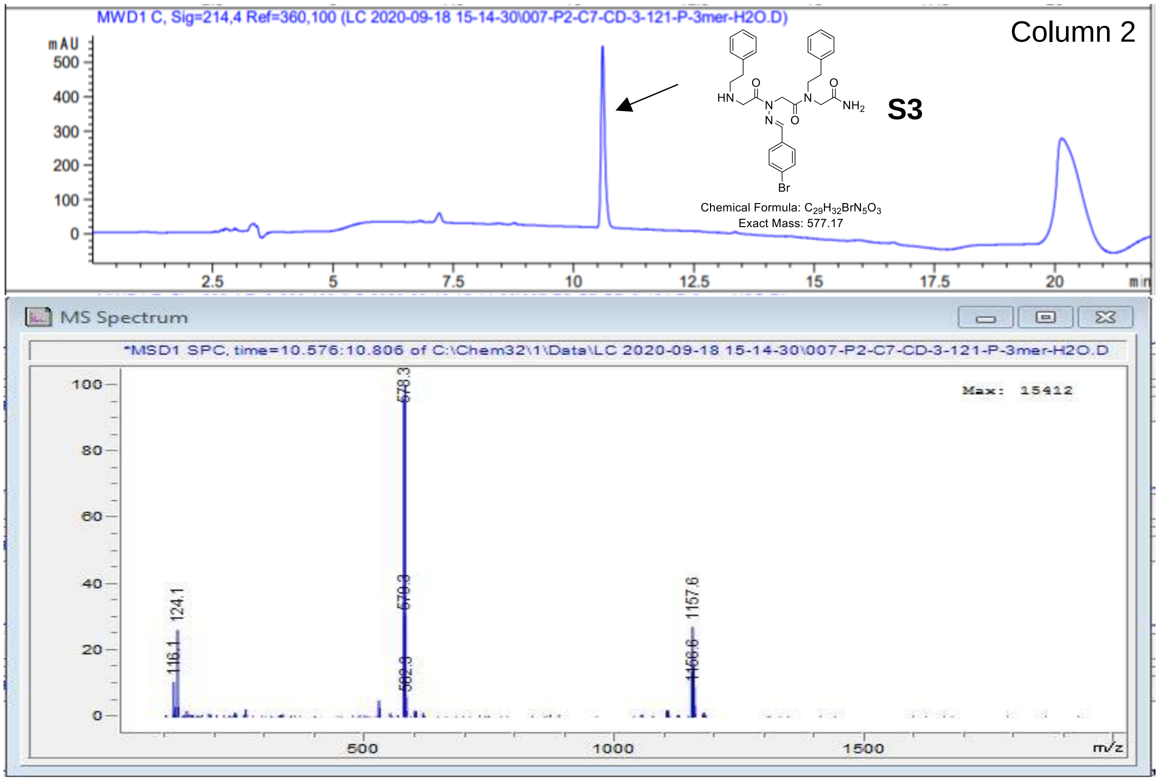

Figure S2. LCMS chromatogram at 214 nm of a test tripeptoid synthesized by using *p*-Br-benzaldehyde hydrazone that was redissolved in CH_2Cl_2 and washed with saturated brine (x 2) and 5% $NaHCO_3$ (x 2).

<u>Note 2:</u> Because of their high volatility and increased water solubility, some aliphatic hydrazones were obtained in lower yields following ***General Procedure A****. Li et al.*[4] *report synthesizing hydrazones with no aqueous workup, which was attempted but ultimately abandoned due to variability in subsequent peptoid synthesis yields (Table S1).*

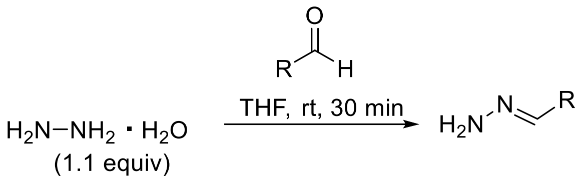

Attempted alternative procedure for the *in situ* synthesis of aliphatic hydrazones:[4] The selected aldehyde (1.0 equiv, 2 mmol) was added dropwise to a stirring solution of hydrazine monohydrate (1.1 equiv, 2.2 mmol) in THF (500 µL) for 30 min at room temperature. Na_2SO_4 (120 mg) was added to the stirring reaction and stirred for 10 more minutes. The solution was then decanted into another vial and diluted to 2 M by adding THF (~500 µL). The solutions can be stored at room temperature if necessary, but it is best to use them as soon as possible.

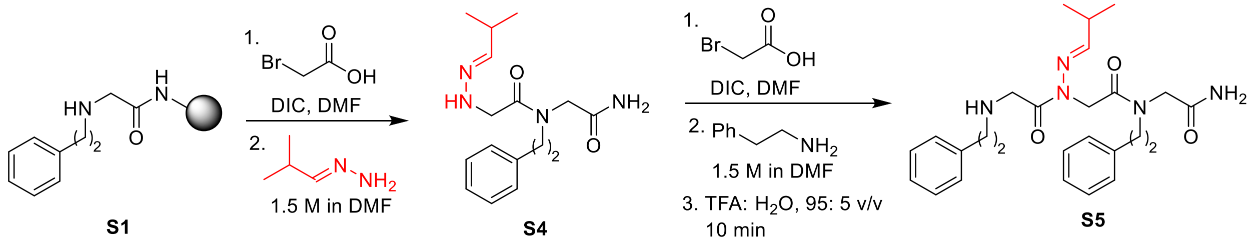

Table S1. Attempted optimization of the *in situ* isobutyraldehyde hydrazone synthesis in THF, which was subsequently used in submonomer synthesis of trimer **S5**.

	**Hydrazone synthesis parameters**				**Effect on trimer S5 synthesis**	
**Entry**	**Na_2SO_4 amount**	**aldehyde addition procedure**	**Filtration**	**Scale (mmol)**	**S5 (%)**	**unidentified byproduct, m/z 630.5 (%)**
**1**	120 mg	Dropwise	No	2	59	8
**2**	120 mg[a]	Dropwise	Yes	2	58	6
**3**					41	17
**4**					56	8
**5**	0 mg	Dropwise	No (no salt)	2	26	5
**6**	60 mg	Dropwise	No	2	56	2
**7**	400 mg	Dropwise	No	2	38	
**8**	1.2 g	Syringe pump (50 □L/min)	Yes	10	43	33
**9**	240 mg	Portioned, 6 x 61□L with 2 min between additions	Yes	4	39	12
**10**	120 mg	Single addition	Yes	2	41	17

[a]Performed in triplicate

5. Hydrolysis of *N*-imino glycine residues is more prevalent when at the *N*-terminus.

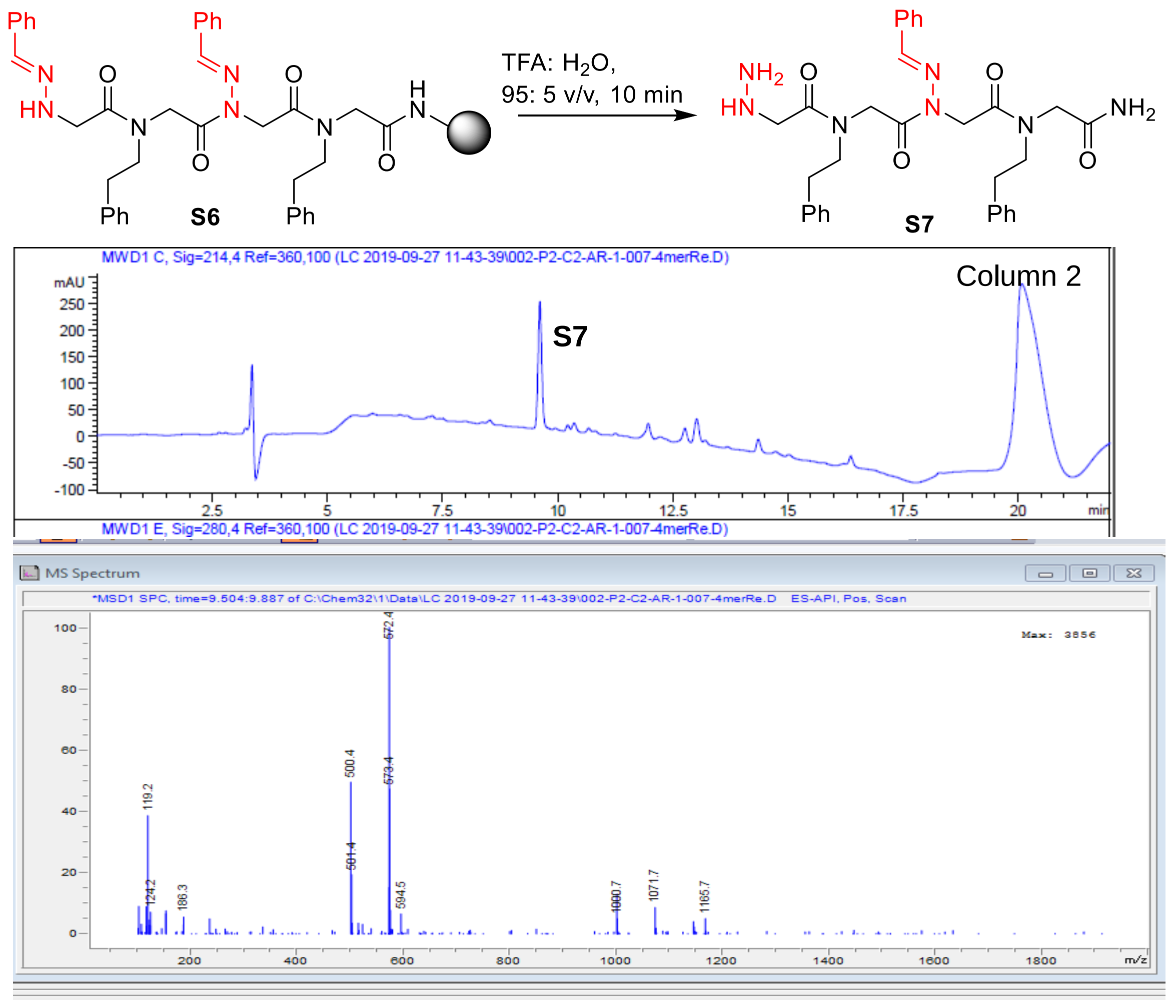

Figure S3. LCMS chromatogram at 214 nm showing hydrolysis of the *N*-terminal *N*-imino glycine residue as the major side product with Cleavage III (TFA:H_2O 95:5 v/v, 10 min).

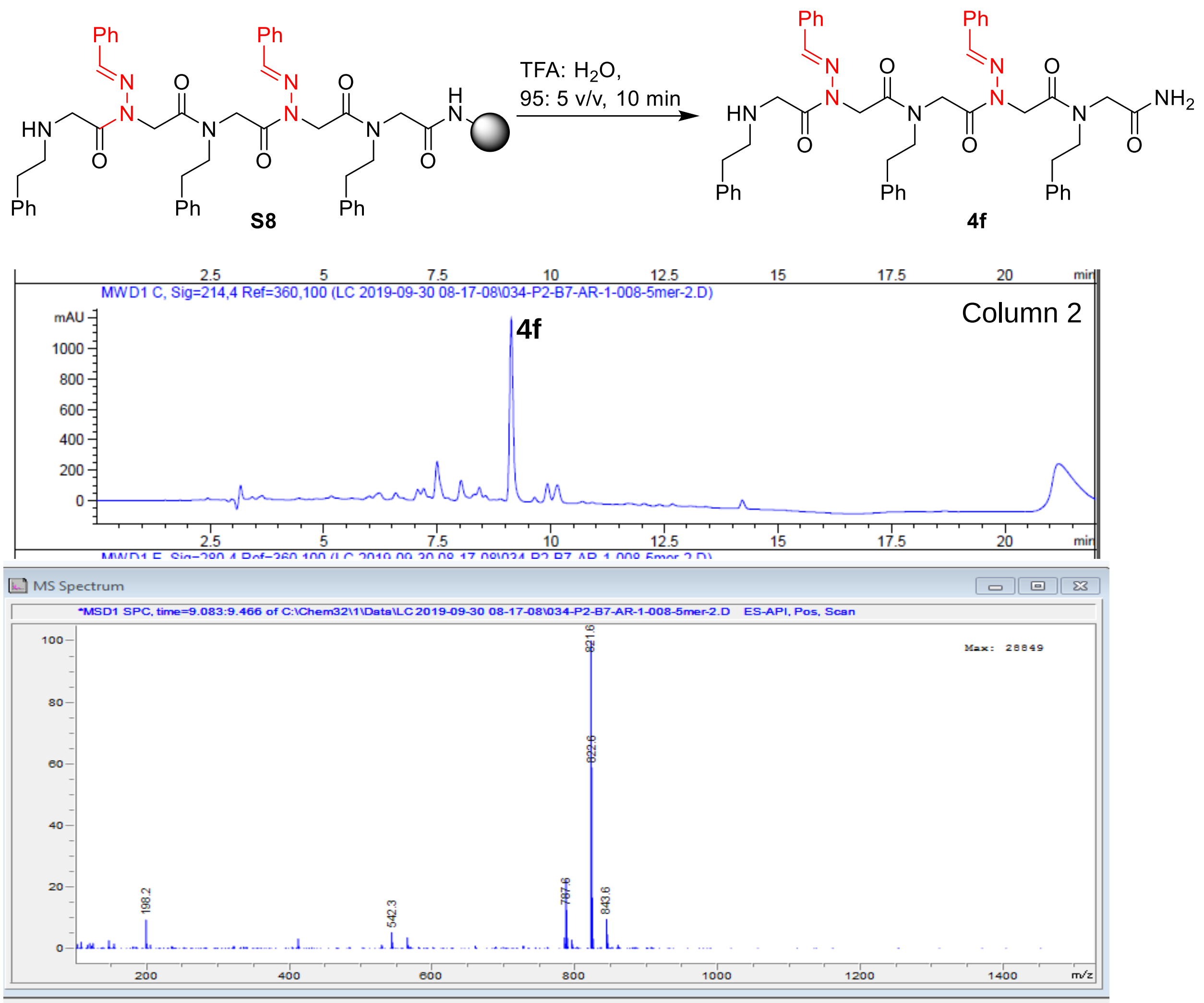

Figure S4. LCMS chromatogram at 214 nm of showing no hydrolysis of the *N*-imino glycine residues with Cleavage III (TFA:H_2O 95:5 v/v, 10 mins) when located away from the *N*-terminus.

6. Comparing chloroacetic acid *vs* bromoacetic acid. The benzaldehyde hydrazone submonomer has increased nucleophilicity compared to aniline but shows decreased nucleophilicity compared to other primary amines. Benzaldehyde hydrazone is not a good enough nucleophile to displace the chloride in the submonomer method unless KI is also present.[5]

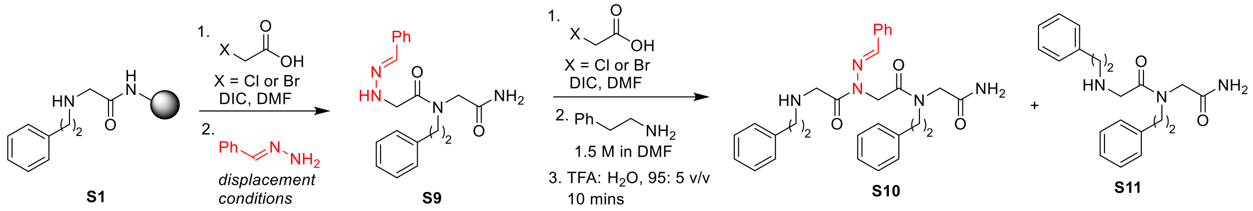

Table S2. Nucleophilicity of benzaldehyde hydrazone in peptoid trimer synthesis.

Entry	Haloacetic acid	Haloacetylation reaction time	hydrazone displacement conditions	Crude purity of S10	Crude purity of S11
**1**	Bromoacetic acid	5 min	1.5 M in DMF	91	0
**2**	Chloroacetic acid	5 min	1.5 M in DMF	9	86
**3**	Chloroacetic acid	5 min	1.5 M in 1.0 M KI in DMF	95	0

[a]Bromoacetic acid was used in the haloacetylation step to make compound **S1**. The haloacetic acid listed in the table was used for the final two submonomer additions in the respective tripeptoid

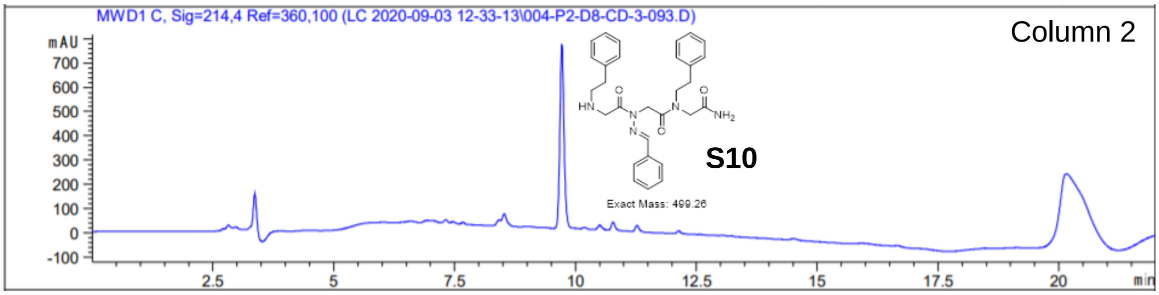

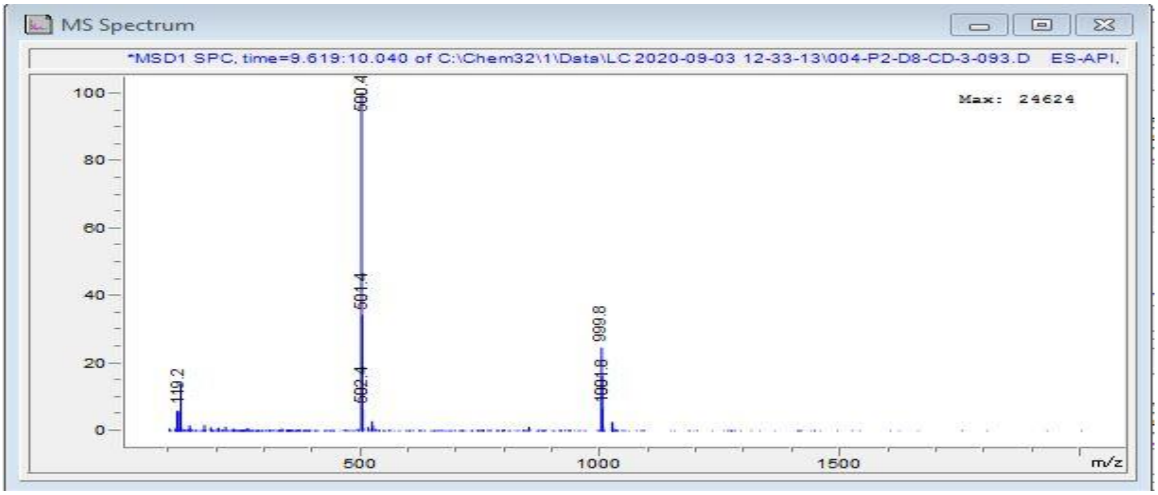

Figure S5. LCMS chromatogram at 214 nm showing desired tripeptoid obtained when using bromoacetic acid/DIC during peptoid synthesis (Table S2, entry 1).

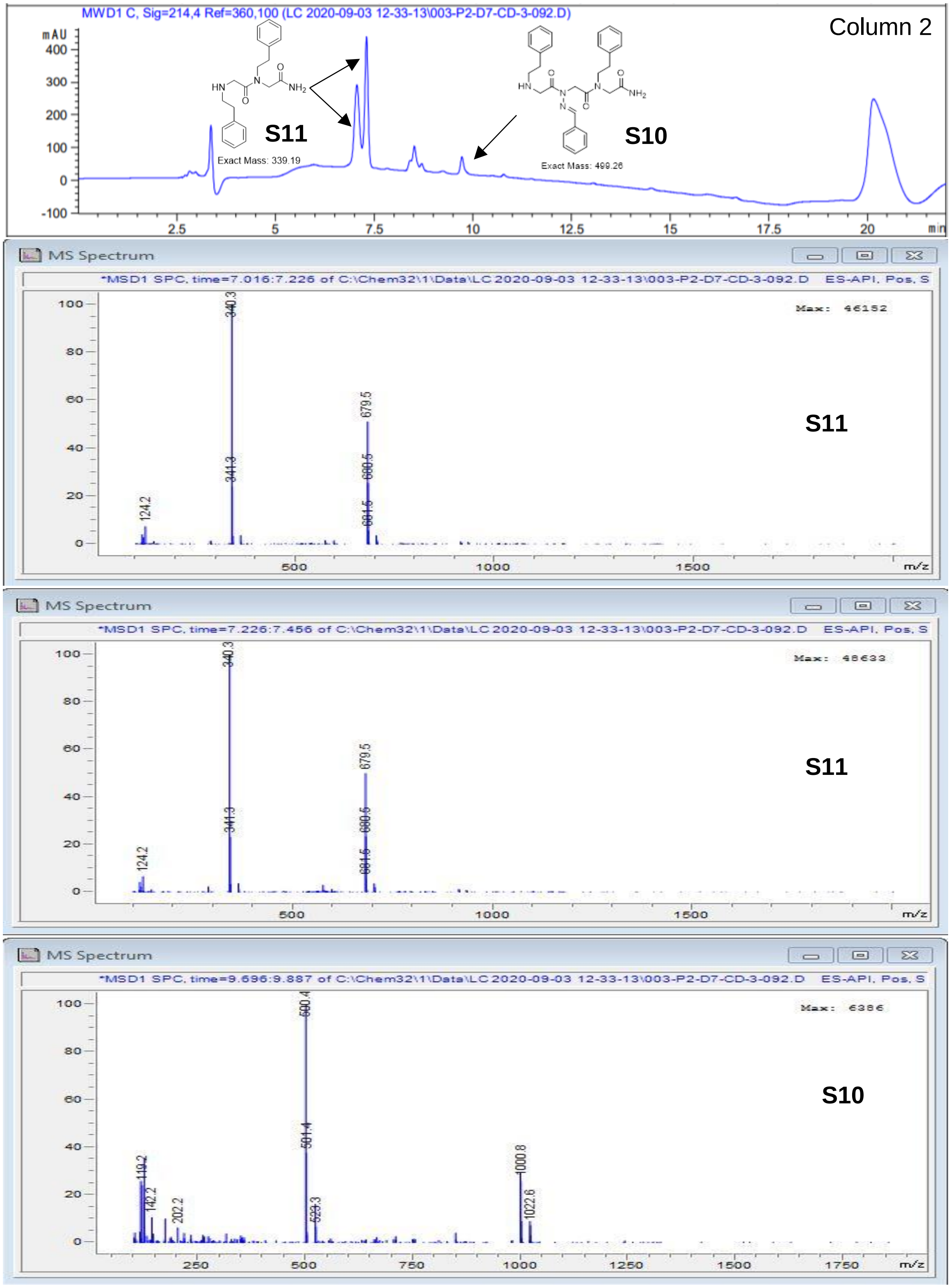

Figure S6. LCMS chromatogram at 214 nm showing undesired dipeptoid obtained when using chloroacetic acid/DIC during peptoid synthesis but no KI during the displacement with benzaldehyde hydrazone submonomer (Table S2, entry 2).

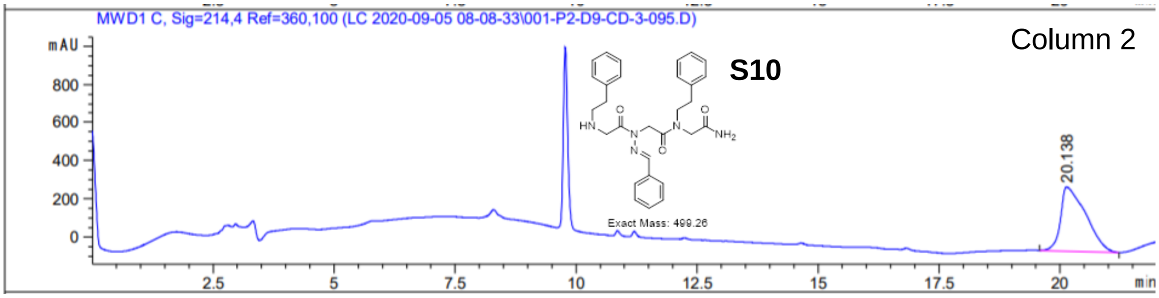

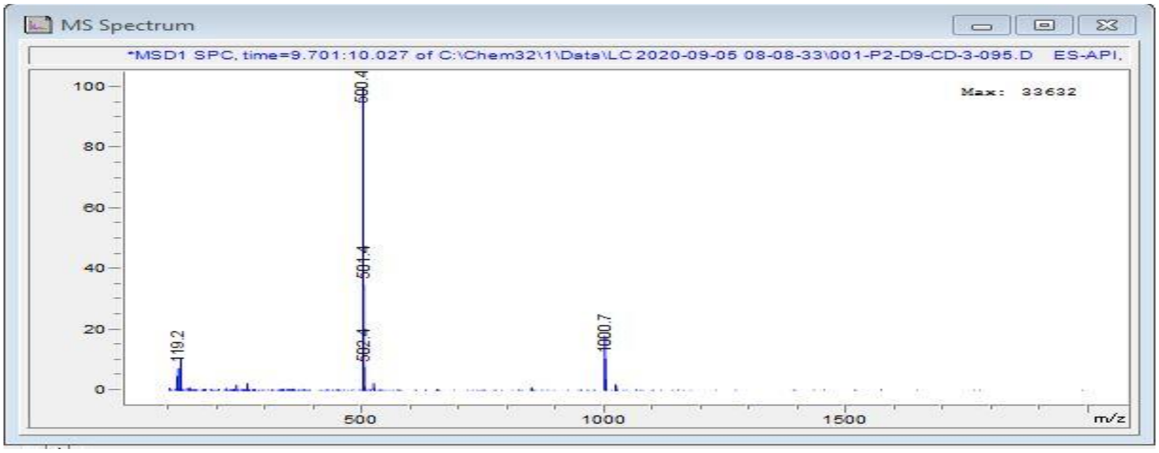

Figure S7. LCMS chromatogram at 214 nm showing desired tripeptoid obtained when using chloroacetic acid/DIC during peptoid synthesis and 1 M KI during the displacement with benzaldehyde hydrazone submonomer (Table S2, entry 3).

7. Optimization of hydrazone displacements.

Table S3. Optimization of the synthesis of pentamer peptoid **11c,d,i/12c,d,i**.

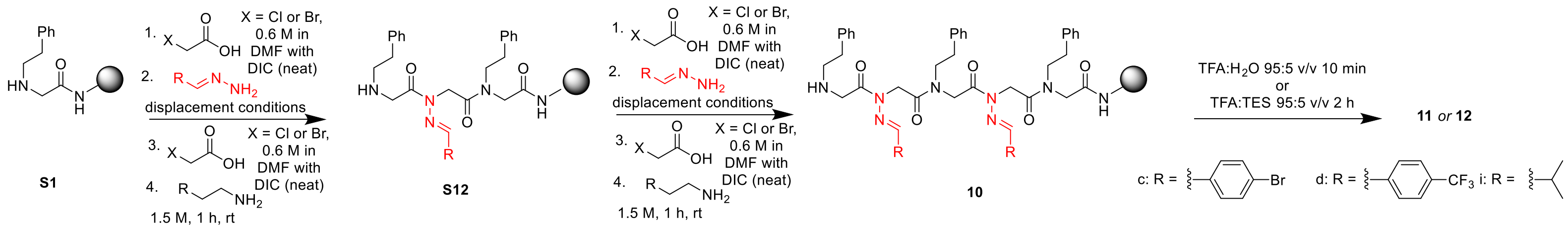

	First Hydrazone Displacement					Second Hydrazone Displacement						
	Haloacetylation		Displacement			Haloacetylation		Displacement				
R group	Haloacetic acid used	Time (min)	Conc. (M)	Time (h)	Temp.	Haloacetic acid used[a]	Length (min)	Conc. (M)	Time (h)	Temp.	Crude Purity with Cleavage I **11**[b]	Crude purity with Cleavage VI **12**[c]
**c**	BrAc	25	1.5	1	rt	BrAc	25	1.5	1	60 °C[e]	58	54
	BrAc	25	1.5	1	rt	BrAc	25	1.5	1	rt	40	NA[f]
	BrAc	25	1.5	1	rt	ClAc	5	1.5[d]	1	rt	38	NA[f]
	BrAc	25	1.5	1	rt	ClAc	5	1.5[d]	1	60 °C[e]	66	NA[f]
	BrAc	25	1.5	1	rt	BrAc	25	1.5[d]	1	60 °C[e]	58	55
	BrAc	25	1.5	1	60 °C[e]	BrAc	25	1.5	1	60 °C[e]	63	72
**d**	BrAc	25	3.0	4	rt	BrAc	25	3.0	4	rt	31	NA[f]
	BrAc	25	3.0	4	rt	BrAc	25	3.0	4	60 °C[e]	50	NA[f]
	BrAc	25	3.0	4	rt	BrAc	25	3.0	2	60 °C[e]	81	58(10)
	BrAc	25	3.0	4	rt	BrAc	25	1.5	1	60 °C[e]	80	NA[f]
	BrAc	25	3.0	2	60 °C[e]	BrAc	25	3.0	2	60 °C[e]	89	74(11)
**i**	ClAc	5	1.5[d]	1	rt	ClAc	5	1.5[d]	1	rt	20(35)	63
	BrAc	5	1.5	1	rt	BrAc	5	1.5	1	rt	19(27)	51
	BrAc	25	1.5	1	rt	BrAc	25	1.5	1	60 °C[e]	37(29)	NA[f]
	BrAc	25	1.5	1	rt	BrAc	25	1.5	1	rt	27(42)	73

[a]ClAc is chloroacetic acid and BrAc is bromoacetic acid [b]Values in parentheses represents the crude purity of the pentamer with one hydrolyzed *N*-imino glycine side chain [c]Values in parentheses represents the crude purity of the pentamer with only a single *N*-imino glycine side chain reduced to the *N*-aminoalkyl glycine. [d]Hydrazone solution made using 1.0 M KI in DMF instead of DMF.[e]Heated in a sonicator bath [f]Cleavage VI was not performed on this sample

8. Stability studies of tripeptoids 13a-d and S13a-d

A small amount of tripeptoids **13a-d** or **S13a-d** were cleaved with Cleavage III (TFA:H_2O 95:5 v/v, 10 mins) for each stability experiment run. The cleavage solution was collected by filtering the resin through the disposable fritted cartridge into a 20 mL scintillation glass vial. The resin in the cartridge was washed with CH_2Cl_2 (3 x 500µL) into the same vial. The filtrate was evaporated to dryness using a Biotage® V10 evaporator, then the peptoid redissolved in fresh CH_2Cl_2 and evaporated to dryness again on the Biotage® V10 evaporator. To ensure removal of all residual TFA, samples were dissolved in 1 mL 1:1 MeCN:H_2O, frozen, and put on the lyophilizer overnight.* To make the samples for the stability studies containing 0.1% acid, tripeptoid samples were dissolved in either 1 mL 1:1 0.1% TFA or formic acid in MeCN: 0.1% TFA or formic acid in H_2O. To make the samples for the stability studies at neutral pH tripeptoids were dissolved in 1 mL of phosphate buffer (0.1 M, pH 7), tripeptoid samples were dissolved in 1 mL pH 7 phosphate buffer and 150 µL MeCN to increase peptoid solubility in the buffer. LCMS data was taken for each sample approximately every 24 hours for about ~100 hours or every 2 hours for 8 hours. Percent hydrolysis conversions were calculated using areas obtained from peaks on LCMS traces at 214 nm, where percent hydrolysis = [(area of **13**)/(area of **13** +**14**)] x 100 or [(area of **S13**)/(area of **S13** +**S14**)] x 100.

*<u>*Note:</u> hydrazone exchange may be observed in samples containing multiple different N-imino glycines if there is residual TFA when the crude peptoids are redissolved in water. Redissolving the crude peptoid in phosphate buffer pH 7 can help prevent this exchange.*

Further observations can be found below:

a. Unacetylated samples **S13c and d** can hydrolyze more quickly in pH 7.

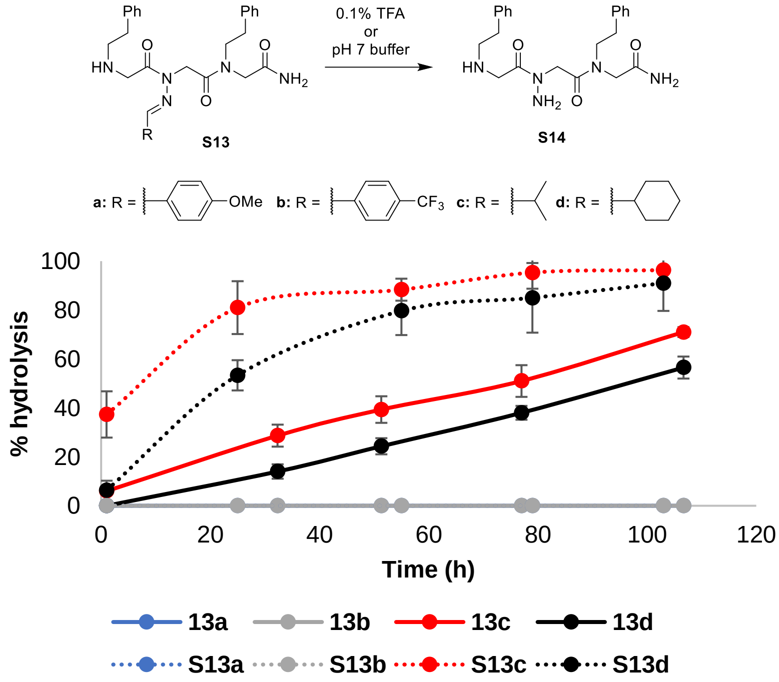

Figure S8. % Hydrolysis of samples **S13a-d** in pH 7 buffer (dashed lines) compared to **13a-d** in pH 7 buffer. The blue curves overlay with the gray curves and are not visible.

b. Acetylated peptoids **13a-d** hydrolyze faster in 0.1% TFA *vs* 0.1% FA 1: 1 H_2O: MeCN. Moreover, 0.1% TFA can lead to the appearance of a side-product with the same mass as **14**, presumed to be **S15** (shown below).

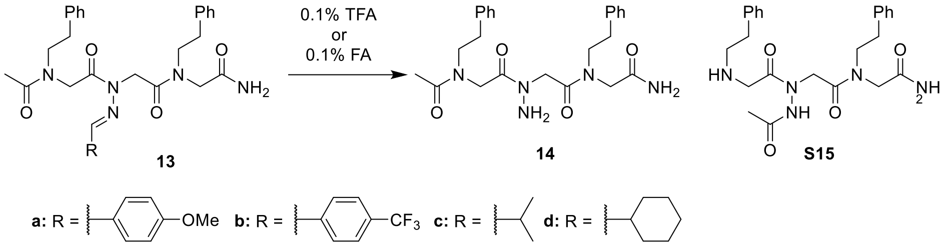

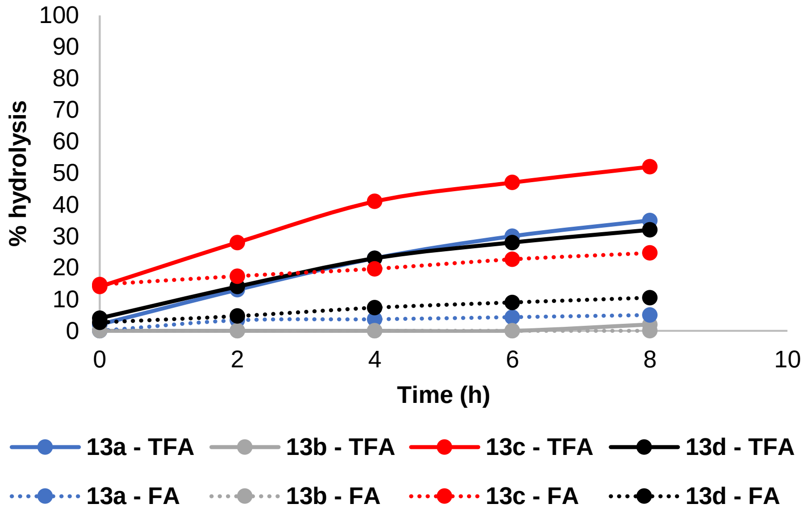

Figure S9. % hydrolysis of acetylated samples **13a-d** in 0.1% TFA 1:1 MeCN: H_2O compared to 0.1% FA 1: 1 MeCN: H_2O (dashed lines). Total hydrolysis for TFA samples is the sum of **14** + **S15** integrations divided by the total of **13** + **14** + **S15**.

Examples of LCMS traces showing the appearance of a second peak (**S15**) with 0.1% TFA can be found on the next page.

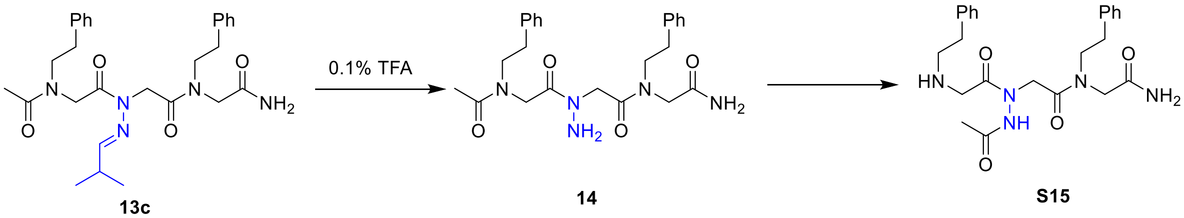

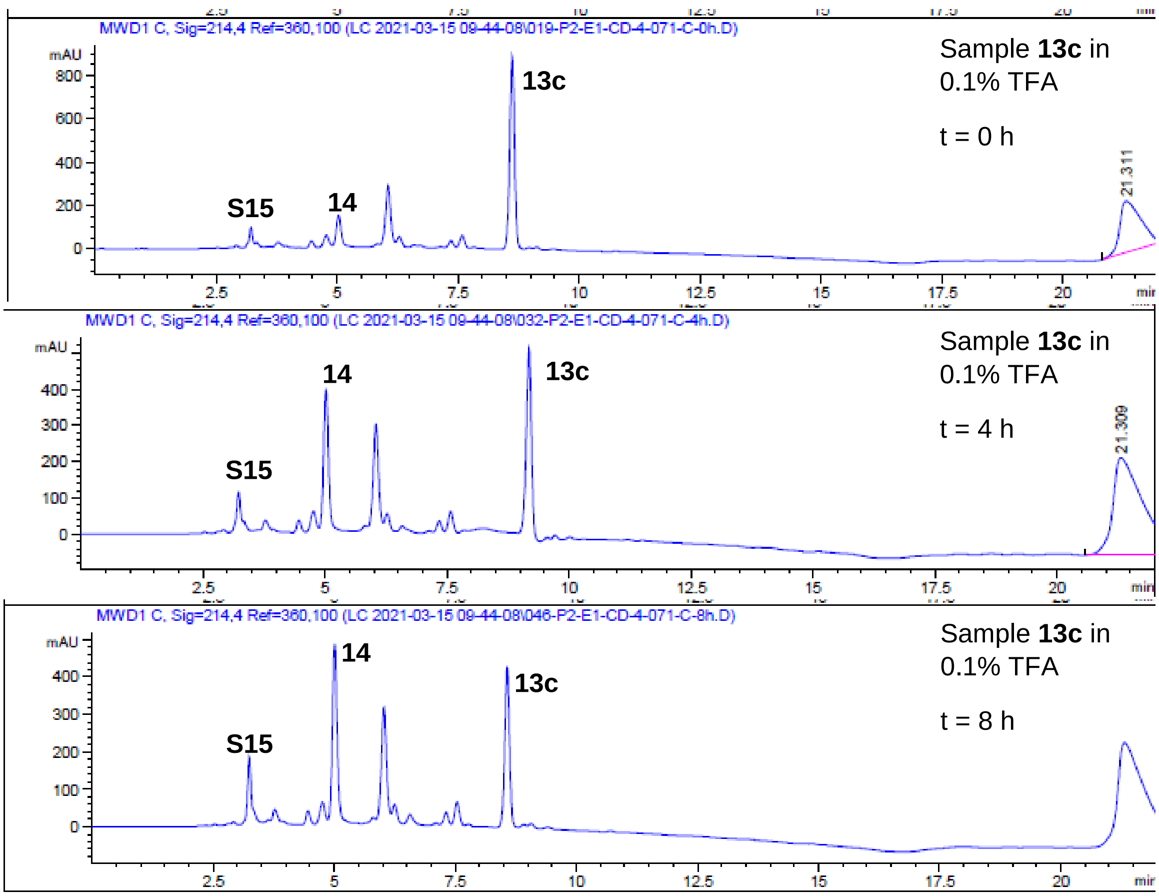

Figure S10. LCMS chromatogram at 214 nm showing decomposition of **13c** over 8 hours.

A side-product with the same mass as **14** begins to appear at 4 h (**S15**) and increases at 8 h as the peak corresponding to **14** depletes. In longer studies (data not shown), 100% conversion of **14** to **S15** was detected after 4 days.

When the acid used is changed from TFA to FA, this second hydrolysis/acetyl transfer peak does not appear within 8 hours.

c. In the presence of 1 mM Lys or Cys, no increase in decomposition was detected for any analogs tested compared to decomposition in pH 7 buffer alone. To make these samples, 100 µL of 2 mM amino acid solution in pH 7 buffer was mixed with 100 µL of 2 mM peptoid trimer. Minimal MeCN was used to adjust solubility in the 2 mM solutions.

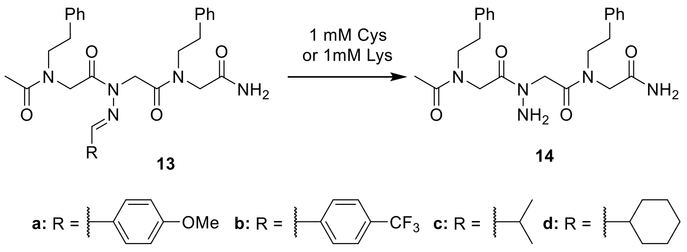

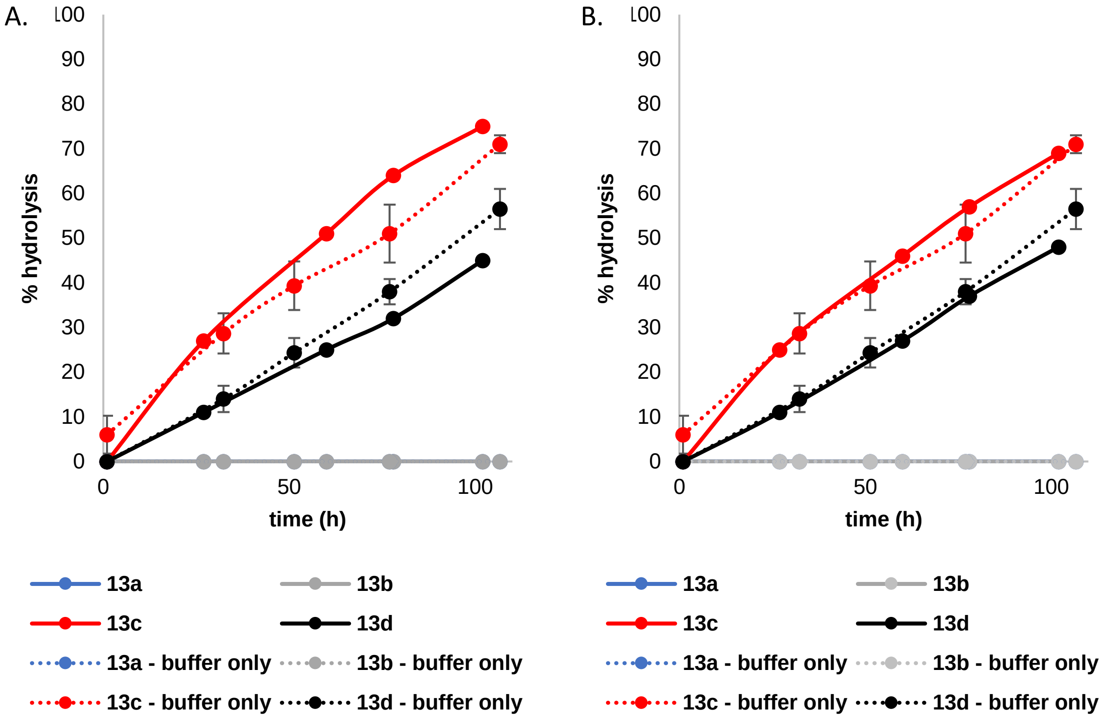

Figure S11. % hydrolysis of samples **13a-d** in pH 7 buffer in the presence of A) cysteine or B) lysine. In both plots, the dashed lines represent the data in the main text corresponding to hydrolysis in pH 7 buffer without the presence of either amino acid. The blue curves overlay with the gray curves and are not visible.

9. Hydrazone exchange using hexapeptoid model compounds.

In order to investigate the hydrazone exchange phenomenon, four different hexapeptoids were synthesized using automated synthesis with different pairs of hydrazones. With only two hydrazones included in the oligomer, only three exchange products are possible, making them more straightforward to analyze. Using Cleavage III on these residues led to the most exchange occurring. In contrast, 95: 5 TFA: phenol (cleavage X) or short cleavages with 45: 50: 5 TFA:CH_2Cl_2: TIPS (cleavage VIII) provided the desired *N*-imino glycine-containing product as the major product.

Rules learned with these model 6mers were applied to the compound **15** included in the main text to obtain *N*-imino glycine-containing peptoids without exchange, hydrolysis or reduction.

Table S4. Ratios of exchange products in acetylated hexapeptoids **S16-18** with two hydrazone side chains.

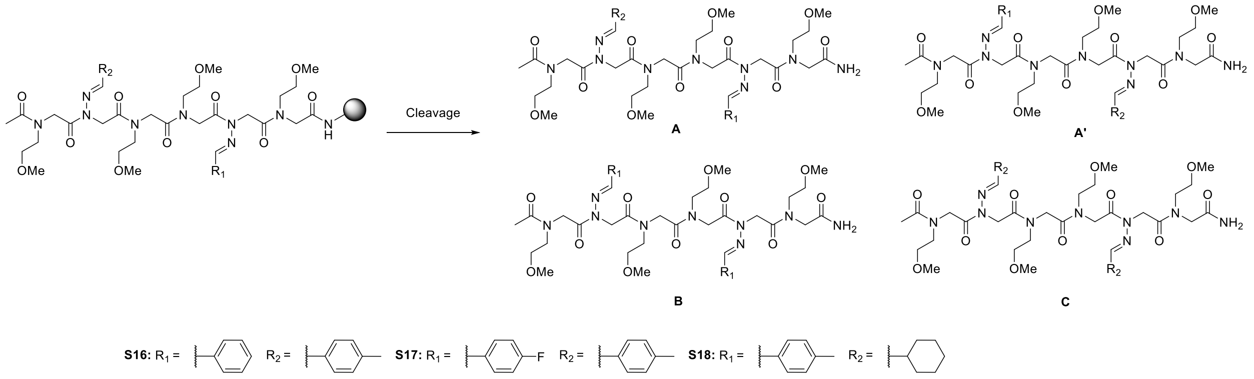

Compound	Cleavage	Ratio of products A/A':B:C
**S16**	III	51:36:13
	VIII	84:9:8
	X	79:16:5
**S17**	III	52:19:29
	VIII	100:0:0
	X	81:0:19
**S18**	III	41:50:9
	VIII	100[a]:0:0
	X	58:35:8

[a]A mass of M+2 is detected in the peak as well, indicating partial reduction, likely of the aliphatic side chain.

Representative LCMS traces for hexapeptoid **S16** cleaved with the three different cleavage cocktails can be found on the next pages.

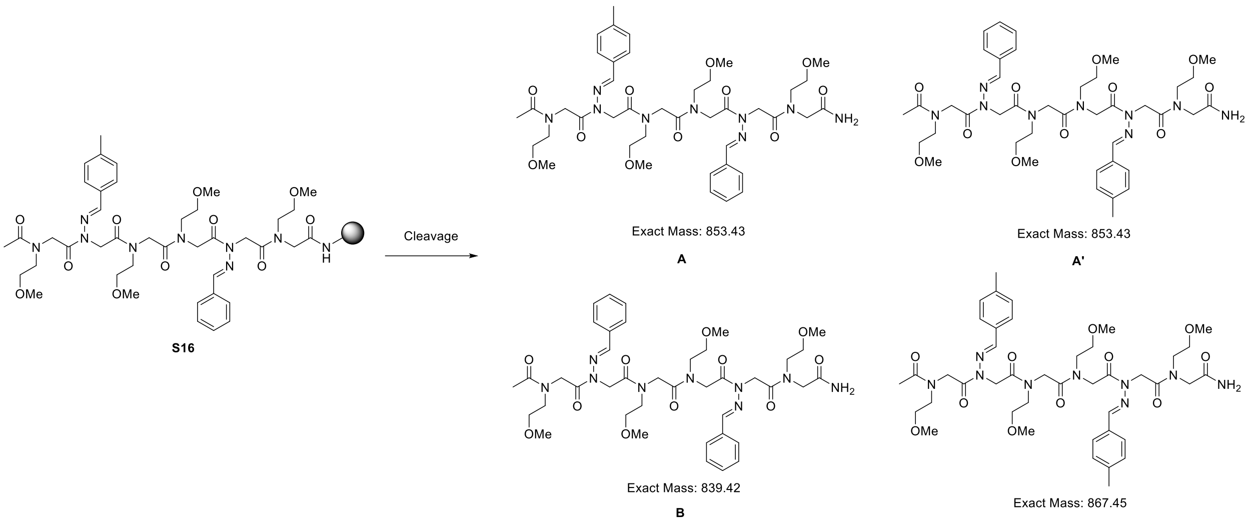
S16
Cleavage
Exact Mass: 853.43
A
Exact Mass: 853.43
A'
Exact Mass: 839.42
B
Exact Mass: 867.45
C

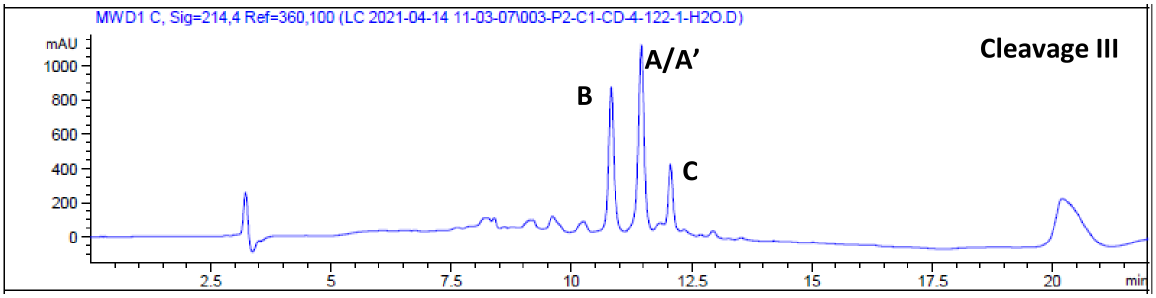
MWD1 C, Sig=214,4 Ref=360,100 (LC 2021-04-14 11-03-07\003-P2-C1-CD-4-122-1-H2O.D)
mAU
Cleavage III
A/A'
B
C
min

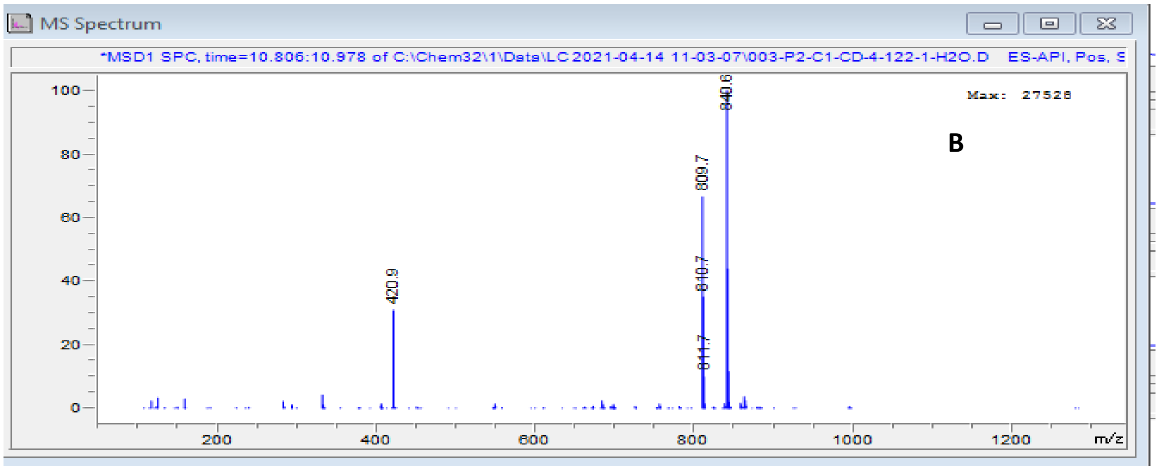
MS Spectrum
*MSD1 SPC, time=10.806:10.978 of C:\Chem32\1\Data\LC 2021-04-14 11-03-07\003-P2-C1-CD-4-122-1-H2O.D ES-API, Pos,
Max: 27528
B
840.6
809.7
810.7
420.9
m/z

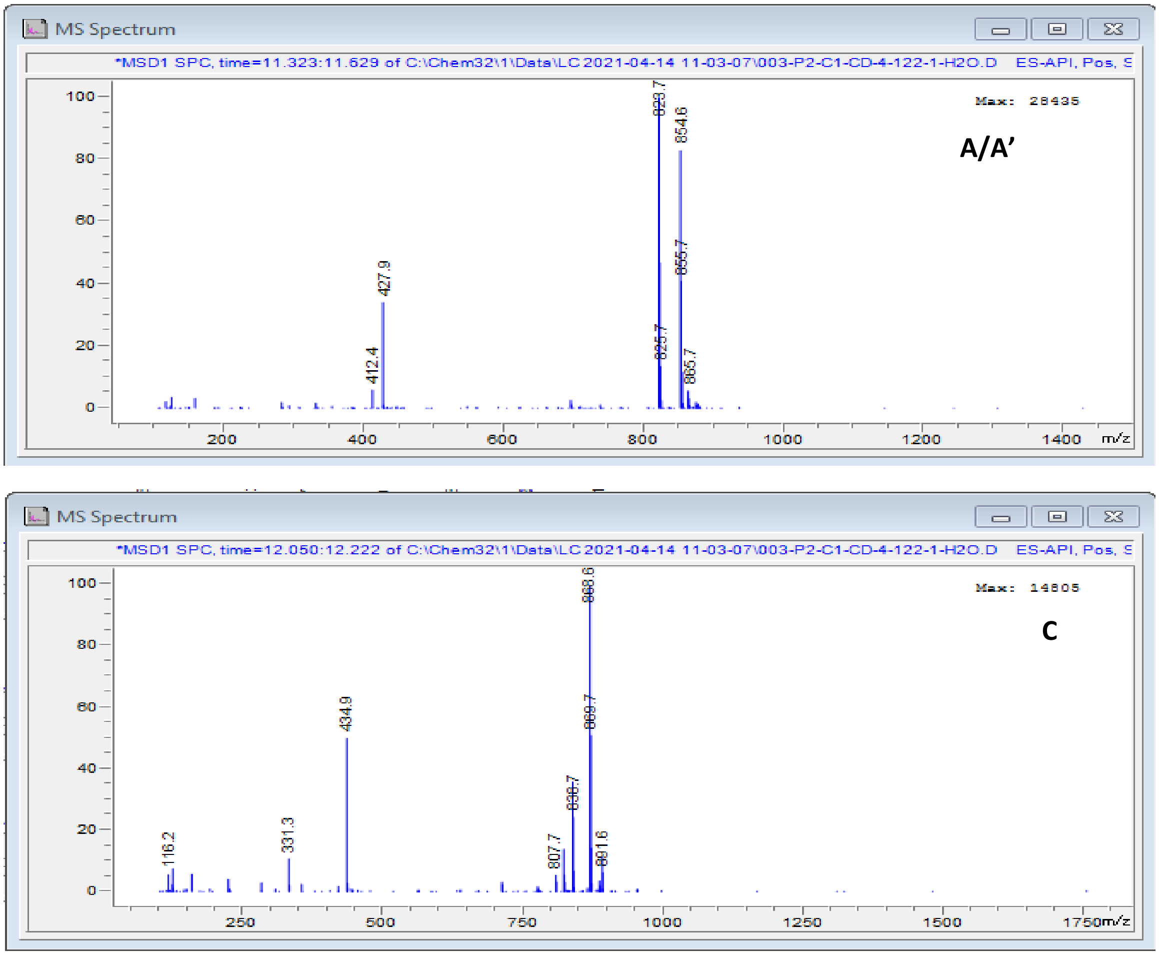

Figure S12. Representative LCMS chromatograms at 214 nm of peptoid **S16** when using cleavage III.

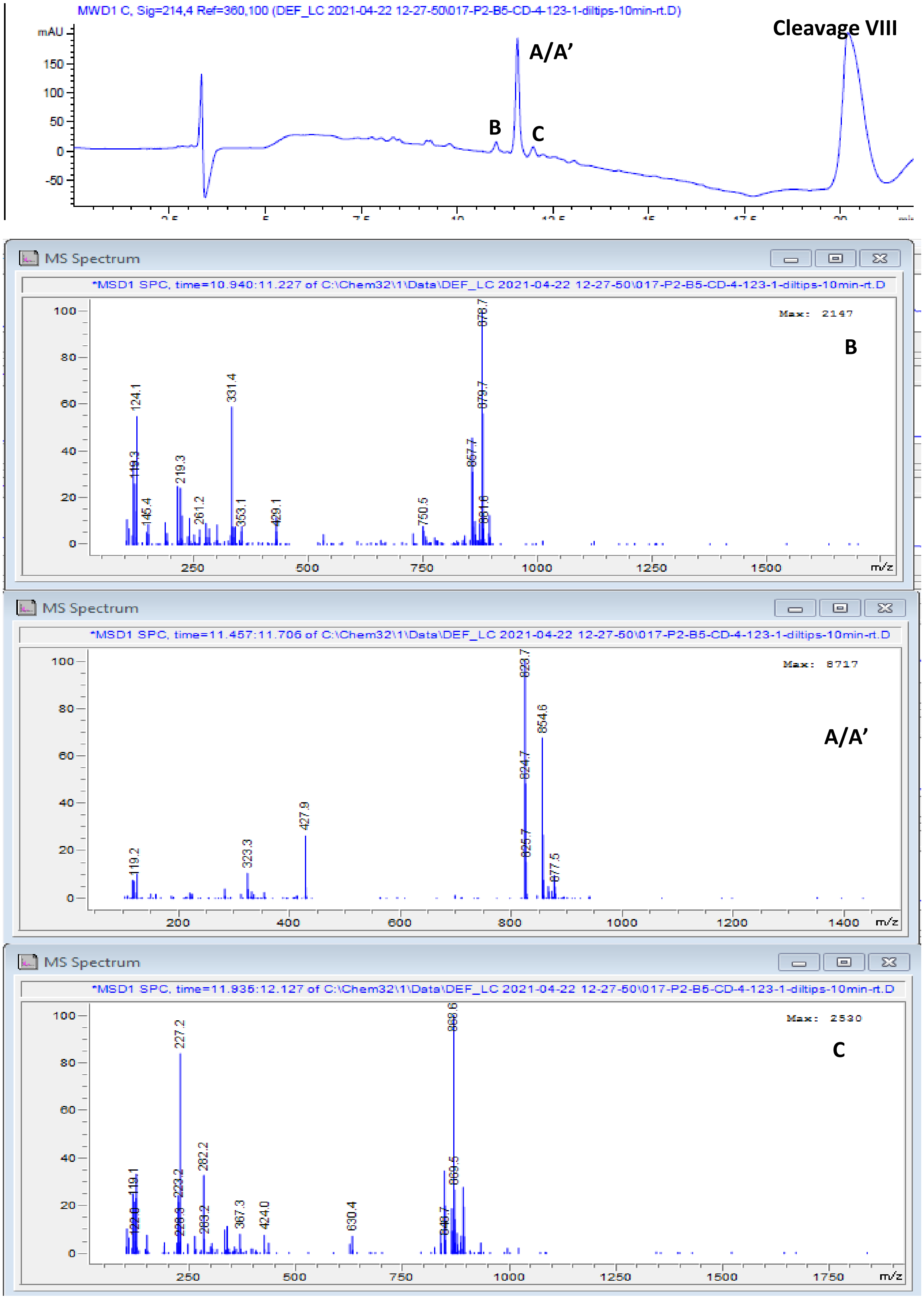

Figure S13. Representative LCMS chromatograms at 214 nm of peptoid **S16** when using cleavage VIII.

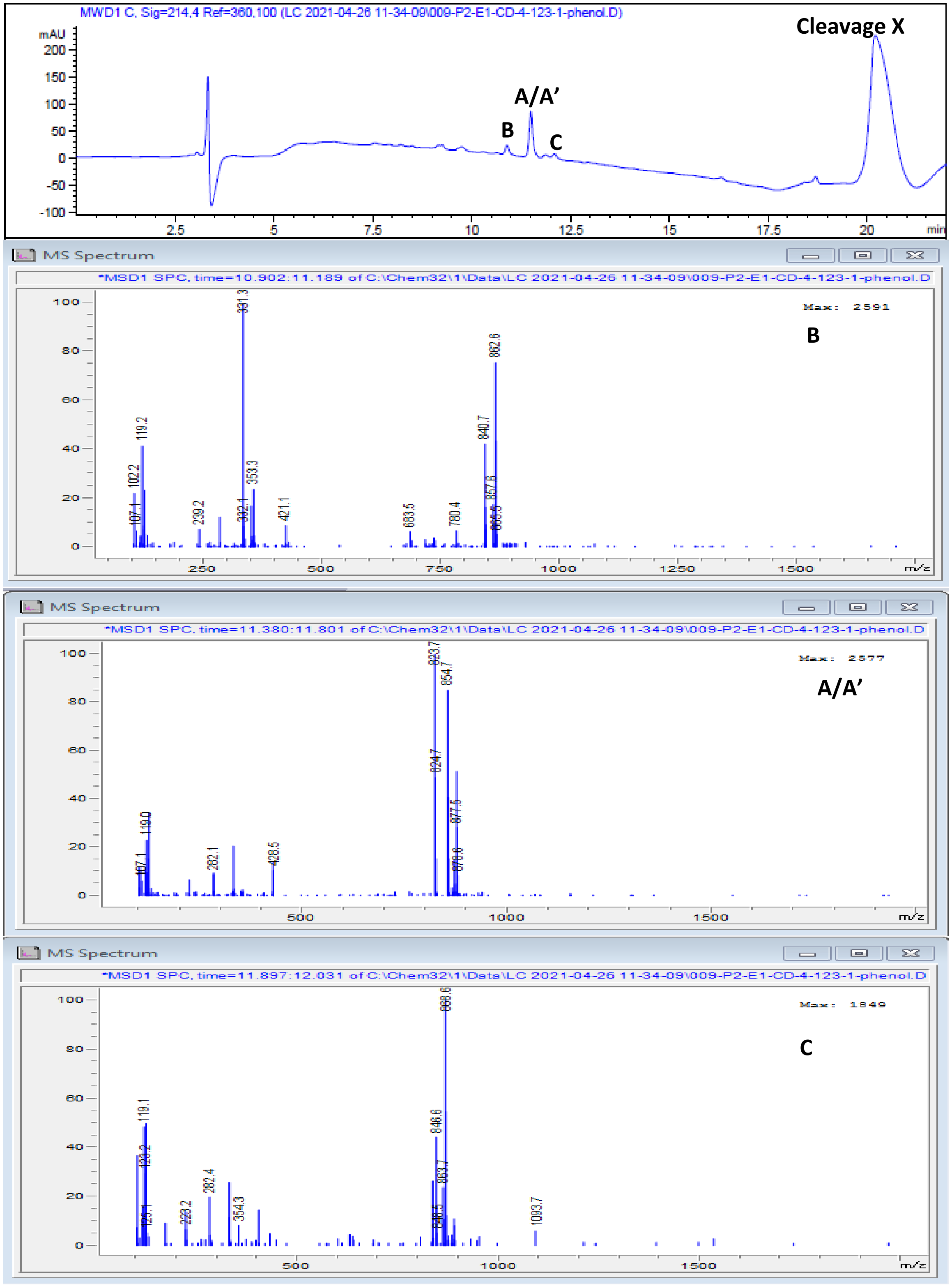

Figure S14. Representative LCMS chromatograms at 214 nm of peptoid **S16** when using cleavage X.

Stability of these acetylated 6mers was then investigated over time using peptoids **S16** and **S18** as representative examples, using the same procedure described in section 8 for the stability studies of tripeptoids **13a-d** and **S13a-d**. The samples were cleaved using Cleavage VIII and the crude peptoids were analyzed *without lyophilization prior* to analysis.

There was no evidence of hydrazone exchange in both pH 7 buffer and 0.1% formic acid 1:1 MeCN:H_2O over the course of the study (8-15 hours).

0.1% FA, 0 h

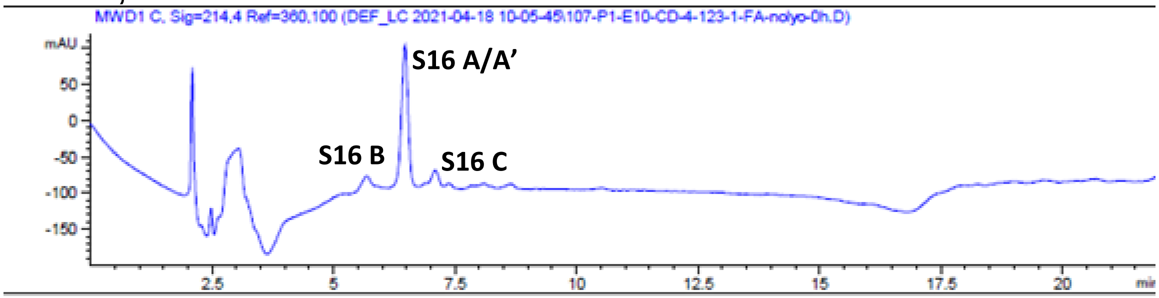

0.1% FA, 8 h

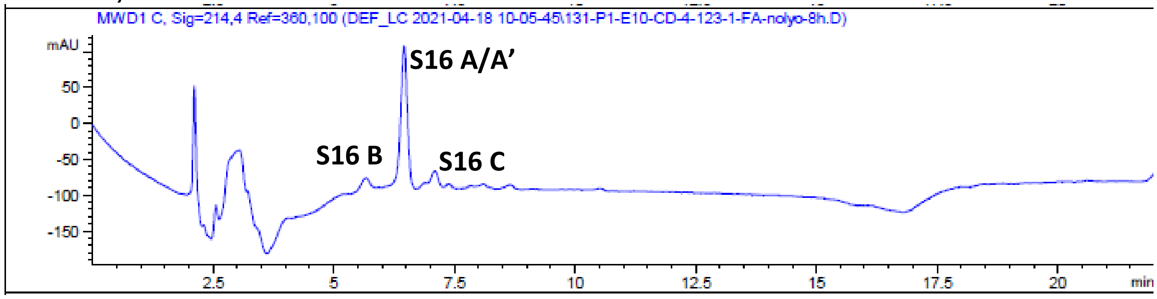

pH 7, 0 h

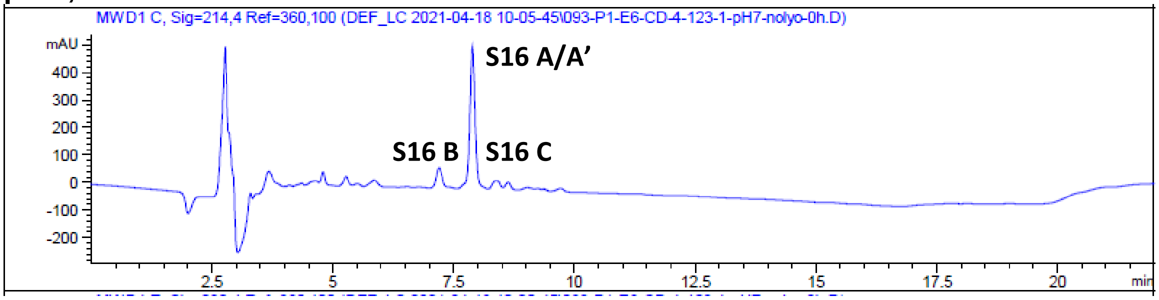

pH 7, 15 h

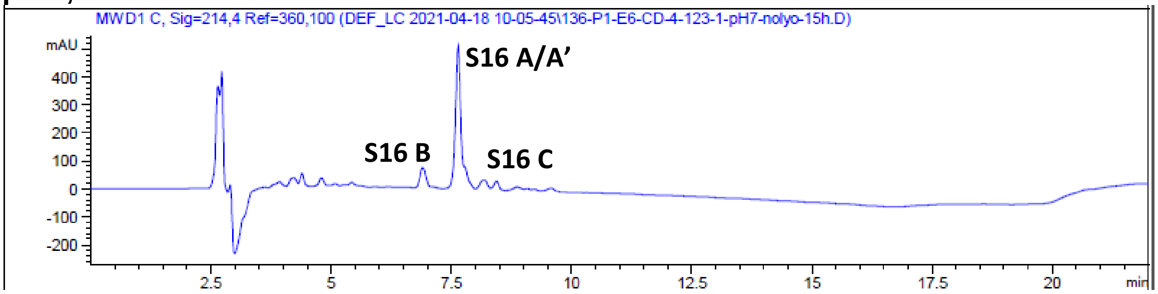

Figure S15. Representative LCMS chromatograms at 214 nm showing stability of peptoid **S16** to pH 7 and 0.1% FA conditions.

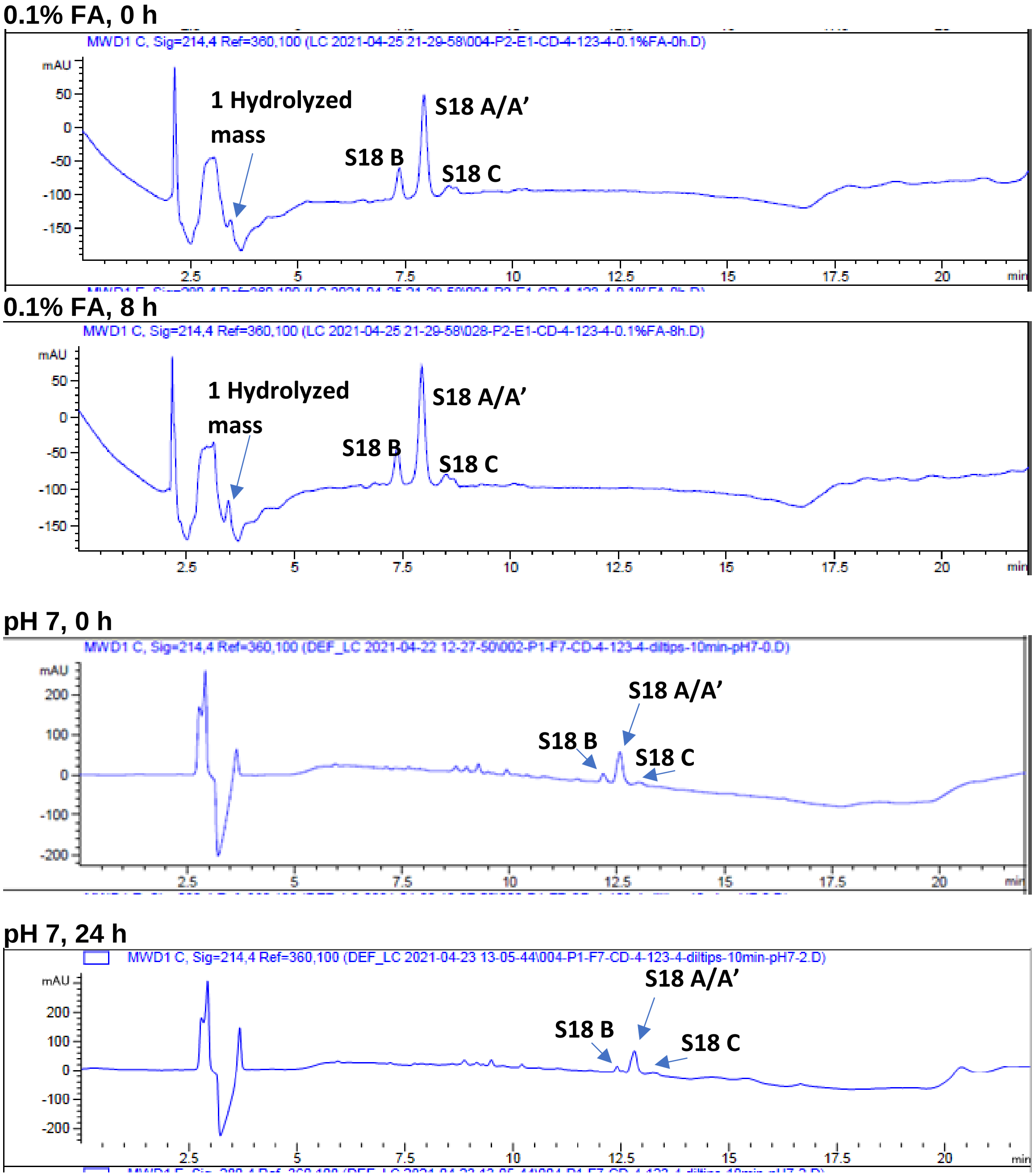

Figure S16. Representative LCMS chromatograms at 214 nm showing stability of peptoid **S18** to pH 7 after 24 h and 0.1% FA conditions after 8 h.

10. Peptoid Characterization Data.

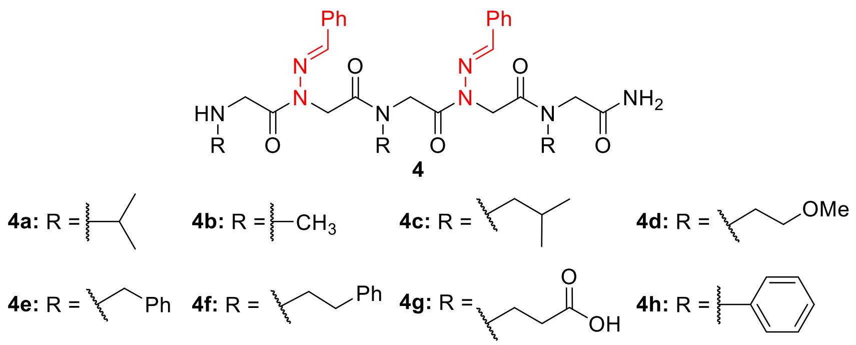

Table S5. Characterization data for peptoids **4a-4h**.

Peptoid	Calculated *m/z* [M + H]$^+$	Observed *m/z* [M + H]$^+$	RT (min)
**4a**	635.3664	635.3642	6.17[a]
**4b**	551.2725	551.2715	9.57[b]
**4c**	677.4133	677.4117	5.91[c]
**4d**	683.3511	683.3495	10.57[b]
**4e**	779.3664	779.3669	9.47[a]
**4f**	821.4133	821.4115	7.60[c]
**4g**	725.2889	725.2886	9.16[b]
**4h**	737.3194	737.3186	12.00[d]

[a]LC/MS analyses were performed using a 12 min 40-80% linear gradient of MeCN (0.1% TFA) in water (0.1% TFA) on Column 1.
[b]LC/MS analyses were performed using a 12 min 5-95% linear gradient of MeCN (0.1% TFA) in water (0.1% TFA) on Column 1.
[c]LC/MS analyses were performed using a 12 min 50-95% linear gradient of MeCN (0.1% TFA) in water (0.1% TFA) on Column 1.
[d]LC/MS analyses were performed using a 12 min 50-95% linear gradient of MeCN (0.1% TFA) in water (0.1% TFA) on Column 2.

Table S6. Characterization data for peptoids **8-9** and **S20-S23**.

	Structure	Synthesis type	Cleavage Conditions	Crude purity[a]	Calcd *m/z* $[M + H]^+$	Obsd *m/z* $[M + H]^+$	RT (min)
**8**	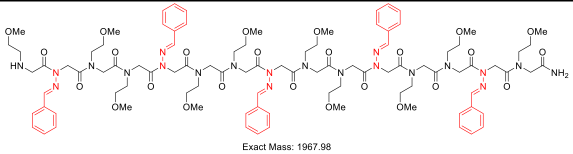	Automated	III	63	984.9964[b]	984.9944[b]	9.62
**9**	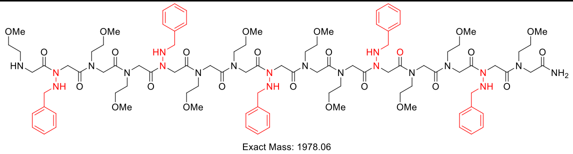	Automated	VI[c]	69	990.0355[d]	990.0329[d]	7.94
**S19**	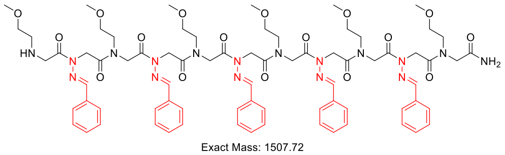	Manual	III	47	1508.8104	1508.7357	9.45
		Automated	III	55	1508.8104	1508.7357	9.44
**S20**	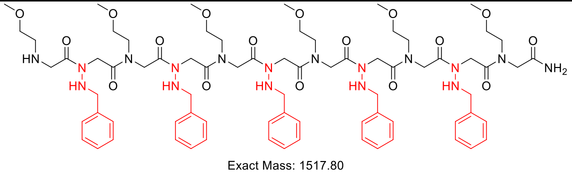	Automated	VI[c]	40	1518.8104	1518.8117	8.16
**S21**	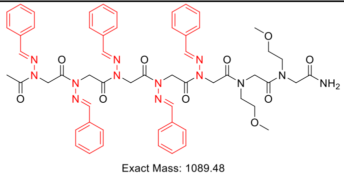	Manual	III	47	1090.4894	1090.4870	10.71
**S22**	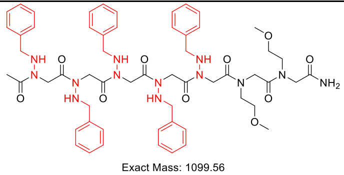	Manual	VI	26	1110.5676	1100.5649	8.85

[a]LC/MS analyses were performed using a 12 min 50-95% linear gradient of MeCN (0.1% TFA) in water (0.1% TFA) on Column 2. [b]LC/MS analyses were performed using a 12 min 50-95% linear gradient of MeCN (0.1% TFA) in water (0.1% TFA) on Column 1. [b]$[M + 2H]^{2+}$ instead of $[M + H]^+$ [c]Crude peptoid was treated with a fresh solution of the Cleavage VI cocktail for an additional 2 h.

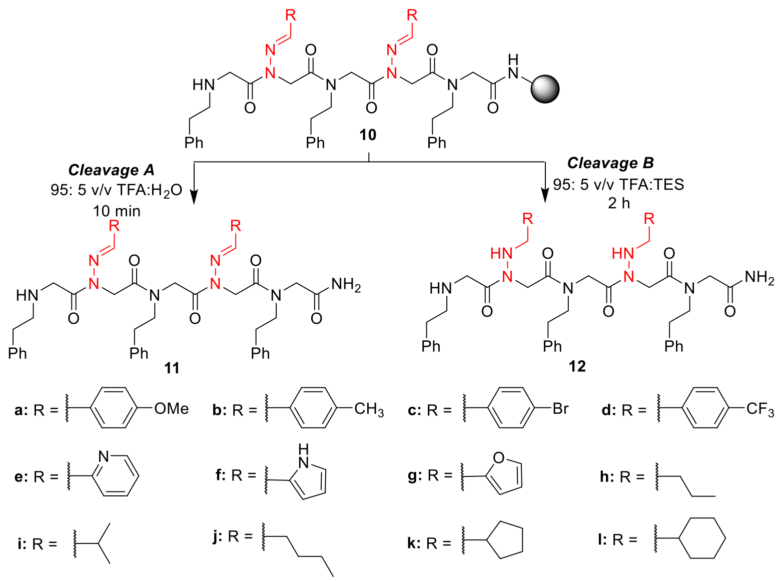

Table S7. Characterization data for peptoids **11a-l** and **12 a-l**.

Peptoid	Calculated *m/z* [M + H]$^+$	Observed *m/z* [M + H]$^+$	RT (min)[a]	Peptoid	Calculated *m/z* [M + H]$^+$	Observed *m/z* [M + H]$^+$	RT (min)[a]
**11a**	881.4345	881.4328	9.07	**12a**	885.4658	885.4632	8.64
**11b**	849.4446	849.4424	10.33	**12b**	853.4759	853.4734	10.17
**11c**	977.2344	977.2319	10.59	**12c**	981.2657	981.2613	10.34
**11d**	957.3881	957.3883	11.00	**12d**	961.4194	961.4172	10.56
**11e**[b]	823.4038	823.4027	8.58	N/A	N/A	N/A	N/A
**11f**	799.4038	799.4029	7.71	N/A	N/A	N/A	N/A
**11g**	801.3719	801.3696	7.57	**12g**	805.4032	805.4013	7.47
**11h**	753.4446	753.4429	8.54	**12h**	757.4759	757.474	9.02
**11i**	753.4446	753.4428	8.57	**12i**	757.4759	757.4744	8.96
**11j**	781.4759	781.4753	9.93	**12j**	785.5072	785.5067	10.35
**11k**	805.4759	805.4751	10.15	**12k**	809.5072	809.5066	10.66
**11l**	833.5072	833.5067	11.14	**12l**	837.5385	837.5375	11.61

[a]LC/MS analyses were performed using a 12 min 50-95% linear gradient of MeCN (0.1% TFA) in water (0.1% TFA) on Column 2 unless otherwise indicated. [b]LC/MS analyses were performed using a 12 min 30-90% linear gradient of MeCN (0.1% TFA) in water (0.1% TFA) on Column 2.

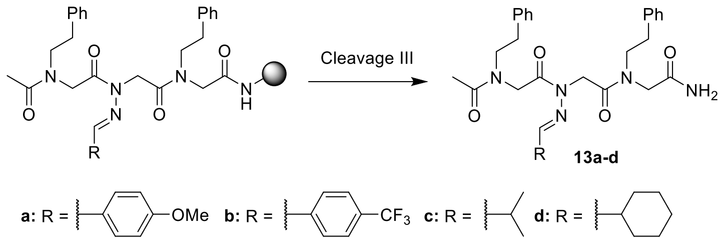

Table S8. Characterization data for peptoids **13a-13d** used in stability studies.

Peptoid	Calculated *m/z* [M + H]$^+$	Observed *m/z* [M + H]$^+$	RT (min)[a]
**13a**	572.2868	572.2866	9.27
**13b**	610.2636	610.2633	11.07
**13c**	508.2918	508.2922	8.91
**13d**	548.3231	548.3230	10.93

[a]LC/MS analyses were performed using a 12 min 30-90% linear gradient of MeCN (0.1% TFA) in water (0.1% TFA) on Column 2

Table S9. Characterization data for peptoid 6mers (with two different hydrazones)

	Structure	Synthesis type	Cleavage Conditions	Crude purity	Calcd *m/z* $[M + H]^{2+}$	Obsd *m/z* $[M + H]^{2+}$	RT (min)
**S16**	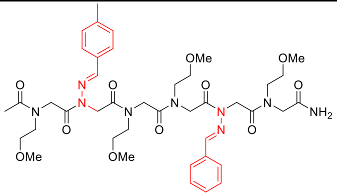 Chemical Formula: $C_{41}H_{59}N_9O_{11}$ Exact Mass: 853.43	Automated	VIII	75[a]	854.4407	854.4396	11.58
**S17**	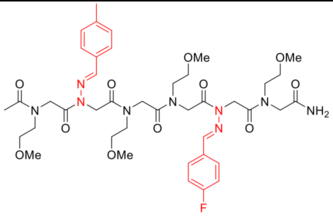 Chemical Formula: $C_{41}H_{58}FN_9O_{11}$ Exact Mass: 871.42	Automated	VIII	84[a]	872.4313	872.4300	11.81
**S18**	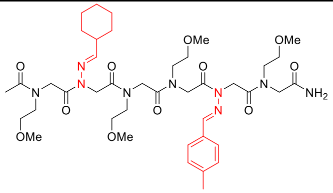 Chemical Formula: $C_{41}H_{65}N_9O_{11}$ Exact Mass: 859.48	Automated	VIII	79[b]	860.4876	860.4856	9.08

[a]LC/MS analyses were performed using a 12 min 30-90% linear gradient of MeCN (0.1% TFA) in water (0.1% TFA) on Column 2.
[b]LC/MS analyses were performed using a 12 min 50-95% linear gradient of MeCN (0.1% TFA) in water (0.1% TFA) on Column 2.

Table S10. Characterization data for peptoids 15 and 16

	Structure	Synthesis type	Cleavage Conditions	Crude purity[a]	Calcd *m/z* $[M + 2H]^{2+}$	Obsd *m/z* $[M + 2H]^{2+}$	RT (min)
**15**	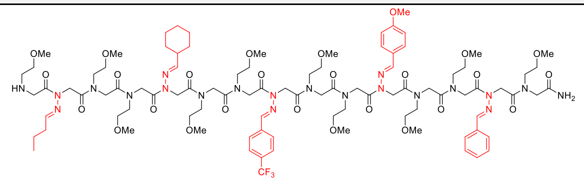 Exact Mass: 2038.04	Automated	VIII	52	1020.0266	1020.0275	10.87
**16**	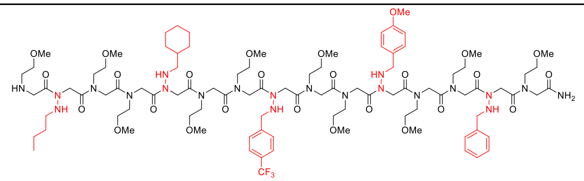 Exact Mass: 2048.12	Automated	VI[b]	69	1025.0658	1025.0685	10.00

[a]LC/MS analyses were performed using a 12 min 50-95% linear gradient of MeCN (0.1% TFA) in water (0.1% TFA) on Column 2. [b]Crude peptoid was treated with a fresh solution of the Cleavage VI cocktail for an additional 2 h**.**

11. Representative protocols for synthesis of model peptoid in solution

2-bromo-1-(piperidin-1-yl)ethan-1-one (18)

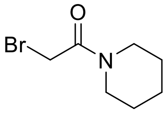

The synthesis was performed following literature procedure with minor modifications.[6] A solution of piperidine (5 mmol, 0.5 mL) and TEA (8 mmol, 1.1 mL) in dry CH_2Cl_2 (13 mL) was cooled to −78 °C in an oven-dried round bottom flask under an argon atmosphere. Bromoacetyl bromide (7.5 mmol, 0.65 mL) was added dropwise to the stirring solution. After addition, the solution was reacted at room temperature for 15 minutes. After the reaction was complete, the solution was transferred to a separatory funnel with 30 mL CH_2Cl_2 and washed with cold saturated aq. $NaHCO_3$ (2x), 10% w/v citric acid solution (2x), and saturated brine (1x). The organic layer was collected, dried with Na_2SO_4, and the solvent was removed under reduced pressure. The material was carried on to the next step without further purification.

(*E*)-2-(2-benzylidenehydrazineyl)-1-(piperidin-1-yl)ethan-1-one (19a)

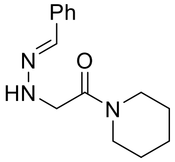

The crude 2-bromo-1-(piperidin-1-yl)ethan-1-one, **18** (5.0 mmol) and TEA (1.4 mL, 10.0 mmol) were dissolved in dry THF (20 mL) in an oven-dried round bottom flask under an argon atmosphere and cooled to 0 °C in an ice bath. Benzaldehyde hydrazone (2.40 g, 20.0 mmol) was dissolved in dry THF (5 mL) in a 20 mL vial under argon atmosphere and cooled to 0 °C before being added dropwise to the solution of **18**. After the addition, the reaction was removed from the ice bath and left under an argon balloon for 2 hours at room temperature. The reaction mixture was filtered and the THF was removed under reduced pressure. The product was purified by flash chromatography with a Biotage® Isolera using a 40-100% ethyl acetate in hexanes gradient to give **19a** as an orange oil (0.7894 g, 3.2 mmol, 64% yield over two steps): **Rf** = 0.42 (40% ethyl acetate in hexanes); ^{1}**H NMR** (600 MHz, $CDCl_3$) δ 7.70 (s, 1H), 7.53 (d, *J* = 7.69 Hz, 2H), 7.31 (t, *J* = 7.41 Hz, 2H), 7.24 (t, *J* = 7.35 Hz, 1H), 6.30 (broad s, 1H), 4.05 (s, 2H), 3.55 (t, *J* = 5.66 Hz, 2H), 3.37 (t, *J* = 5.50 Hz, 2H), 1.65-1.53 (m, 6H); ^{13}**C NMR** (150 MHz, $CDCl_3$) δ 167.50, 138.99, 135.54, 128.21, 127.87, 125.80, 49.63, 45.23, 42.70, 26.03, 25.20, 24.17; **HRMS** (HESI/orbitrap) calculated for [$C_{14}H_{19}N_3O$] calculated *m/z* $[M + H]^+$ = 246.1601, observed *m/z* $[M + H]^+$ = 246.1605.

(*E*)-2-(2-(4-methoxybenzylidene)hydrazineyl)-1-(piperidin-1-yl)ethan-1-one (19b)

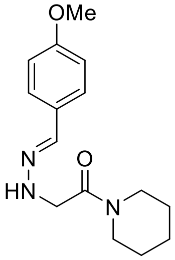

19b was synthesized according to the procedure described above for the synthesis of **19a** from **18** (3.4 mmol) and 4-methoxybenzaldehyde hydrazone (1.02 g, 6.8 mmol). The product was purified by flash chromatography with a Biotage® Isolera using a 20-100% ethyl acetate in hexanes gradient to give **19b** as a dark yellow oil (0.3194 g, 1.2 mmol, 23% yield over two steps). *Due to its instability, the product was used immediately after purification in the next step.*

(*E*)-1-(piperidin-1-yl)-2-(2-(4-(trifluoromethyl)benzylidene)hydrazineyl)ethan-1-one (19c)

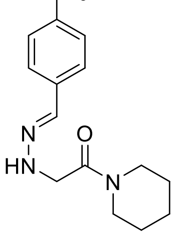

19c was synthesized according to the procedure described above for the synthesis of **19a** from **18** (2.2 mmol) and 4-(trifluoromethyl)benzaldehyde (800 mg, 4.3 mmol). The product was purified by flash chromatography with a Biotage® Isolera using a 20-100% ethyl acetate in hexanes gradient to give **19c** as an orange oil (0.3523 g, 1.1 mmol, 52% yield over two steps). *Due to its instability, the product was used immediately after purification in the next step.*

(*E*)-*N'*-benzylidene-*N*-(2-oxo-2-(piperidin-1-yl)ethyl)acetohydrazide (20a)

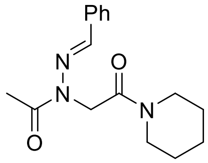

Compound **19a** (500 mg, 2.04 mmol) was dissolved in dry CH_2Cl_2 (21 mL) in an oven-dried 100 mL round bottom flask under an argon atmosphere and cooled to 0 °C in an ice bath. TEA (0.70 mL, 5.10 mmol) was added followed by the dropwise addition of acetyl chloride (0.40 mL, 5.10 mmol). The reaction (yellow solution) was removed from the ice bath and stirred under an argon atmosphere for 30 minutes at room temperature. The solvent was removed under reduced pressure and the product was purified by flash chromatography with a Biotage® Isolera using a 40-100% ethyl acetate in hexanes gradient to give **20a** as a light yellow solid (0.5044 g, 1.76 mmol, 86% yield): **Rf** = 0.24 (40% ethyl acetate in hexanes); ^{1}**H NMR** (600 MHz, $CDCl_3$) δ 7.67 (d, *J* = 6.38 Hz, 2H), 7.64 (s, 1H), 7.39-7.33 (m, 3 H), 4.82 (s, 2H), 3.54 (t, *J* = 5.67 Hz, 2 H), 3.38 (t, *J* = 5.34 Hz, 2 H), 2.52 (s, 3H), 1.67-1.54 (m, 6H); ^{13}**C NMR** (150 MHz, $CDCl_3$) δ 172.62, 163.68, 139.30,134.41, 129.38, 128.33, 126.90, 45.95, 43.07, 42.52, 26.18, 25.26, 24.12, 21.38 **IR** (neat) 2939, 2859, 1670, 1636, 1411, 1233, 945, 756, 695, 554. **HRMS** (HESI/orbitrap) calculated for [$C_{16}H_{21}N_3O_2$] calculated *m/z* $[M + H]^+$ = 288.1707, observed *m/z* $[M + H]^+$ = 288.1710. **MP** = 143.4 °C - 146.5 °C.

(*E*)-*N*'-(4-methoxybenzylidene)-N-(2-oxo-2-(piperidin-1-yl)ethyl)acetohydrazide (20b)

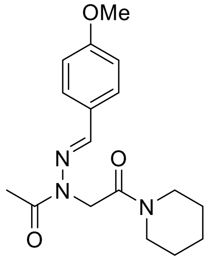

20b was synthesized according to the procedure described above for the synthesis of **20a** from **19b** (311 mg, 1.1 mmol). The product was purified by flash chromatography with a Biotage® Isolera using a 30-100% ethyl acetate in hexanes gradient to give **20b** as a light orange solid (0.2995 g, 0.94 mmol, 84% yield); **Rf** = 0.18 (40% ethyl acetate in hexanes); ^{1}**H NMR** (600 MHz, $CDCl_3$) δ 7.60 (s, 2H), 7.58 (s, 1H), 6.88 (d, *J* = 8.85 Hz, 2 H), 4.79 (s, 2H), 3.81 (s, 3H), 3.53 (t, *J* = 5.77 Hz, 2 H), 3.47 (t, *J* = 4.76 Hz, 2 H), 2.49 (s, 3H), 1.65-1.53 (m, 6H) ^{13}**C NMR** (125 MHz, $CDCl_3$) δ 172.87, 164.38, 160.90, 139.75, 128.67, 127.48. 114.05. 55.35, 46.36, 43.47, 43.12, 26.47, 25.56, 24.43, 21.66; **IR** (neat) 2934, 1645, 1512, 1409, 1243, 1165, 1025, 829, 530. **HRMS** (HESI/orbitrap) calculated for [$C_{17}H_{23}N_3O_3$] calculated *m/z* $[M + H]^+$ = 318.1812, observed *m/z* $[M + H]^+$ = 318.1810. **MP** = 121.7 °C -122.8 °C.

(*E*)-*N*-(2-oxo-2-(piperidin-1-yl)ethyl)-N'-(4-(trifluoromethyl)benzylidene)acetohydrazide (20c)

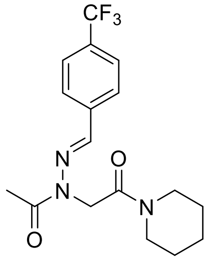

20c was synthesized according to the procedure described above for the synthesis of **20a** from **19c** (350 mg, 1.1 mmol). The product was purified by flash chromatography with Biotage® Isolera using a 30-100% ethyl acetate in hexanes gradient to give **20c** as a light yellow solid (0.3290 g, 0.93 mmol, 83% yield): **Rf** = 0.23 (40% ethyl acetate in hexanes); ^{1}**H NMR** (500 MHz, $CDCl_3$) δ 7.77 (d, *J* = 8.12 Hz, 2H), 7.65 (s, 1H), 7.62 (d, *J* = 8.31 Hz, 2H), 4.83 (s, 2H), 3.55 (t, *J* = 5.55 Hz, 2 H), 3.49 (t, *J* = 5.23 Hz, 2 H), 2.53 (s, 3H), 1.68-1.55 (m, 6H); ^{13}**C NMR** (125 MHz, $CDCl_3$) δ 173.18, 163.95, 138.00, 131.68 (q, *J* = 32.2 Hz), 127.42, 125.74 (q, *J* = 3.8 Hz), 46.51, 43.61, 43.15, 26.62, 25.66, 24.51, 21.76; **IR** (neat) 2938, 1675, 1645, 1410, 1157, 1106, 1064, 979, 951, 928, 841, 599, 563, 435. **HRMS** (HESI/orbitrap) calculated for [$C_{17}H_{20}F_3N_3O_2$] calculated *m/z* $[M + H]^+$ = 356.1580, observed *m/z* $[M + H]^+$ = 356.1578. **MP** = 184.6 °C – 186.9 °C.

***N'*-benzyl-*N*-(2-oxo-2-(piperidin-1-yl)ethyl)acetohydrazide (21a)**

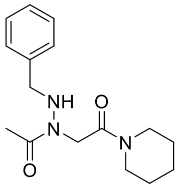

Compound **20a** (100 mg, 0.35 mmol) was dissolved in THF (1.7 mL) in a 10 mL round bottom flask. AcOH (59 µL, 1.05 mmol) was added followed by $NaBH_3CN$ (218.7 mg, 3.48 mmol) and the solution was stirred for 2 h at room temperature. The THF was removed under reduced pressure, and the cloudy oil was redissolved in EtOAc and washed with 1 M $KHSO_4$ (1x) and saturated brine (1x). The organic layer was dried with Na_2SO_4, filtrered, and the solvent was removed under reduced pressure. The solution was redissolved in EtOH and was refluxed for 2 h at 85 °C, followed by removal of EtOH under reduced pressure. The oil was purified by flash chromatography with a Biotage® Isolera with a 4-10% MeOH in CH_2Cl_2 gradient to give **21a** as a white solid (0.0799 g, 0.28 mmol, 79% yield). **Rf** = 0.43 (5% MeOH in CH_2Cl_2), stains with $KMnO_4$; **^{1}H NMR** (600 MHz, $CDCl_3$) *trans* rotamer: *δ* 7.32-7.21 (m, 5H), 4.94 (t, *J* = 5.26 Hz, 1H), 4.39 (s, 2H), 3.88 (d, *J* = 5.24 Hz, 2H), 3.47 (t, *J* = 5.56 Hz, 2H), 3.33 (t, *J* = 5.47 Hz, 2H), 2.07 (s, 3H), 1.66-1.53 (m, 6H). *cis* rotamer: *δ* 7.37-7.25 (m, 5H), 5.40 (t, *J* = 5.12 Hz, 1H), 3.95 (s, 2H), 3.94 (d, *J* = 3.64 Hz, 2H), 3.52 (t, *J* = 5.61 Hz, 2H), 3.16 (t, *J* = 5.46 Hz, 2H), 2.04 (s, 3H), 1.60-1.48 (m, 6H); **^{13}C NMR** (150 MHz, $CDCl_3$) δ 175.22, 166.66, 137.31, 129.05, 128.62, 127.79, 52.35, 46.18, 43.06, 42.22, 26.38, 25.53, 24.47, 21.01; **IR** (neat) 3282, 2936, 2854, 1637, 1229,1026, 756, 737, 549. **HRMS** (HESI/orbitrap) calculated for [$C_{16}H_{23}N_3O_2$] calculated *m/z* $[M + H]^+$ = 290.1863, observed *m/z* $[M + H]^+$ = 290.1869; **MP** = 109.5 °C - 112.5 °C.

***N*'-(4-methoxybenzyl)-N-(2-oxo-2-(piperidin-1-yl)ethyl)acetohydrazide (21b)**

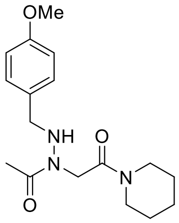

21b was synthesized according to the procedure described above for the synthesis of **21a** from **20b** (150.0 mg, 0.47 mmol). The oil was purified by flash chromatography with a Biotage® Isolera with a 2-8% MeOH in CH_2Cl_2 gradient to give **21b** as a pale yellow solid (0.1099 g, 0.34 mmol, 73% yield). **Rf** = 0.50 (5% MeOH in CH_2Cl_2), product stains with $KMnO_4$; **^{1}H NMR** (500 MHz, $CDCl_3$) *trans* rotamer: 7.22 (d, *J* = 8.85 Hz, 2 H), 6.85 (d, *J* = 8.85 Hz, 2 H), 4.91 (s, broad, 1H), 4.44 (s, 2H), 3.86 (s, 2H), 3.79 (s, 3H), 3.52 (t, *J* = 5.77 Hz, 2H), 3.39 (t, *J* = 4.76 Hz, 2H), 2.11 (s, 3H), 1.65-1.53 (m, 6H) *cis* rotamer: 7.22 (d, *J* = 8.85 Hz, 2 H), 6.85 (d, *J* = 8.85 Hz, 2 H), 4.81 (s, broad, 1H), 4.01 (s, 2H), 3.92 (s, 2H), 3.83 (s, 3H), 3.52 (t, *J* = 5.77 Hz, 2 H), 3.22 (t, *J* = 5.56 Hz, 2 H), 2.03 (s, 3H), 1.65-1.53 (m, 6H); **^{13}C NMR** (125 MHz, $CDCl_3$) δ 175.07, 166.57, 159.14, 130.17, 129.24, 113.88, 55.28, 51.52, 46.10, 42.96, 42.05, 26.28, 25.44, 24.37, 20.92; **IR** (neat) 3276, 2929, 2852, 1634, 1442, 1249, 1032, 849, 803, 585, 542 **HRMS** (HESI/orbitrap) calculated for [$C_{17}H_{25}N_3O_3$] calculated *m/z* $[M + H]^+$ =320.1969, observed *m/z* $[M + H]^+$ = 320.1965; **MP** = 84.6 °C – 87.6 °C.

***N*-(2-oxo-2-(piperidin-1-yl)ethyl)-N'-(4-(trifluoromethyl)benzyl)acetohydrazide (21c)**

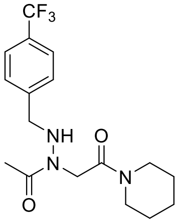

21c was synthesized according to the procedure described above for the synthesis of **21a** from **20c** (150 mg, 0.42 mmol). The oil was purified by flash chromatography on the Biotage® Isolera with a 2-8% MeOH in CH_2Cl_2 gradient to give **21c** as a white solid (0.1268 g, 0.35 mmol, 84% yield). **Rf** = 0.42 (5% MeOH in CH_2Cl_2), product stains with $KMnO_4$; **^{1}H NMR** (500 MHz, $CDCl_3$): *trans* rotamer: 7.59 (d, 2H), 7.45 (d, 2H), 5.12 (t, *J* = 5.23 Hz, 1H), 4.44 (s, 2 H), 3.98 (d, 2 H), 3.52 (t, *J* = 5.23 Hz, 2H), 3.39 (t, *J* = 5.23 Hz, 2H), 2.12 (s, 3H), 1.67-1.54 (m, 6H) *cis* rotamer: 7.59 (d, 2H), 7.45 (d, 2H), 5.12 (t, *J* = 5.23 Hz, 1H), 4.05 (s, 2 H), 4.04 (s, 2H), 3.52 (t, *J* = 5.23 Hz, 2H), 3.22 (t, *J* = 5.52 Hz, 2H), 2.03 (s, 3H), 1.67-1.54 (m, 6H); **^{13}C NMR** (125 MHz, $CDCl_3$) δ 175.15, 166.62, 141.40, 130.52 (q, *J* = 32.4 Hz),129.31, 125.63 (q, *J* = 3.7 Hz), 51.91, 46.24, 43.11, 42.13, 26.42, 25.55, 24.46, 21.09; **IR** (neat) 2950, 2860, 1633,1447, 1405, 1324,

1158, 1110, 1066, 1018, 814, 801, 615, 590, 557. **HRMS** (HESI/orbitrap) calculated for [$C_{17}H_{22}F_3N_3O_2$] calculated *m/z* $[M + H]^+$ = 358.1737, observed *m/z* $[M + H]^+$ = 358.1733; **MP** = 138.6 °C – 141.6 °C.

2-(phenethylamino)-1-(piperidin-1-yl)ethan-1-one (22)

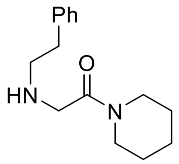

22 was synthesized according to the procedure described above for the synthesis of **19a** from **18** (5.0 mmol) and 2-phenylethylamine (1.3 mL, 10 mmol). The product was purified by flash chromatography with a Biotage® Isolera using a 3-8% MeOH in CH_2Cl_2 gradient to give **22** as a dark orange oil (0.8892 g, 3.6 mmol, 72% yield over two steps): **Rf** = 0.29 (5% MeOH in CH_2Cl_2); **^{1}H NMR** (600 MHz, $CDCl_3$) δ 7.26-7.15 (m, 5H), 3.50 (t, *J* = 5.63 Hz, 2H), 3.38 (s, 2H), 3.25 (t, *J* = 5.58 Hz, 2H), 2.86-2.83 (m, 2H), 2.81-2.78 (m, 2H), 2.33 (broad s, 1H), 1.59-1.48 (m, 6H); **^{13}C NMR** (150 MHz, $CDCl_3$) δ 168.89, 139.92, 128.63, 128.34, 126.03, 51.35, 50.28, 45.43, 42.88, 36.61, 26.24, 25.45, 24.42; **IR** (neat) 2933, 2853, 1635, 1440, 1252, 1228, 1121, 1011, 852, 748, 699, 588, 486. **HRMS** (HESI/orbitrap) calculated for [$C_{15}H_{22}N_2O$] calculated *m/z* $[M + H]^+$ = 247.1805, observed *m/z* $[M + H]^+$ = 247.1801.

***N*-(2-oxo-2-(piperidin-1-yl)ethyl)-*N*-phenethylacetamide (23)**

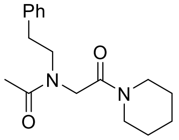

23 was synthesized according to the procedure described above for the synthesis of **20a** from **22** (800 mg, 3.2 mmol). The product was purified by flash chromatography with a Biotage® Isolera using a 3-10% MeOH in CH_2Cl_2 gradient to give **23** as an orange oil (0.5535 g, 1.9 mmol, 59% yield): **Rf** = 0.35 (5% MeOH in CH_2Cl_2); **^{1}H NMR** (600 MHz, $CDCl_3$) δ *trans* rotamer: 7.30-7.15 (m, 5H), 4.10 (s, 2H), 3.62 (t, *J* = 7.38 Hz, 2H), 3.52 (t, *J* = 5.74 Hz, 2H), 3.33 (t, *J* = 5.57 Hz, 2H), 2.86 (t, *J* = 7.38 Hz, 2H), 1.98 (s, 3H), 1.63-1.52 (m, 6H) *cis* rotamer: 7.30-7.15 (m, 5H), 3.79 (s, 2H), 3.59 (t, *J* = 7.38 Hz, 2H), 3.52 (t, *J* = 5.74 Hz, 2H), 3.17 (t, *J* = 5.61 Hz, 2H), 2.86 (t, *J* = 7.38 Hz, 2H), 1.98 (s, 3H), 1.63-1.52 (m, 6H); **^{13}C NMR** (150 MHz, $CDCl_3$) δ 171.05, 166.39, 138.50, 128.83, 128.56, 126.8, 51.17, 46.59, 46.13, 43.23, 35.06, 26.39, 25.57, 24.56, 21.04; **IR** (neat) 2935, 1636, 1440, 1251, 1226, 1003, 746, 700, 497. **HRMS** calculated for [$C_{17}H_{24}N_2O_2$] calculated *m/z* $[M + H]^+$ = 289.1911, observed *m/z* $[M + H]^+$ = 289.1916

Compounds **18, 19a, 20a-c, 21a-c, 22,** and **23** were characterized by ^{1}H and ^{13}C NMR in $CDCl_3$ (see pp. S61-69). Compounds **20a**, **21a,** and **23** were further characterized in three different solvents ($CDCl_3$, CD_3CN, and CD_3OD) and four concentrations (1, 5, 10, and 100 mM) to measure average $K_{cis/trans}$ values reported in Table 2 of the main text. ^{1}H NMRs of **20a** and **21a** were also taken at temperatures ranging from −35°C to 45°C. ^{1}H and NOESY experiments were used to assign proton signals and analyze interactions through space to identify *cis*- or *trans*- amide configurations of **20a**, **21a,** and **23**.

12. Variable solvent and concentration: 1D spectra were collected for samples prepared at 100, 10, 5, and 1 mM concentrations at 25 °C in three solvents ($CDCl_3$, CD_3OD, and CD_3CN) for **20a**, **21a,** and **23**.

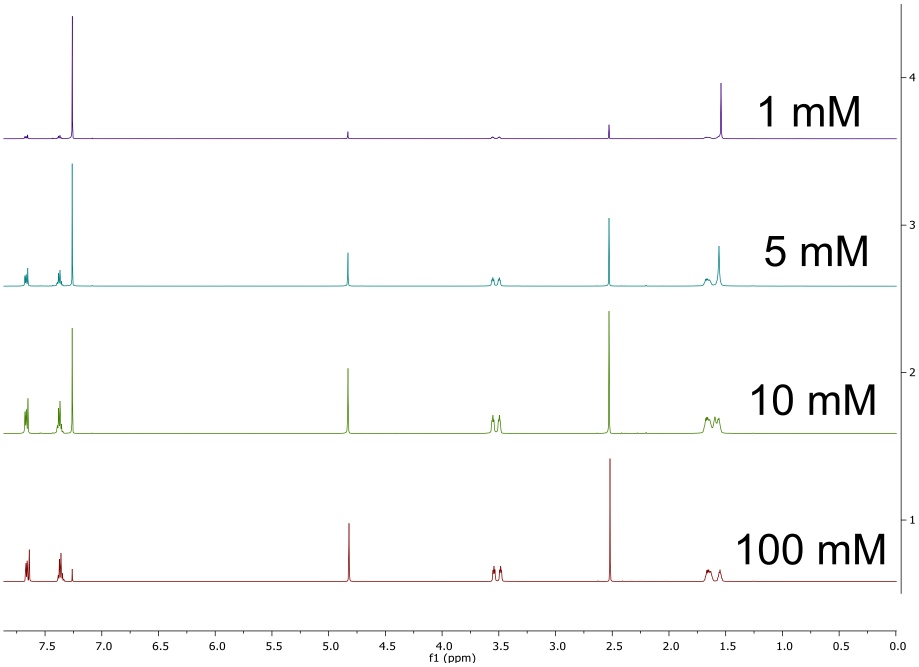

Chemical shifts for all peaks remained the same throughout.

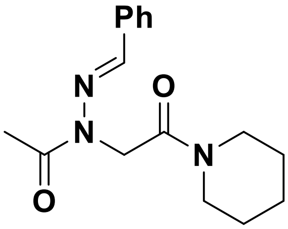

20a

Figure S17. ^{1}H NMR (600 MHz, $CDCl_3$) 1mM, 5 mM, 10mM and 100 mM (top to bottom) of **20a**

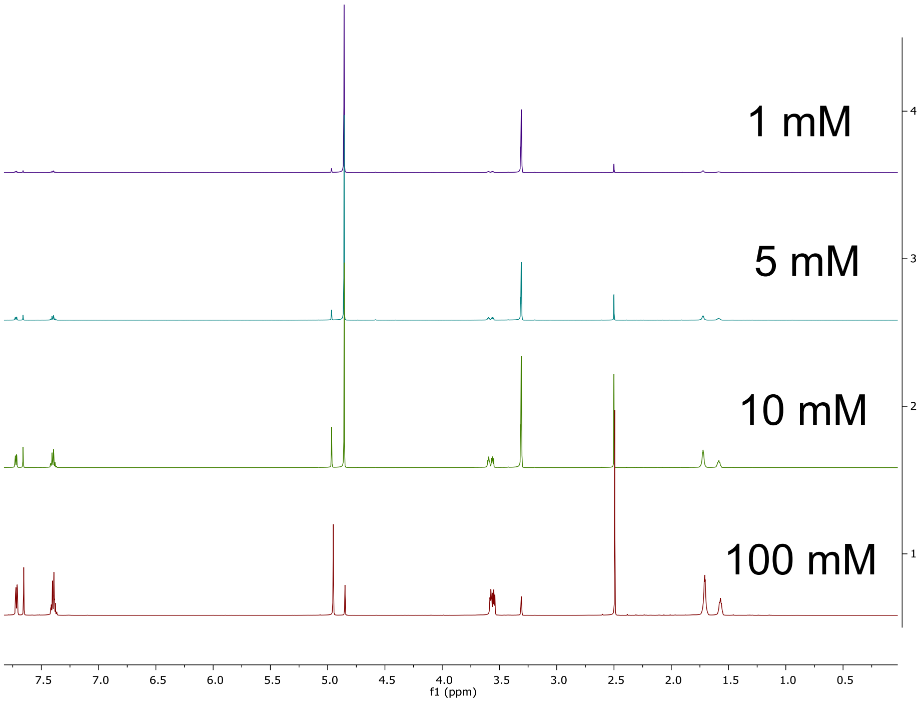

Chemical shifts for all peaks remain the same for 10, 5, and 1 mM with 100 mM shifts off by 0.01 ppm.

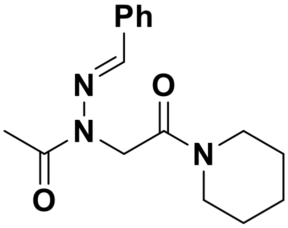

20a

Figure S18. ^{1}H NMR (600 MHz, CD_3OD) 1mM, 5 mM, 10mM and 100 mM (top to bottom) of **20a**

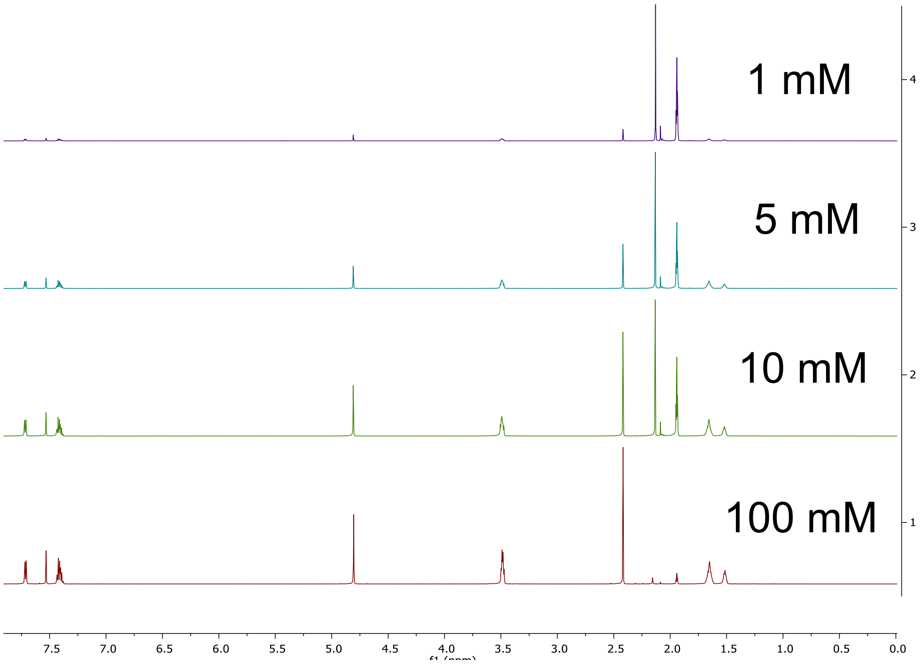

Chemical shifts for all peaks remain the same for 10, 5, and 1 mM with 100 mM shifts off by 0.01 ppm.

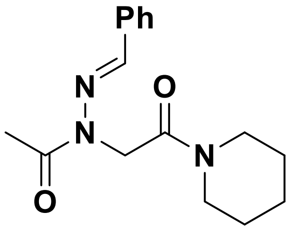

20a

Figure S19. ^{1}H NMR (600 MHz, CD_3CN) 1mM, 5 mM, 10mM and 100 mM (top to bottom) of **20a**

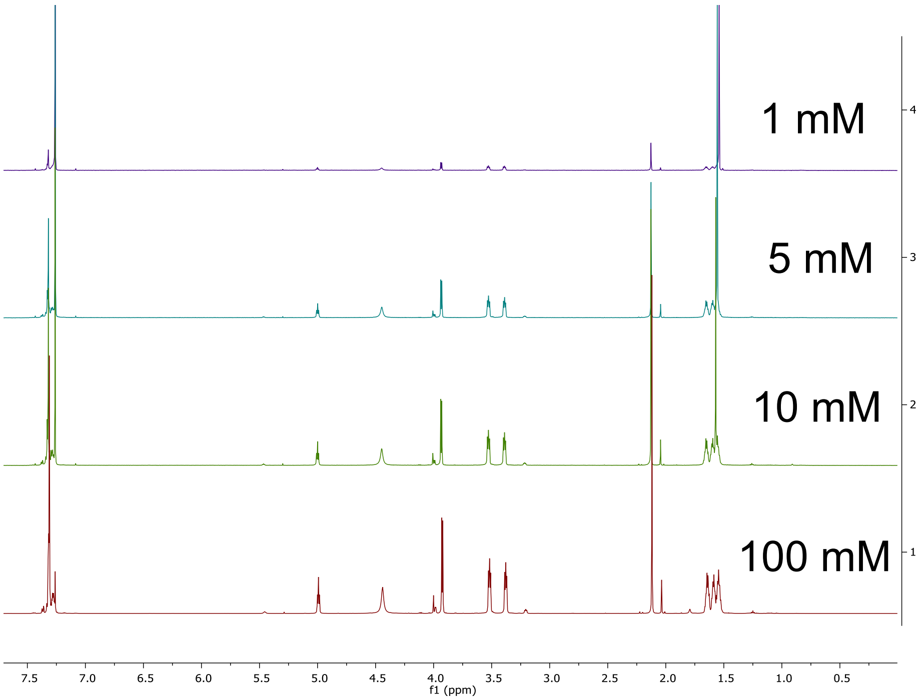

Chemical shifts for all peaks remained the same throughout.

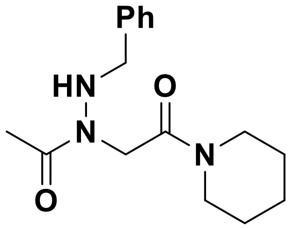

21a

Figure S20. ^{1}H NMR (600 MHz, $CDCl_3$) 1mM, 5 mM, 10mM and 100 mM (top to bottom) of **21a**

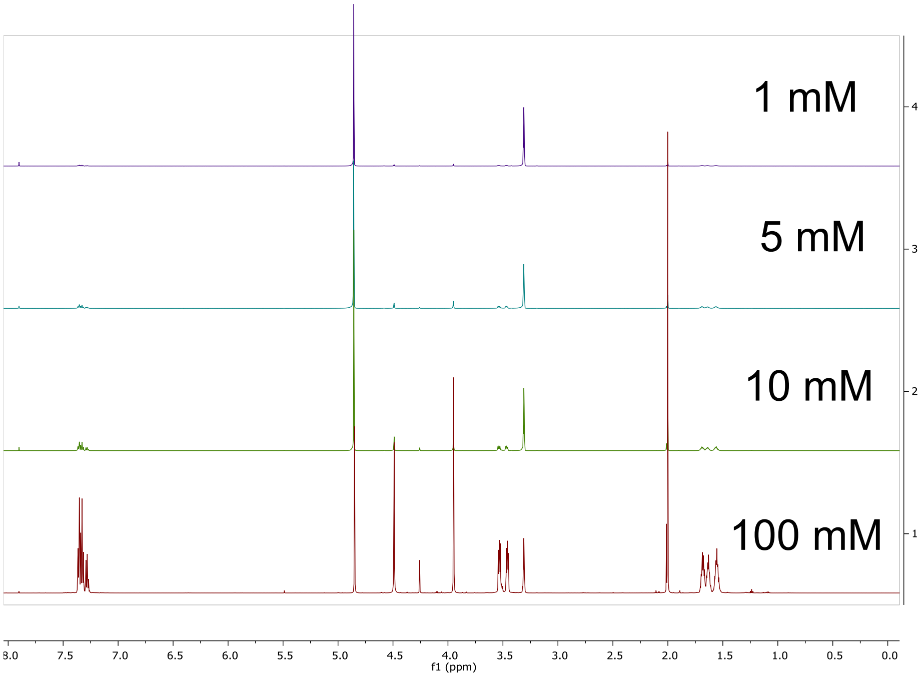

Chemical shifts for all peaks remain the same for 10, 5, and 1 mM with 100 mM shifts off by 0.01 ppm.

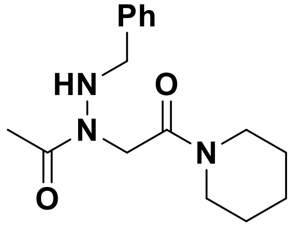

21a

Figure S21. ^{1}H NMR (600 MHz, CD_3OD) 1mM, 5 mM, 10mM and 100 mM (top to bottom) of **21a**

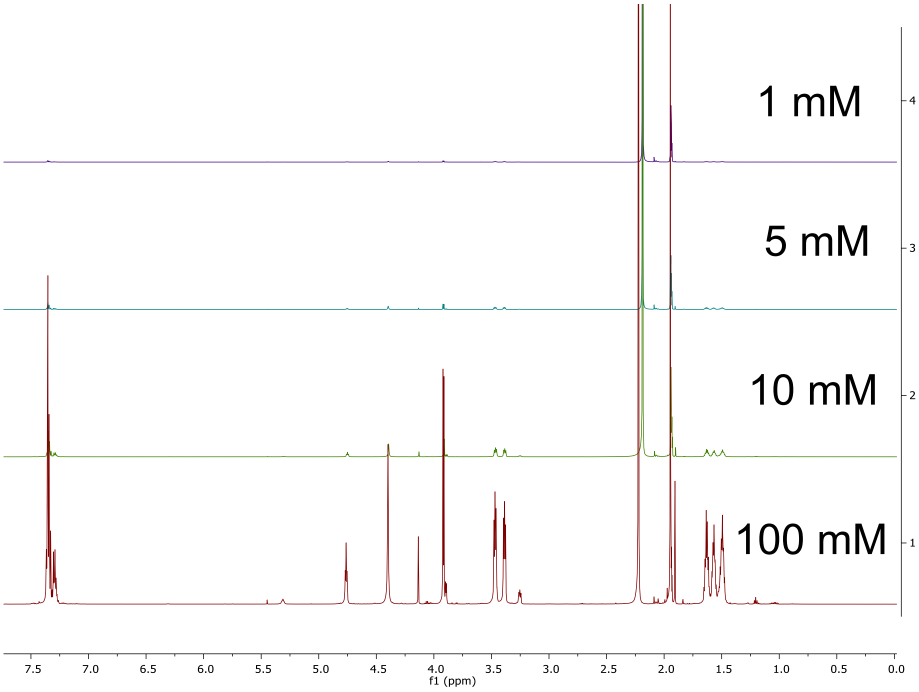

Chemical shifts for all peaks remain the same for 10, 5, and 1 mM with 100 mM shifts off by 0.01 ppm.

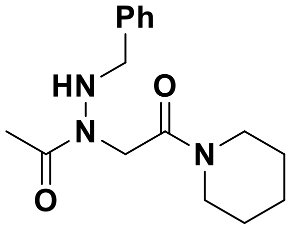

21a

Figure S22. ^{1}H NMR (600 MHz, CD_3CN) 1mM, 5 mM, 10mM and 100 mM (top to bottom) of **21a**

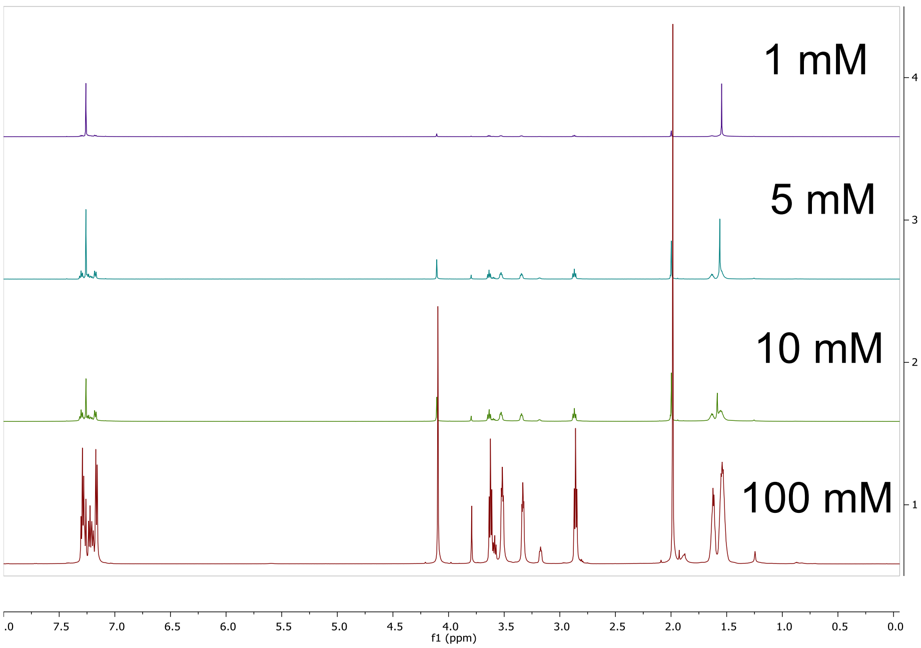

Chemical shifts for all peaks remain the same for 10, 5, and 1 mM with 100 mM shifts off by 0.02 ppm.

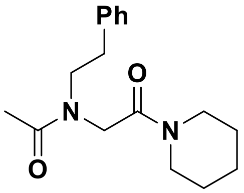

23

Figure S23. ^{1}H NMR (600 MHz, $CDCl_3$) 1mM, 5 mM, 10mM and 100 mM (top to bottom) of **23**

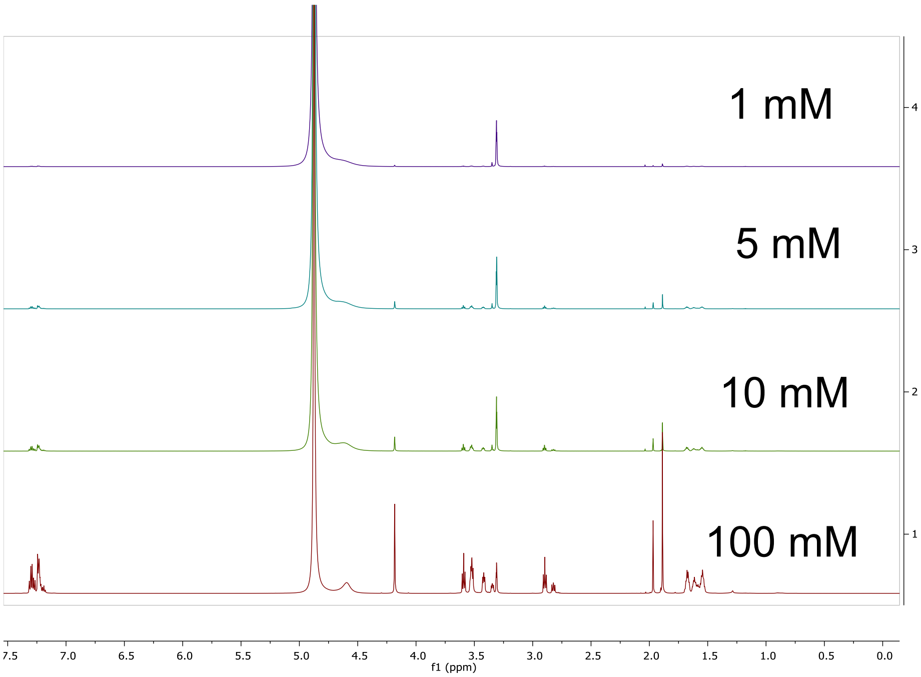

Chemical shifts for all peaks remain the same for all concentrations.

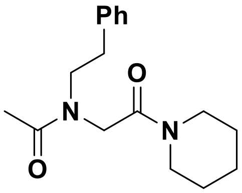

23

Figure S24. ^{1}H NMR (600 MHz, CD_3OD) 1mM, 5 mM, 10mM and 100 mM (top to bottom) of **23**

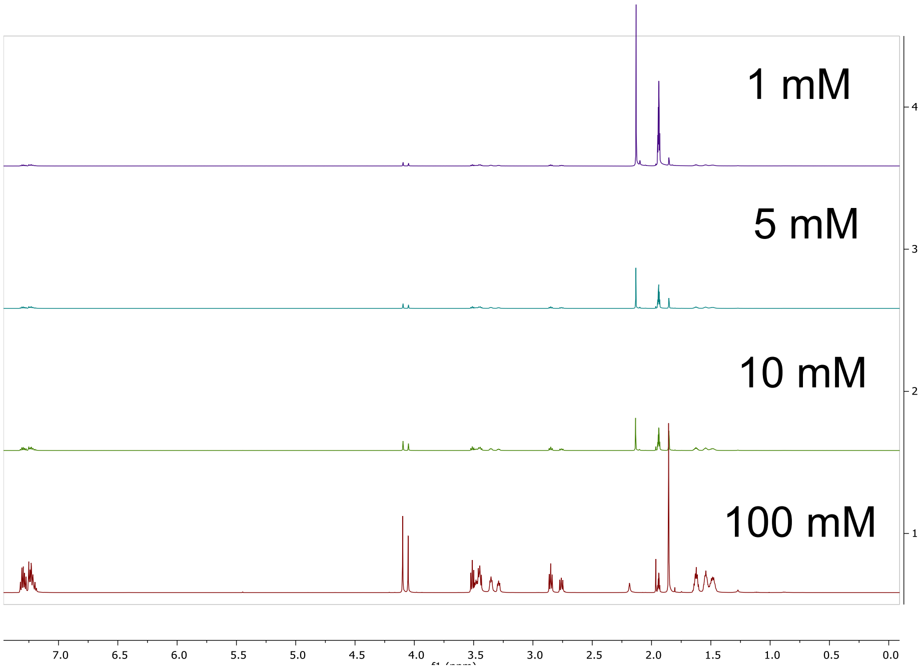

Chemical shifts for all peaks remain the same for 10, 5, and 1 mM with 100 mM shifts off by 0.01 ppm.

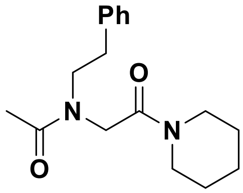

23

Figure S25. ^{1}H NMR (600 MHz, CD_3CN) 1mM, 5 mM, 10mM and 100 mM (top to bottom) of **23**

13. Analysis of peptoid 21a

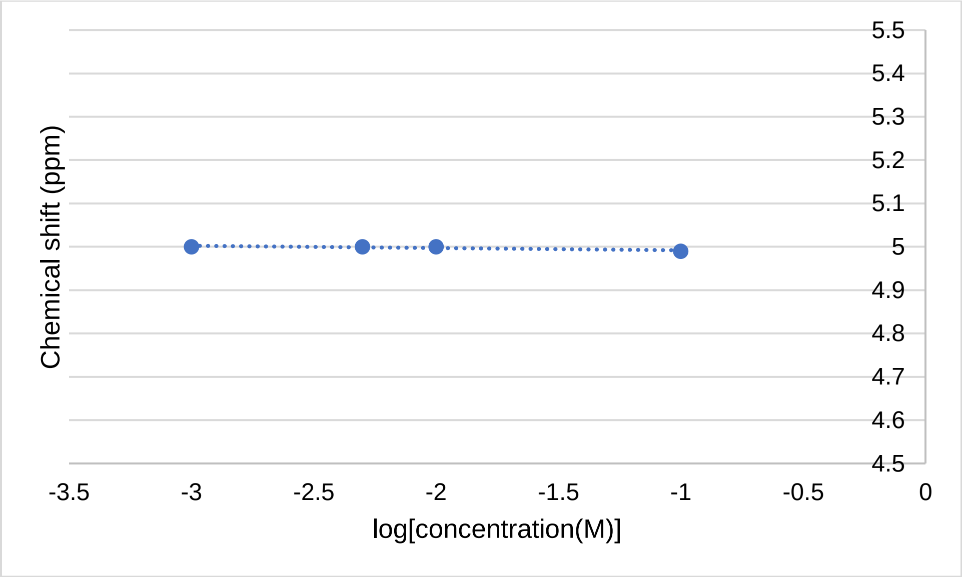

Figure S26. NMR chemical shift of the NH proton of the amino alkyl side chain of **21a** in $CDCl_3$ at room temperature, as a function of the logarithm of concentration in M.

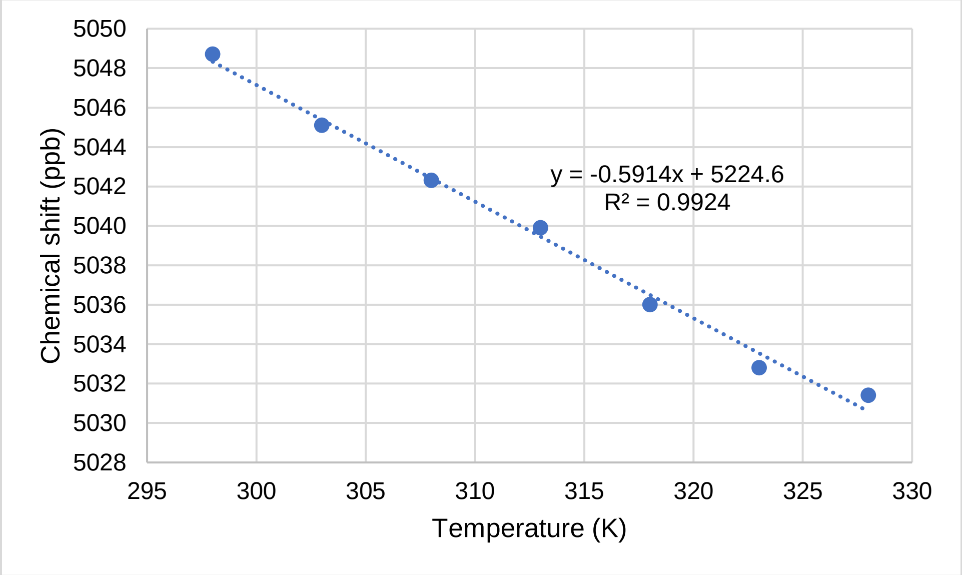

Figure S27. Temperature coefficient (T_{coeff}) of compound **21a** in DMSO-d_6 determined using the ^{1}H-NMR chemical shift of the NH proton of the amino alkyl side chain of **21a** at 17 mM with respect to temperature (25-55 °C). T_{coeff} = –0.5914, implying intramolecular hydrogen bonding in the molecule.

14. $K_{cis/trans}$ values

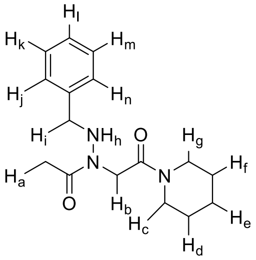

General procedure for NMR $K_{cis/trans}$ determination: $K_{cis/trans}$ is reported as the average value of the ratio between the *cis* and *trans* pairs of signals arising from two sets of rotamer related peaks[7]: the backbone methylene protons (H_b) and the methyl acetyl protons (H_a). Mean values are given with the corresponding standard deviation. The values observed in each solvent are shown in **Table S8** below.

Table S11. $K_{cis/trans}$ values

Peptoid	^1H-NMR (*$CDCl_3$*)								AVG $K_{cis/trans}$	$^{AVG}\Delta G$ *cis/trans* (kcal/mol)
	^1H_a, *cis/trans* 100 mM	^1H_a, *cis/trans* 10 mM	^1H_a, *cis/trans* 5 mM	^1H_a, *cis/trans* 1 mM	^1H_b, *cis/trans* 100 mM	^1H_b, *cis/trans* 10 mM	^1H_b, *cis/trans* 5 mM	^1H_b, *cis/trans* 1 mM		
**20a**[a]	<0.05	<0.05	<0.05	<0.05	<0.05	<0.05	<0.05	<0.05	<0.05	
**21a**	0.11	0.14	0.13	0.14	<0.05	<0.05	<0.05	<0.05	0.13± 0.01	1.22± 0.06
**23**	--[b]	--[b]	--[b]	--[b]	0.24	0.25	0.21	0.35	0.26± 0.05	0.80± 0.11

Peptoid	^1H-NMR (*CD_3CN*)								AVG $K_{cis/trans}$	$^{AVG}\Delta G$ *cis/trans* (kcal/mol)
	^1H_a, *cis/trans* 100 mM	^1H_a, *cis/trans* 10 mM	^1H_a, *cis/trans* 5 mM	^1H_a, *cis/trans* 1 mM	^1H_b, *cis/trans* 100 mM	^1H_b, *cis/trans* 10 mM	^1H_b, *cis/trans* 5 mM	^1H_b, *cis/trans* 1 mM		
**20a**[a]	<0.05	<0.05	<0.05	<0.05	<0.05	<0.05	<0.05	<0.05	<0.05	
**21a**	--[c]	--[c]	--[c]	--[c]	0.15	0.17	0.17	0.19	0.17± 0.01	1.05± 0.05
**23**	--[b]	--[b]	--[b]	--[b]	0.74	0.67	0.73	0.74	0.72± 0.03	0.19± 0.02

Peptoid	^1H-NMR (*CD_3OD*)								AVG $K_{cis/trans}$	$^{AVG}\Delta G$ *cis/trans* (kcal/mol)
	^1H_a, *cis/trans* 100 mM	^1H_a, *cis/trans* 10 mM	^1H_a, *cis/trans* 5 mM	^1H_a, *cis/trans* 1 mM	^1H_b, *cis/trans* 100 mM	^1H_b, *cis/trans* 10 mM	^1H_b, *cis/trans* 5 mM	^1H_b, *cis/trans* 1 mM		
**20a**[a]	<0.05	<0.05	<0.05	<0.05	<0.05	<0.05	<0.05	<0.05	<0.05	
**21a**	0.15	0.17	0.16	0.16	0.15	0.18	0.16	0.19	0.16± 0.01	1.07± 0.05
**23**	0.45	0.44	0.45	0.42	--[b]	--[b]	--[b]	--[b]	0.44± 0.01	0.48± 0.02

[a]No additional peaks for a minor conformer were detected. [b]Shoulder begins to appear that may represent different rotamers but are not fully resolved. [c]The residual solvent peak has the same ppm shift as the acetyl group; *cis/trans* ratios were measured using the backbone methylene peaks only.

Table S12. $K_{cis/trans}$ values of **20a-c**, **21a-c**, and **23** at 100 mM in $CDCl_3$

Peptoid	^{1}H-NMR (*$CDCl_3$*)	
	$^1H_{a,\ cis/trans}$ 100 mM	$^1H_{b,\ cis/trans}$ 100 mM
**20a**[a]	<0.05	<0.05
**21a**	0.11	<0.05
**20b**[a]	<0.05	<0.05
**21b**	0.10	0.14
**20c**[a]	<0.05	<0.05
**21c**	0.16	--[b]
**23**	--[b]	0.24

[a]No additional peaks for a minor conformer were detected for any *N*-imino monomers (**20a-c**) [b]Shoulder begins to appear that may represent different rotamers, but they are not fully resolved at this concentration

15. Variable temperature NMRs for peptoids 19a and 20a

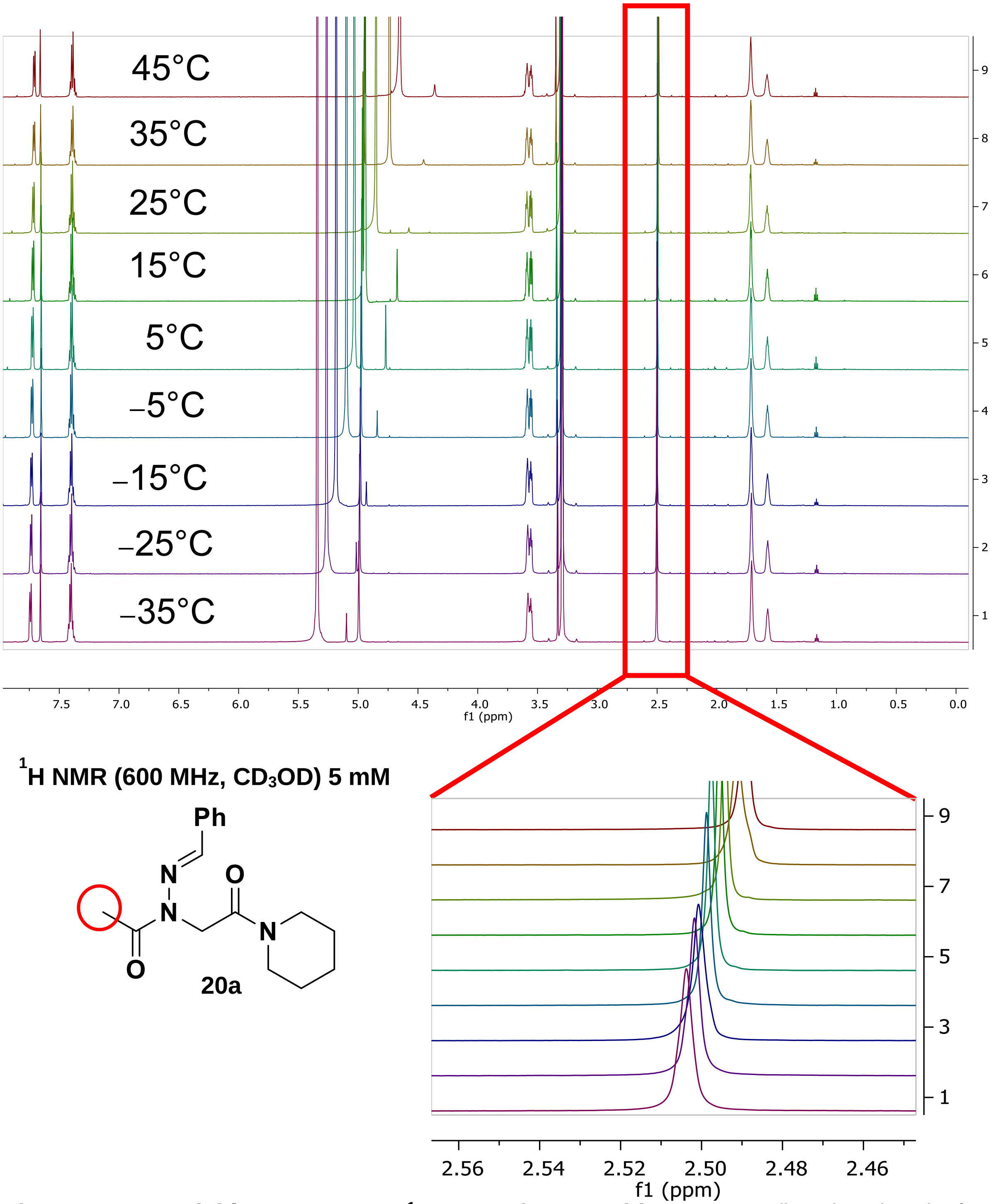

Figure S28a. Variable temperature ^{1}H NMRs for peptoid 20a To confirm that the single peak was due to the presence of a single conformer, ^{1}H NMRs were taken at a range of temperatures (−35°C to 45ºC), presented below. The acetyl peak for monomer **20a** shifted downfield, but did not split into two peaks or broaden considerably over this temperature range.

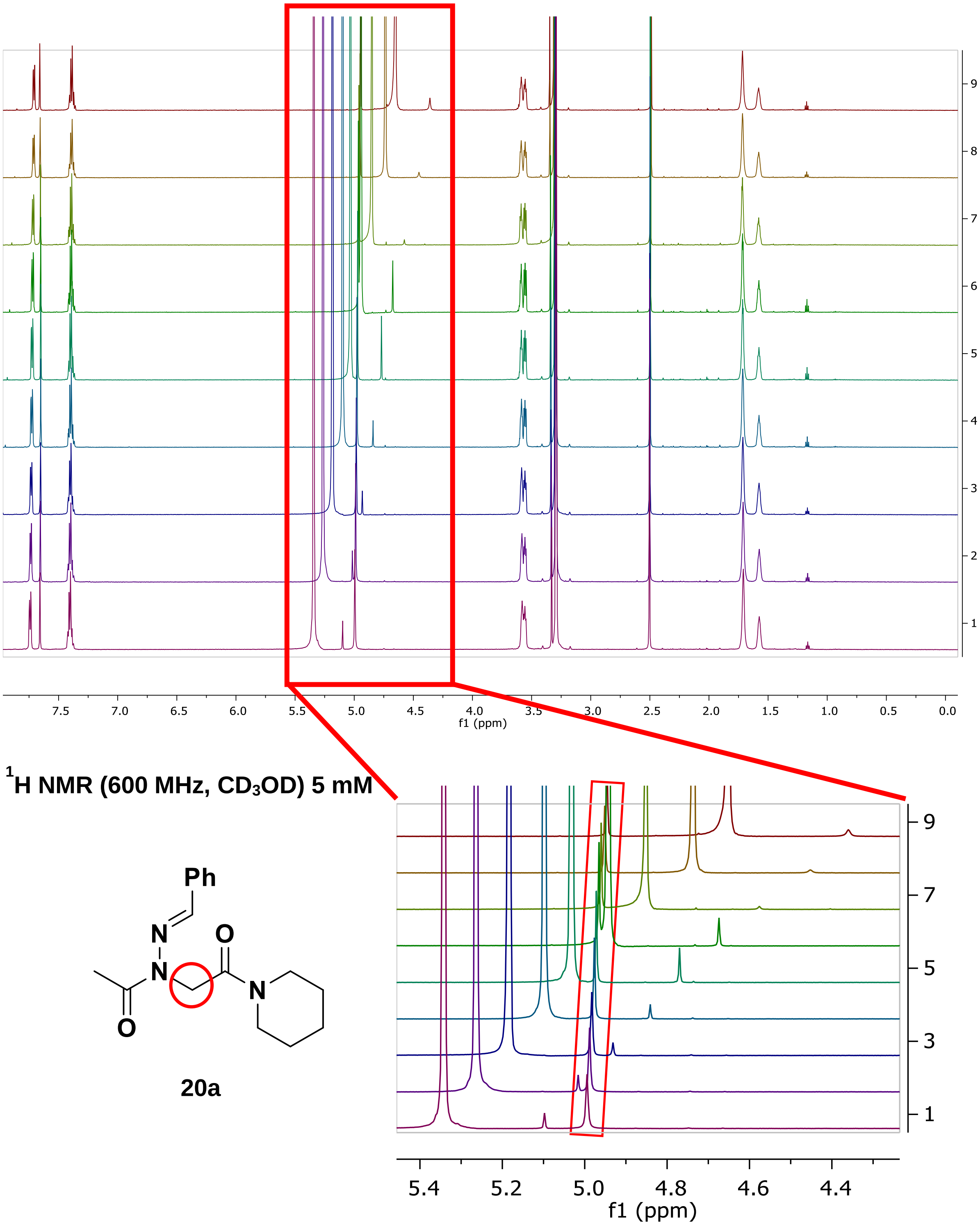

Figure S28b. Variable temperature ^{1}H NMRs for peptoid 20a The backbone methylene proton singlet (δ 4.96 ppm in CD_3OD at 5mM at 25ºC) also did not split into multiple peaks but instead shifted downfield slightly as the temperature was lowered. As the temperature is lowered, the water peaks (δ 4.85 and 4.48 ppm in CD_3OD at 5mM at 25ºC) shift downfield quickly at the same rate and overlap slightly with the CH_2 peak at 15°C. The backbone methylene peak broadens slightly as the temperature is lowered.

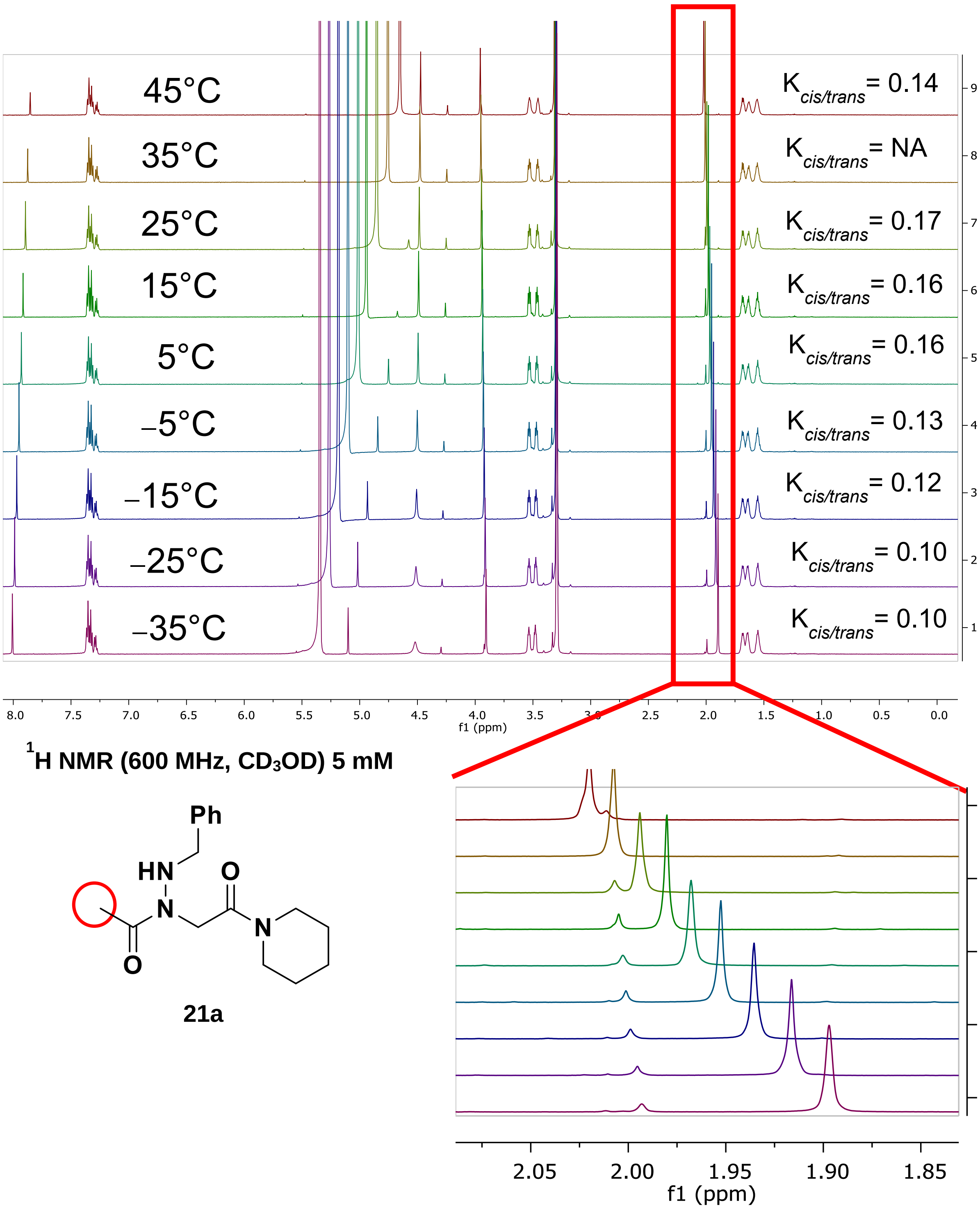

Figure S29a. Variable temperature ^{1}H NMRs for peptoid 21a The major peak (δ 1.99 in CD_3OD at 5 mM at 25°C) shifted downfield slightly as the temperature was increased (total of 0.12 ppm across the observed temperature range), while the minor peak shifted a total of 0.02 ppm across the observed temperature range with the major peak at 35°C. Although the peaks did not coalesce, the $K_{cis/trans}$ values trended lower as the temperature was lowered.

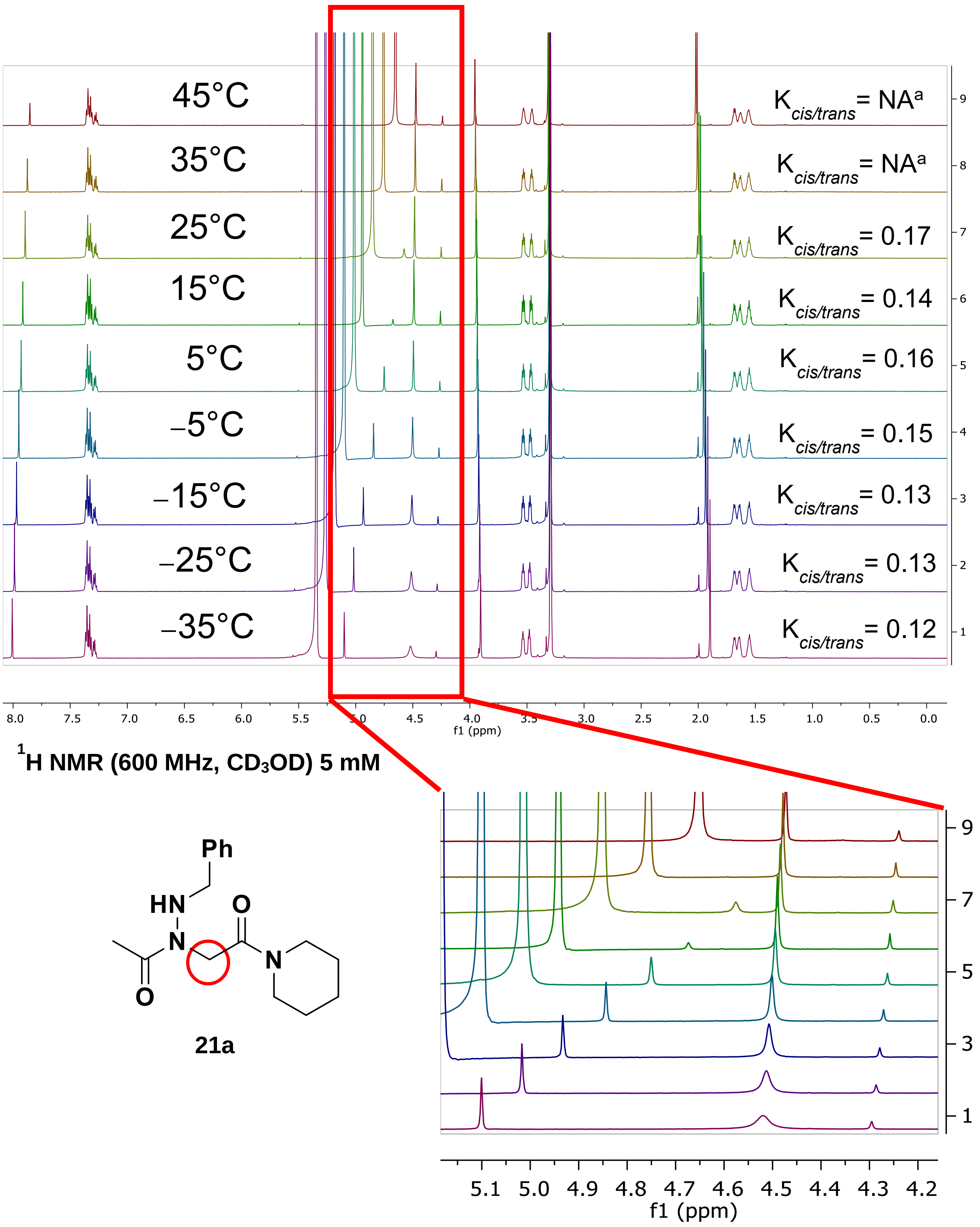

Figure S29b. Variable temperature ^{1}H NMRs for peptoid 21a The backbone methylene proton peaks (δ 4.48 and 4.25 ppm in CD_3OD at 5mM at 25ºC) also did not coalesce as the temperature was raised. Both peaks shifted upfield as the temperature increased. The major peak broadened as temperature was lowered.

[a]$K_{cis/trans}$ values are listed as NA because the water peak begins to overlap with the major peak at these temperatures

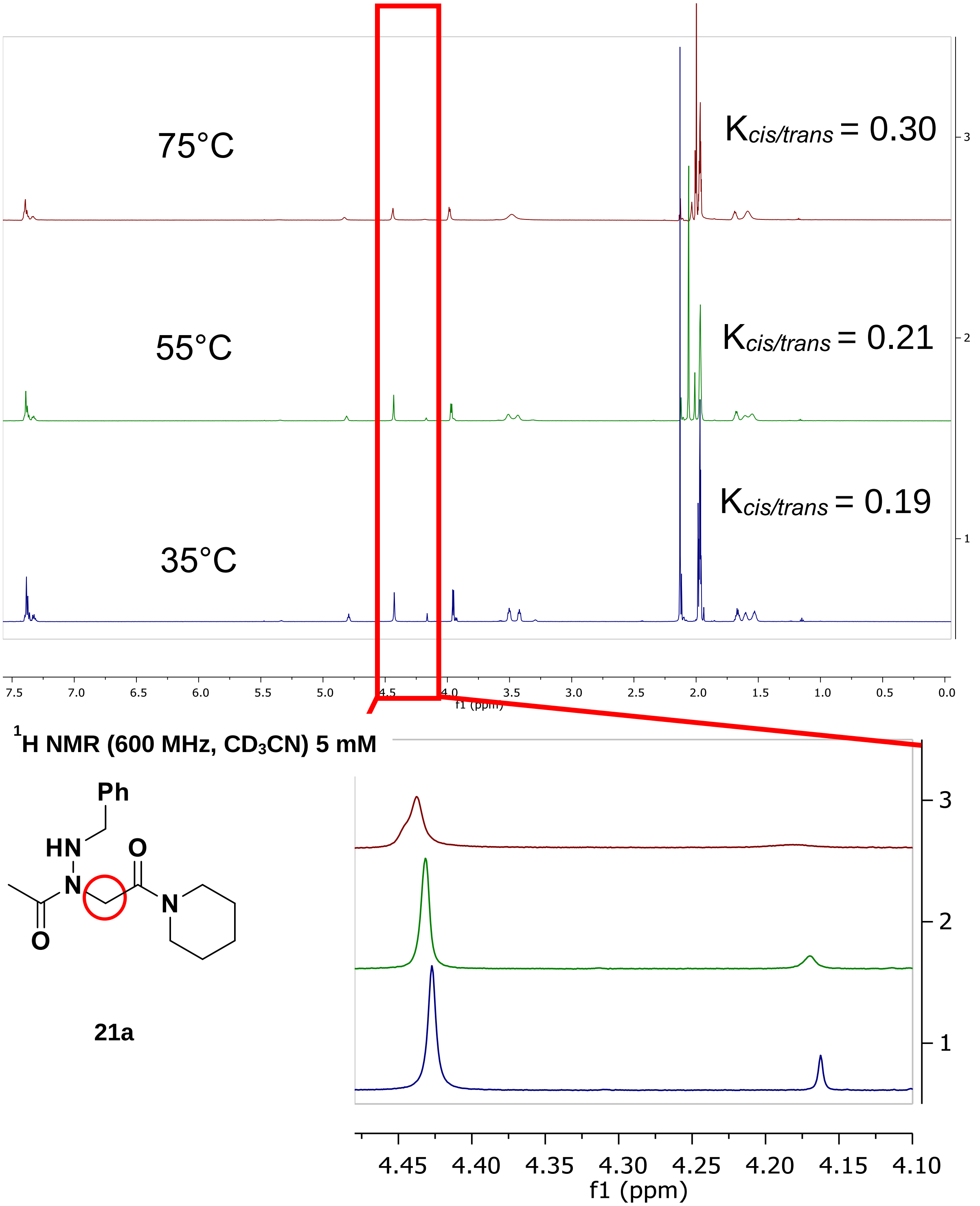

Figure S29c. Variable temperature tests of peptoid 21a To determine whether the peaks would coalesce at a higher temperature than 45 ºC, a ^{1}H NMR was taken of **21a** in CD_3CN at 5 mM at 35, 55, and 75 ºC. There was no detected coalescence at these temperatures in the backbone methylene peaks (the acetyl CH_3 peaks are obscured by the residual solvent), though there is a slight shift downfield as the temperature increases. The peaks also broaden and the $K_{cis/trans}$ increases as temperature does. The piperidine peaks do coalesce as the temperature increases.

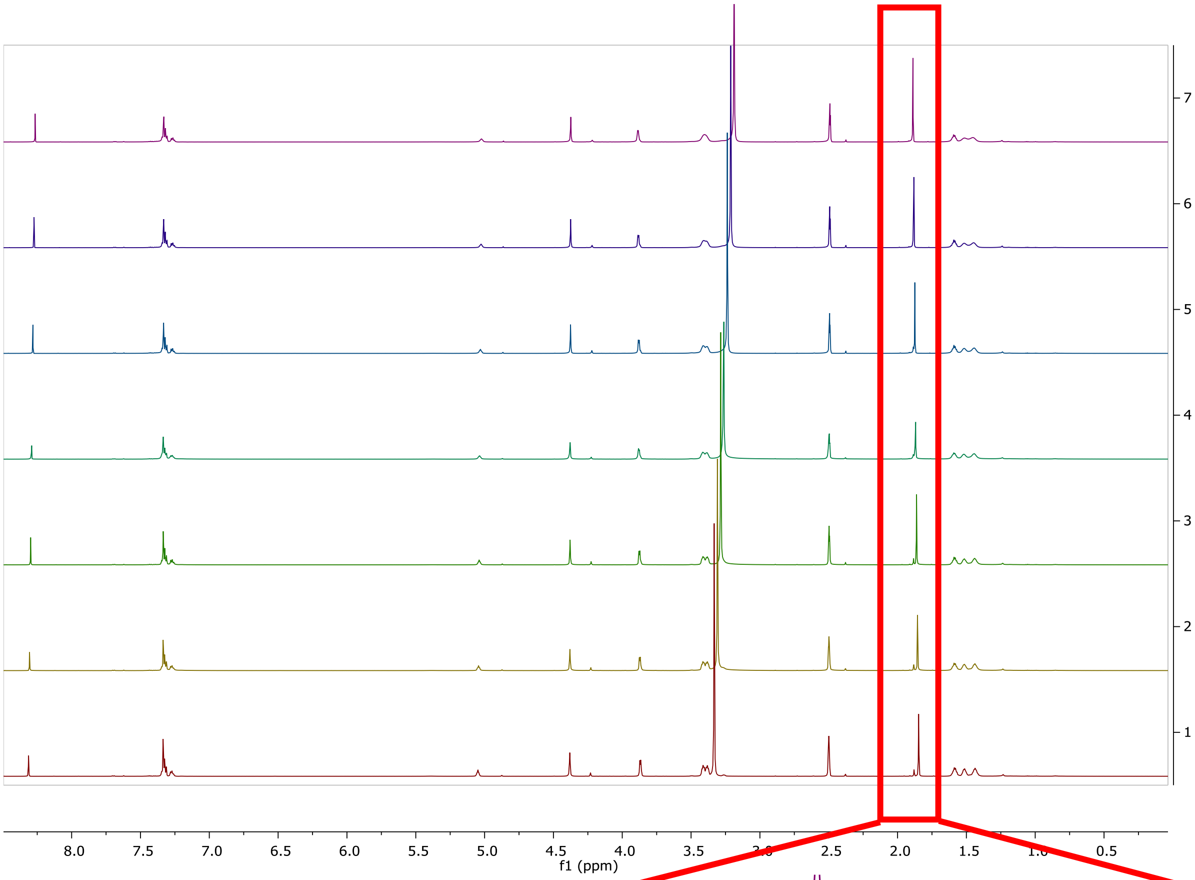

^{1}H NMR (600 MHz, DMSO-d6) 17 mM

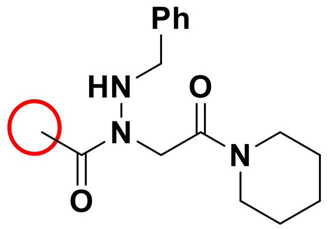

21a

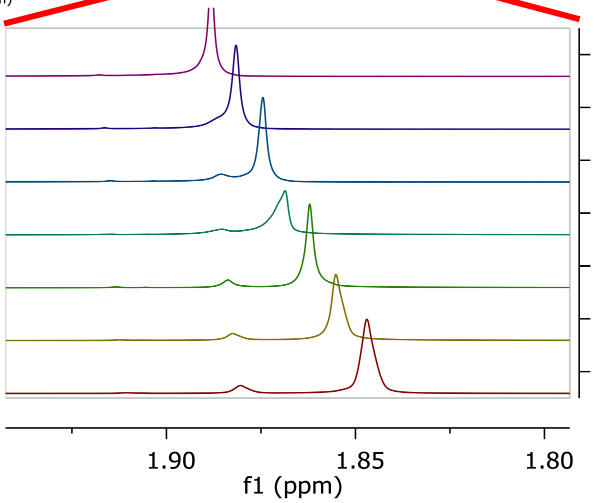

Figure S29d. Variable temperature tests of peptoid 21a Variable temperature NMRs were collected in DMSO-d6 to make $T_{coefficient}$ plot (see **Fig. S27**). Acetyl CH_3 peaks seem to coalesce at 55 ºC, but may just be crossing over as in CD_3OD.

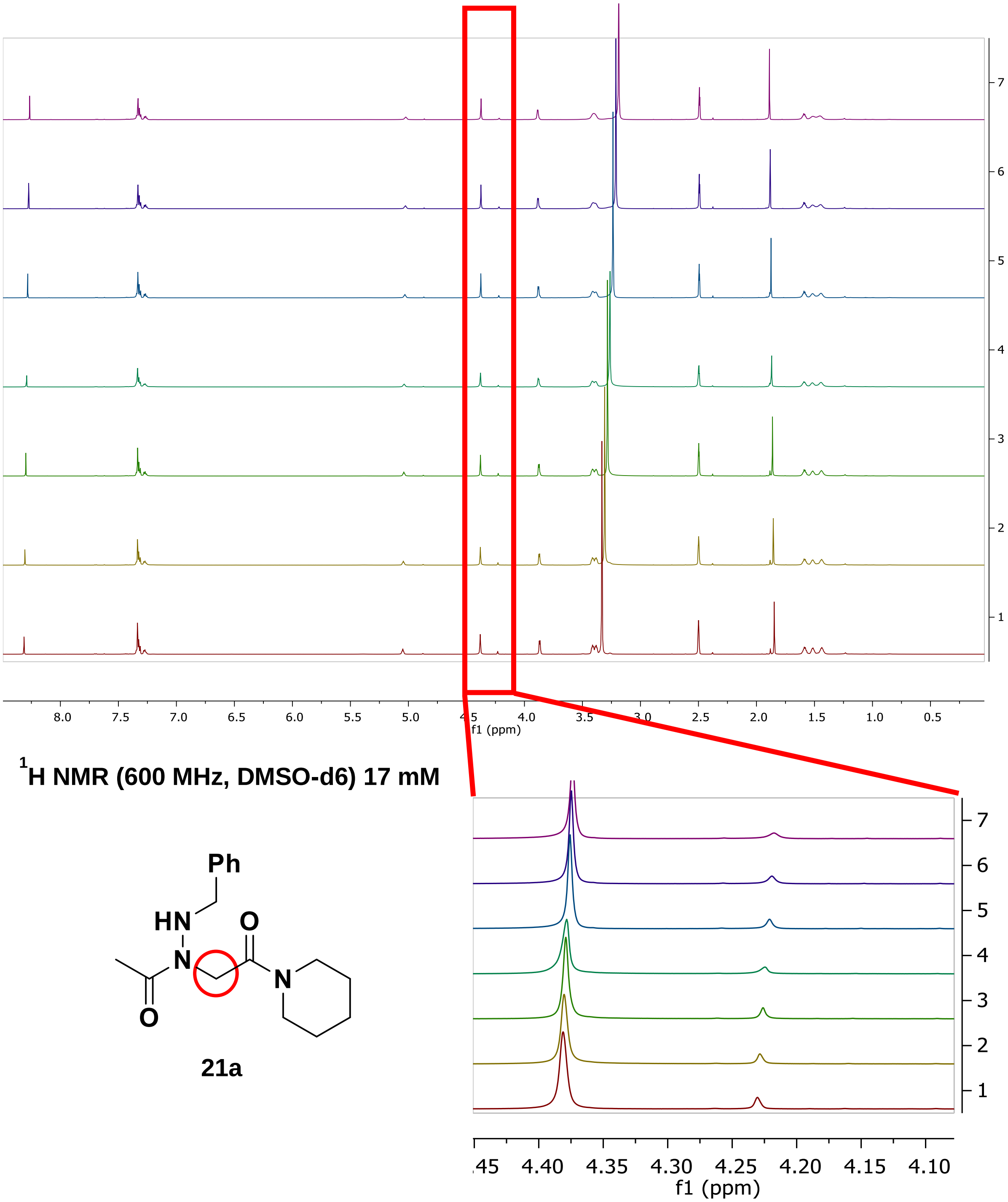

Figure S29e. Variable temperature tests of peptoid 21a Variable temperature NMRs were collected in DMSO-d6 to make $T_{coefficient}$ plot (see **Fig. S27**). Backbone CH_2 peaks shift very slightly upfield across the observed temperatures.

16. NOESY NMRs of 20a, 21a, and 23

Table S13. NOESY Parameters[6] for peptoids **20a, 21a,** and **23**

Spectral Width (ppm)	nt	ni (s)	Mix time (s)	d1 (s)
7.5	8	256	2	19

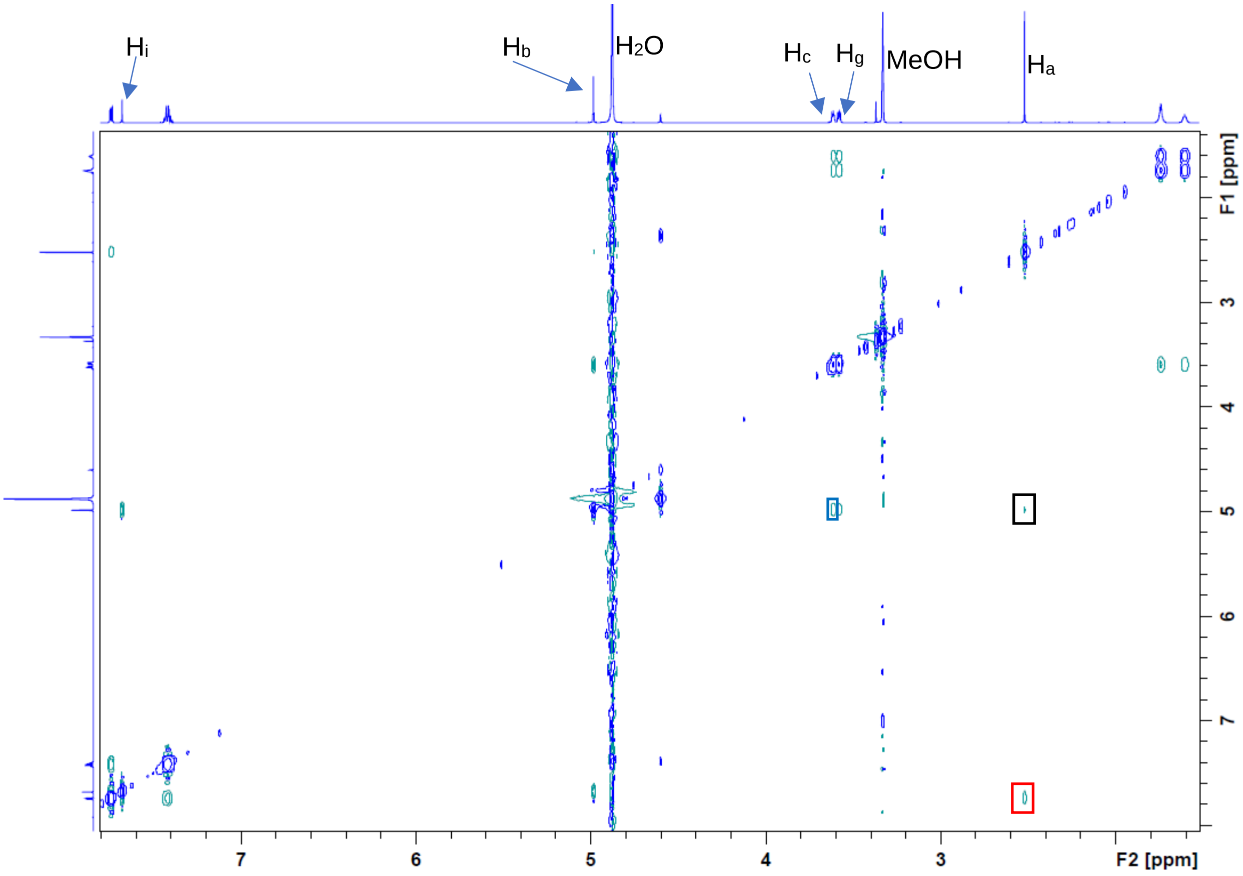

2D NOESY (700 MHz, CD_3OD) 10 mM

Figure S30. 2D NOESY NMR of peptoid **20a**

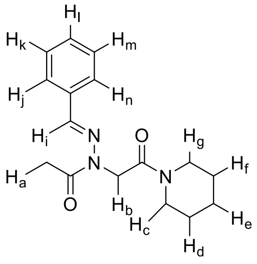

Positive crosspeaks are blue and negative are green. The acetyl/sidechain Ph (H_a/H_j) interaction [atomic distance of 3.18 Å from crystal structure] is boxed in red, and the acetyl/backbone methylene (H_a/H_b) interaction is boxed in black [atomic distance of 4.32 Å from crystal structure]. While still detectable, the H_a/H_b interaction is less intense than the H_a/H_j interaction, and also 13x less intense than the necessarily *cis* backbone methylene/piperidinyl interaction (H_b/H_c) boxed in blue [atomic distance of 2.03 Å from crystal structure].

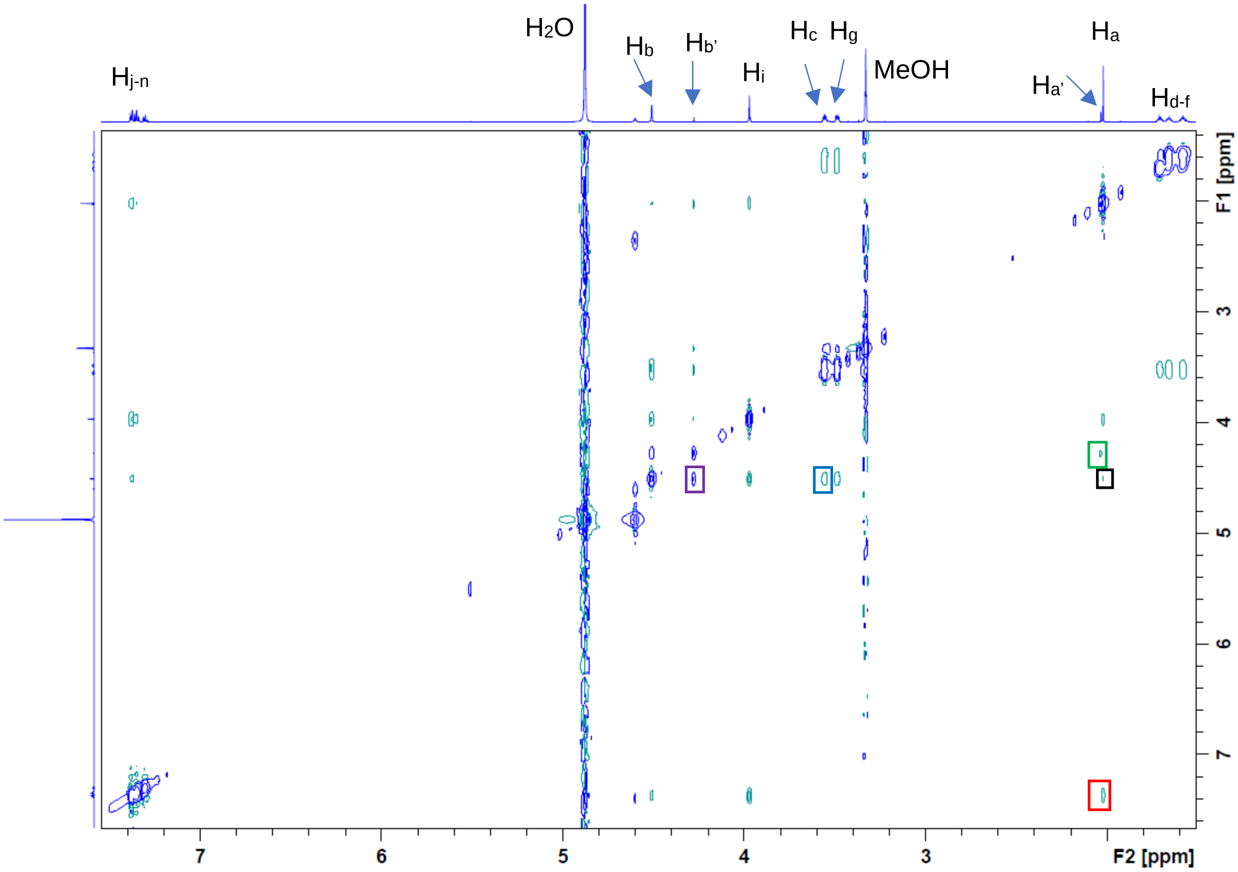

2D NOESY (700 MHz, CD_3OD) 10 mM

Figure S31. 2D NOESY NMR and crystal structure of peptoid **21a**

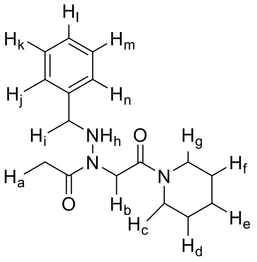

Positive cross-peaks are blue and negative are green. The expected *trans* acetyl/sidechain Ph (H_a/H_j) NOE [atomic distance of 3.14 Å from crystal structure] is boxed in red, and the small NOE between the acetyl/backbone methylene (H_a/H_b) interaction is boxed in black [atomic distance of 4.37 Å from crystal structure]. While still detectable because the atomic distance between the protons is within the upper limit detectable by NOESY and the long parameters used obtaining the NMR, the H_a/H_b interaction is less intense than the H_a/H_j interaction, and also 8x less intense than the necessarily *cis* backbone methylene/piperidinyl interaction (H_b/H_c) boxed in blue [atomic distance of 2.19 Å from crystal structure], suggesting that there are weak NOEs between the H_a/H_b protons in the *trans* conformation as well. A positive crosspeak (boxed in purple) between $H_b/H_{b'}$ indicate exchange between the two protons.

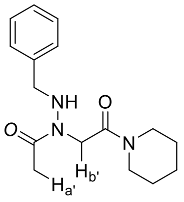

Boxed in green is an interaction between the acetyl and backbone methylene protons in what we believe is the *cis* conformer ($H_{a'}/H_{b'}$).

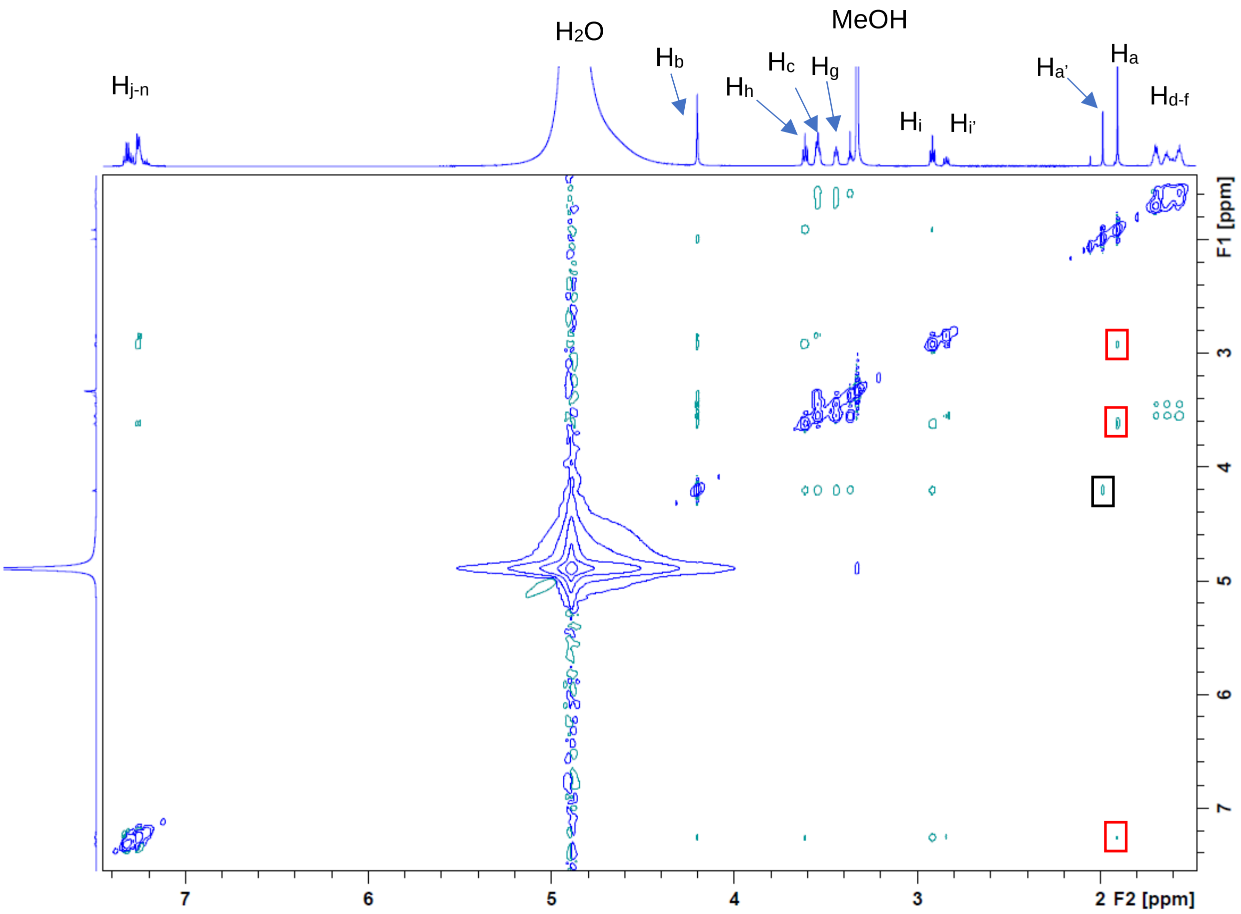

2D NOESY (600 MHz, CD_3OD) 10 mM

Figure S32. 2D NOESY NMR of peptoid **23**

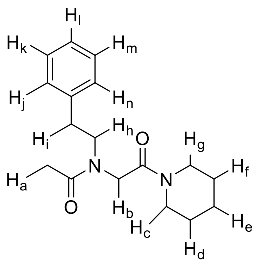

Positive cross-peaks are blue and negative are green. The expected *trans* acetyl/sidechain methylene and Ph (H_a/H_j, H_a/H_h, H_a/H_i) NOEs are boxed in red. The minor conformation acetyl peak ($H_{a'}$) has an interaction with the backbone methylene (H_b) boxed in black that isn't detected with H_a, evidence that the minor peak corresponds to the *cis* conformation.

17. Computational Studies. All computational studies were done with the Gaussian 2016[8] implementation of B3LYP.[9–12] The structures of **20a** and **21a** were generated using the coordinates from their respective X-ray crystal structures, and **23** by editing the crystal structure of **20a** in GaussView. The geometry of each structure was optimized in the gas phase using tight convergence criteria ("opt=tight") and pruned ultrafine grids ("int=ultrafine) with the 6-31G(d,p)[13] basis set. All structures were fully optimized and analytical frequency calculations were performed on all structures to ensure either a zeroth-order or first-order saddle point (a local minimum or transition state) was achieved. After optimizing the geometry of each structure, a relaxed potential energy scan was run about the ω dihedral angle of each structure with 36 steps of 10° at the same level of theory. Structures from the energy minima of each scan (ω = ~0° and ω = ~180°) were optimized once more at the same level of theory. For compound **21a** in the *cis* conformation (ω = ~0°), further investigation into the χ dihedral angle was done to further understand potential hydrogen bonding with the acetyl carbonyl (see **Figure S33**). A new energy minimum was found for the *cis* conformer by running another relaxed potential energy scan was run about the χ dihedral angle with 36 steps of 10° at the same level of theory. Energy calculations were done on optimized structures from the energy minima of each scan (B3LYP-D3/6-31G(d,p)) to obtain the reported ΔG values. Energies were calculated with the 6-311+G(2d,p)[14] basis set and also included the D3 version of Grimme's empirical dispersion correction.[15] Reported energies utilized analytical frequencies and the zero-point corrections from the gas phase optimized geometries, along with solvation corrections with the PCM solvation corrections method[16] in acetonitrile, as implemented in Gaussian 16. Natural bond orbital analysis was performed with NBO 6.0.[17,18]

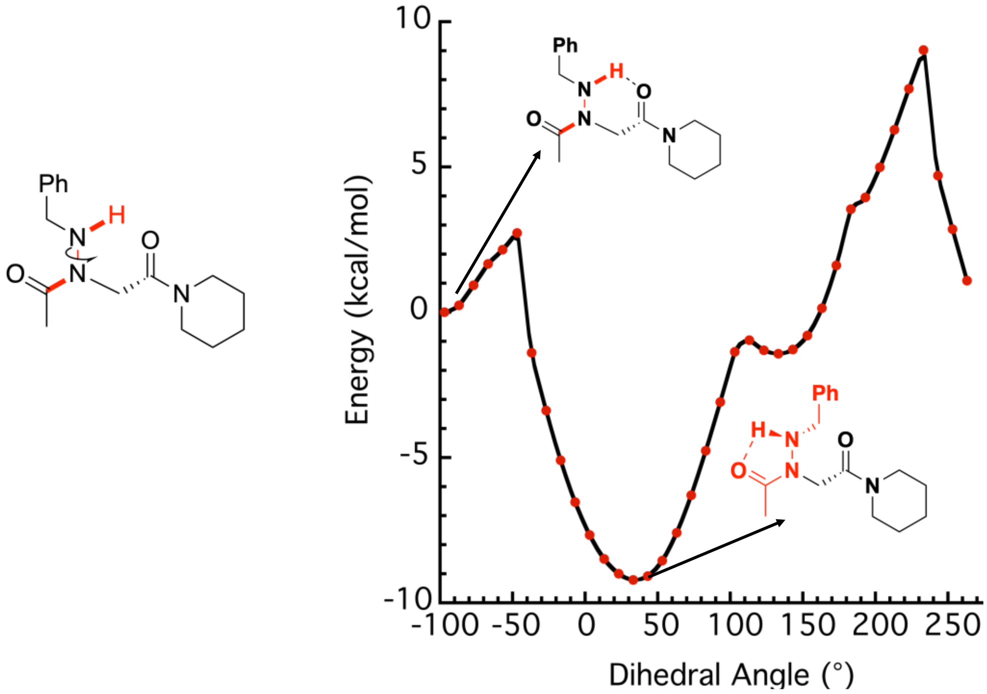

Figure S33. Potential energy surface scan for rotation around the χ (C–N–N–H) dihedral angle in the *cis* conformation of **21a**. Two minima were identified with hydrogen bonding to the carbonyl oxygens. A total of 36 points were scanned at 10° increments.

C. X-ray crystallographic information

X-ray crystallographic data for peptoids 20a-c and **21a-c**

Vapor diffusion of hexanes into ethyl acetate solutions of **20a-c** and **21a-c** provided crystals of all compounds suitable for X-ray analysis.

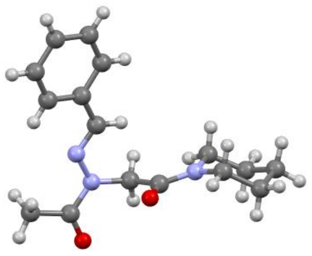

20a

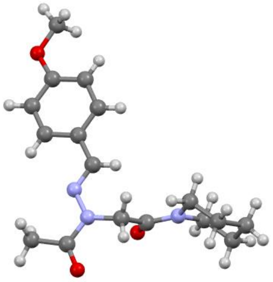

20b

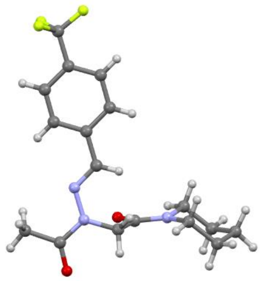

20c

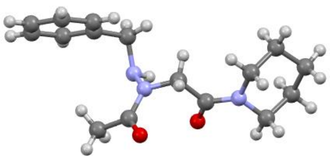

21a

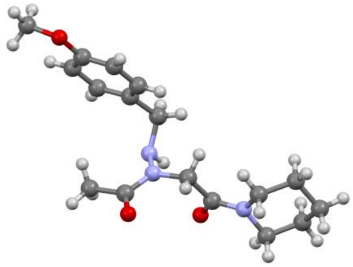

21b

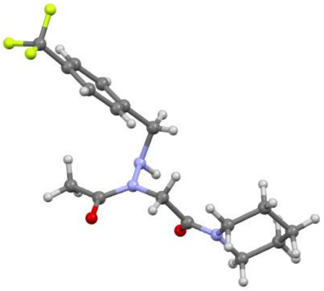

21c

Figure S34. X-ray crystal structures of peptoids **20a-c** and **21a-c**

Table S14. Dihedral angles of peptoids **20a-c** and **21a-c**

Structure	ω	φ	ψ	X
**20a**	–179.1	–102.7	–159.8	1.8
**21a**[a]	–167.4	77.9	–178.0	–69.5
**20b**	173.6	105.5	164.6	5.9
**21b**	–178.1	98.8	–174.3[b]	–65.3
**20c**	178.9	100.2	158.0	0.7
**21c**	–176.5	108.9	177.8	–65.9

[a]A second structure with opposite dihedral angles was present in the unit cell. [b]Disorder is present in the piperidinyl region. In the minor conformation, ψ = 150.6

Table S15. Hydrogen bond geometry (Å) for compounds **21a-c**

Structure	*D*-H…*A*	*D*-H	H…*A*	*D*…*A*	∠*D*-H…*A*
**21a**	N-H···O	0.88	2.66	3.10	112.4
**21b**	N-H···O	0.90	2.47	3.00	118.4
**21c**	N-H···O	0.90	2.35	2.93	122.0

Table S16. Crystal data and structure refinement of peptoids **20a-c** and **21a-c**

		**20a**	**21a**	**20b**	**21b**	**20c**	**21c**
**Empirical formula**		$C_{16}H_{21}N_3O_2$	$C_{16}H_{23}N_3O_2$	$C_{17}H_{23}N_3O_3$	$C_{17}H_{25}N_3O_3$	$C_{17}H_{20}F_3N_3O_2$	$C_{17}H_{22}F_3N_3O_2$
**Formula weight (g/mol)**		287.36	289.37	317.38	319.40	355.36	357.37
**Crystal size (mm^3)**		0.116 x 0.128 x 0.329	0.096 x 0.246 x 0.338	0.091 x 0.119 x 0.337	0.1664 x 0.198 x 0.319	0.033 x 0.056 x 0.228	0.110 x 0.138 x 0.154
**Crystal system**		monoclinic	monoclinic	Triclinic	Triclinic	Triclinic	Monoclinic
**Radiation**		Mo K_α (λ = 0.71073 Å)	Mo K_α (λ = 0.71073 Å)	CuKα, λ = 1.54178 Å	Cu Kα, λ = 1.54178 Å	Cu Kα, λ = 1.54178 Å	Cu Kα, λ = 1.54178 Å
**Temperature (K)**		100(2)	100(2)	120(2)	298(2)	100(2)	100(2)
**θ range (°)**		2.00 to 26.02	2.20 to 30.58	3.17 to 70.78	4.14 to 66.65	2.96 to 77.41	3.11 to 77.42
**Space group**		P 1 21/c 1	P 1 21 1	P -1	P -1	P -1	$P2_1/c$
**Unit cell dimensions**	***a* (Å)**	5.0586(8)	6.0726(3)	5.0802(2)	8.9857(2)	5.10660(10)	6.1398(2)
	***b* (Å)**	27.489(4)	9.9182(5)	11.6666(5)	10.0171(2)	10.9857(2)	9.9741(3)
	***c* (Å)**	10.9711(17)	26.1540(12)	14.3550(6)	11.6679(2)	15.6081(3)	28.5440(9)
	***α* (°)**	90	90	101.7036(13)	66.1920(10)	105.2980(10)	90
	***β* (°)**	90.664(4)	93.3620(15)	95.1179(12)	82.8390(10)	96.9290(10)	95.1500(10)
	***γ* (°)**	90	90	98.2498(11)	66.4290(10)	91.0110(10)	90
**Volume (Å^3)**		1525.5(4)	1572.53(13)	818.32(6)	879.95(3)	837.29(3)	1740.95(9)
**Z**		4	4	2	2	2	4
**Density (g/cm^3) (calc.)**		1.251	1.222	1.288	1.206	1.410	1.363
**F (000)**		616	624	340	344	372	752
**Reflections collected**		30759	27709	12430	23611	26360	40369
**Independent reflections**		3001 [R(int) = 0.0676]	9683 [R(int) = 0.0361]	2982 [R(int) = 0.0248]	3089 [R(int) = 0.0265]	3472 [R(int) = 0.0392]	3695 [R(int) = 0.0240]
**Completeness**		100.0%	99.9%	94.3%	99.5%	97.5	99.8%
**Data/restraints/parameters**		3001/0/191	9683/1/389	2982/0/210	3089/12/268	3472/0/227	3695/0/230
**Max. transmission**		0.9900	0.9920	0.9370	0.8970	0.9680	0.9020
**Minimum transmission**		0.9730	0.9730	0.7920	0.8130	0.8050	0.8670
**Goodness-of-fit on F^2**		1.186	1.026	1.046	1.076	1.043	1.042
**Final R indices [I > 2 σ (I)]**		R1 = 0.0754	R1 = 0.0419	R1 = 0.0414	R1 = 0.0691	R1 = 0.0368	R1 = 0.0374
		wR2 = 0.1741	wR2 = 0.0823	wR2 = 0.1061	wR2 = 0.2121	wR2 = 0.0938	wR2 = 0.0915
**Final R indices (all data)**		R1 = 0.0964	R1 = 0.0569	R1 = 0.0447	R1 = 0.0775	R1 = 0.0424	R1 = 0.0383
		wR2 = 0.1829	wR2 = 0.0882	wR2 = 0.1091	wR2 = 0.2250	wR2 = 0.0984	wR2 = 0.0923
**Largest diff. peak and hole (eÅ^{-3})**		0.342 and -0.359	0.328 and -0.196	0.178 and -0.267	0.456 and -0.231	0.294 and -0.279	0.472 and -0.435

Davern, C.M.; Lowe, B.D.; Rosfi, A.; Ison, E.A.; Proulx, C.

D. NMR Characterization Spectra

^{1}H NMR (600 MHz, $CDCl_3$)

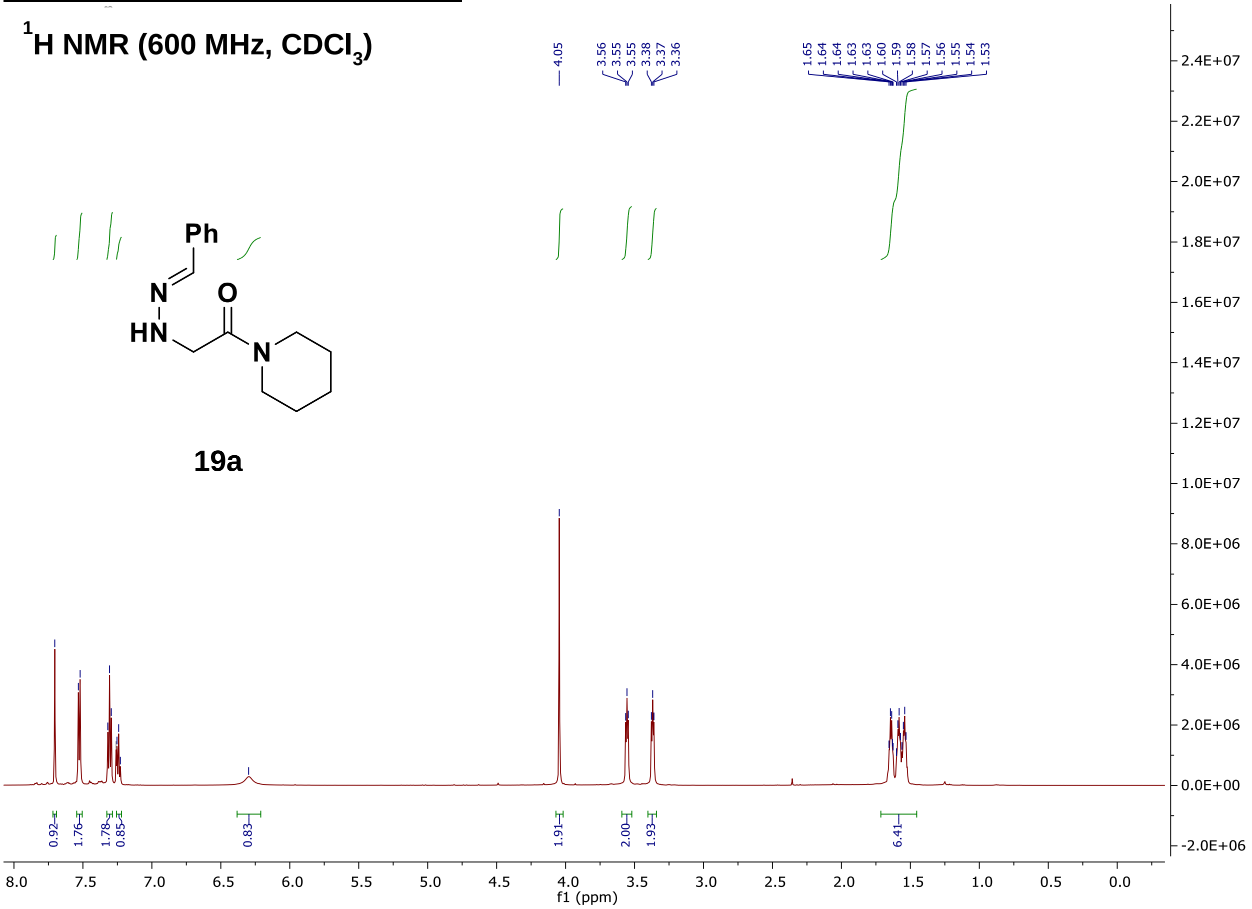

^{13}C NMR (150 MHz, $CDCl_3$)

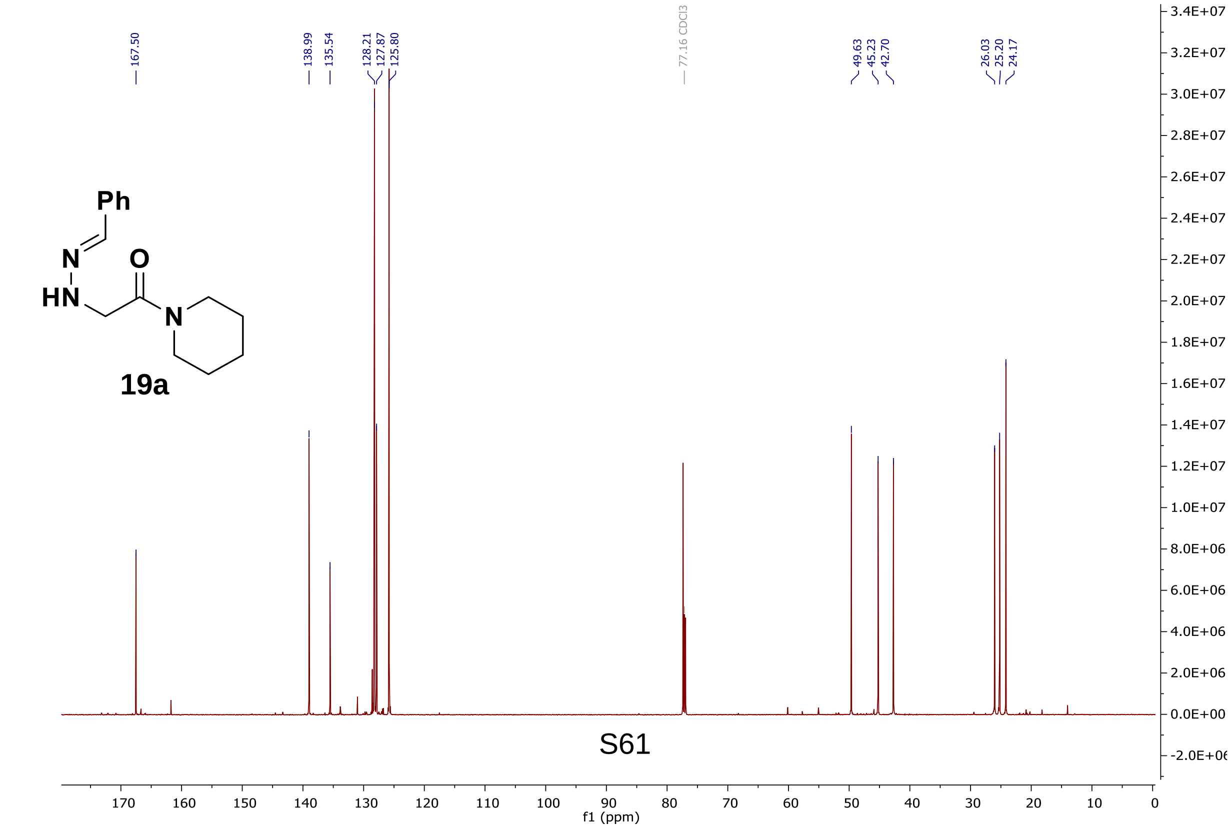

^{1}H NMR (600 MHz, $CDCl_3$)

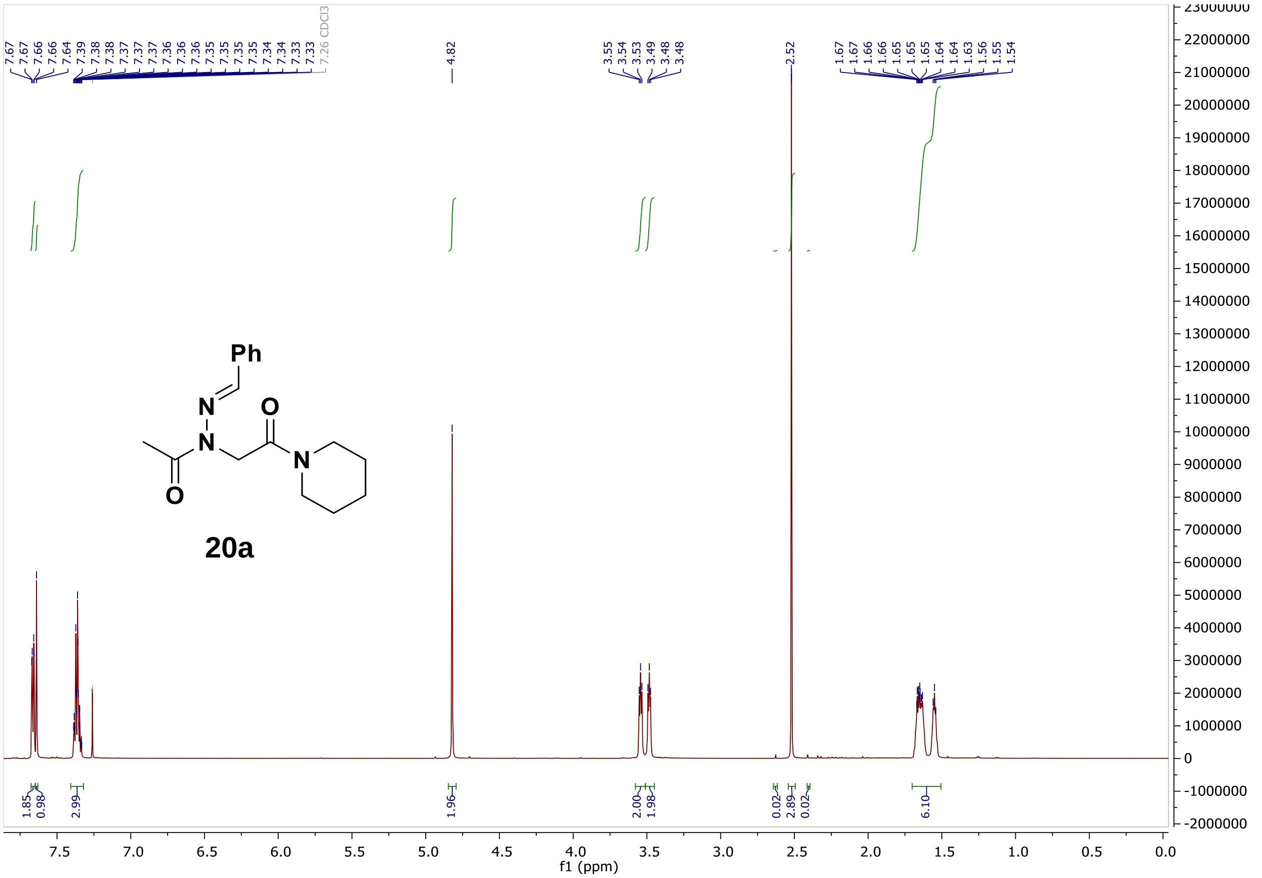

^{13}C NMR (150 MHz, $CDCl_3$)

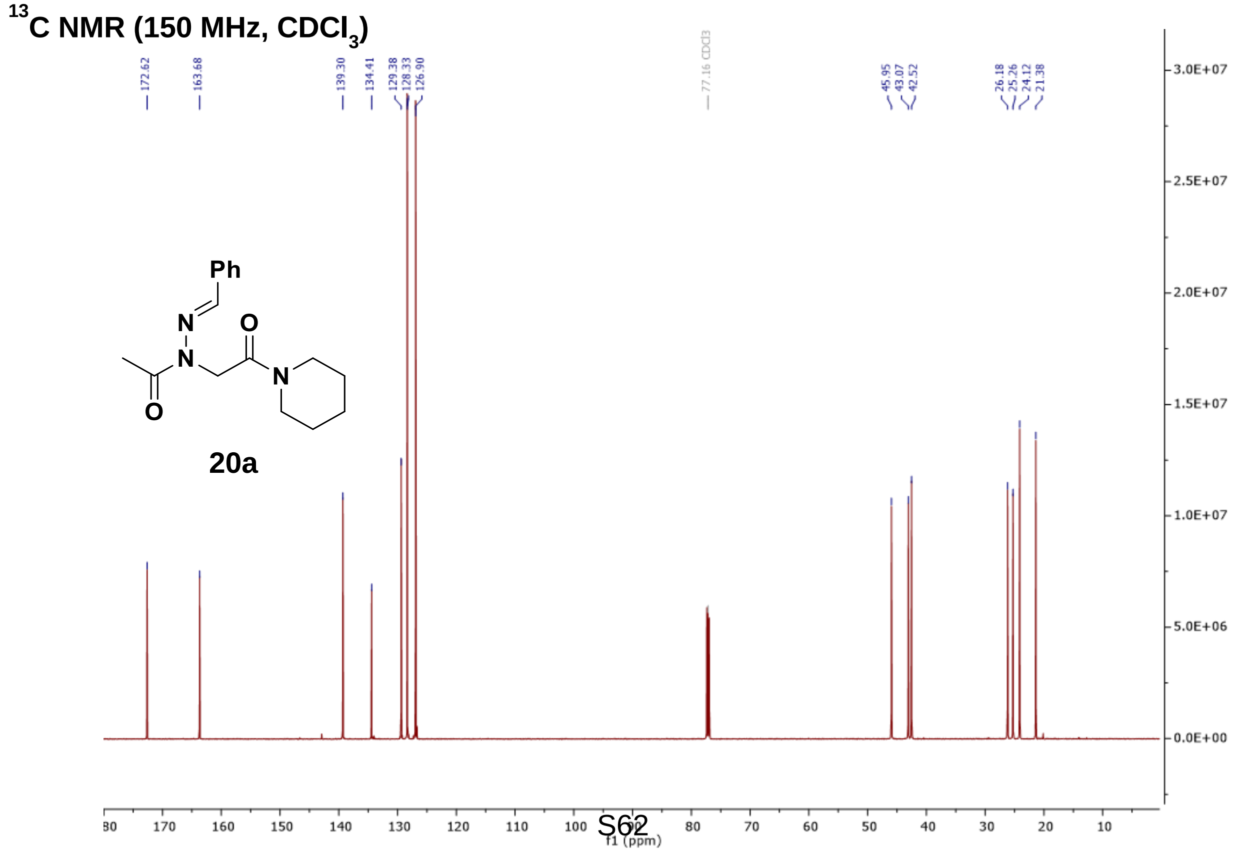

Davern, C.M.; Lowe, B.D.; Rosfi, A.; Ison, E.A.; Proulx, C.

^{1}H NMR (600 MHz, $CDCl_3$)

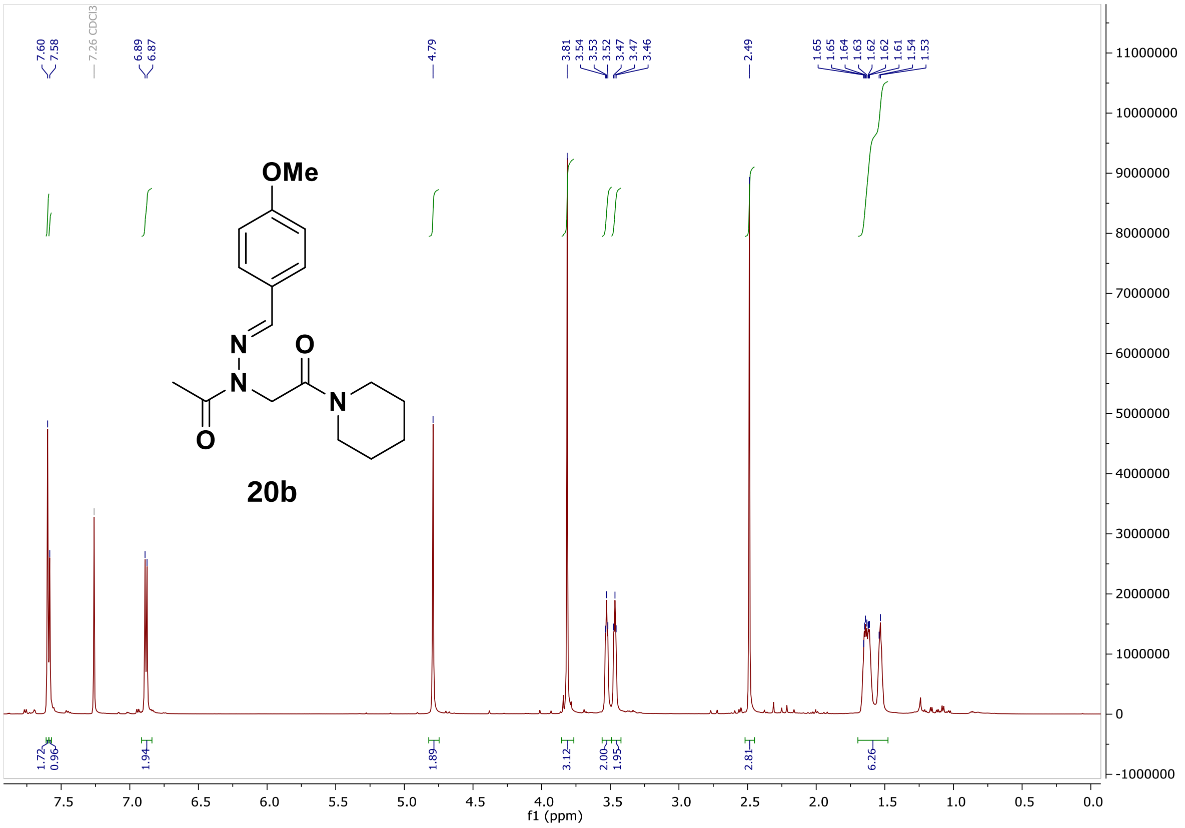

^{13}C NMR (125 MHz, $CDCl_3$)

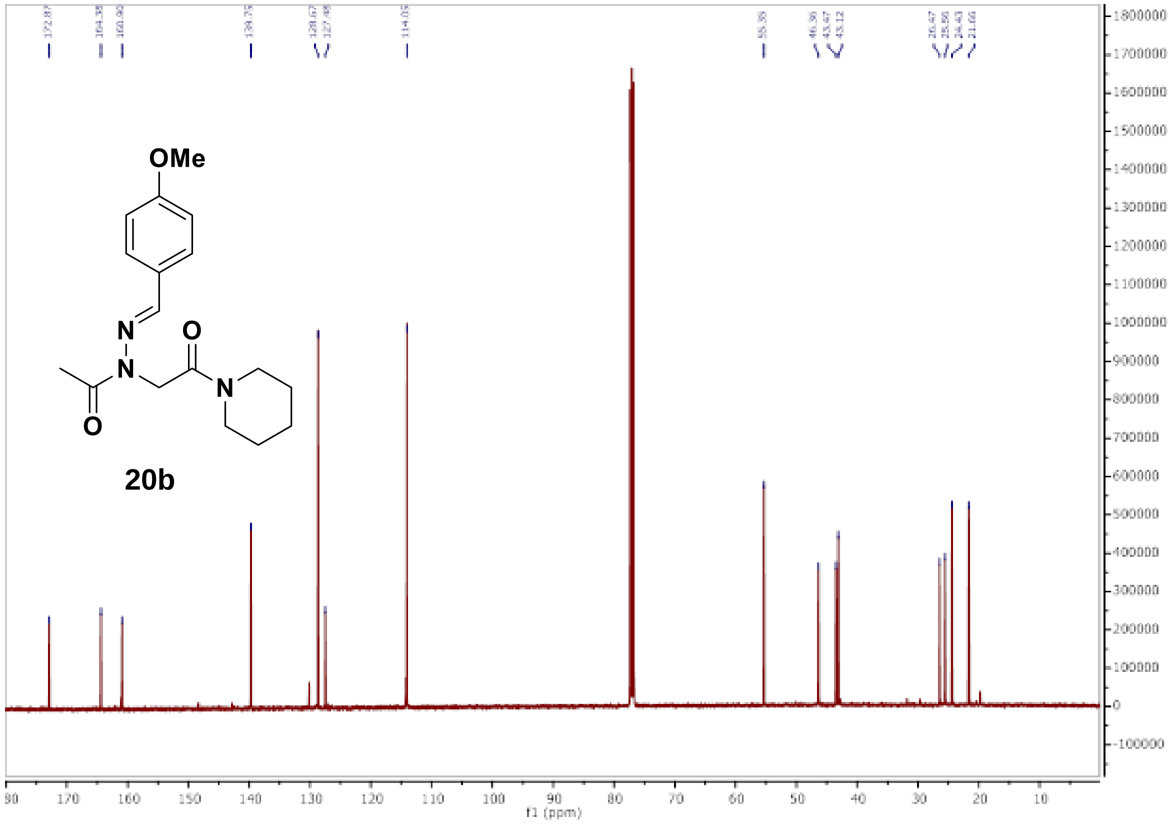

Davern, C.M.; Lowe, B.D.; Rosfi, A.; Ison, E.A.; Proulx, C.

^{1}H NMR (600 MHz, $CDCl_3$)

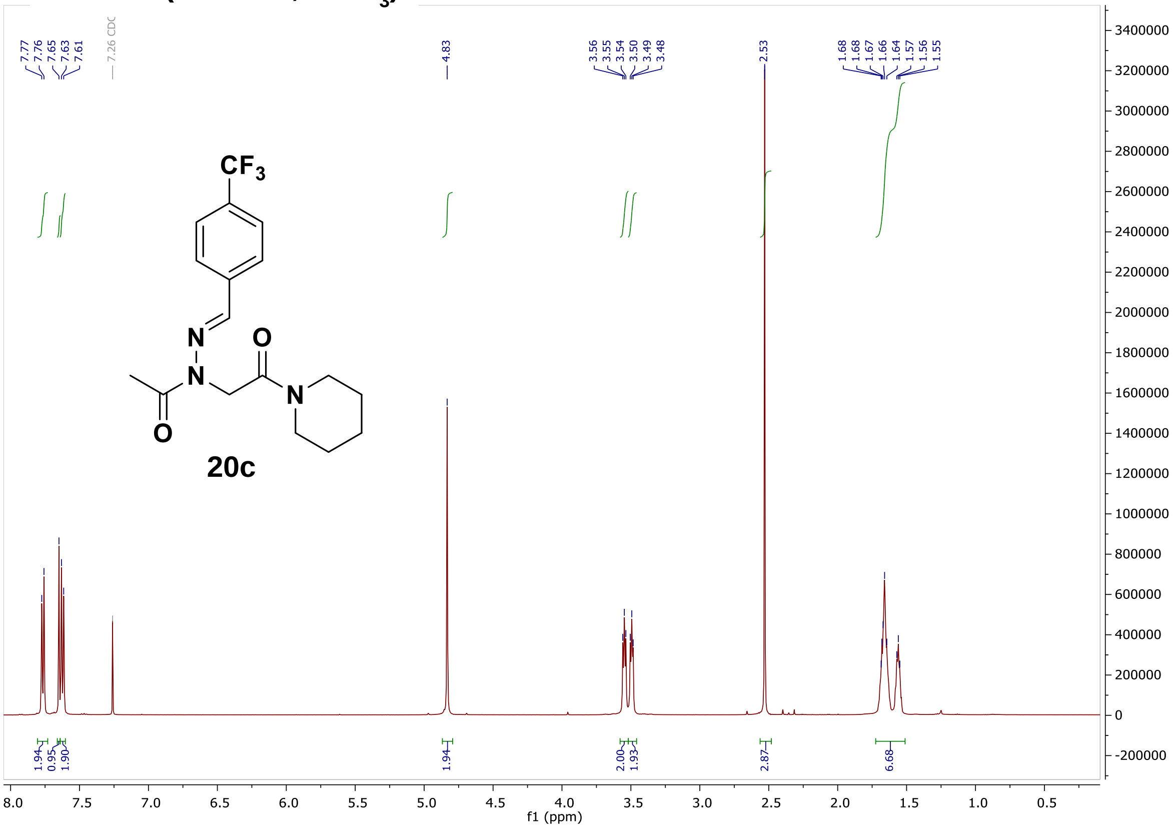

^{13}C NMR (150 MHz, $CDCl_3$)

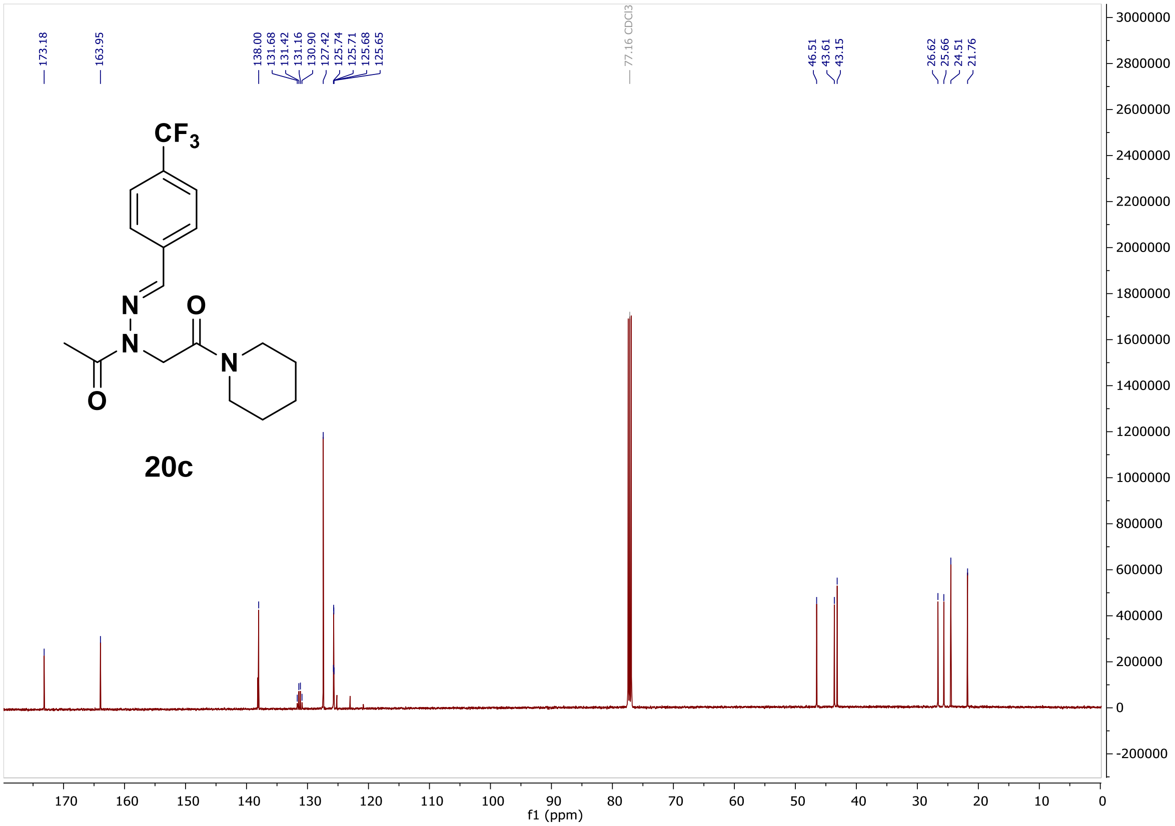

Davern, C.M.; Lowe, B.D.; Rosfi, A.; Ison, E.A.; Proulx, C.

^{1}H NMR (600 MHz, $CDCl_3$)

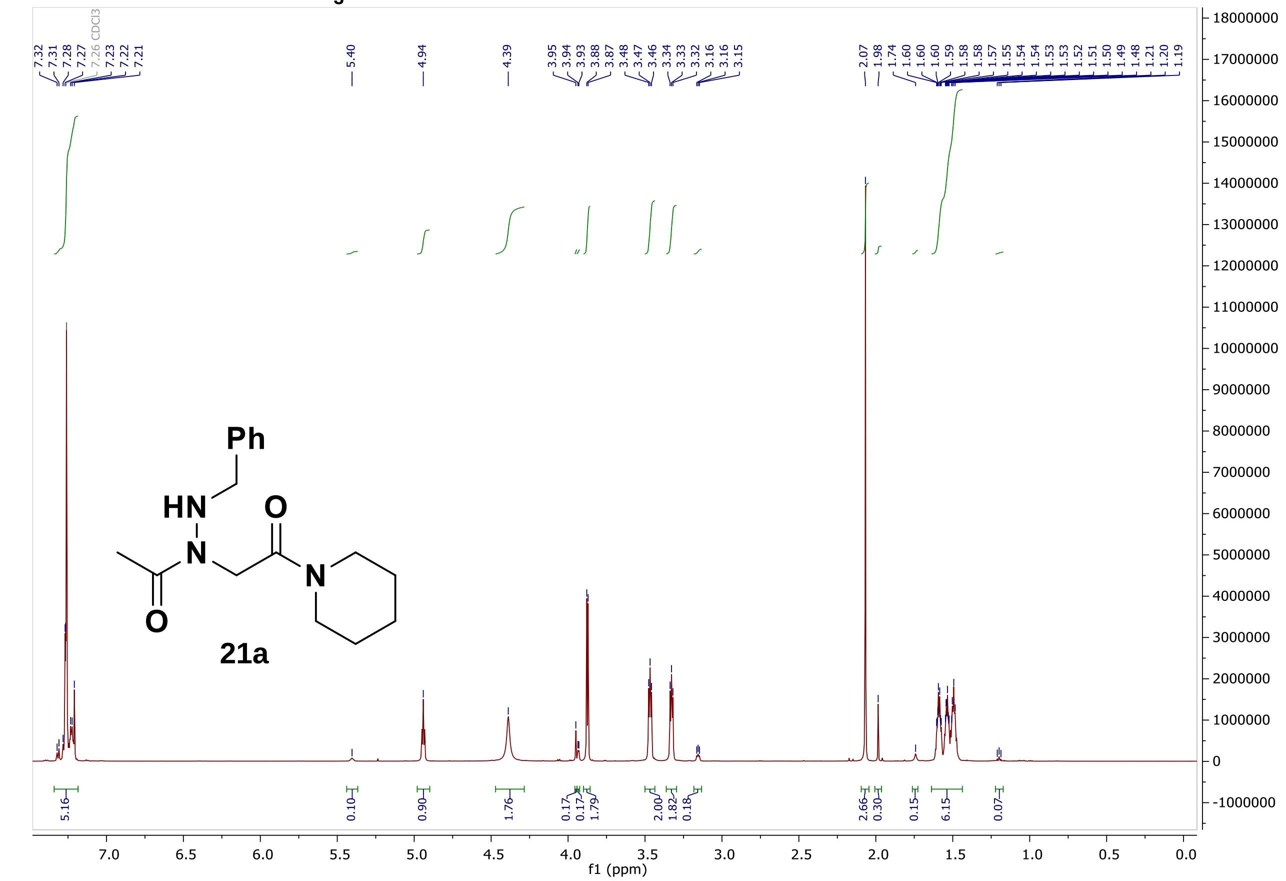

^{13}C NMR (150 MHz, $CDCl_3$)

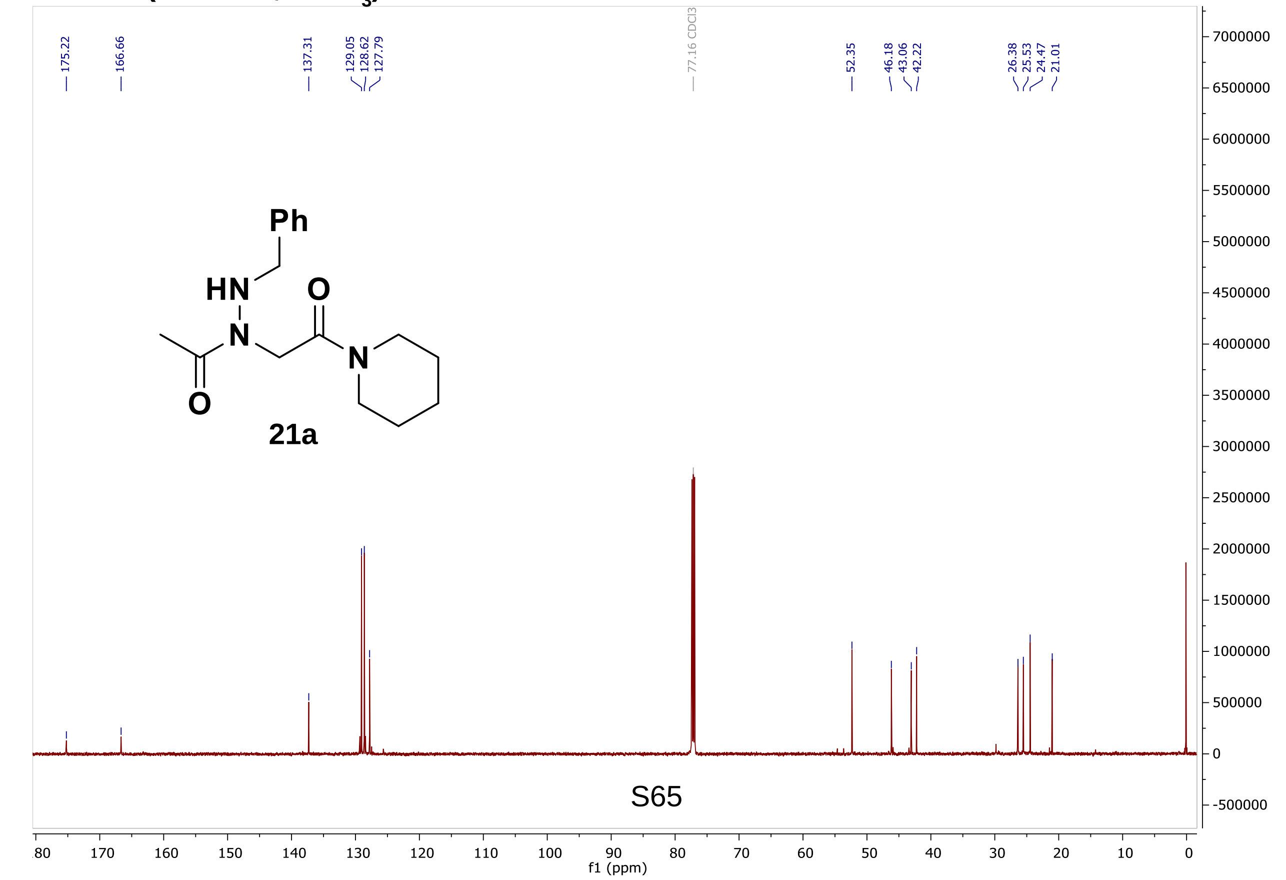

^{1}H NMR (600 MHz, $CDCl_3$)

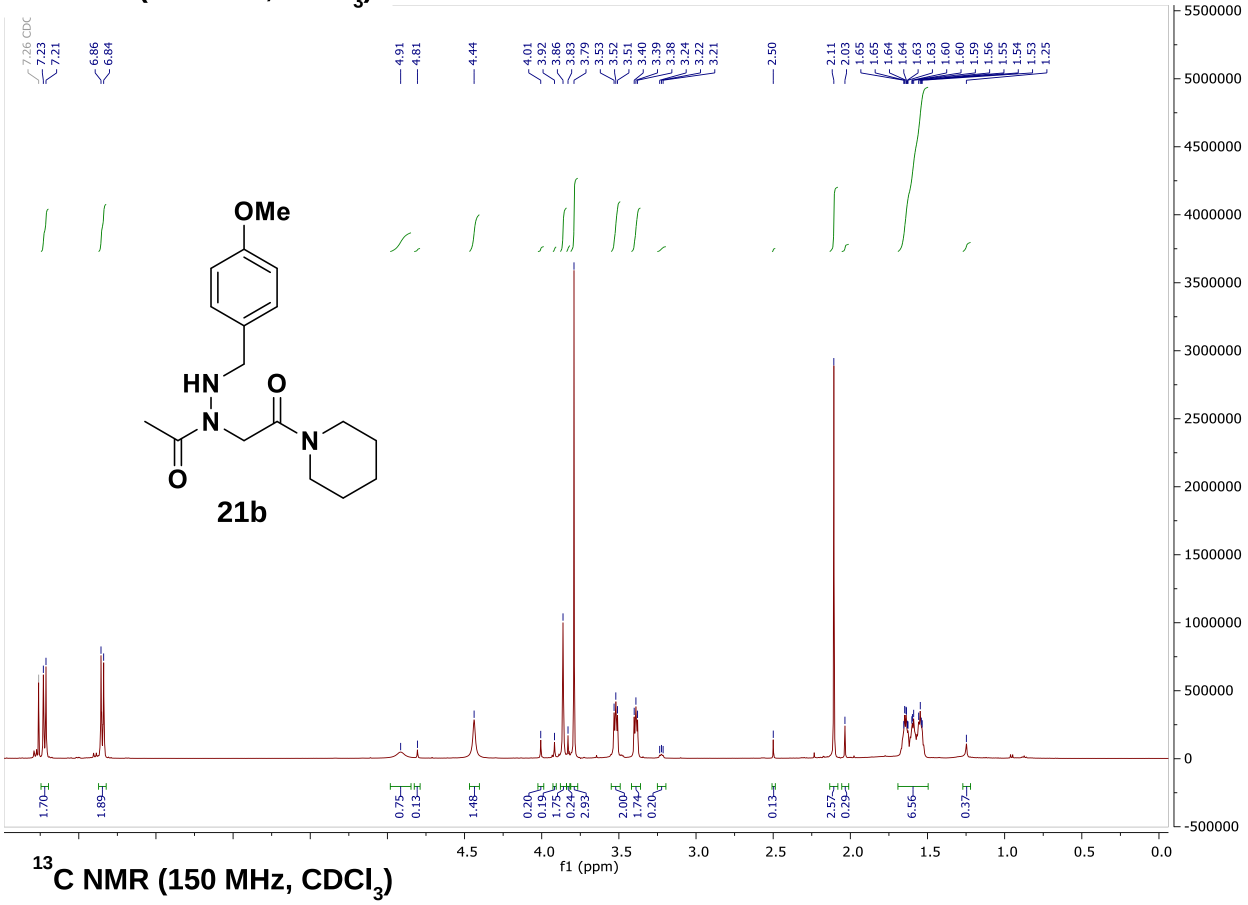

^{13}C NMR (150 MHz, $CDCl_3$)

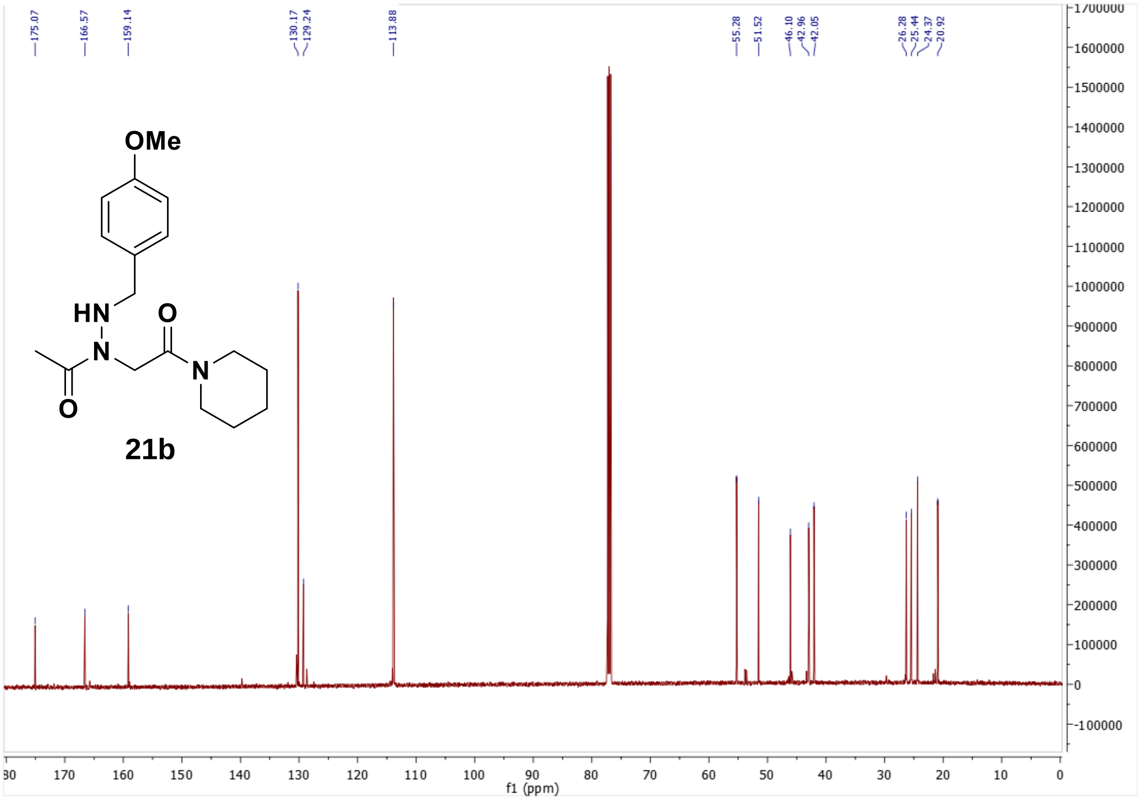

Davern, C.M.; Lowe, B.D.; Rosfi, A.; Ison, E.A.; Proulx, C.

^{1}H NMR (600 MHz, $CDCl_3$)

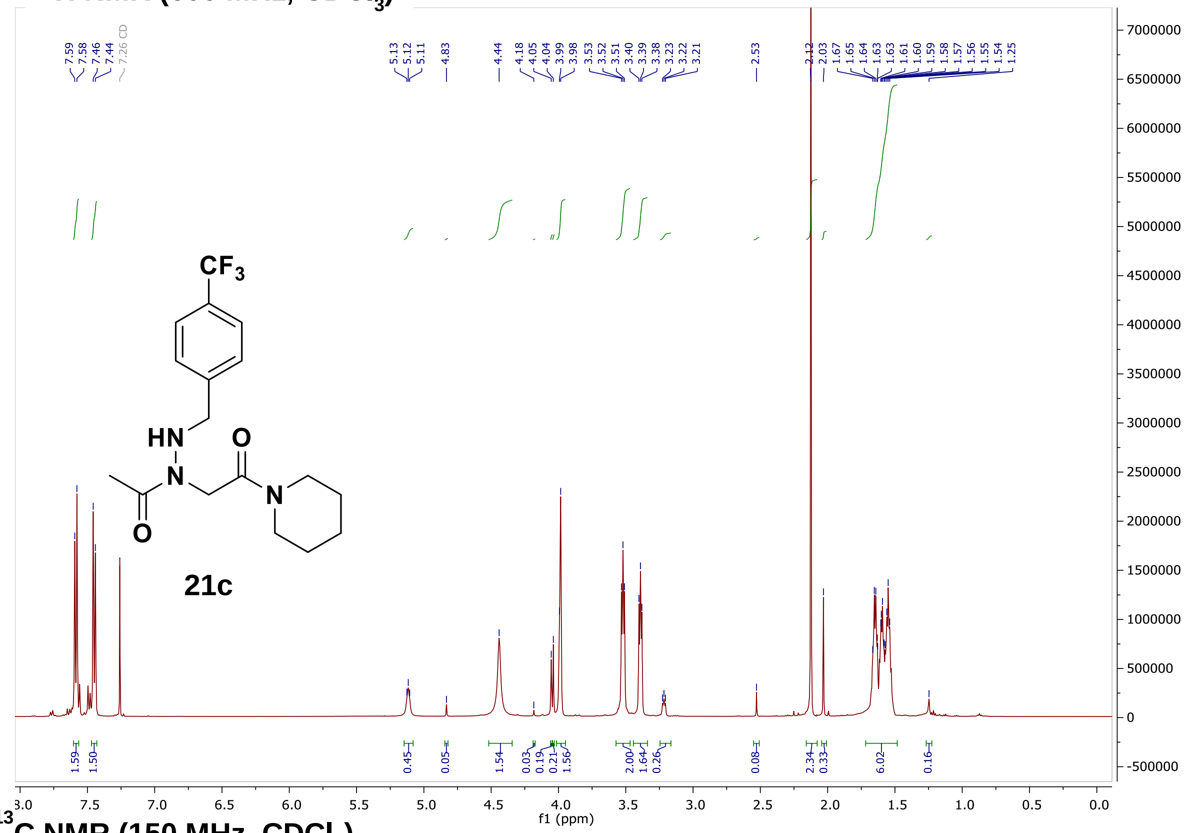

^{13}C NMR (150 MHz, $CDCl_3$)

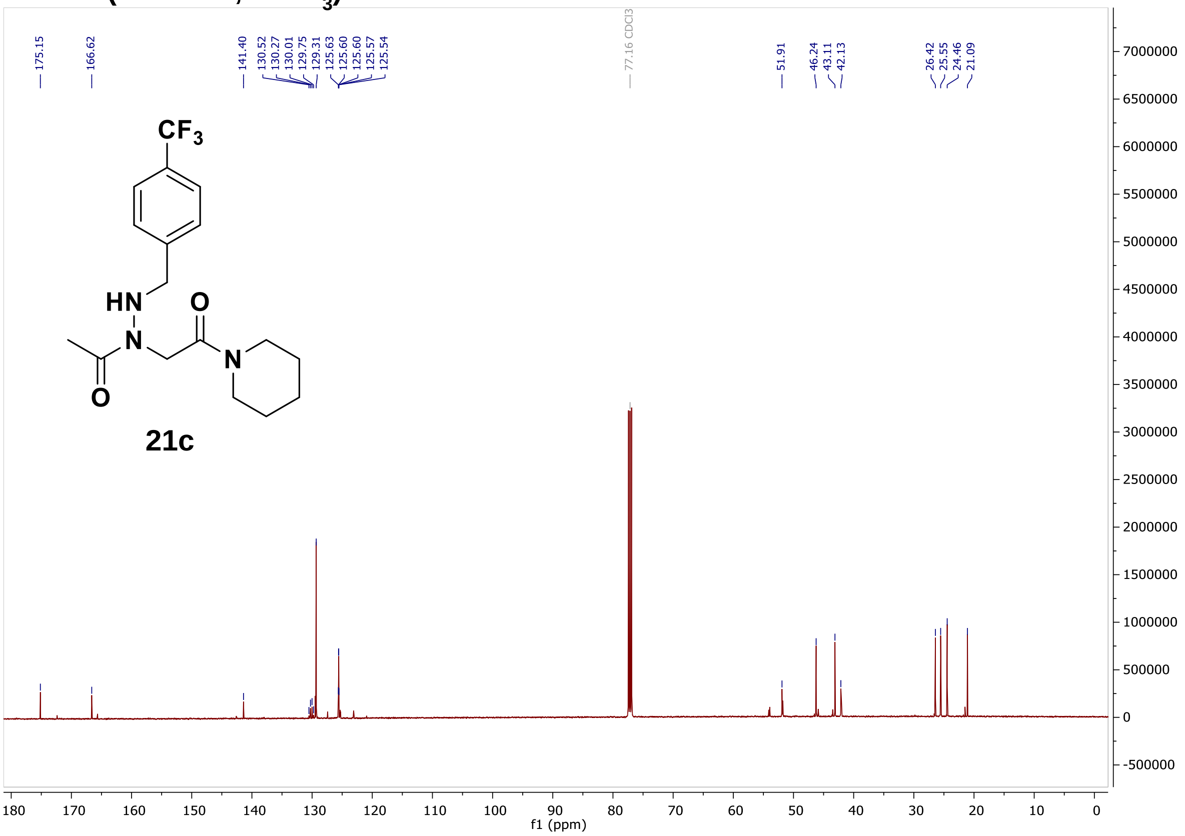

^{1}H NMR (600 MHz, $CDCl_3$)

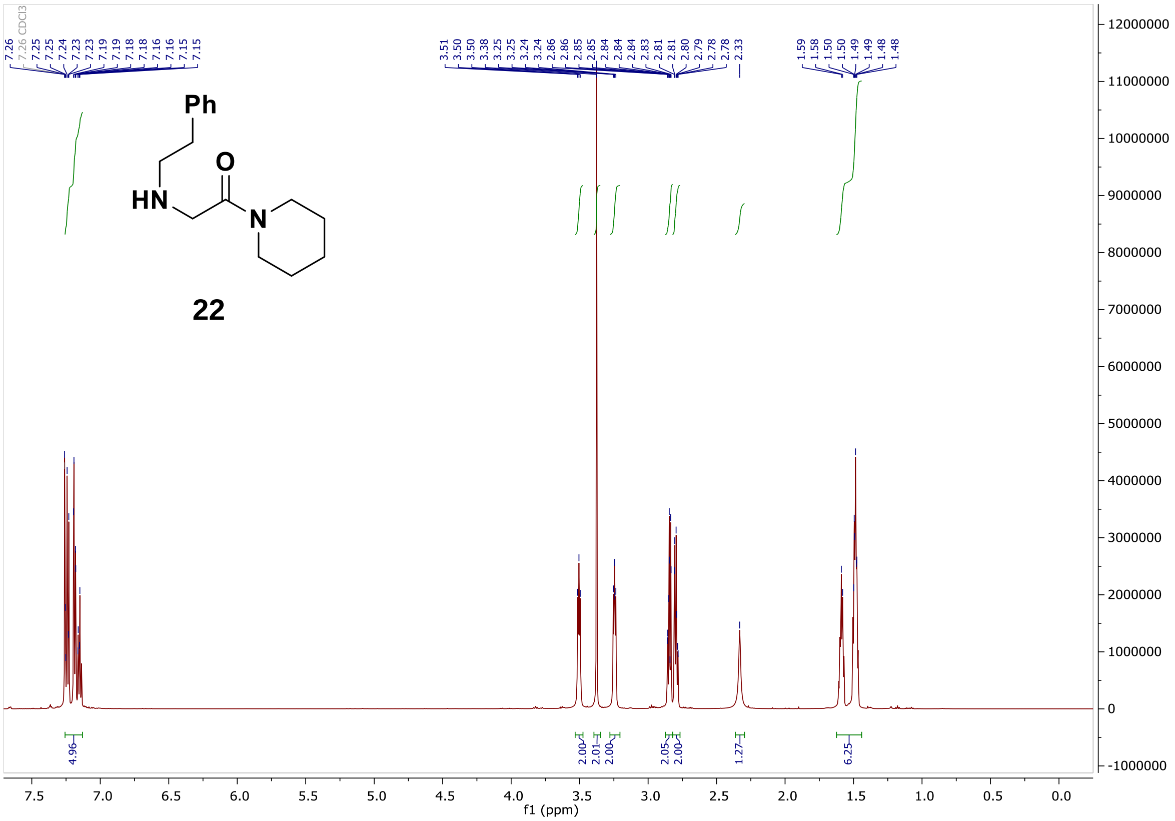

^{13}C NMR (150 MHz, $CDCl_3$)

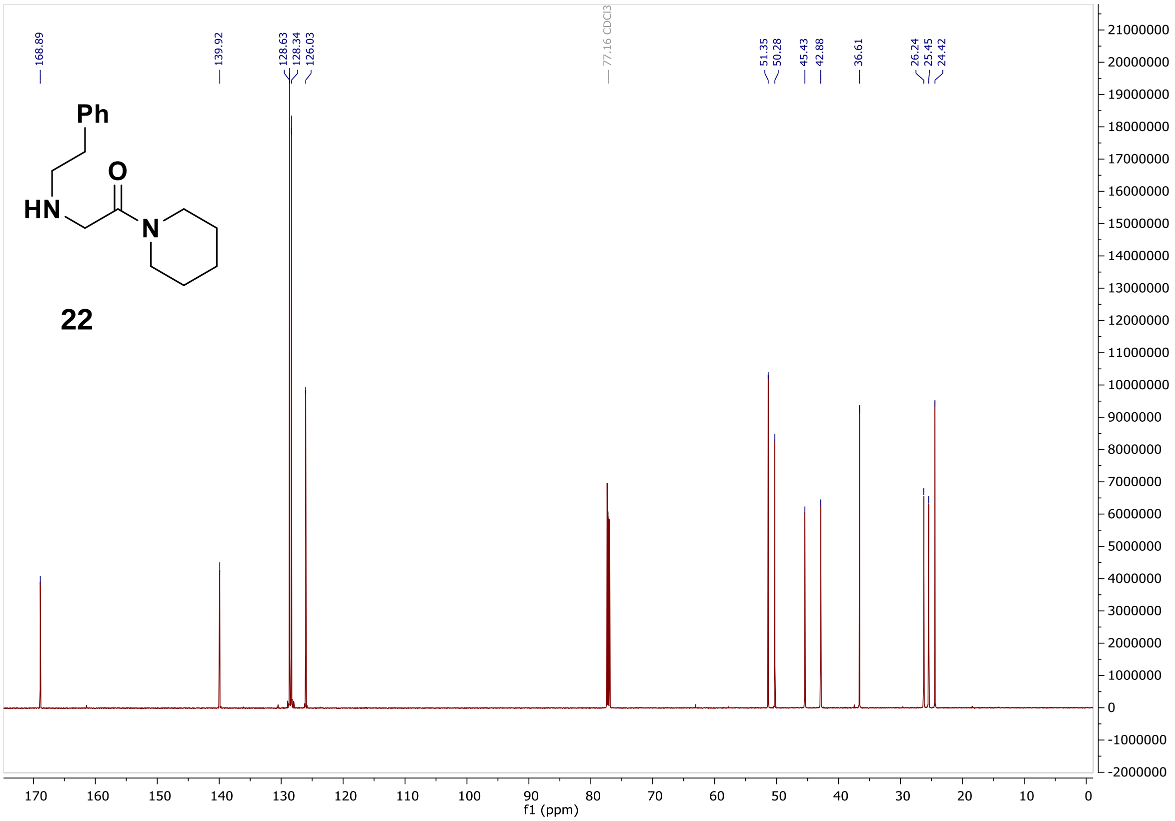

^{1}H NMR (600 MHz, $CDCl_3$)

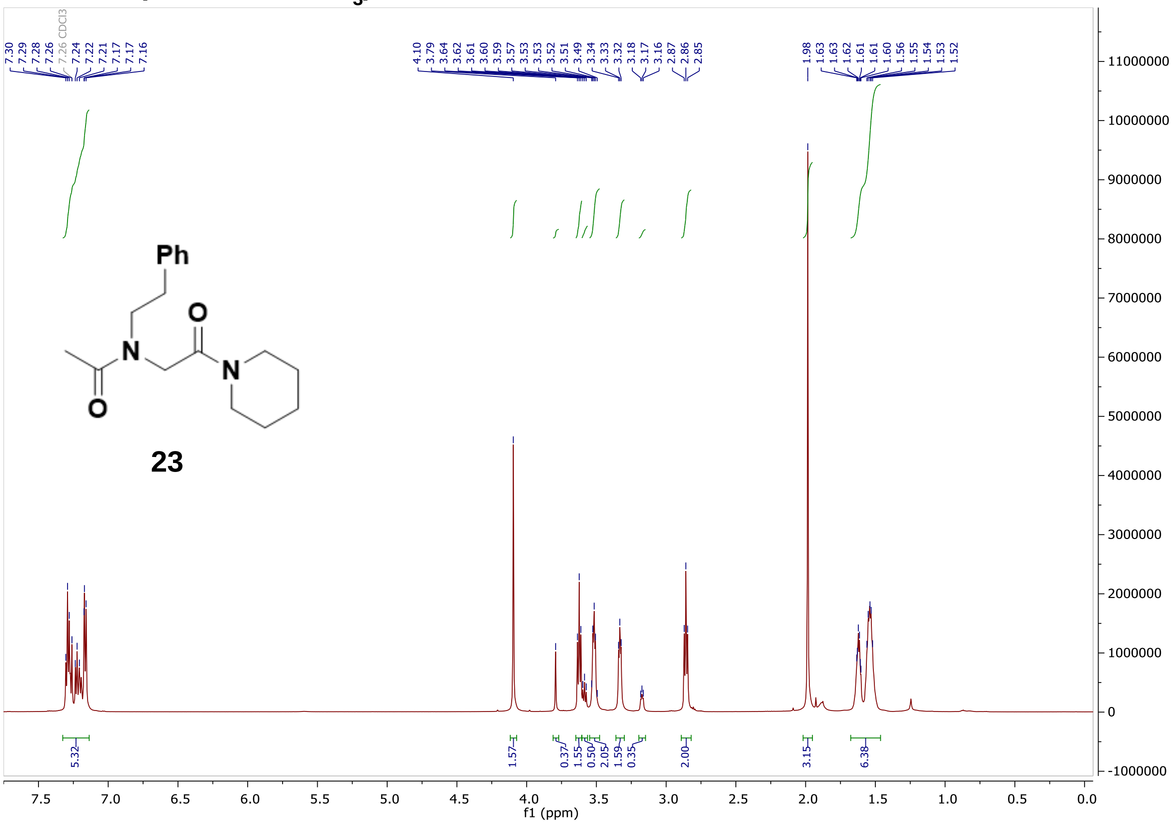

^{13}C NMR (150 MHz, $CDCl_3$)

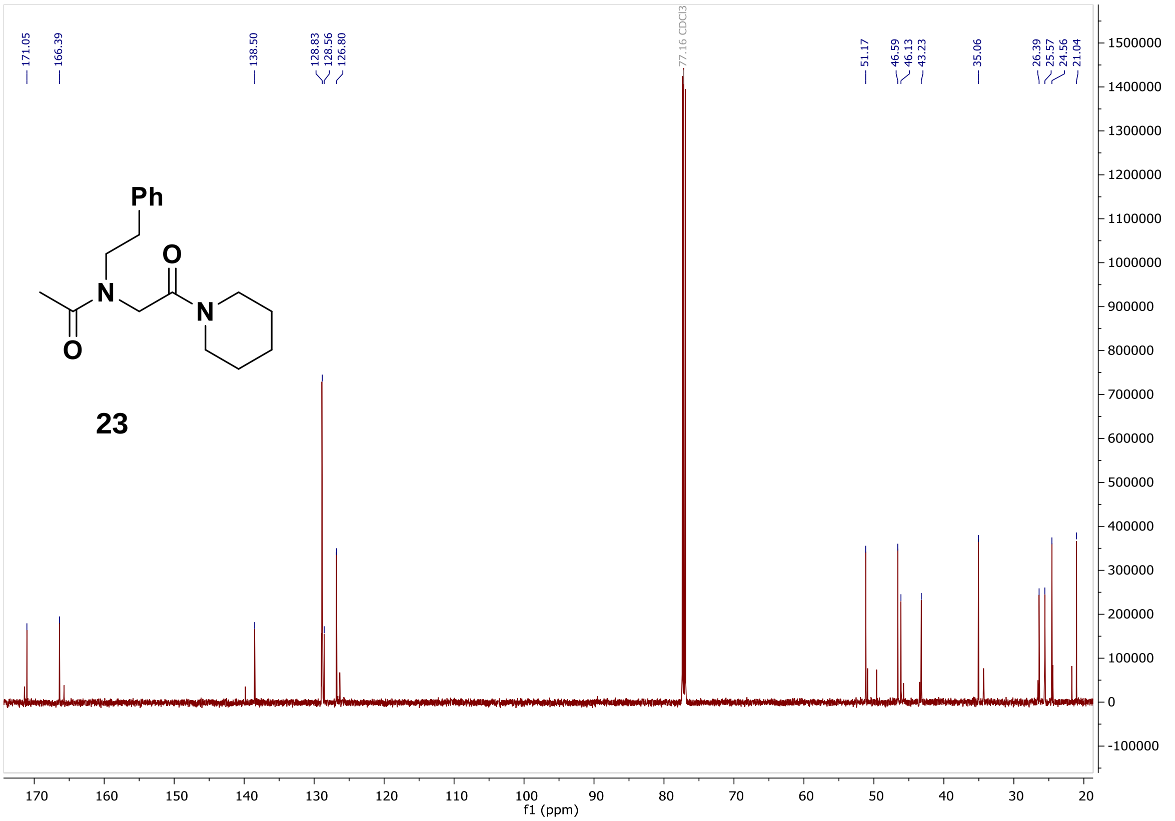

E. LCMS Chromatograms

Characterization for crude peptoid pentamers

Cleavages done with Cleavage I: 95:5 TFA:H_2O for ten minutes and all traces on a 30-90 gradient unless otherwise noted. Traces correspond to data in **Scheme 1** and **Table S4.**

4a

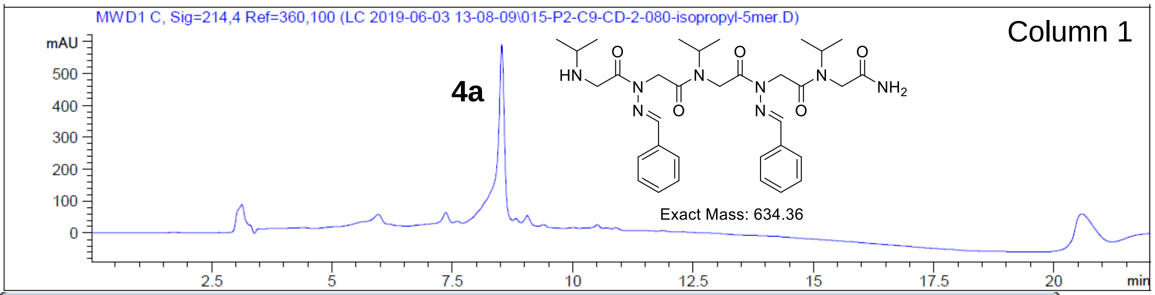

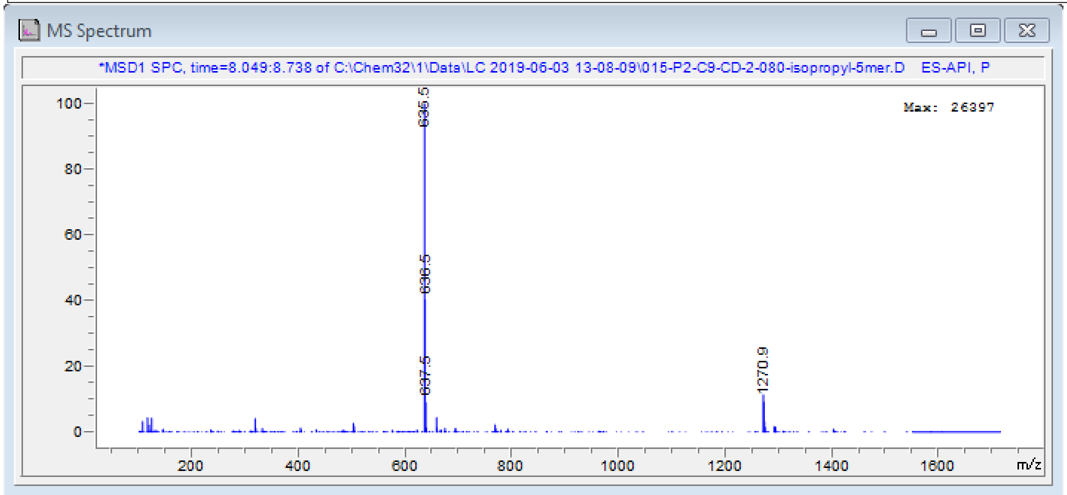

4b

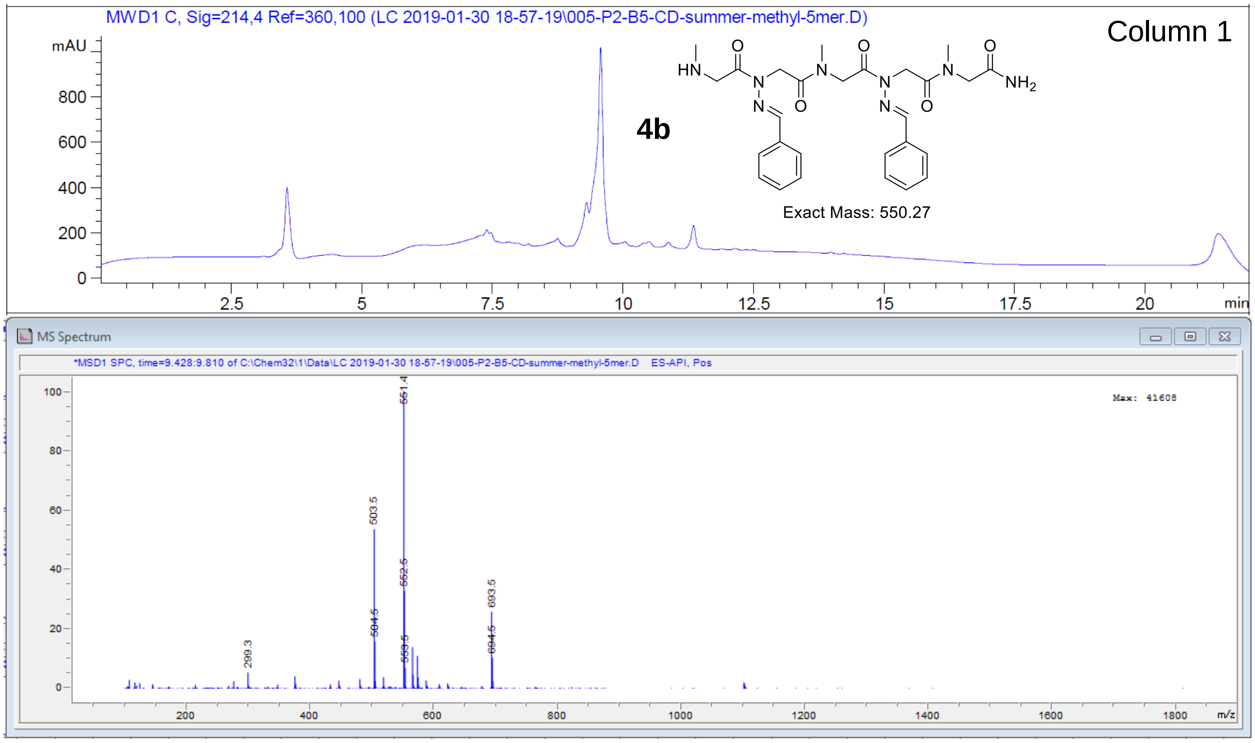
MWD1 C, Sig=214,4 Ref=360,100 (LC 2019-01-30 18-57-19\005-P2-B5-CD-summer-methyl-5mer.D)
Column 1
mAU
800
600
400
200
0
2.5
5
7.5
10
12.5
15
17.5
20
min
4b
HN
O
N
N
O
N
O
N
N
O
N
O
NH2
Exact Mass: 550.27
MS Spectrum
*MSD1 SPC, time=9.428:9.810 of C:\Chem32\1\Data\LC 2019-01-30 18-57-19\005-P2-B5-CD-summer-methyl-5mer.D ES-API, Pos
Max: 41608
100
80
60
40
20
0
551.4
503.5
552.5
693.5
504.5
553.5
694.5
299.3
200
400
600
800
1000
1200
1400
1600
1800
m/z

4c

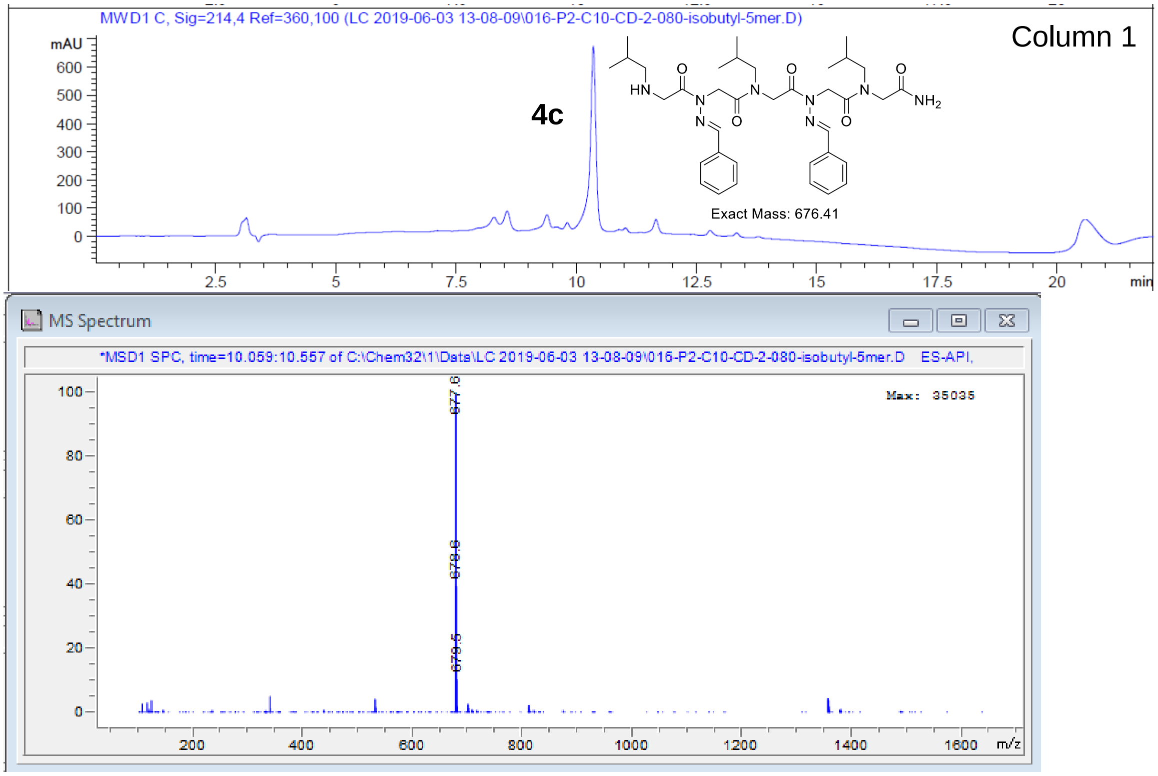
MWD1 C, Sig=214,4 Ref=360,100 (LC 2019-06-03 13-08-09\016-P2-C10-CD-2-080-isobutyl-5mer.D)
Column 1
mAU
600
500
400
300
200
100
0
2.5
5
7.5
10
12.5
15
17.5
20
min
4c
HN
O
N
N
O
N
O
N
N
O
N
O
NH2
Exact Mass: 676.41
MS Spectrum
*MSD1 SPC, time=10.059:10.557 of C:\Chem32\1\Data\LC 2019-06-03 13-08-09\016-P2-C10-CD-2-080-isobutyl-5mer.D ES-API,
Max: 35035
100
80
60
40
20
0
677.6
678.6
679.5
200
400
600
800
1000
1200
1400
1600
m/z

4d

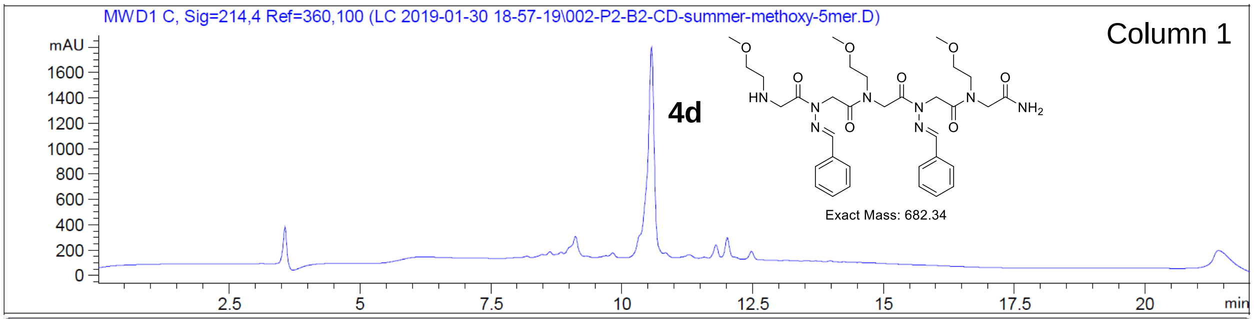
MWD1 C, Sig=214,4 Ref=360,100 (LC 2019-01-30 18-57-19\002-P2-B2-CD-summer-methoxy-5mer.D)
Column 1
4d
Exact Mass: 682.34
mAU
min

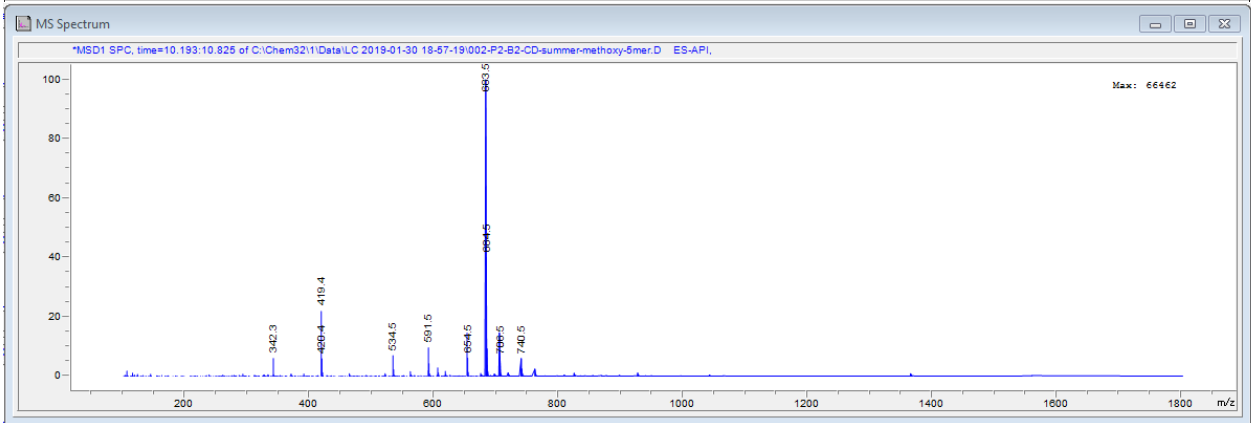
MS Spectrum
*MSD1 SPC, time=10.193:10.825 of C:\Chem32\1\Data\LC 2019-01-30 18-57-19\002-P2-B2-CD-summer-methoxy-5mer.D ES-API,
Max: 66462
683.5
684.5
419.4
420.4
342.3
534.5
591.5
654.5
706.5
740.5
m/z

4e

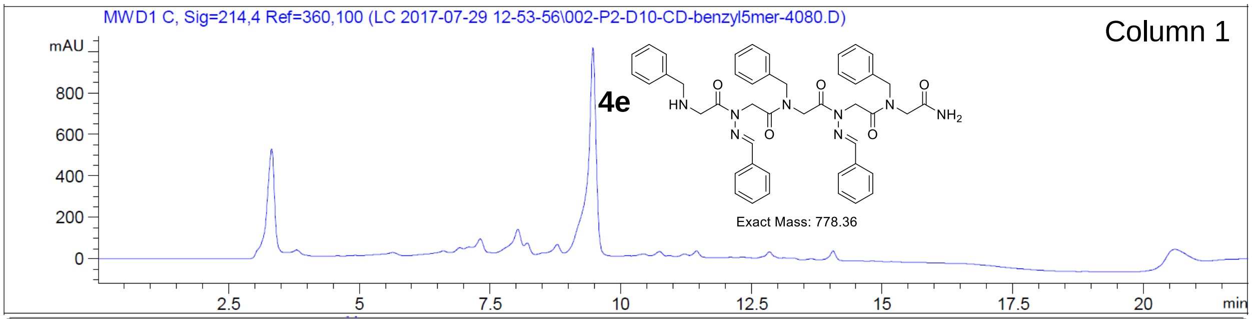
MWD1 C, Sig=214,4 Ref=360,100 (LC 2017-07-29 12-53-56\002-P2-D10-CD-benzyl5mer-4080.D)
Column 1
4e
Exact Mass: 778.36
mAU
min

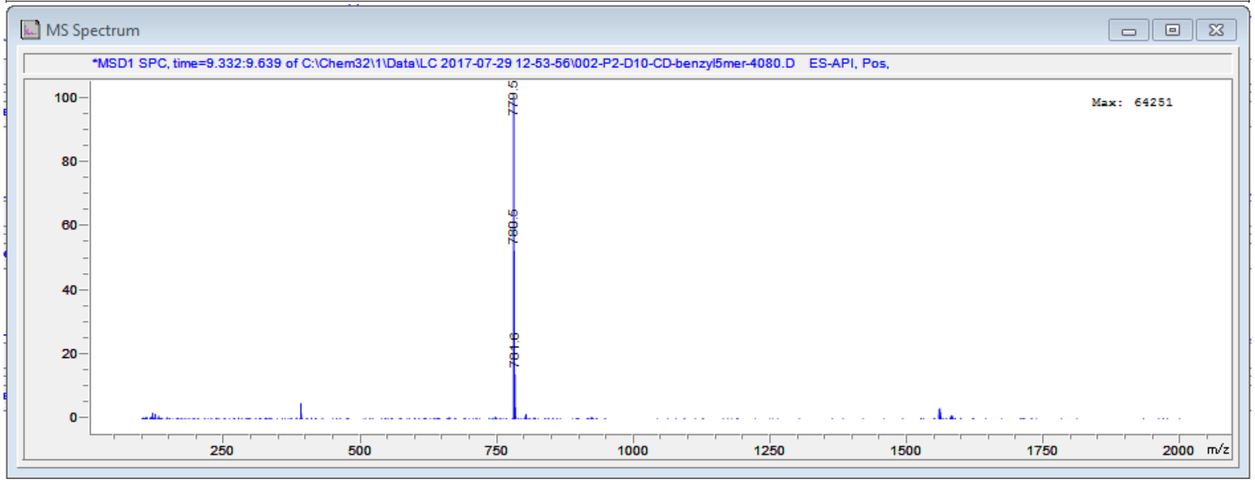
MS Spectrum
*MSD1 SPC, time=9.332:9.639 of C:\Chem32\1\Data\LC 2017-07-29 12-53-56\002-P2-D10-CD-benzyl5mer-4080.D ES-API, Pos,
Max: 64251
779.5
780.5
781.6
m/z

4f

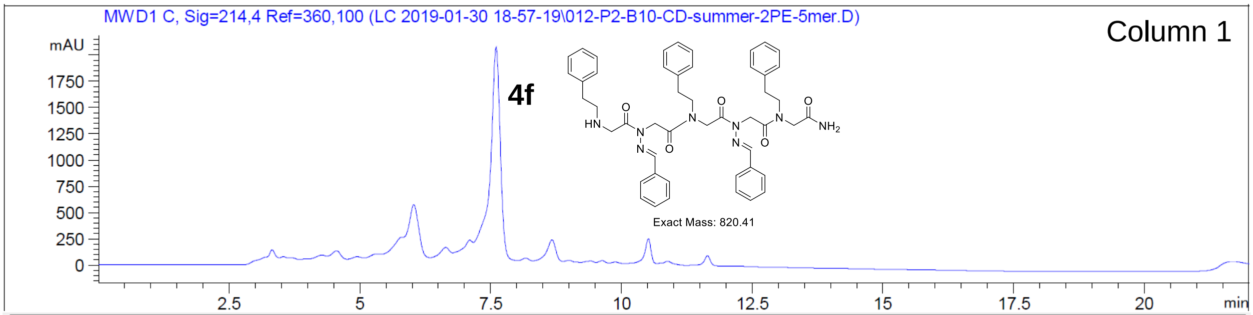

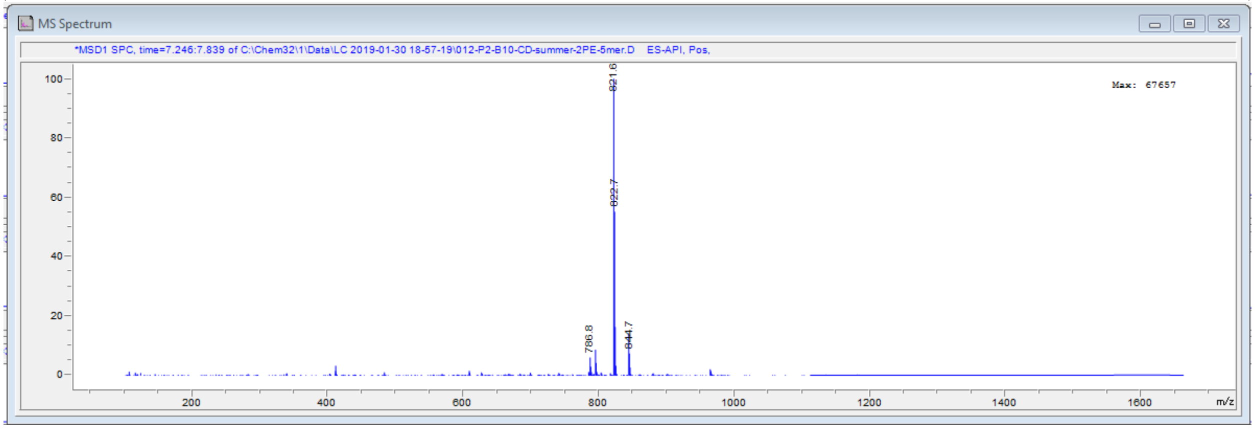

4g

5mer cleaved with Cleavage IV: 95:5 TFA:water 2h (special cleavage for β-alanine residues)

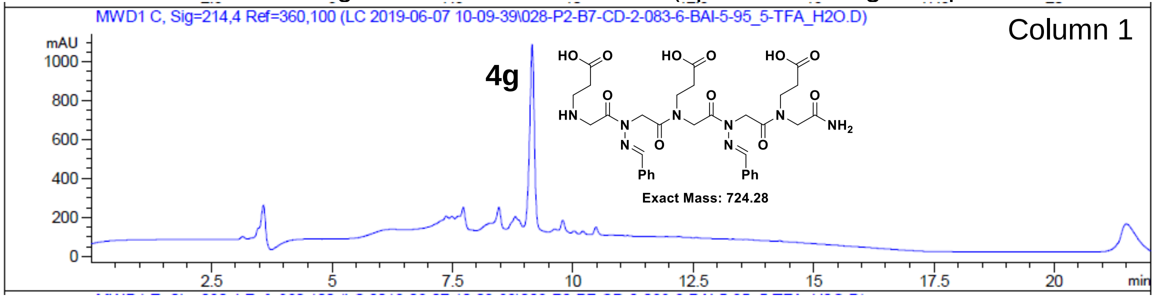

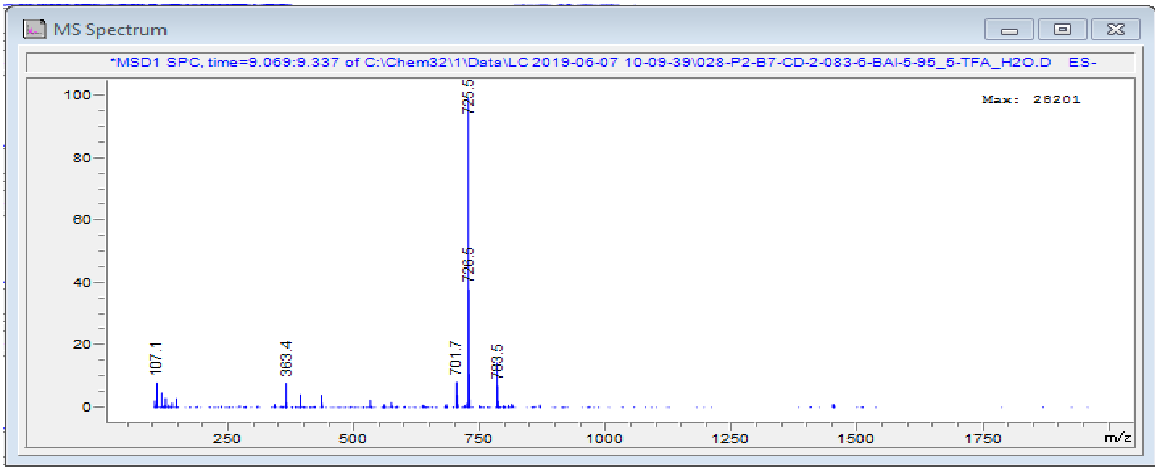

4h Aniline

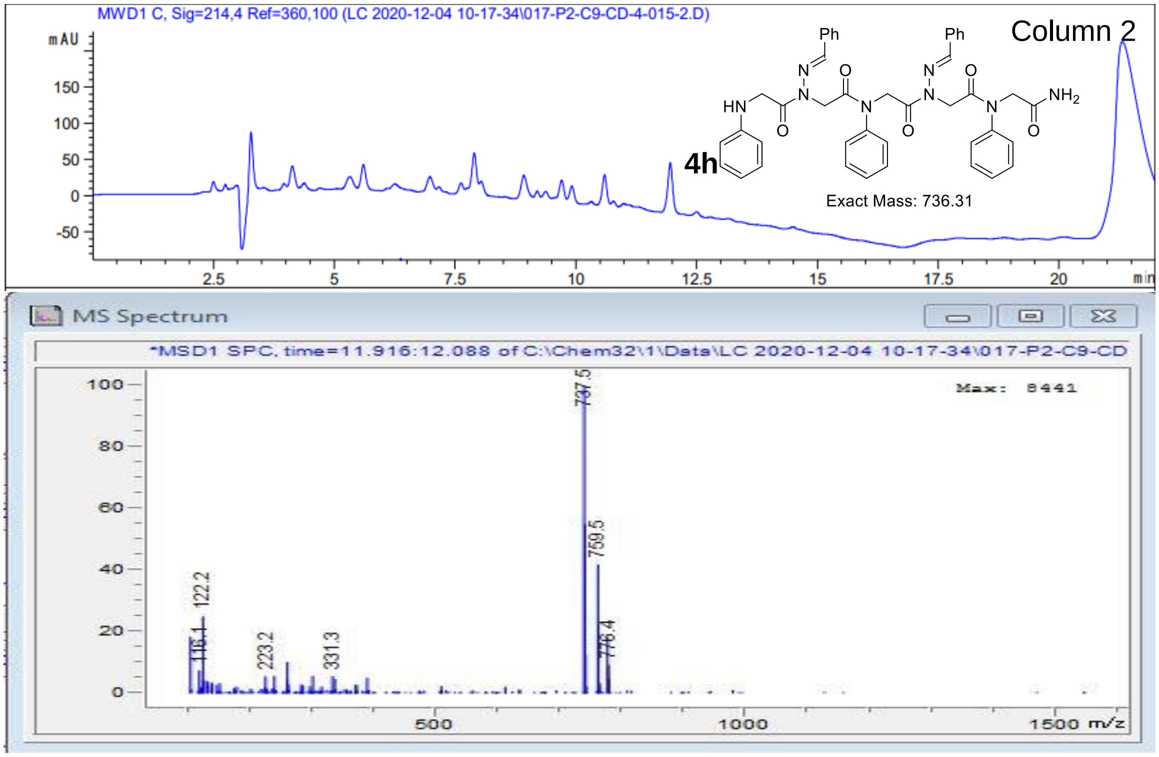
MWD1 C, Sig=214,4 Ref=360,100 (LC 2020-12-04 10-17-34\017-P2-C9-CD-4-015-2.D)
mAU
Column 2
4h
Exact Mass: 736.31
min
MS Spectrum
*MSD1 SPC, time=11.916:12.088 of C:\Chem32\1\Data\LC 2020-12-04 10-17-34\017-P2-C9-CD
Max: 8441
737.5
759.5
776.4
122.2
116.1
223.2
331.3
m/z

Characterization for 5 **with various cleavage conditions.**
All traces on a 30-90 gradient unless otherwise noted. Traces correspond to data in **Table 2**.

Cleavage I: 95:2.5:2.5 TFA:TIPS:H_2O 10 min * 50-95 gradient

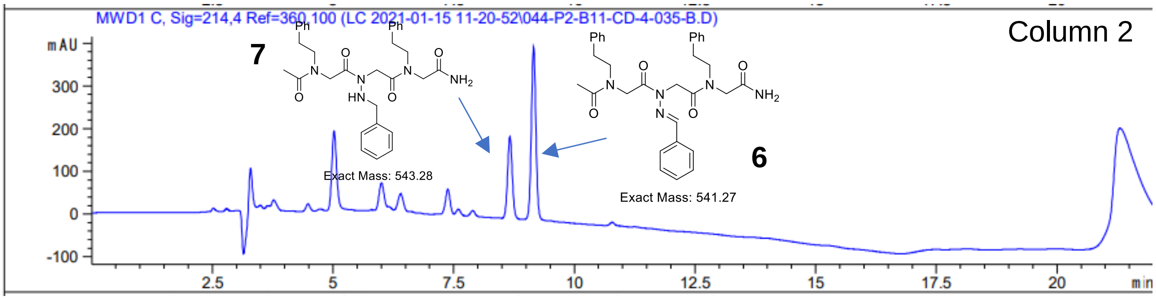

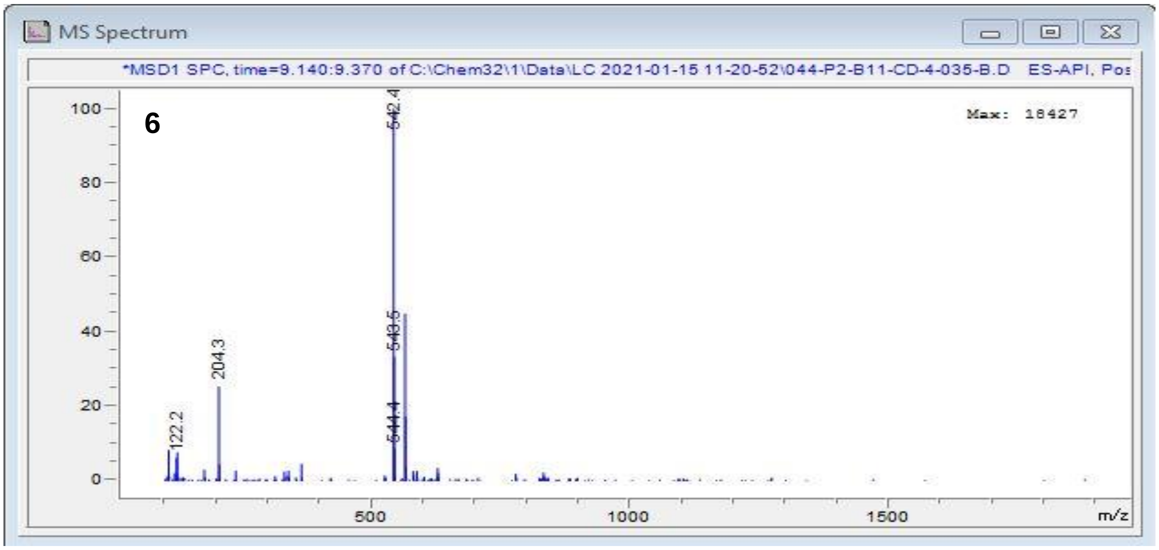

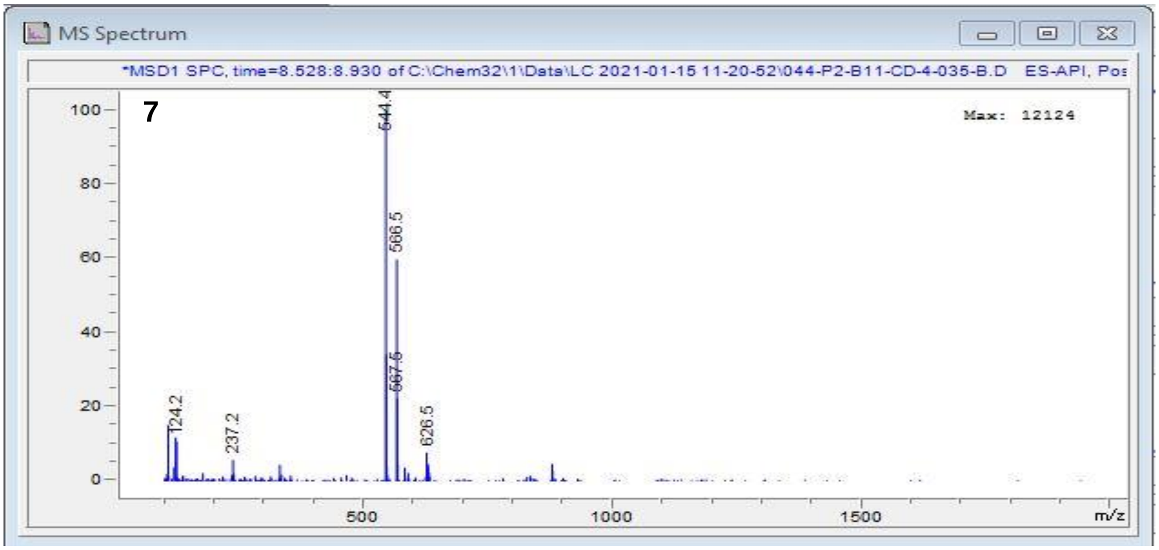

Cleavage II: 95:2.5:2.5 TFA:TIPS:H_2O 2h * 50-95 gradient

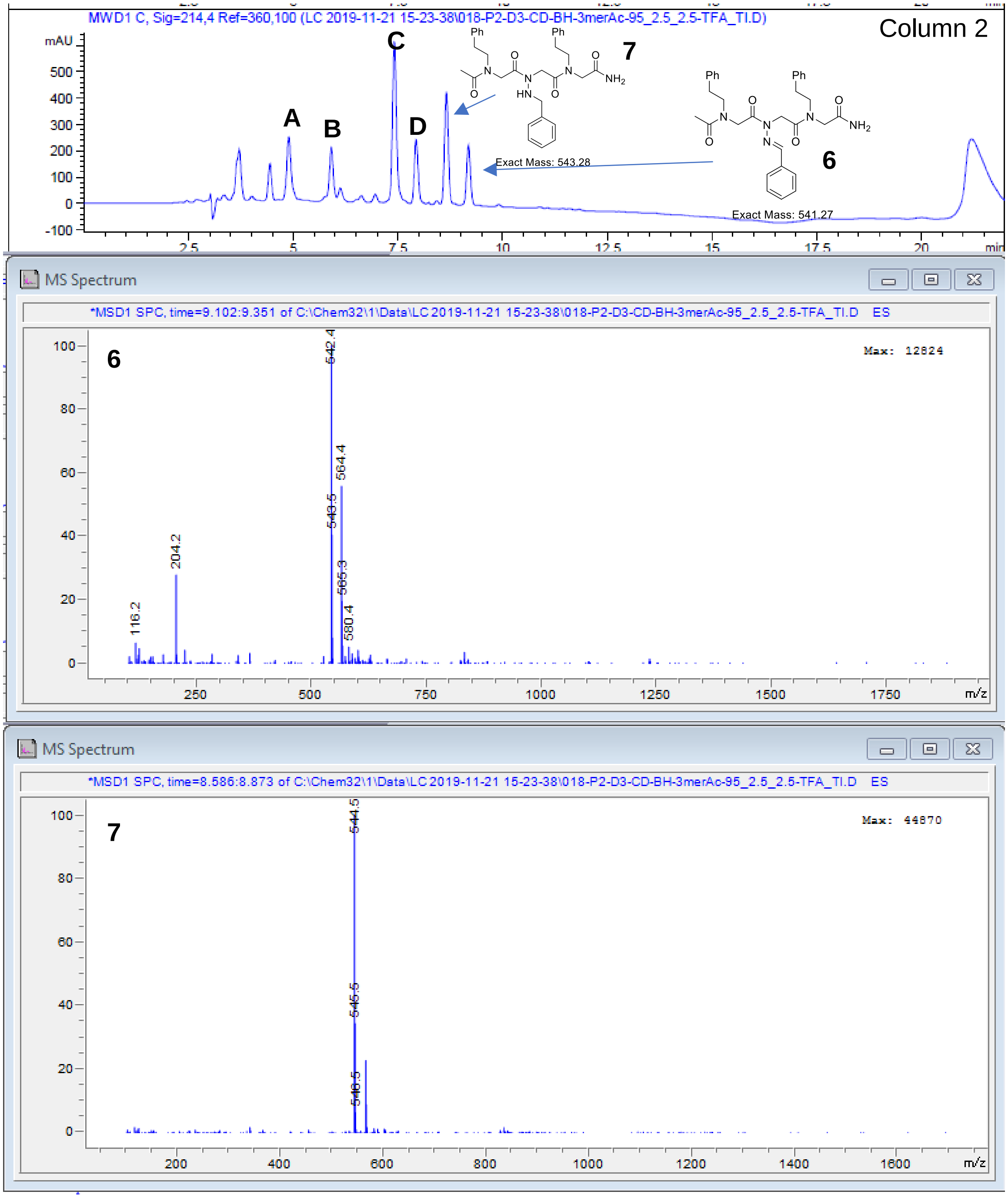

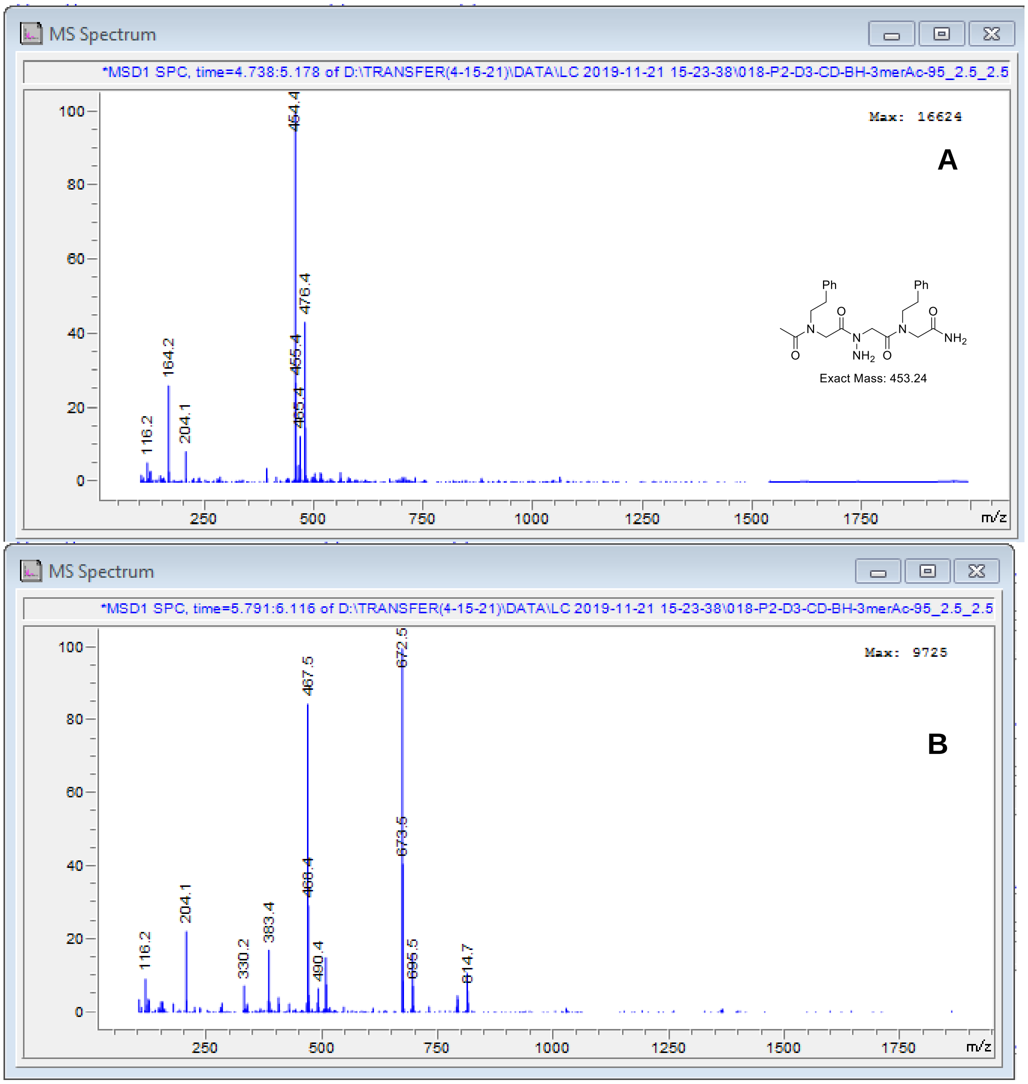
MS Spectrum
*MSD1 SPC, time=4.738:5.178 of D:\TRANSFER(4-15-21)\DATA\LC 2019-11-21 15-23-38\018-P2-D3-CD-BH-3merAc-95_2.5_2.5
Max: 16624
A
Exact Mass: 453.24
454.4
476.4
455.4
465.4
184.2
204.1
116.2
m/z
MS Spectrum
*MSD1 SPC, time=5.791:6.116 of D:\TRANSFER(4-15-21)\DATA\LC 2019-11-21 15-23-38\018-P2-D3-CD-BH-3merAc-95_2.5_2.5
Max: 9725
B
672.5
467.5
673.5
468.4
204.1
383.4
330.2
490.4
695.5
814.7
116.2
m/z

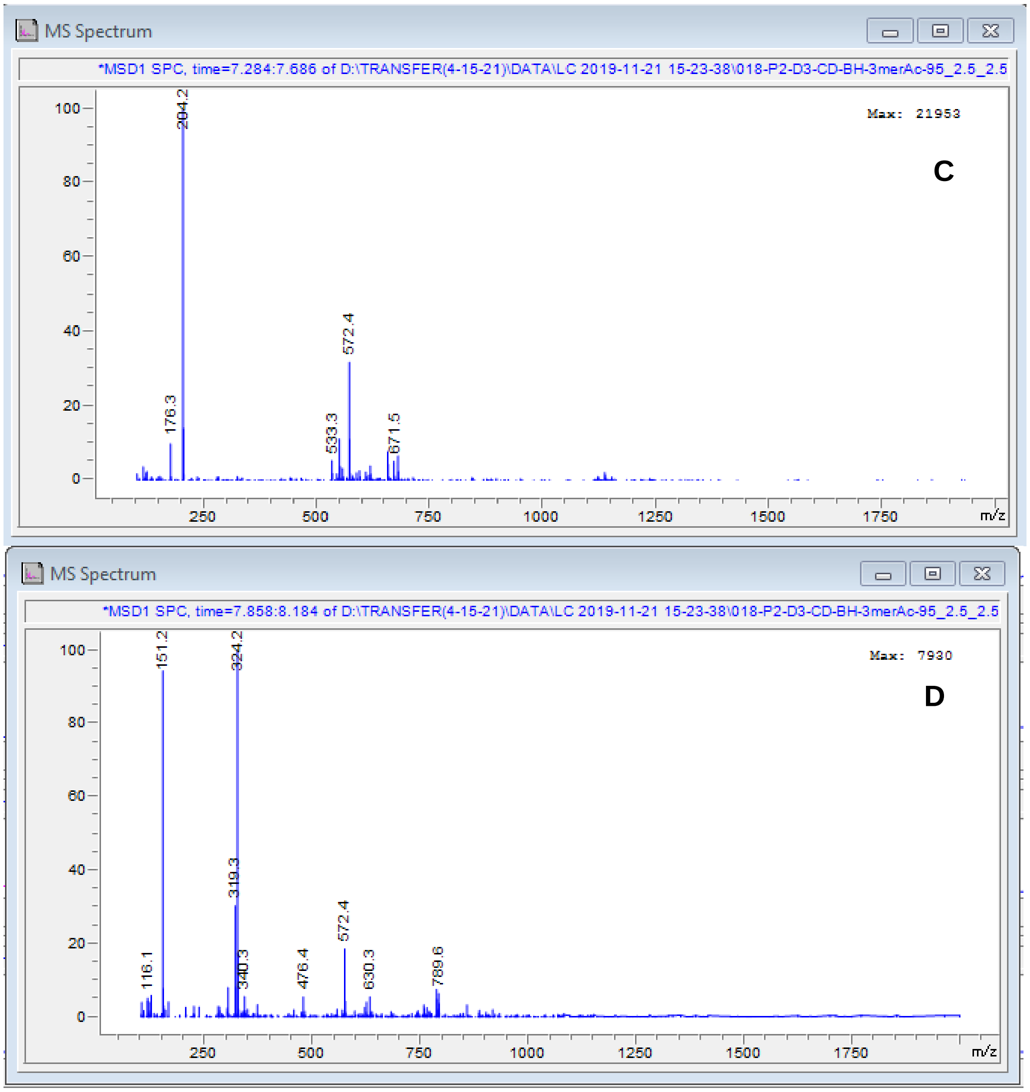
MS Spectrum
*MSD1 SPC, time=7.284:7.686 of D:\TRANSFER(4-15-21)\DATA\LC 2019-11-21 15-23-38\018-P2-D3-CD-BH-3merAc-95_2.5_2.5
Max: 21953
C
204.2
572.4
176.3
533.3
671.5
m/z
MS Spectrum
*MSD1 SPC, time=7.858:8.184 of D:\TRANSFER(4-15-21)\DATA\LC 2019-11-21 15-23-38\018-P2-D3-CD-BH-3merAc-95_2.5_2.5
Max: 7930
D
151.2
324.2
319.3
572.4
116.1
340.3
476.4
630.3
789.6
m/z

Davern, C.M.; Lowe, B.D.; Rosfi, A.; Ison, E.A.; Proulx, C.

Cleavage III: 95:5 TFA:H_2O 10 min

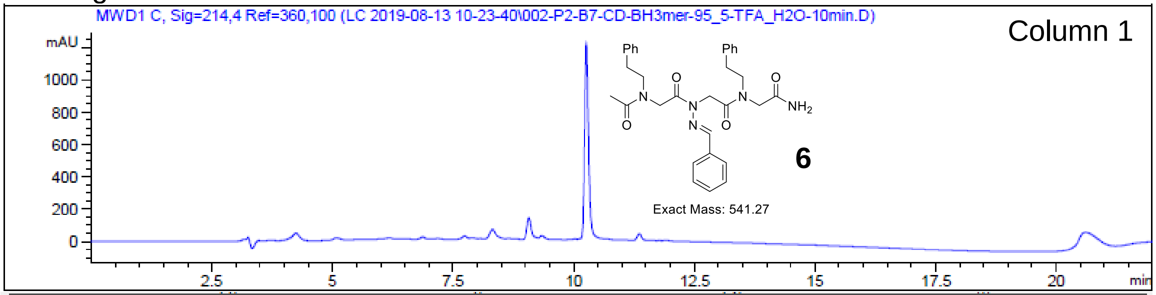

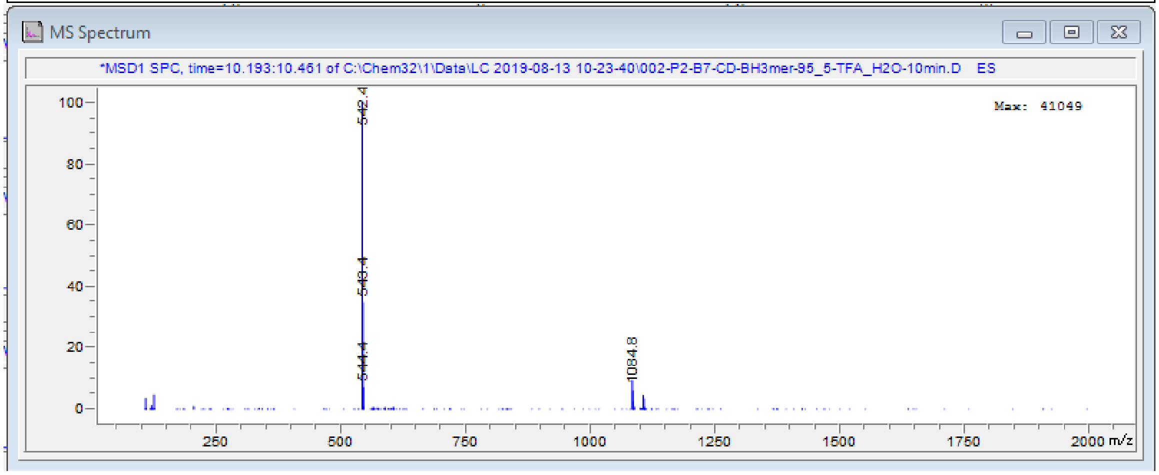

Cleavage IV: 95:5 TFA:H_2O 2h

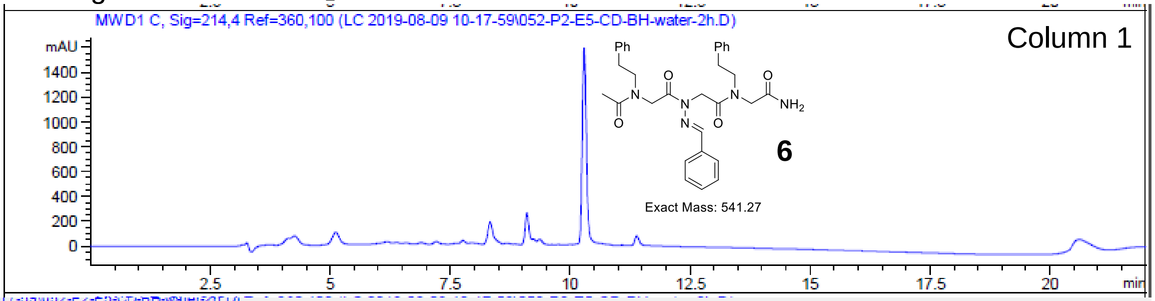

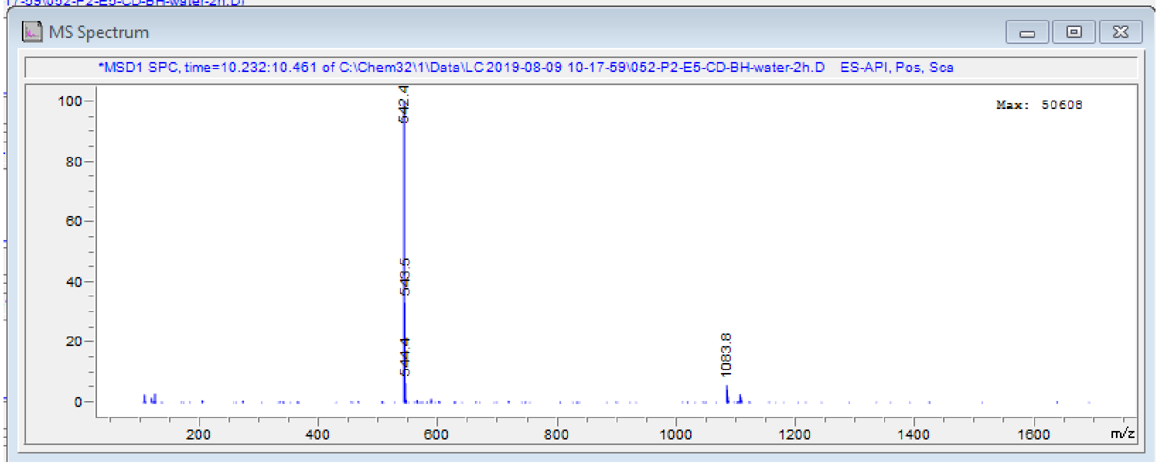

Cleavage V: 95:5 TFA:TES 10 min

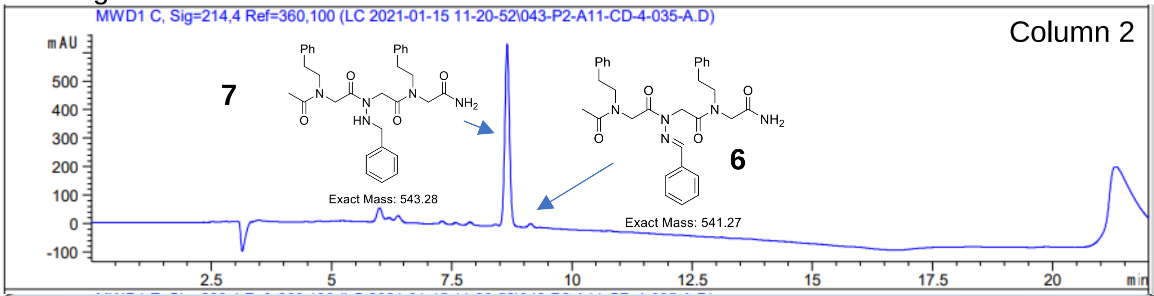

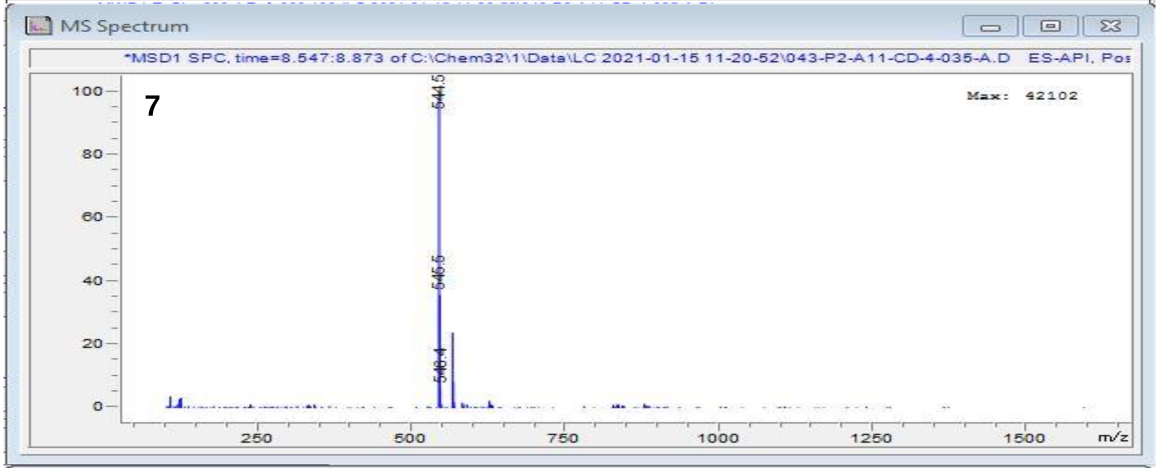

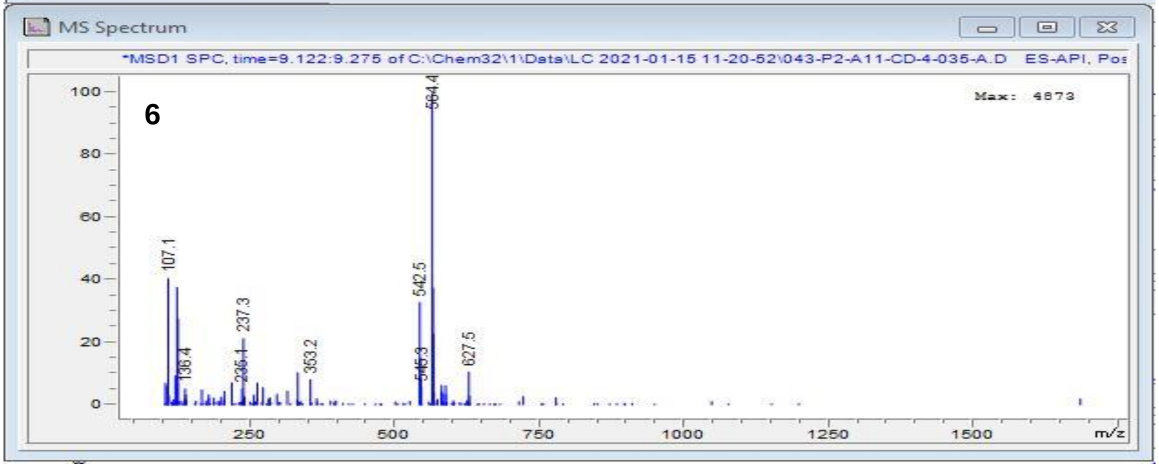

Davern, C.M.; Lowe, B.D.; Rosfi, A.; Ison, E.A.; Proulx, C.

Cleavage VI: 95:5 TFA:TES 2h

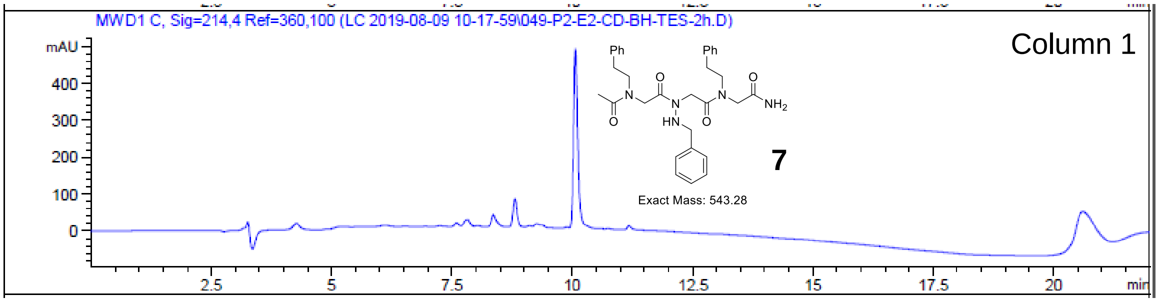

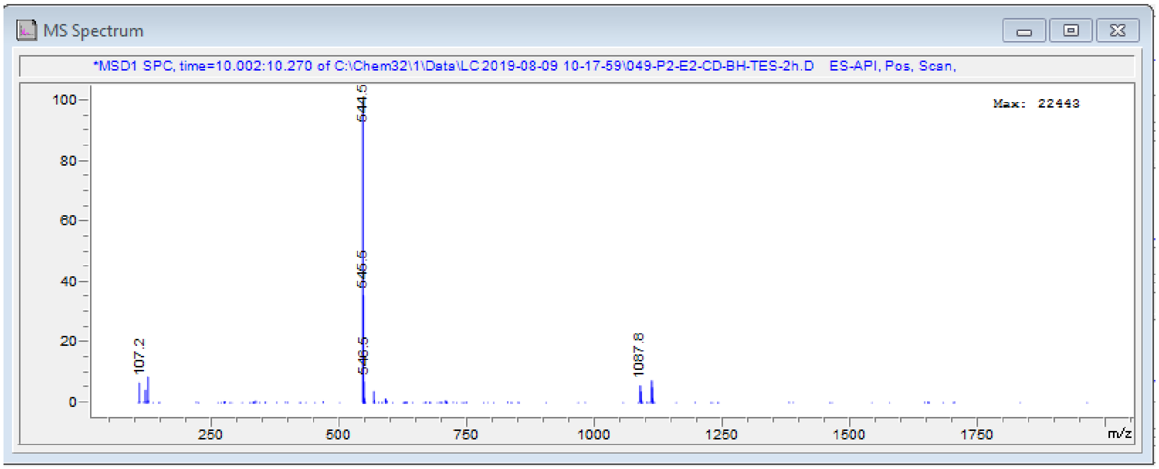

Cleavage VII: 9:1 TFA:TIPS 2h

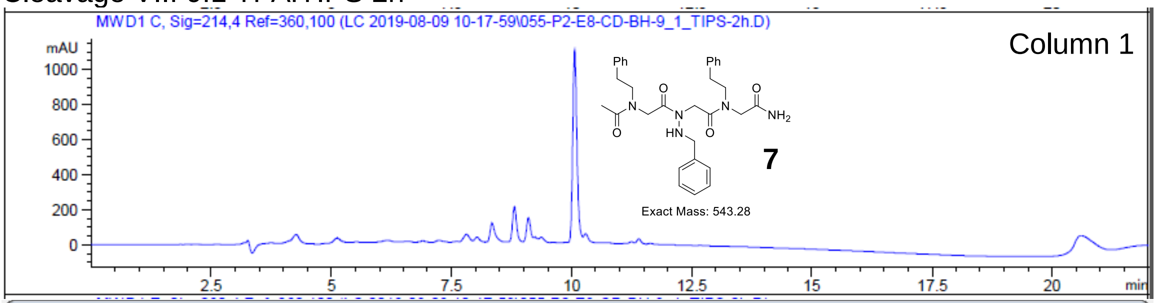

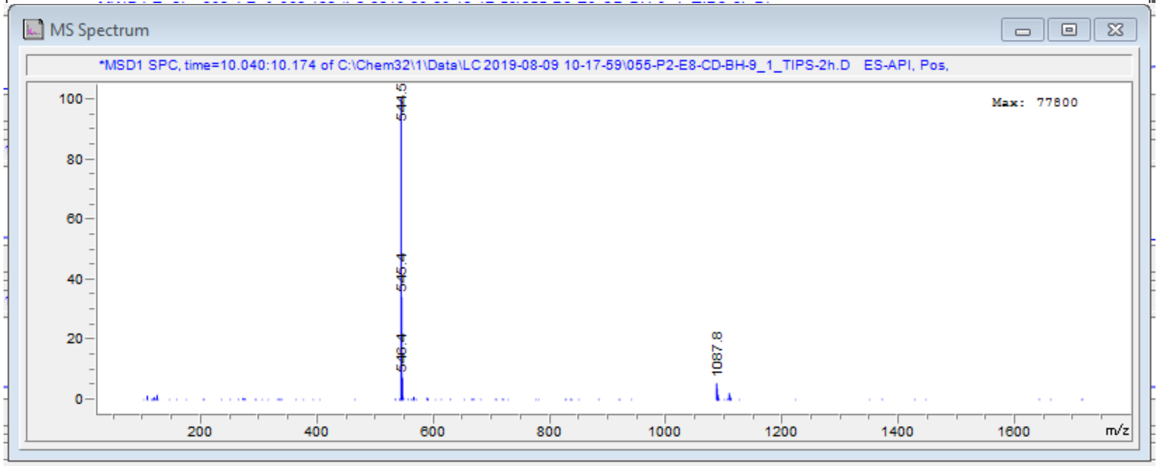

Cleavage VIII: 45:5:50 TFA:TIPS:DCM 10 min

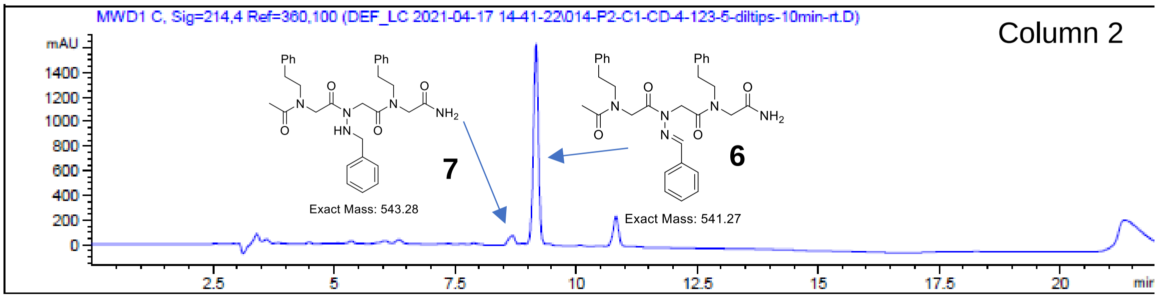

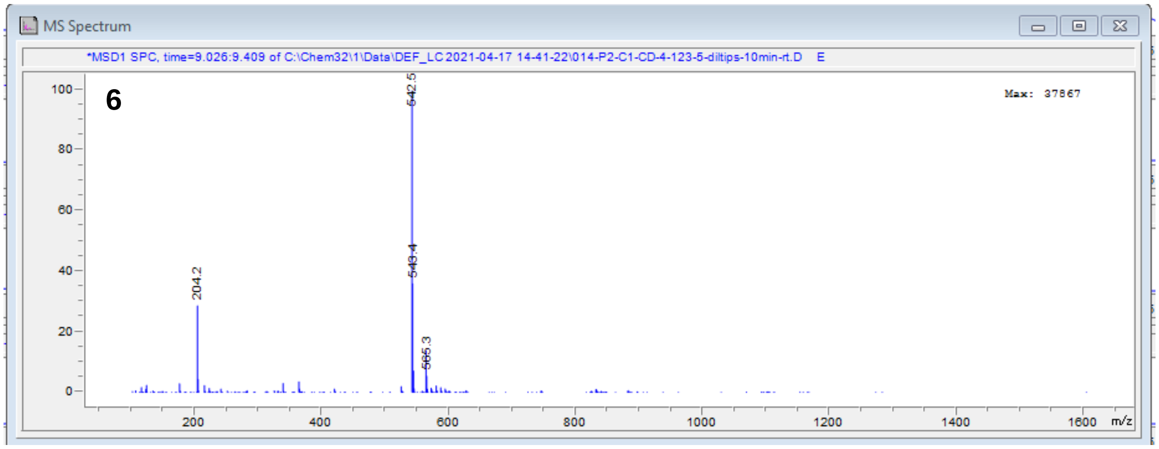

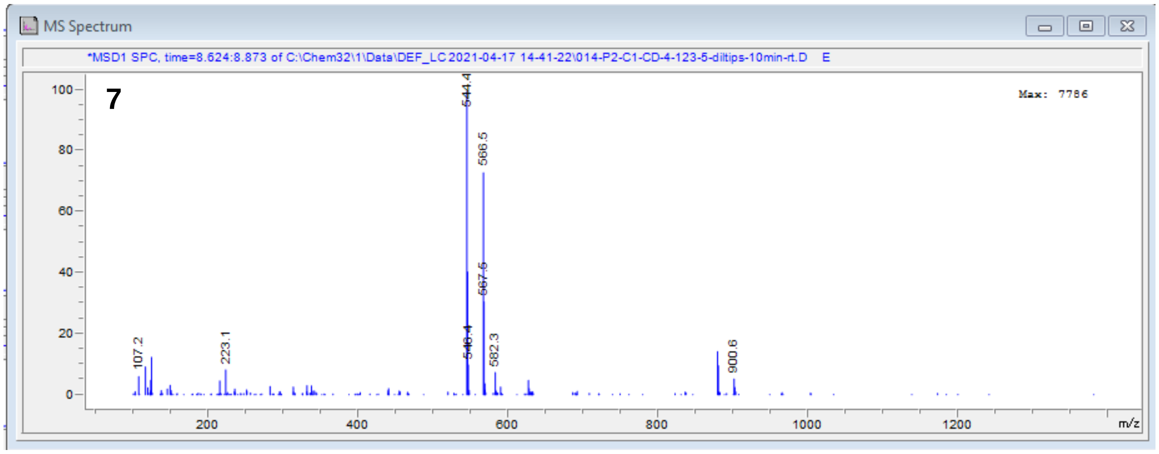

Davern, C.M.; Lowe, B.D.; Rosfi, A.; Ison, E.A.; Proulx, C.

Cleavage IX: 45:5:50 TFA:TIPS:DCM 2h

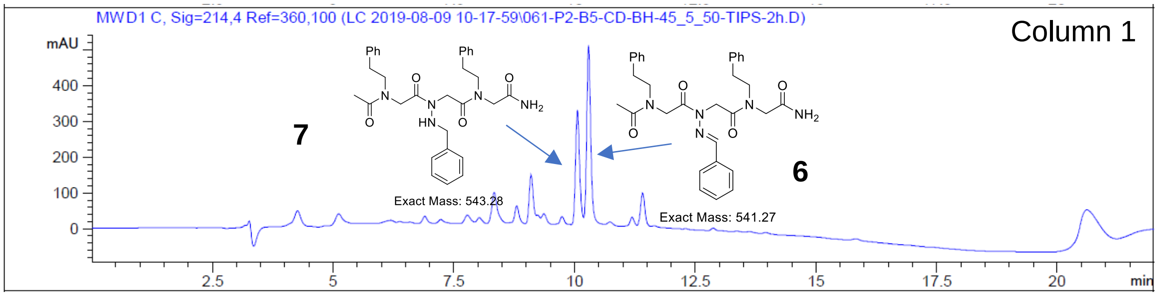

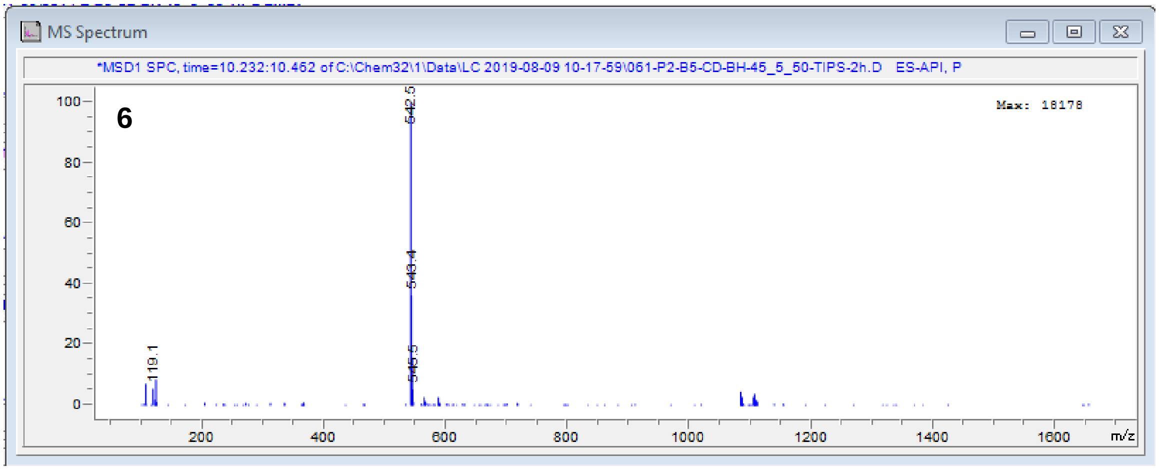

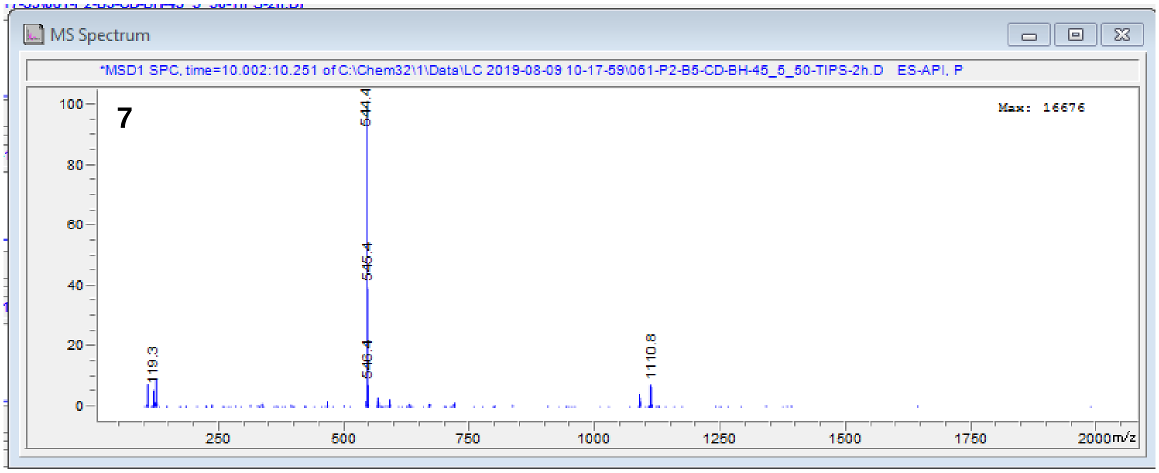

Cleavage X: 95:5 TFA:phenol 2 h followed by cold ether wash

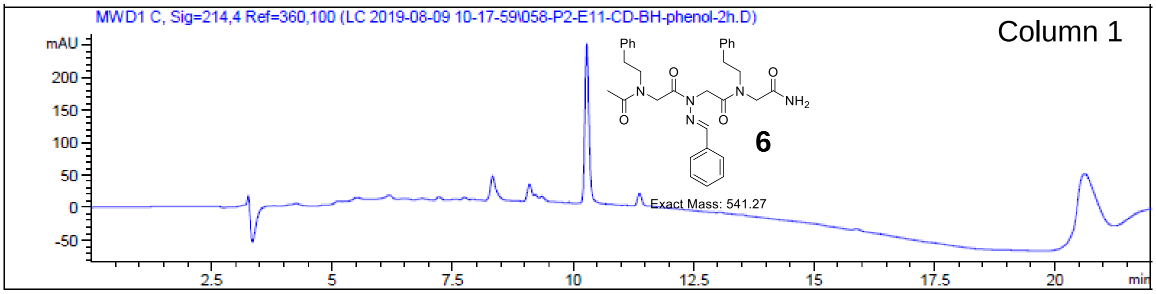

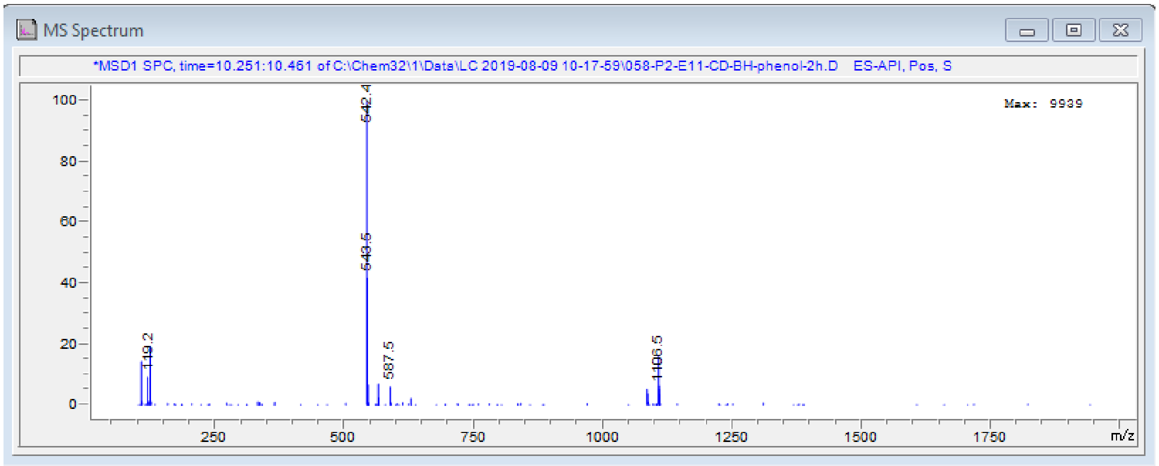

Characterization for S16-18 with Cleavage VIII – Traces correspond to data in **Table S4**

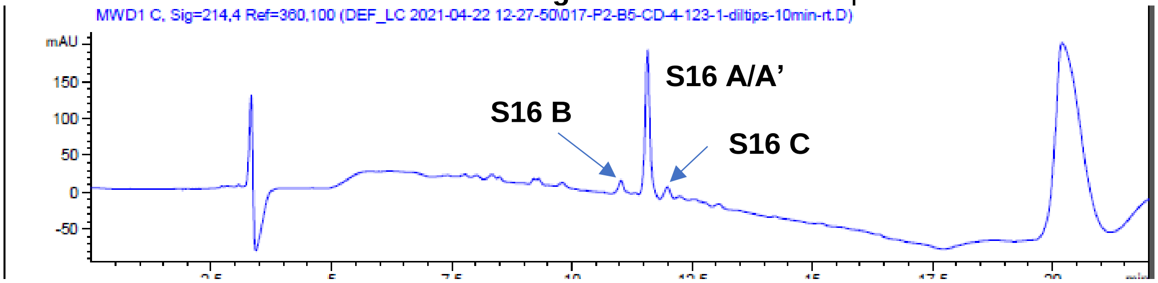

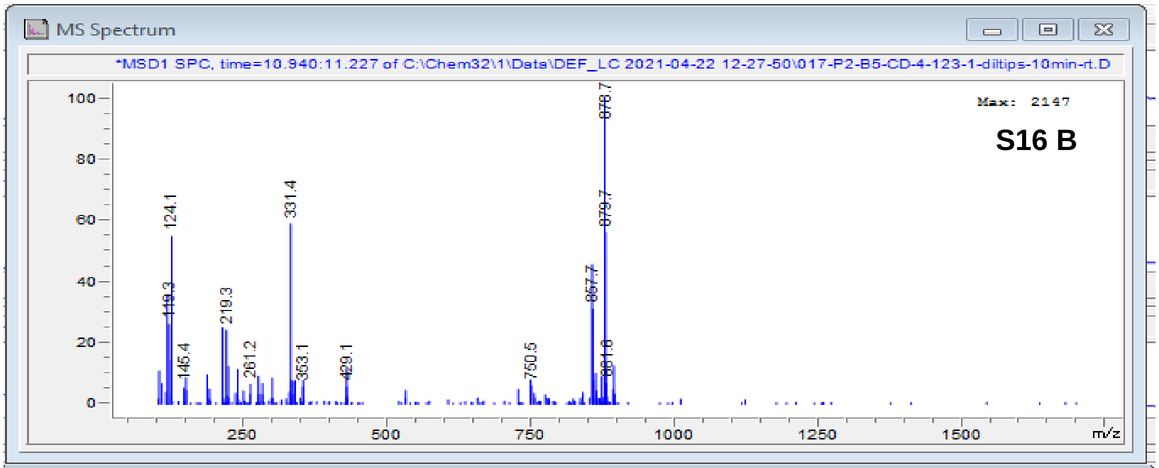

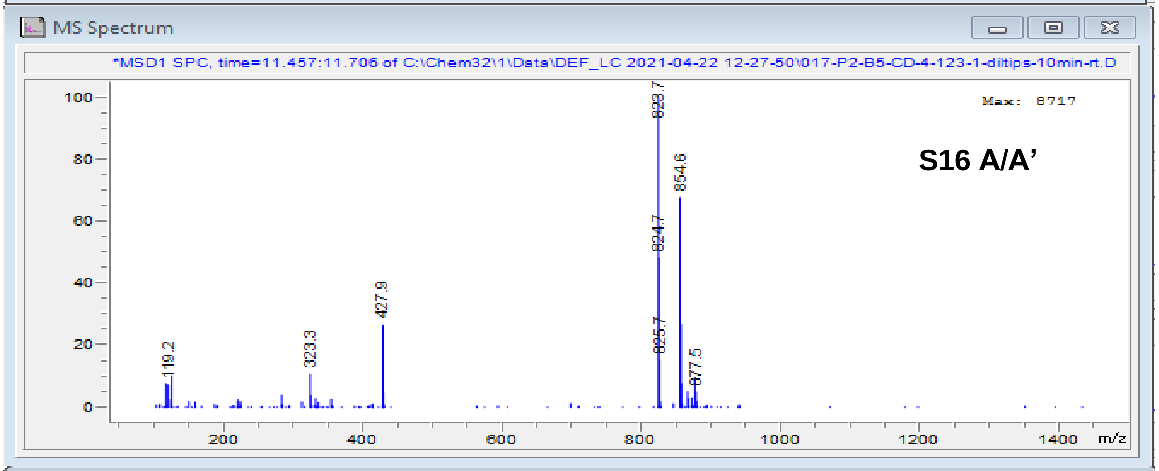

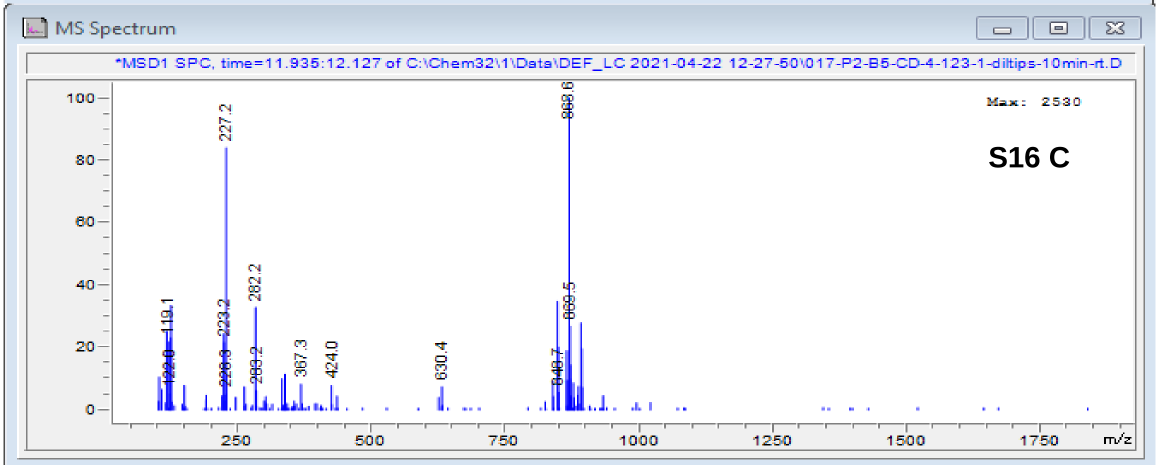

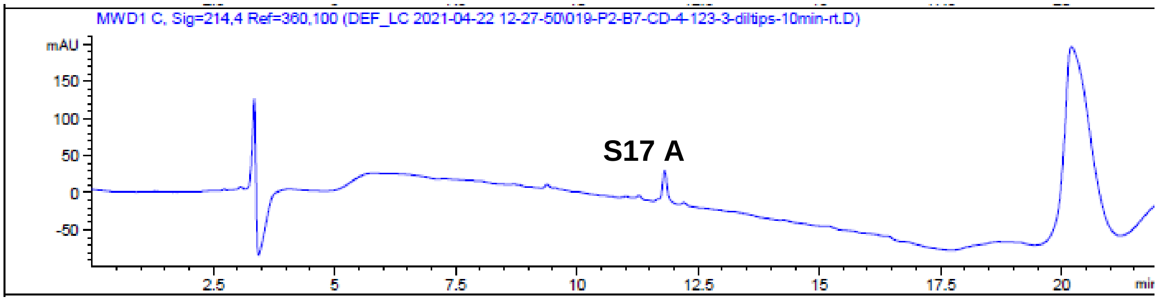
MWD1 C, Sig=214,4 Ref=360,100 (DEF_LC 2021-04-22 12-27-50\019-P2-B7-CD-4-123-3-diltips-10min-rt.D)
mAU
150
100
50
0
-50
S17 A
2.5
5
7.5
10
12.5
15
17.5
20

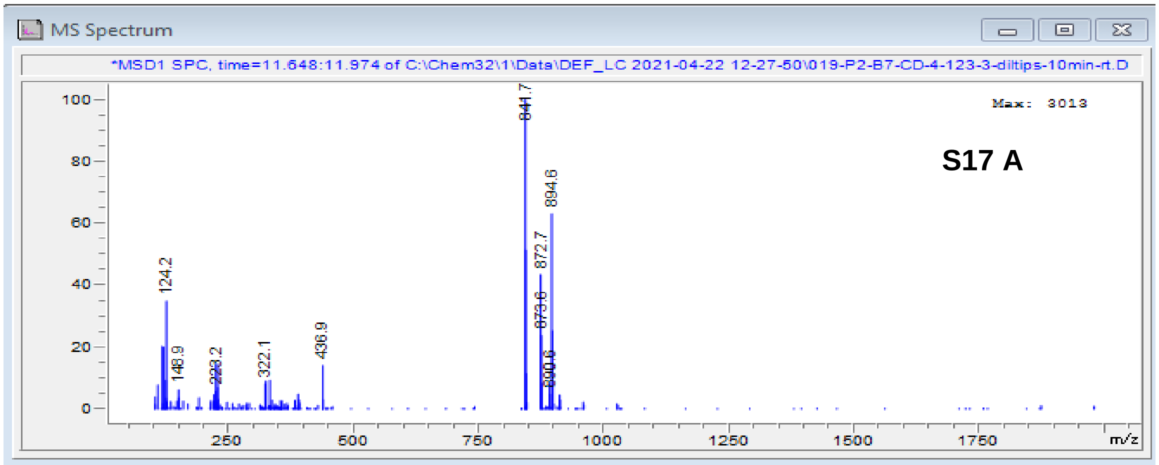
MS Spectrum
*MSD1 SPC, time=11.648:11.974 of C:\Chem32\1\Data\DEF_LC 2021-04-22 12-27-50\019-P2-B7-CD-4-123-3-diltips-10min-rt.D
Max: 3013
S17 A
100
80
60
40
20
0
124.2
148.9
229.2
322.1
436.9
841.7
894.6
872.7
873.6
900.6
250
500
750
1000
1250
1500
1750
m/z

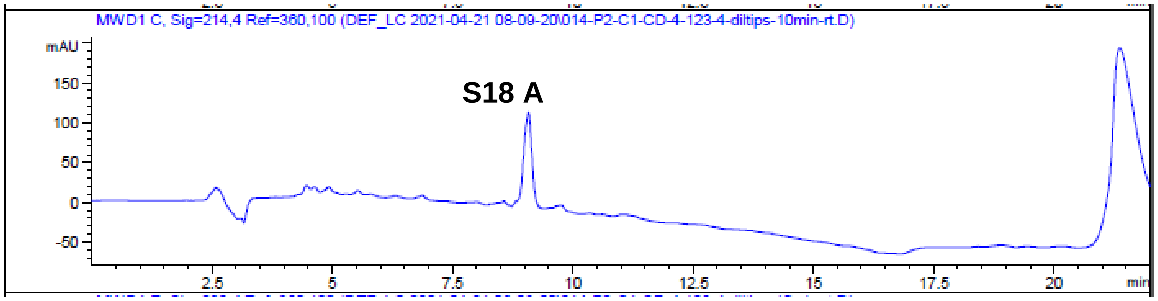
MWD1 C, Sig=214,4 Ref=360,100 (DEF_LC 2021-04-21 08-09-20\014-P2-C1-CD-4-123-4-diltips-10min-rt.D)
mAU
150
100
50
0
-50
S18 A
2.5
5
7.5
10
12.5
15
17.5
20

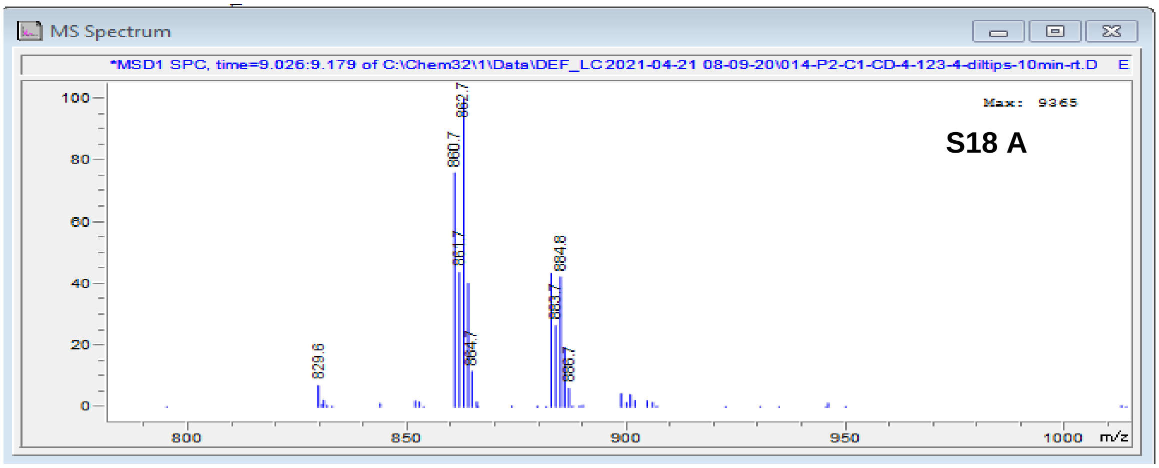
MS Spectrum
*MSD1 SPC, time=9.026:9.179 of C:\Chem32\1\Data\DEF_LC 2021-04-21 08-09-20\014-P2-C1-CD-4-123-4-diltips-10min-rt.D
Max: 9365
S18 A
100
80
60
40
20
0
829.6
860.7
862.7
861.7
864.7
884.8
883.7
886.7
800
850
900
950
1000
m/z

Manual and Automated Synthesis of oligomers with 5 hydrazones

8

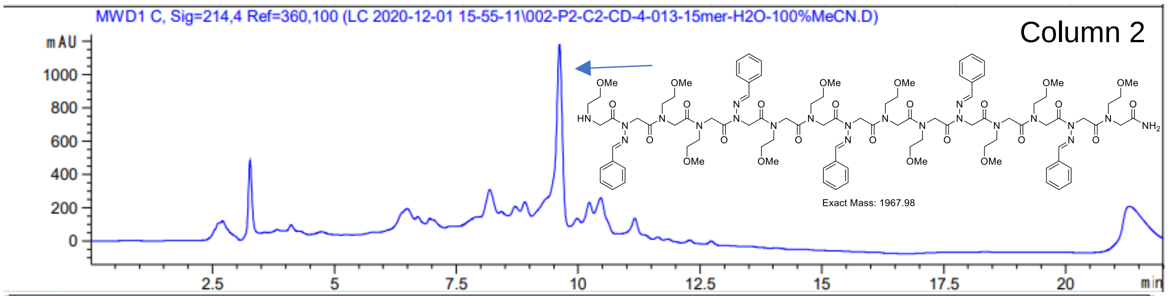

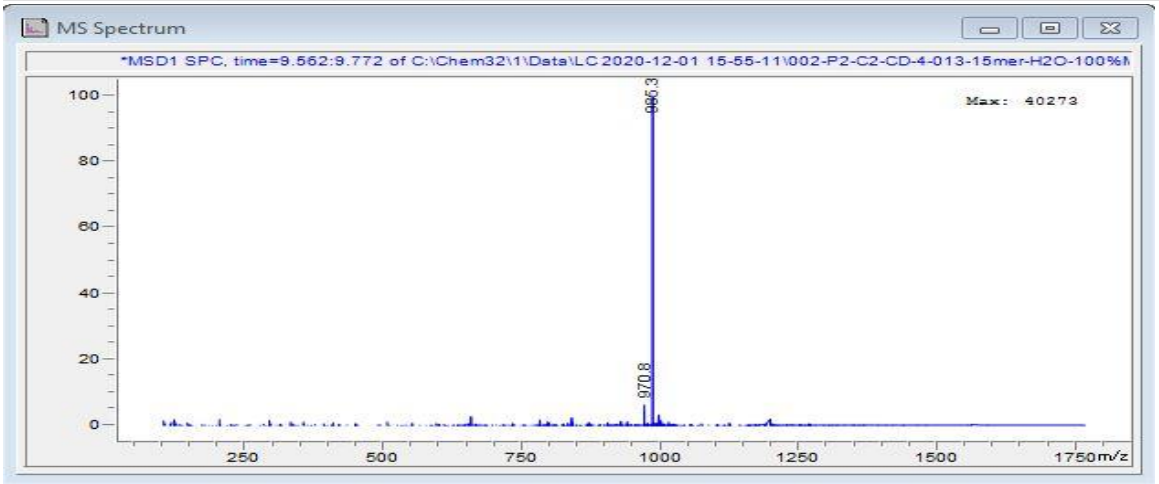

9

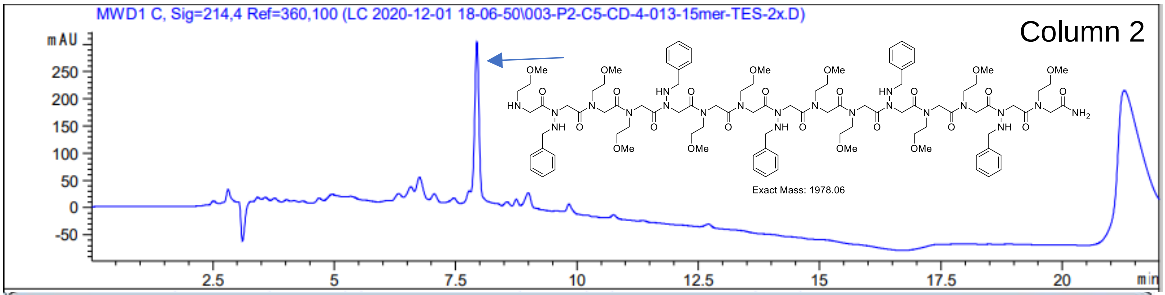

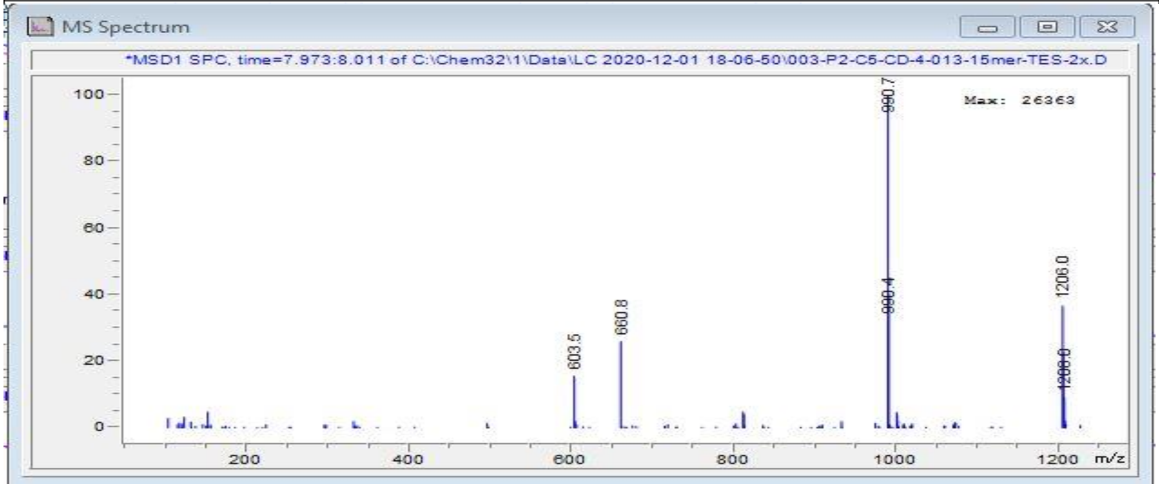

S19 (Manual):

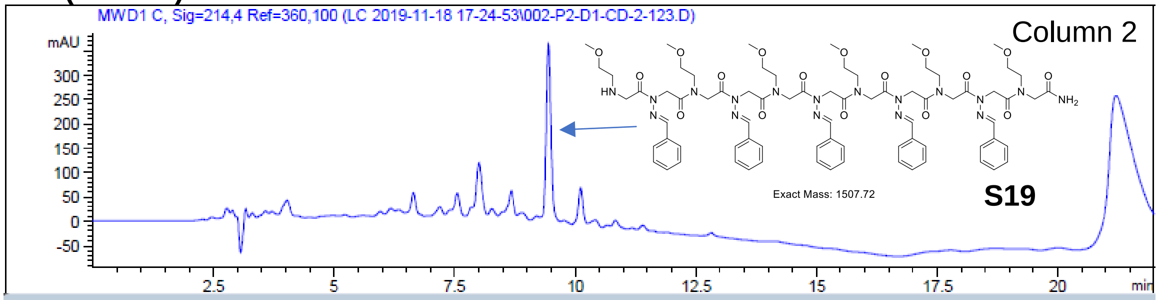

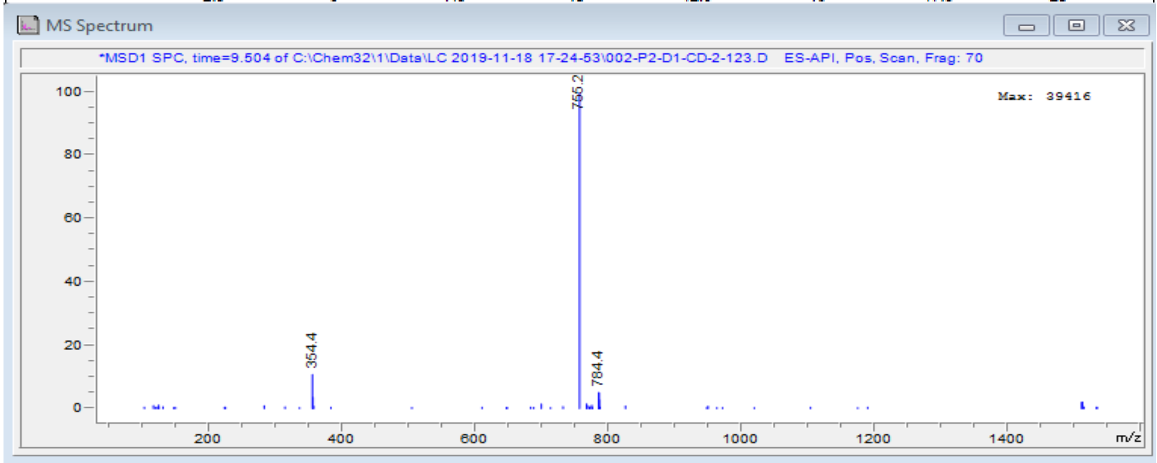

S19 (Automated):

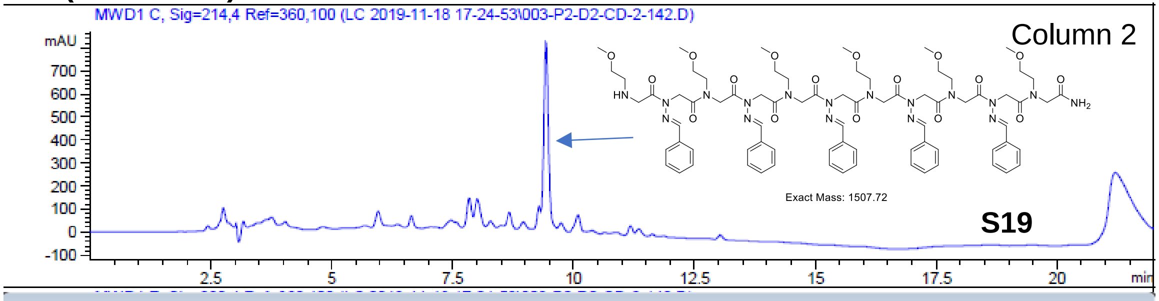

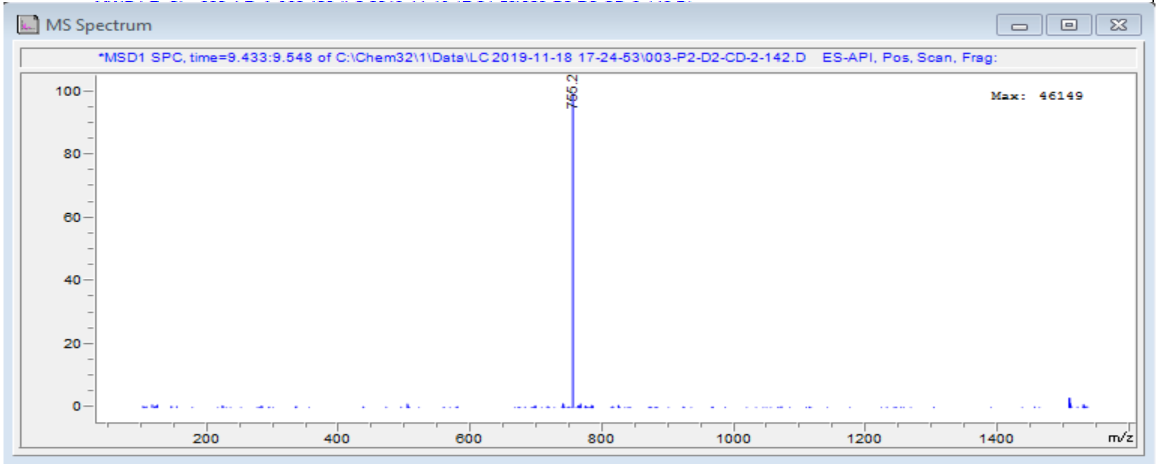

Coinjection of S19 (manual) and S19 (automated) samples:

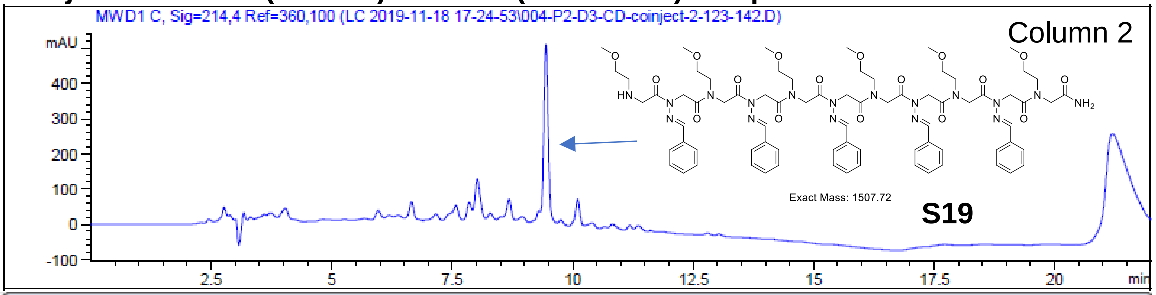

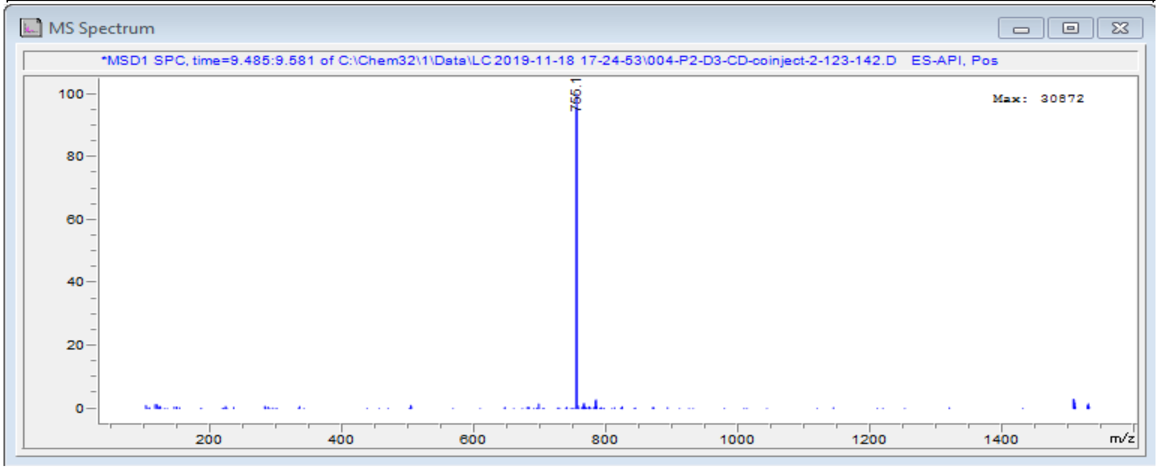

S20:

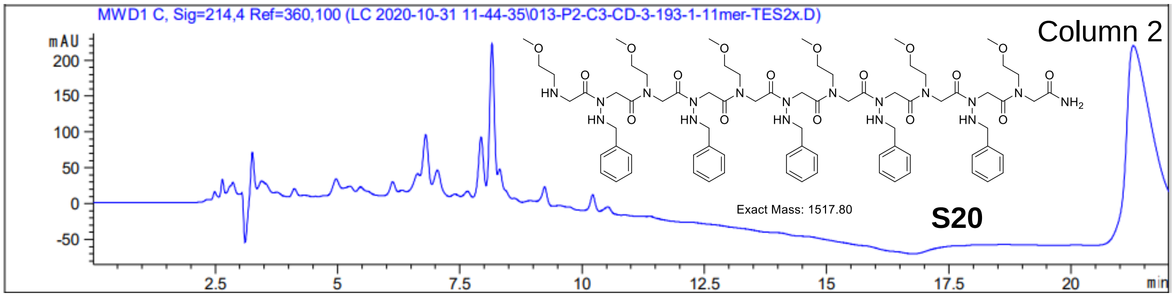

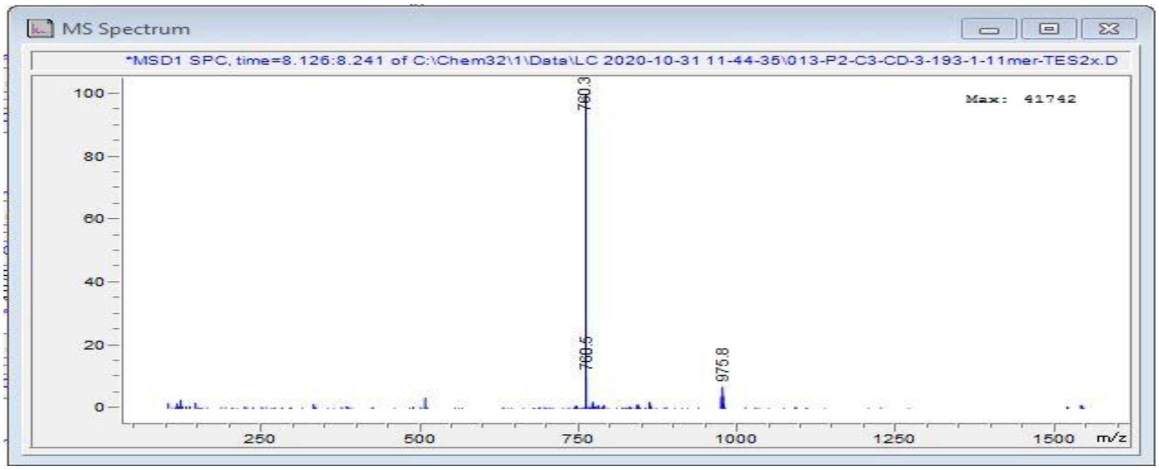

S21:

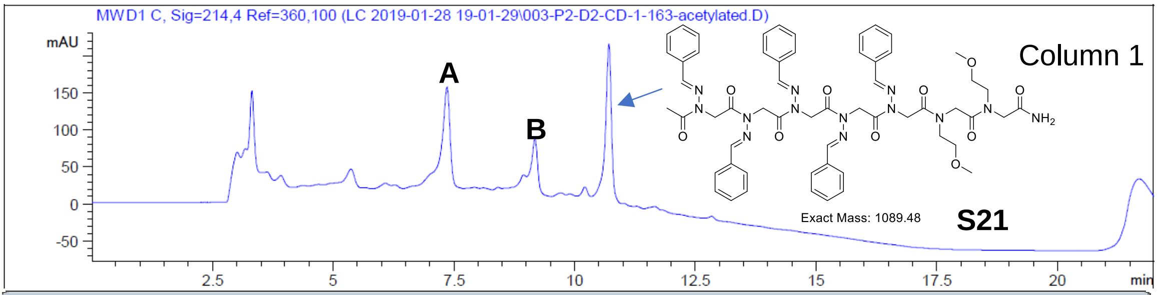
MWD1 C, Sig=214,4 Ref=360,100 (LC 2019-01-28 19-01-29\003-P2-D2-CD-1-163-acetylated.D)
mAU
A
B
Column 1
Exact Mass: 1089.48
S21
min

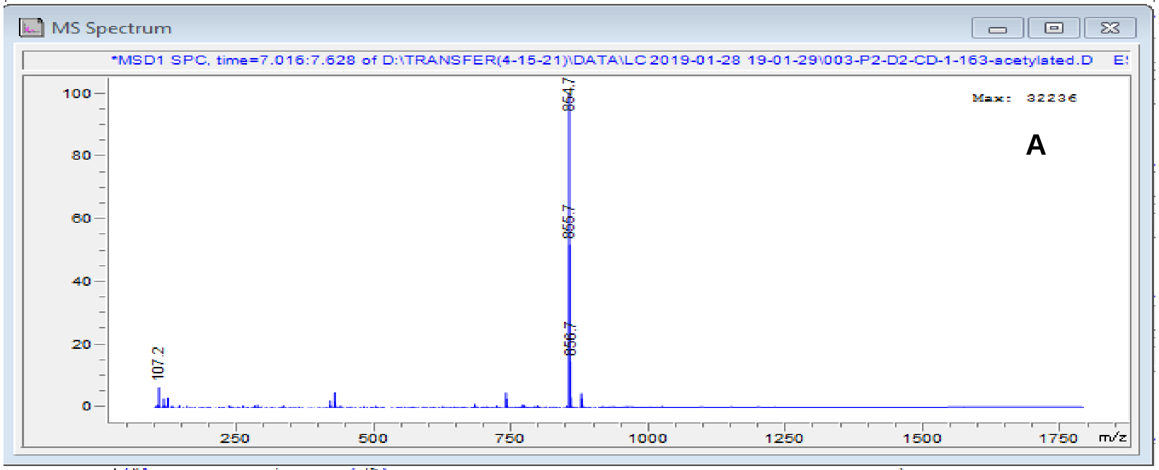
MS Spectrum
*MSD1 SPC, time=7.016:7.628 of D:\TRANSFER(4-15-21)\DATA\LC 2019-01-28 19-01-29\003-P2-D2-CD-1-163-acetylated.D
Max: 32236
A
854.7
855.7
107.2
m/z

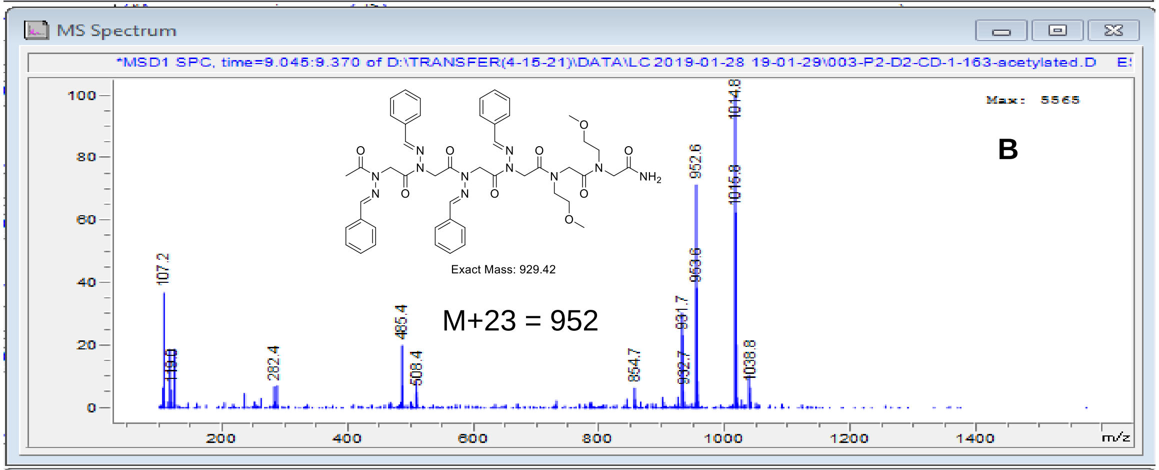
MS Spectrum
*MSD1 SPC, time=9.045:9.370 of D:\TRANSFER(4-15-21)\DATA\LC 2019-01-28 19-01-29\003-P2-D2-CD-1-163-acetylated.D
Max: 5565
B
Exact Mass: 929.42
M+23 = 952
1014.8
1015.8
952.6
953.6
107.2
485.4
508.4
282.4
854.7
931.7
1038.8
m/z

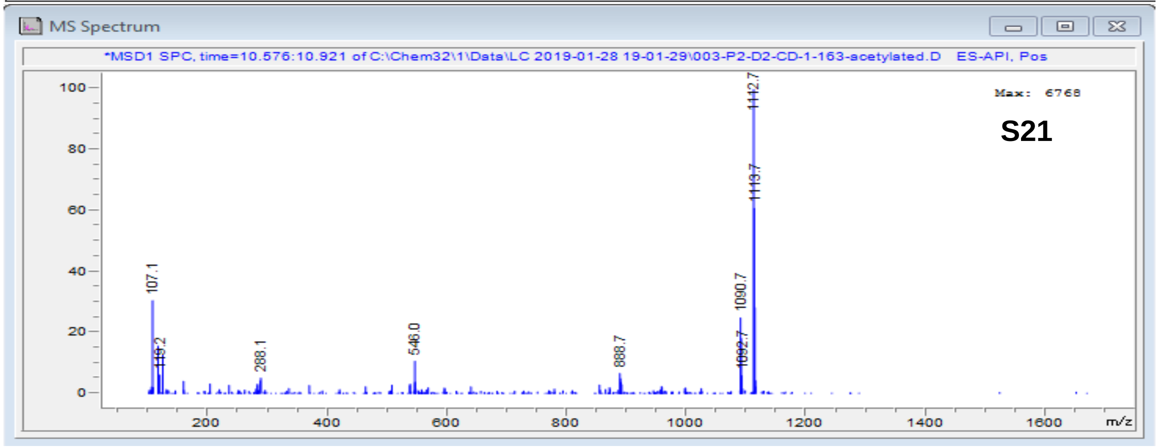
MS Spectrum
*MSD1 SPC, time=10.576:10.921 of C:\Chem32\1\Data\LC 2019-01-28 19-01-29\003-P2-D2-CD-1-163-acetylated.D ES-API, Pos
Max: 6768
S21
1112.7
1113.7
1090.7
107.1
288.1
546.0
888.7
m/z

S22:

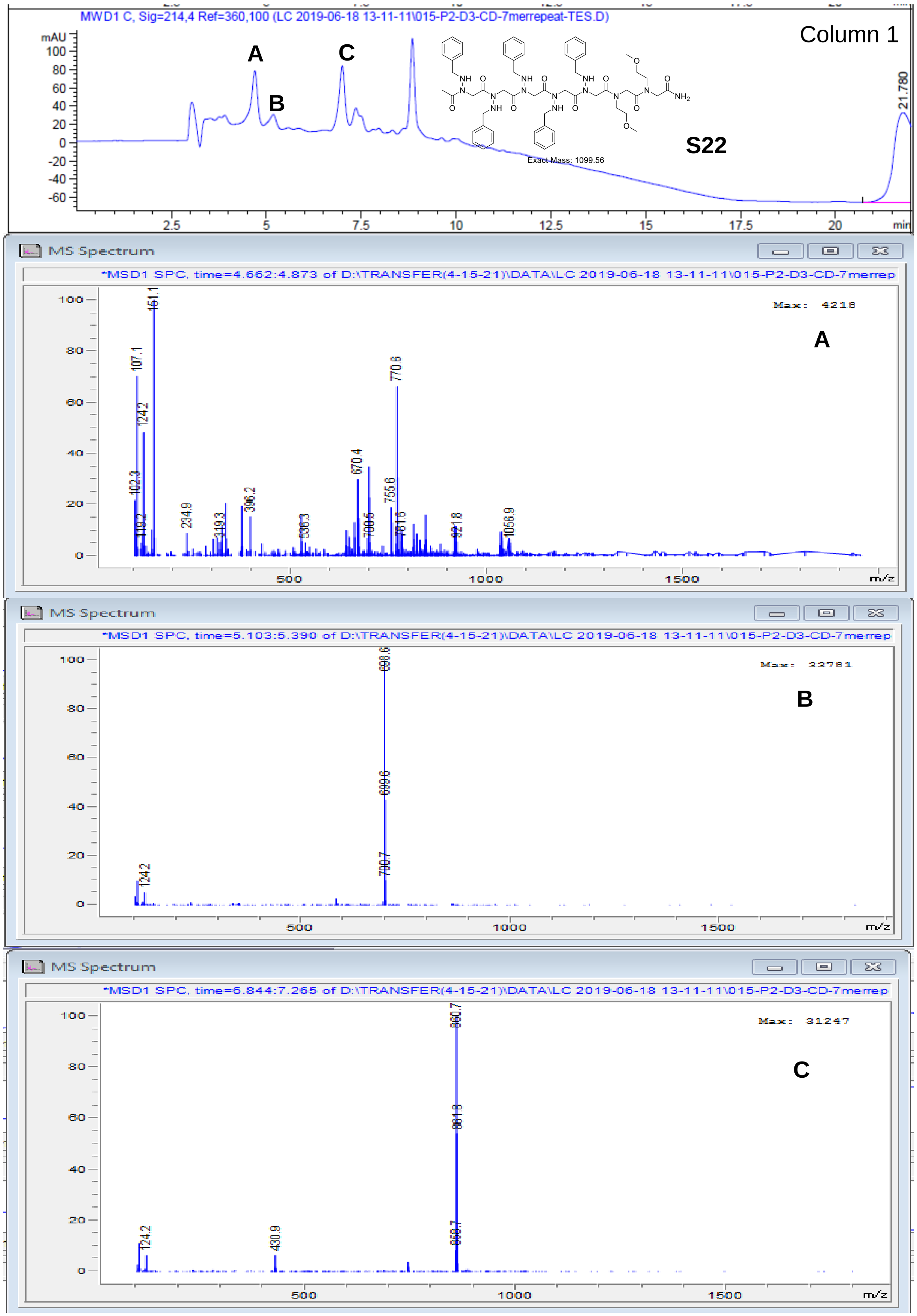
MWD1 C, Sig=214,4 Ref=360,100 (LC 2019-06-18 13-11-11\015-P2-D3-CD-7merrepeat-TES.D)
Column 1
A
B
C
S22
Exact Mass: 1099.56
21.780
mAU
min
MS Spectrum
*MSD1 SPC, time=4.662:4.873 of D:\TRANSFER(4-15-21)\DATA\LC 2019-06-18 13-11-11\015-P2-D3-CD-7merrep
Max: 4218
A
151.1
107.1
124.2
102.3
770.6
670.4
755.6
396.2
234.9
921.8
1056.9
m/z
MS Spectrum
*MSD1 SPC, time=5.103:5.390 of D:\TRANSFER(4-15-21)\DATA\LC 2019-06-18 13-11-11\015-P2-D3-CD-7merrep
Max: 33781
B
698.6
699.6
700.7
124.2
m/z
MS Spectrum
*MSD1 SPC, time=6.844:7.265 of D:\TRANSFER(4-15-21)\DATA\LC 2019-06-18 13-11-11\015-P2-D3-CD-7merrep
Max: 31247
C
860.7
861.8
858.7
430.9
124.2
m/z

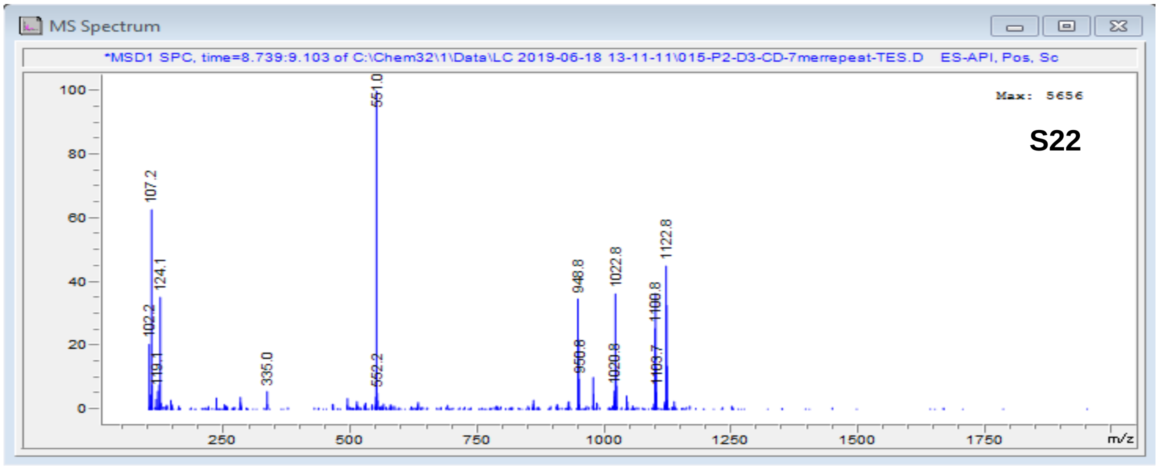
MS Spectrum
*MSD1 SPC, time=8.739:9.103 of C:\Chem32\1\Data\LC 2019-06-18 13-11-11\015-P2-D3-CD-7merrepeat-TES.D ES-API, Pos, Sc
Max: 5656
S22
m/z

Davern, C.M.; Lowe, B.D.; Rosfi, A.; Ison, E.A.; Proulx, C.

Characterization for crude peptoid pentamers 11a-l and 12a-l

11a

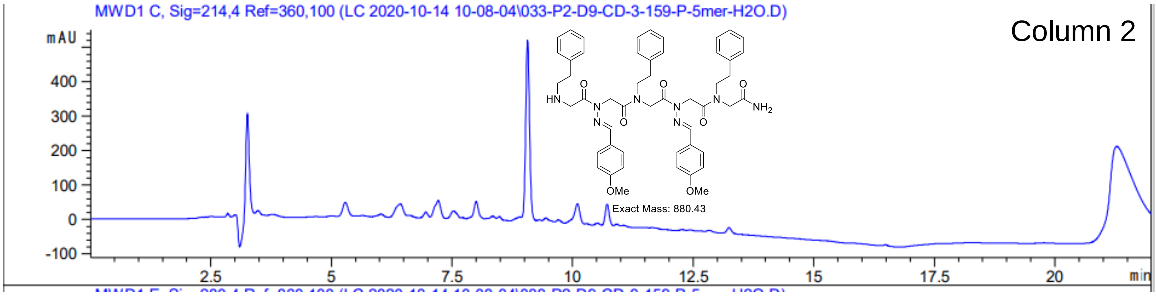

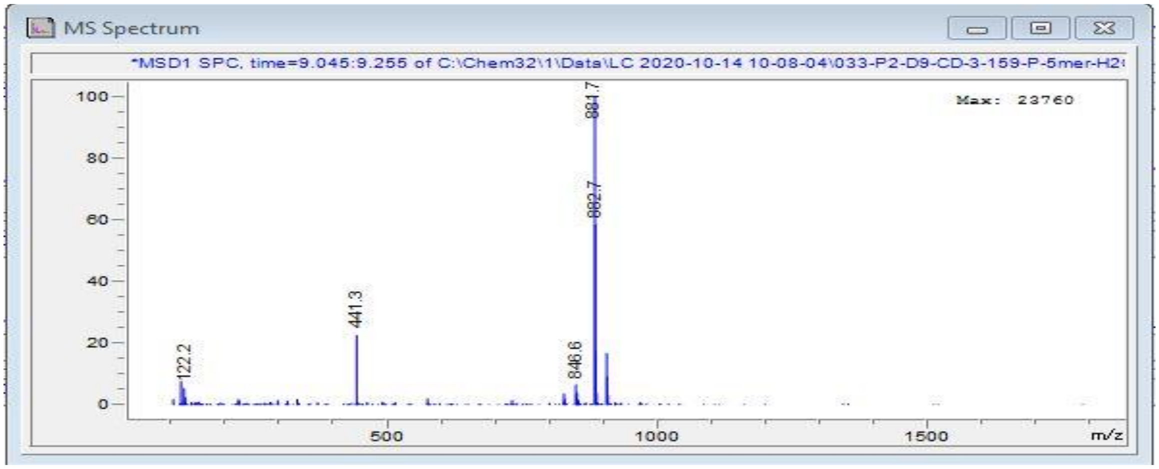

12a

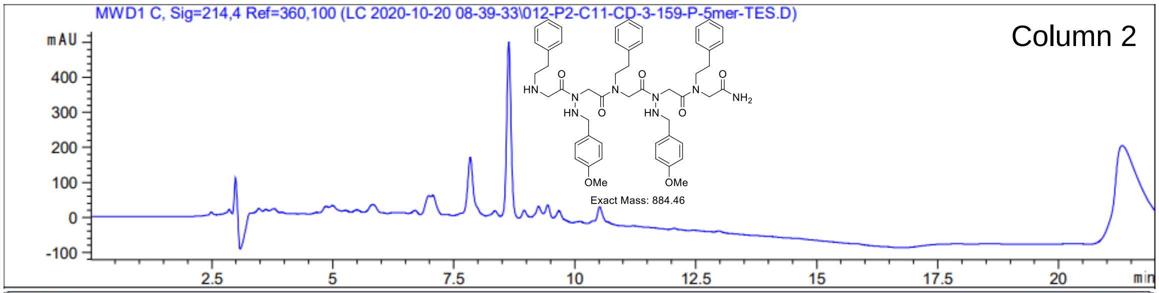

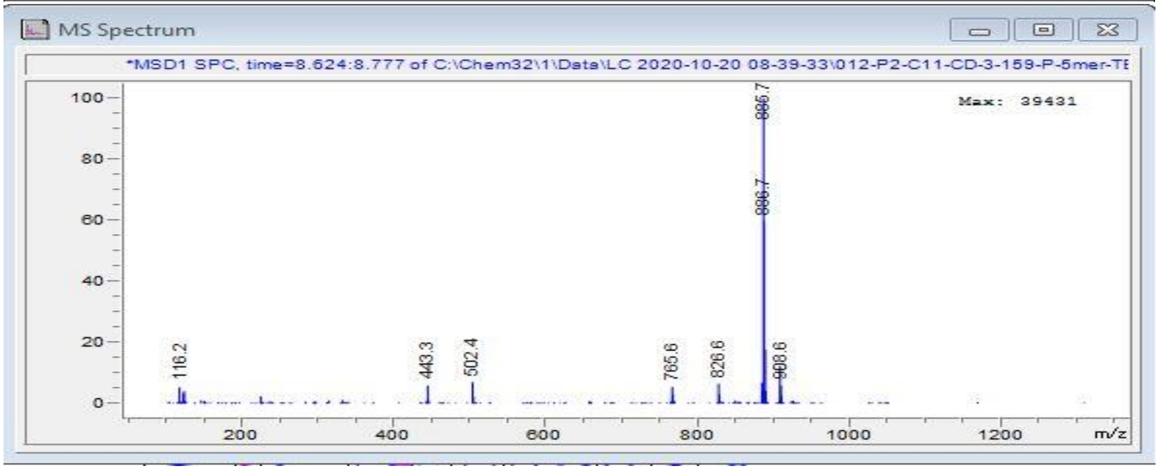

11b

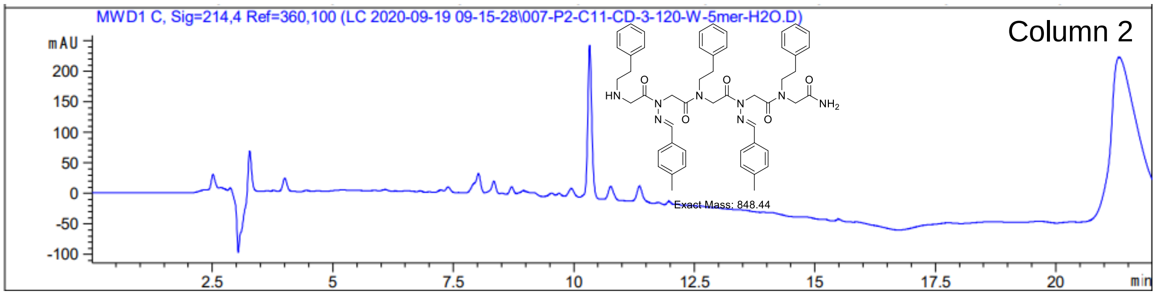
MWD1 C, Sig=214,4 Ref=360,100 (LC 2020-09-19 09-15-28\007-P2-C11-CD-3-120-W-5mer-H2O.D)
Column 2
mAU
Exact Mass: 848.44
min

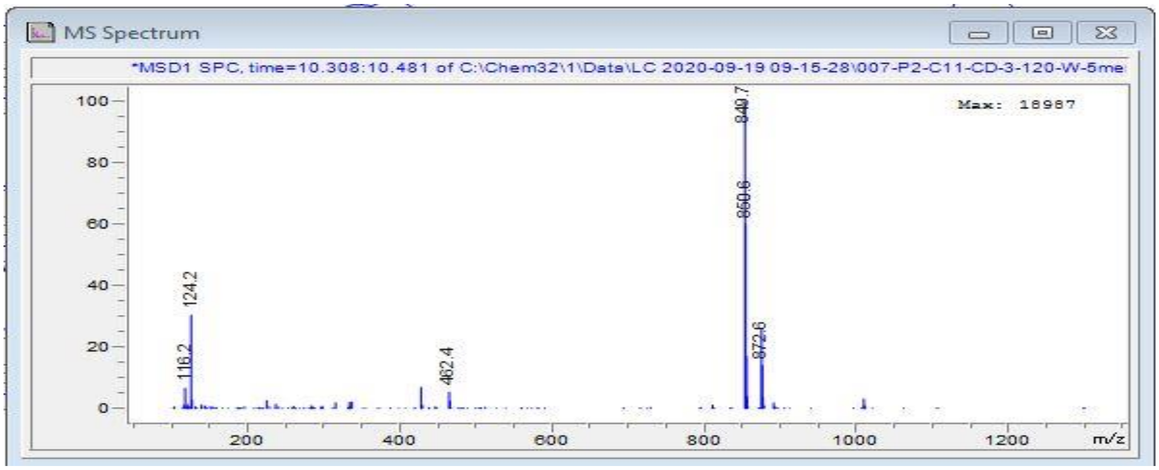
MS Spectrum
*MSD1 SPC, time=10.308:10.481 of C:\Chem32\1\Data\LC 2020-09-19 09-15-28\007-P2-C11-CD-3-120-W-5me
Max: 18987
849.7
850.6
872.6
124.2
116.2
462.4
m/z

12b

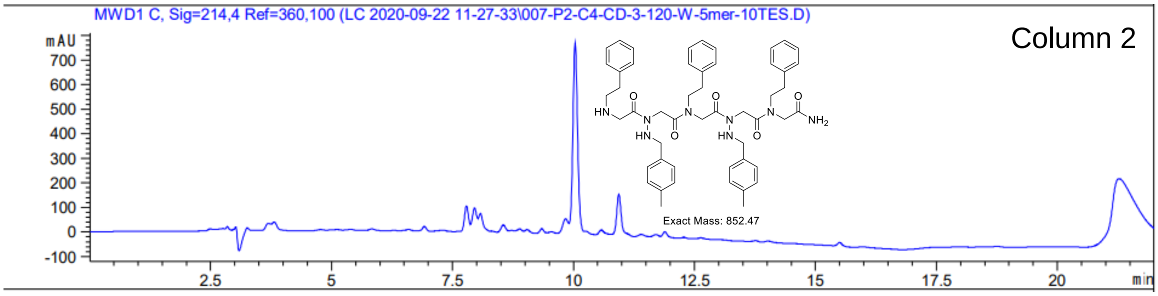
MWD1 C, Sig=214,4 Ref=360,100 (LC 2020-09-22 11-27-33\007-P2-C4-CD-3-120-W-5mer-10TES.D)
Column 2
mAU
Exact Mass: 852.47
min

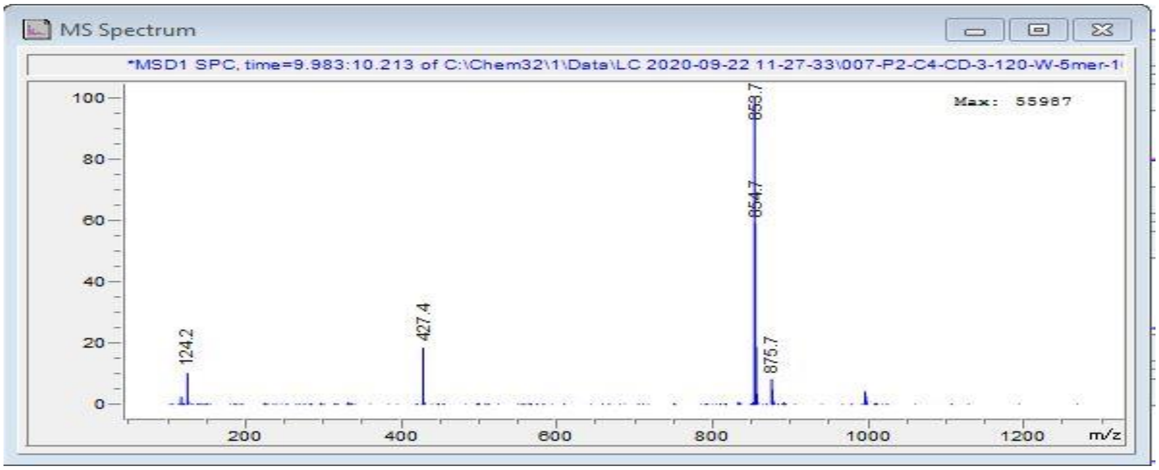
MS Spectrum
*MSD1 SPC, time=9.983:10.213 of C:\Chem32\1\Data\LC 2020-09-22 11-27-33\007-P2-C4-CD-3-120-W-5mer-1
Max: 55987
853.7
854.7
875.7
427.4
124.2
m/z

11c

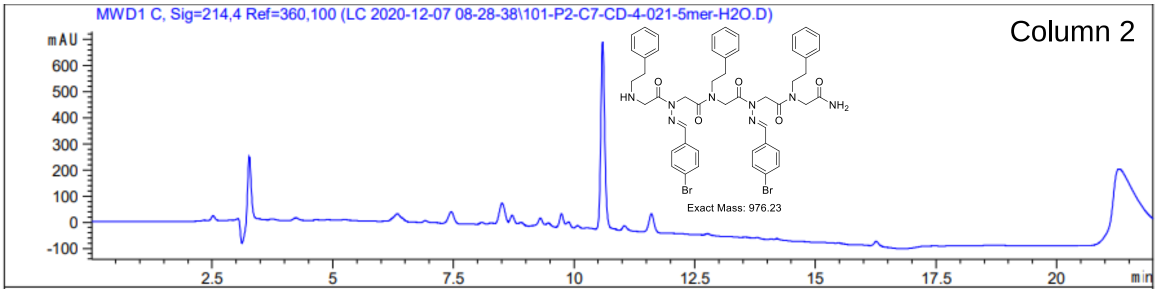
MWD1 C, Sig=214,4 Ref=360,100 (LC 2020-12-07 08-28-38\101-P2-C7-CD-4-021-5mer-H2O.D)
Column 2
mAU
Exact Mass: 976.23
min

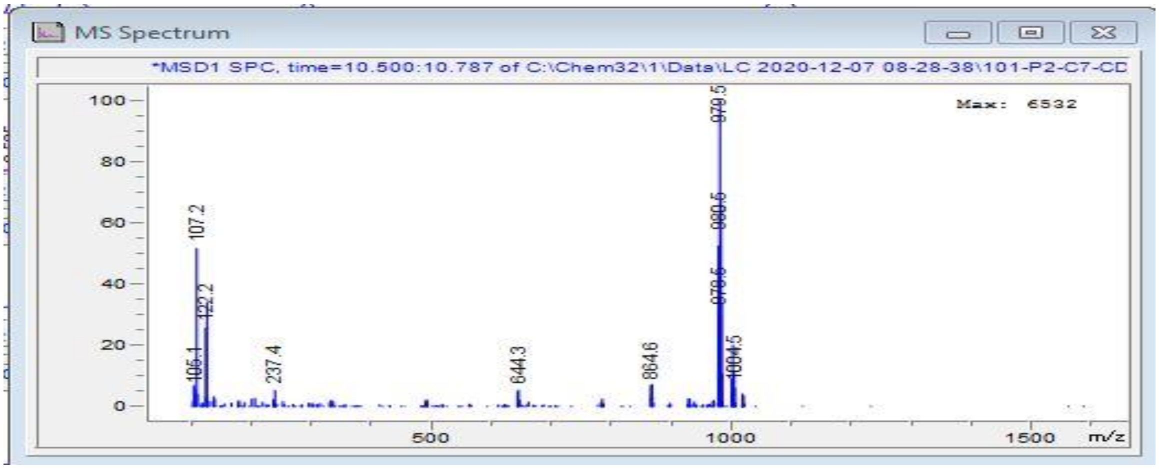
MS Spectrum
*MSD1 SPC, time=10.500:10.787 of C:\Chem32\1\Data\LC 2020-12-07 08-28-38\101-P2-C7-CD
Max: 6532
979.5
107.2
122.2
237.4
644.3
864.6
1004.5
m/z

12c

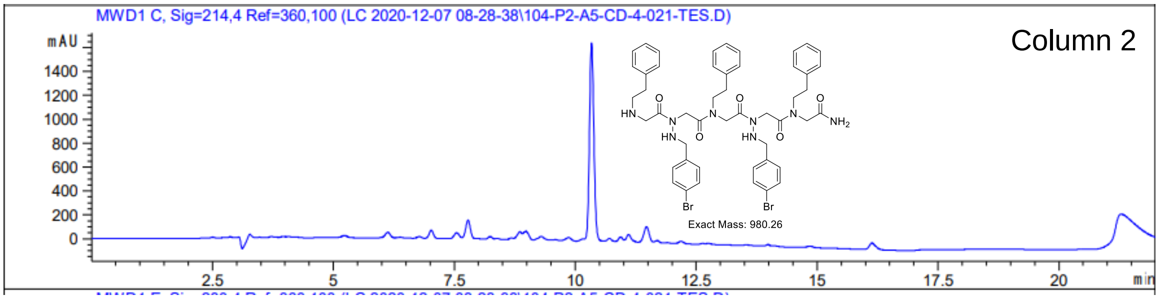
MWD1 C, Sig=214,4 Ref=360,100 (LC 2020-12-07 08-28-38\104-P2-A5-CD-4-021-TES.D)
Column 2
mAU
Exact Mass: 980.26
min

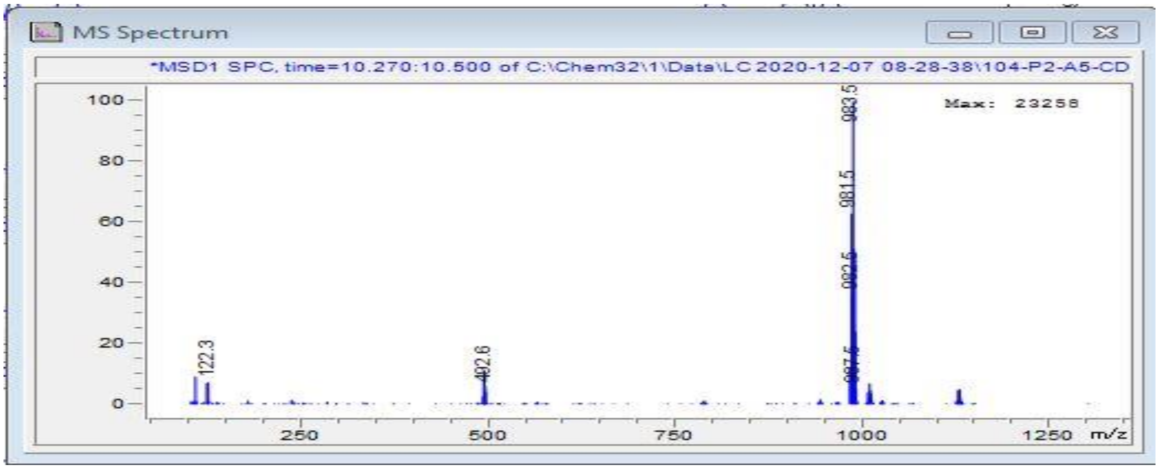
MS Spectrum
*MSD1 SPC, time=10.270:10.500 of C:\Chem32\1\Data\LC 2020-12-07 08-28-38\104-P2-A5-CD
Max: 23258
983.5
981.5
122.3
492.6
m/z

11d

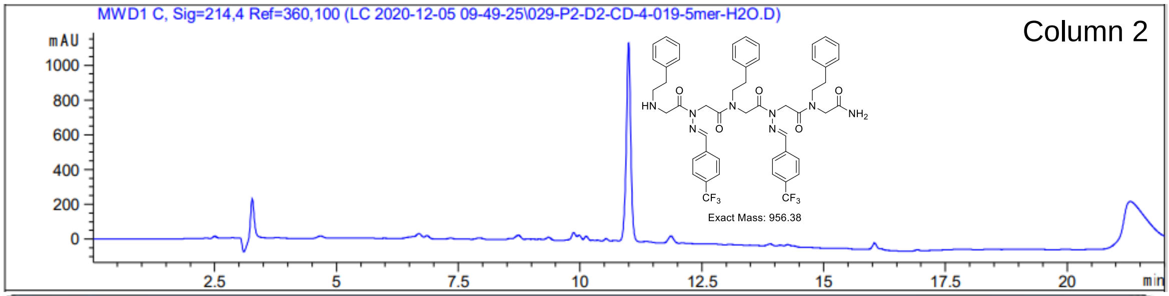

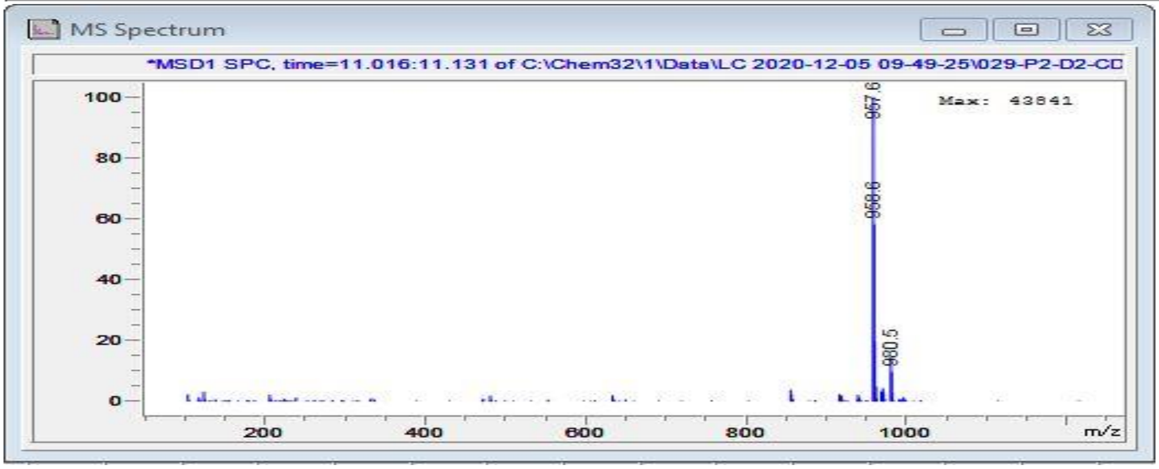

12d (Cleavage VI)

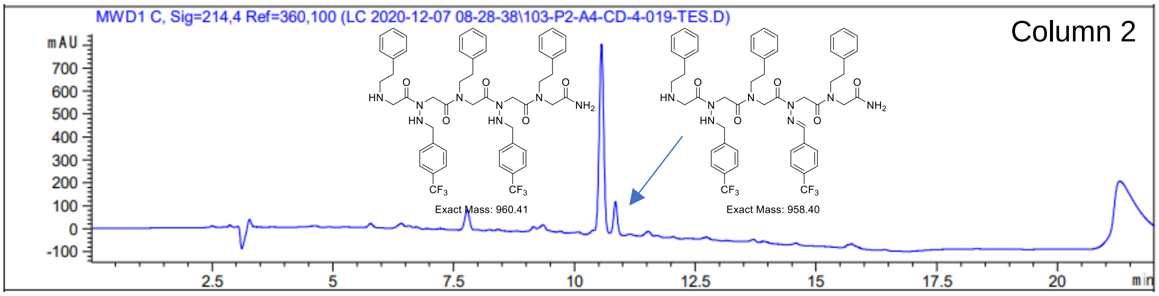

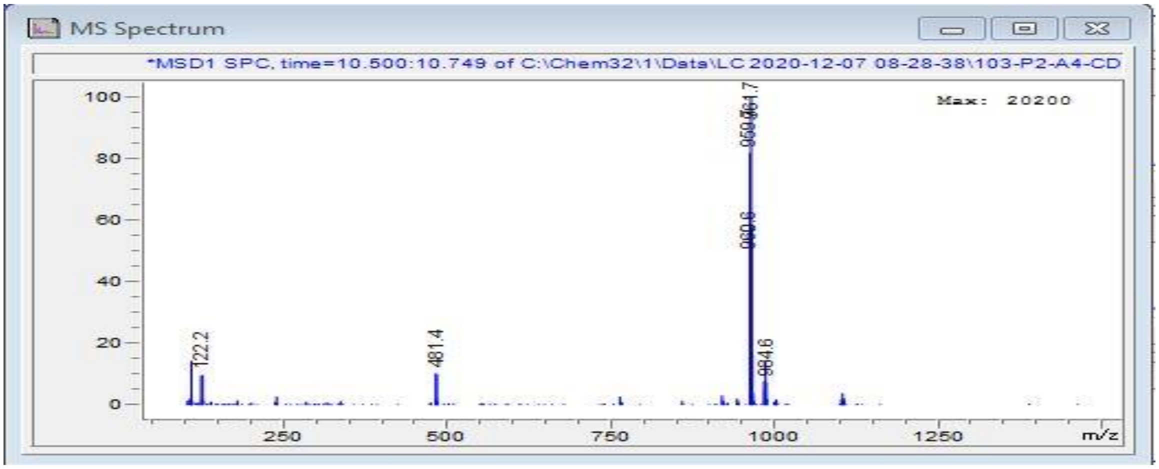

12d[a]

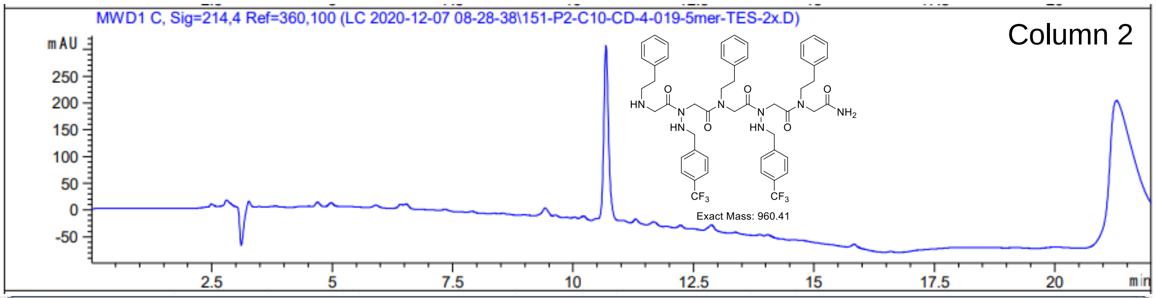

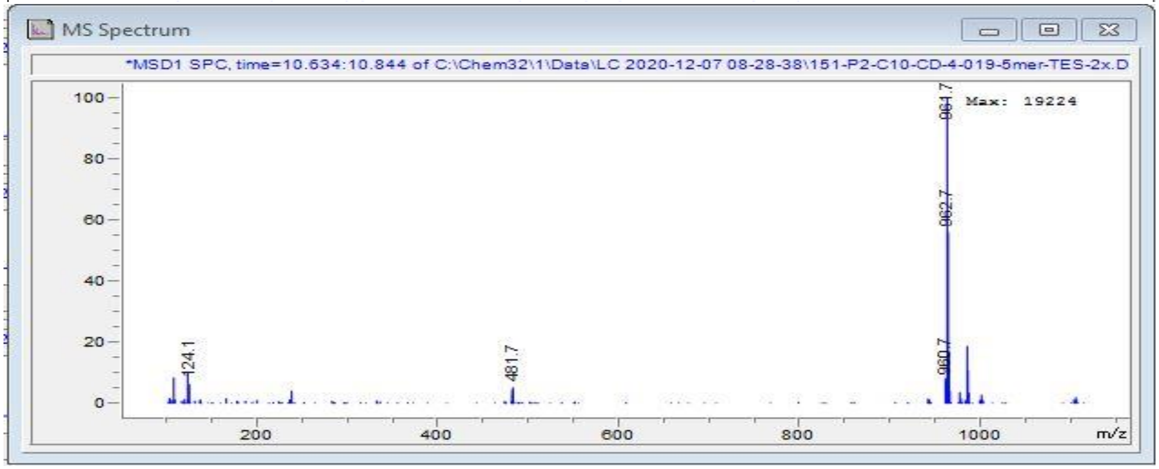

[a]Crude peptoid was treated with a fresh solution of the Cleavage VI cocktail for an additional 2 h

11e

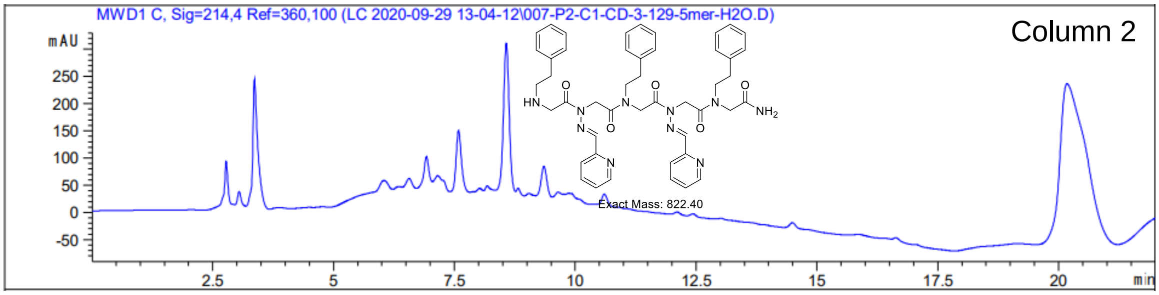

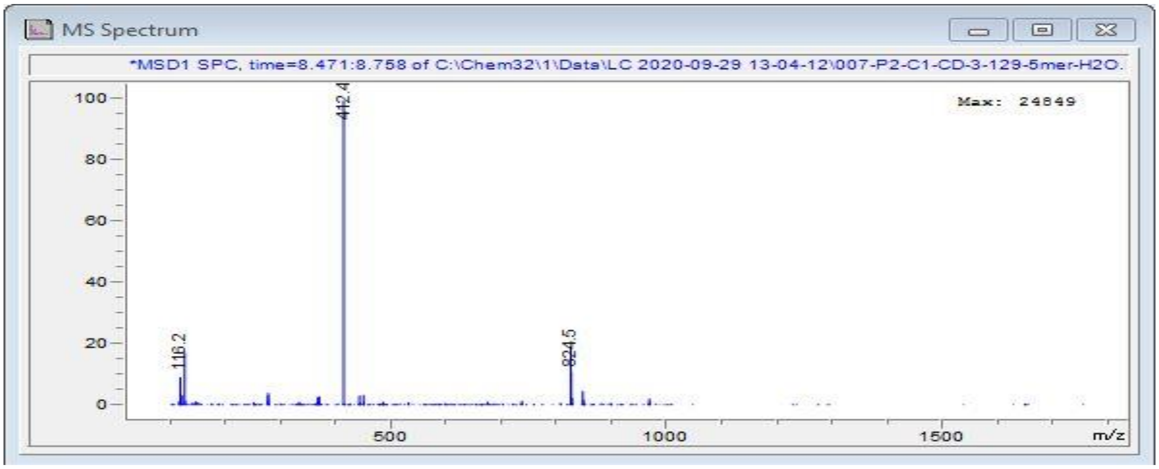

11f

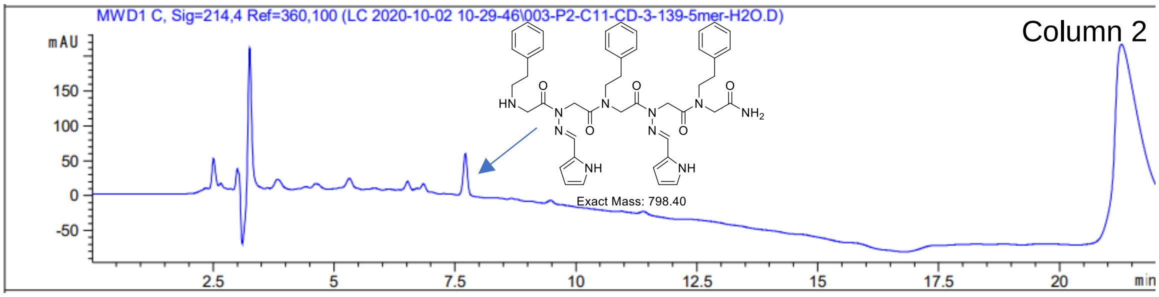
MWD1 C, Sig=214,4 Ref=360,100 (LC 2020-10-02 10-29-46\003-P2-C11-CD-3-139-5mer-H2O.D)
Column 2
Exact Mass: 798.40
mAU
min

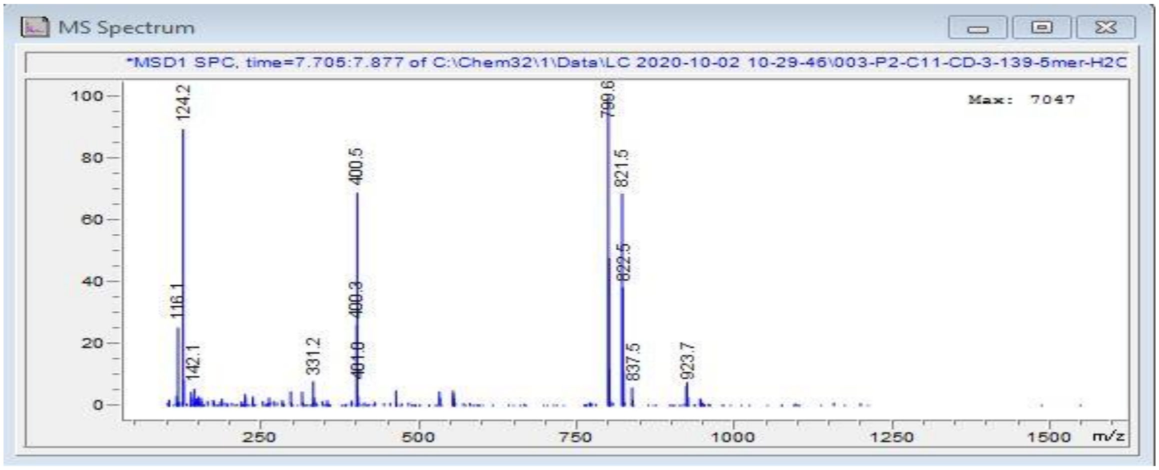
MS Spectrum
*MSD1 SPC, time=7.705:7.877 of C:\Chem32\1\Data\LC 2020-10-02 10-29-46\003-P2-C11-CD-3-139-5mer-H2C
Max: 7047
124.2
116.1
142.1
331.2
400.5
400.3
401.0
799.6
821.5
822.5
837.5
923.7
m/z

11g

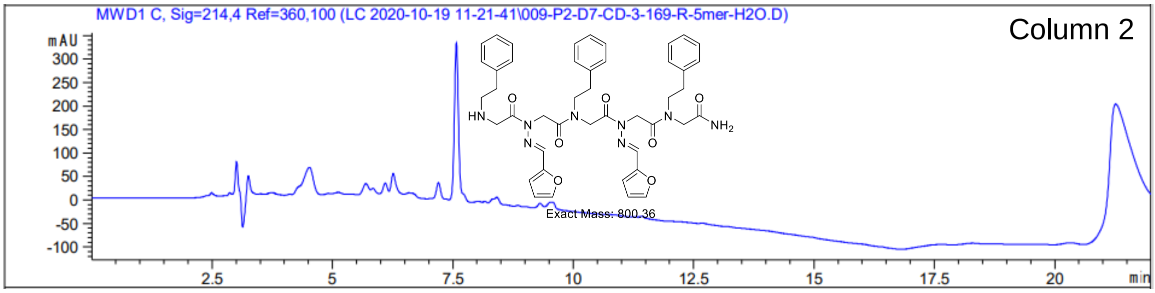
MWD1 C, Sig=214,4 Ref=360,100 (LC 2020-10-19 11-21-41\009-P2-D7-CD-3-169-R-5mer-H2O.D)
Column 2
Exact Mass: 800.36
mAU
min

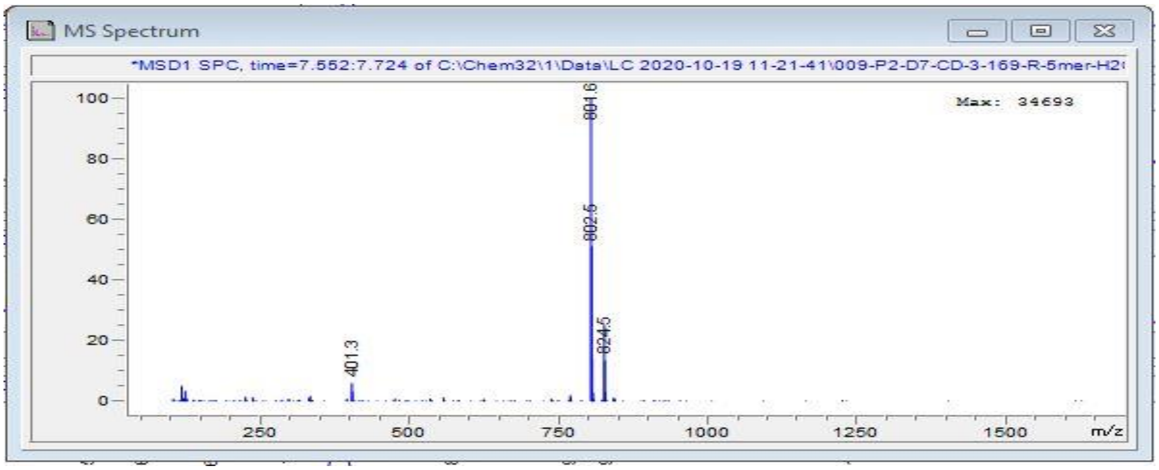
MS Spectrum
*MSD1 SPC, time=7.552:7.724 of C:\Chem32\1\Data\LC 2020-10-19 11-21-41\009-P2-D7-CD-3-169-R-5mer-H2
Max: 34693
801.6
802.5
824.5
401.3
m/z

12g

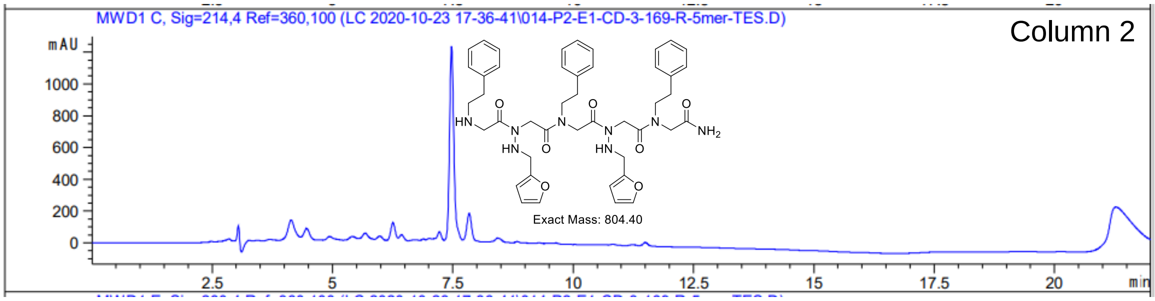
MWD1 C, Sig=214,4 Ref=360,100 (LC 2020-10-23 17-36-41\014-P2-E1-CD-3-169-R-5mer-TES.D)
Column 2
mAU
Exact Mass: 804.40
min

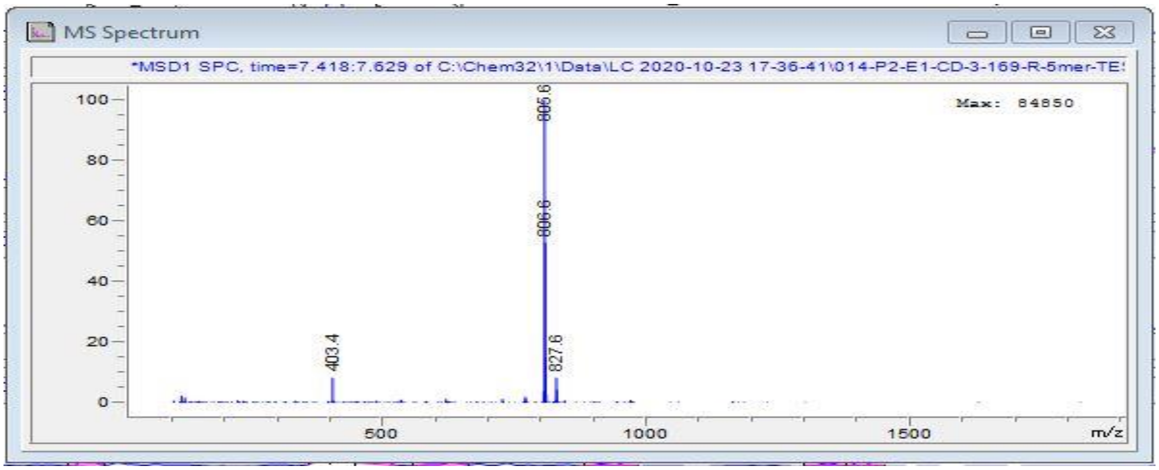
MS Spectrum
*MSD1 SPC, time=7.418:7.629 of C:\Chem32\1\Data\LC 2020-10-23 17-36-41\014-P2-E1-CD-3-169-R-5mer-TE
Max: 84850
805.6
806.6
827.6
403.4
m/z

11h

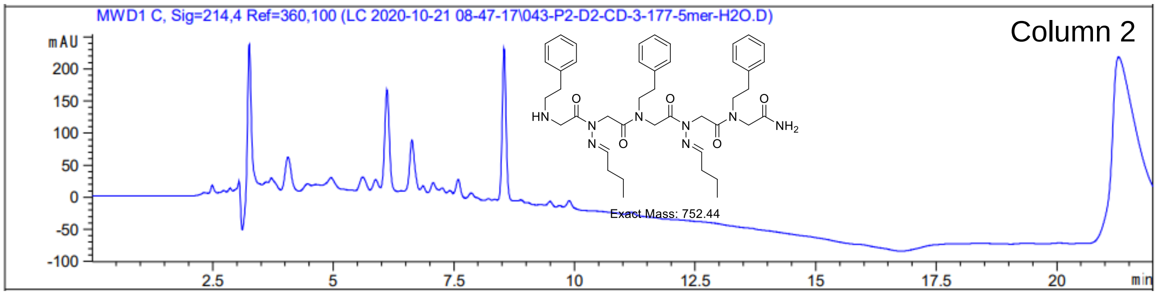
MWD1 C, Sig=214,4 Ref=360,100 (LC 2020-10-21 08-47-17\043-P2-D2-CD-3-177-5mer-H2O.D)
Column 2
mAU
Exact Mass: 752.44
min

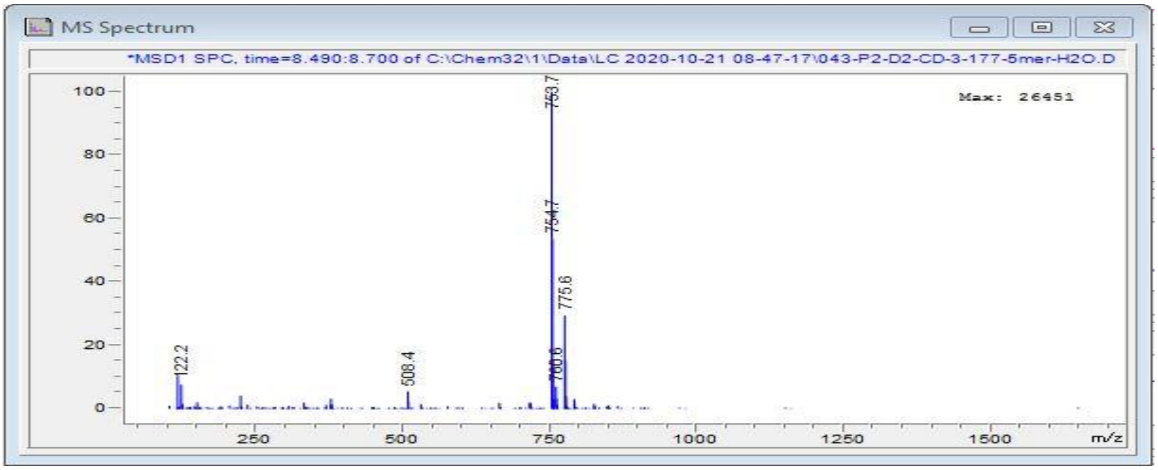
MS Spectrum
*MSD1 SPC, time=8.490:8.700 of C:\Chem32\1\Data\LC 2020-10-21 08-47-17\043-P2-D2-CD-3-177-5mer-H2O.D
Max: 26451
753.7
754.7
775.6
760.6
122.2
508.4
m/z

Davern, C.M.; Lowe, B.D.; Rosfi, A.; Ison, E.A.; Proulx, C.

12h

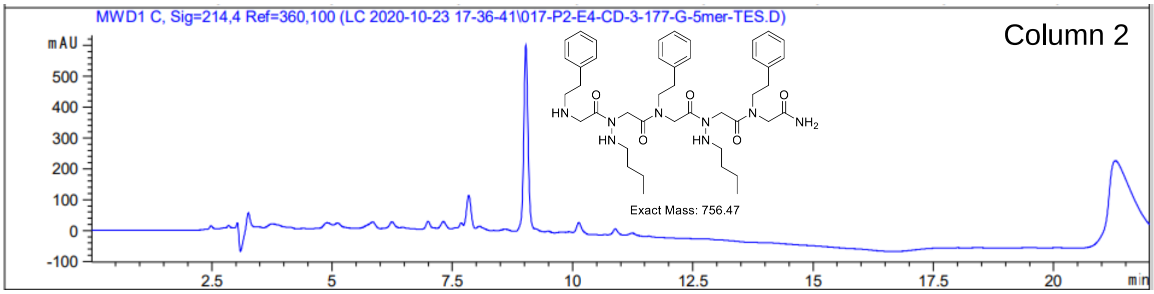

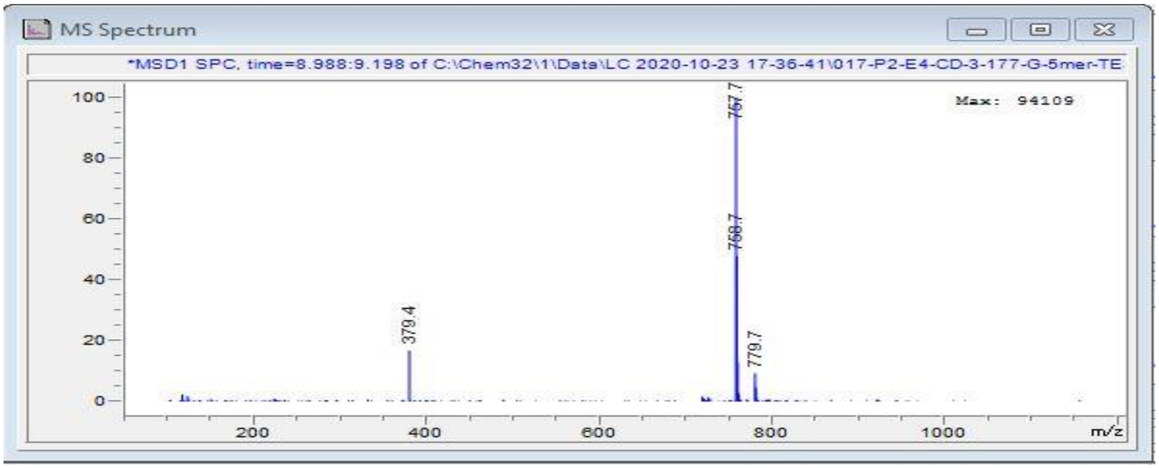

11i

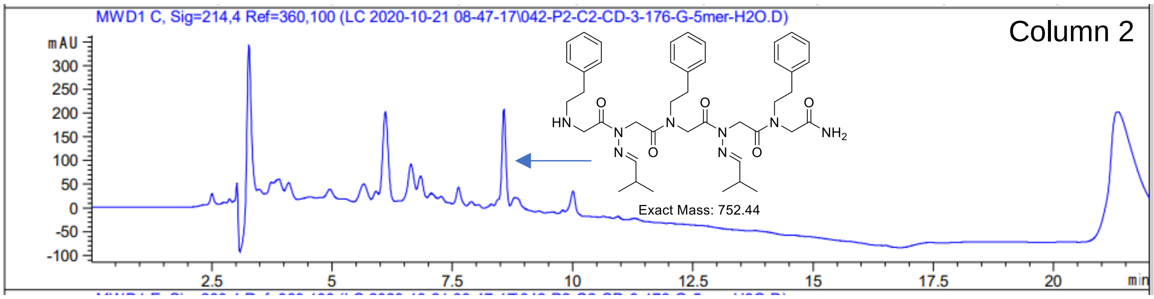

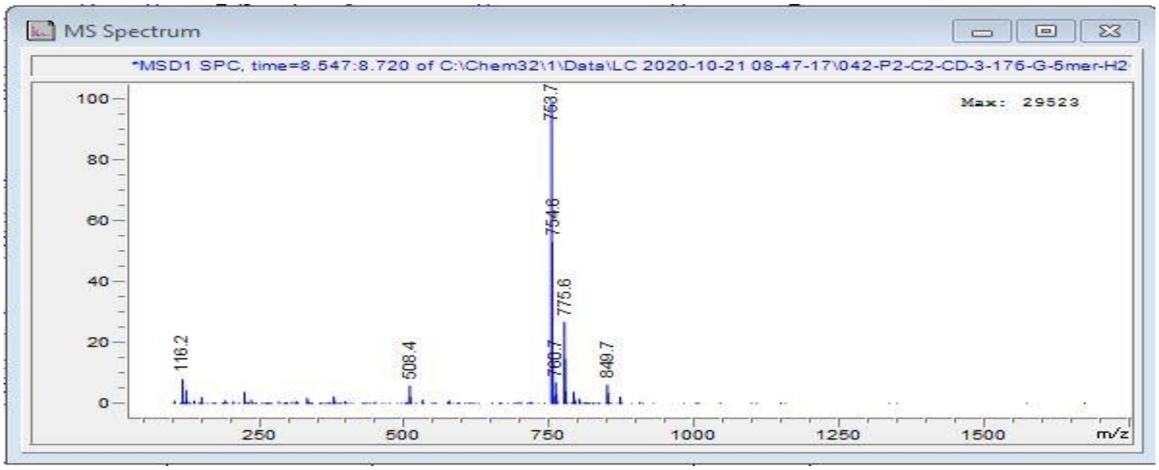

12i

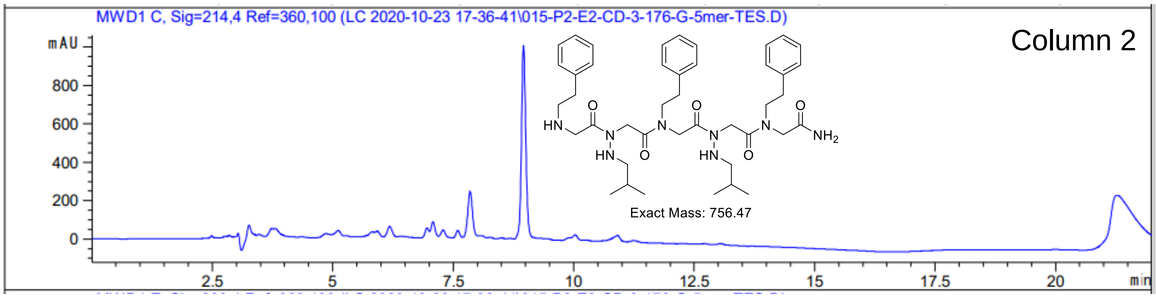
MWD1 C, Sig=214,4 Ref=360,100 (LC 2020-10-23 17-36-41\015-P2-E2-CD-3-176-G-5mer-TES.D)
Column 2
Exact Mass: 756.47
mAU
min

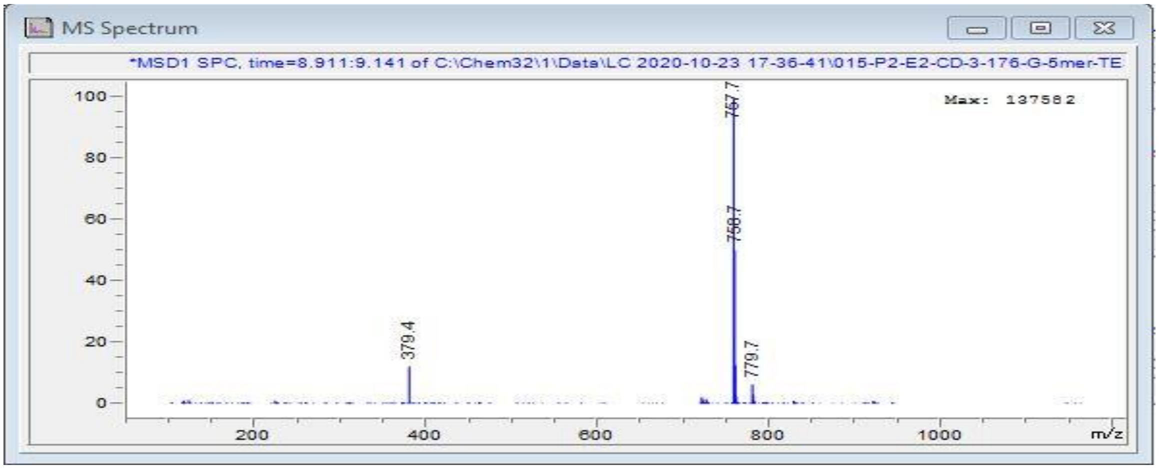
MS Spectrum
*MSD1 SPC, time=8.911:9.141 of C:\Chem32\1\Data\LC 2020-10-23 17-36-41\015-P2-E2-CD-3-176-G-5mer-TE
Max: 137582
757.7
758.7
779.7
379.4
m/z

11j

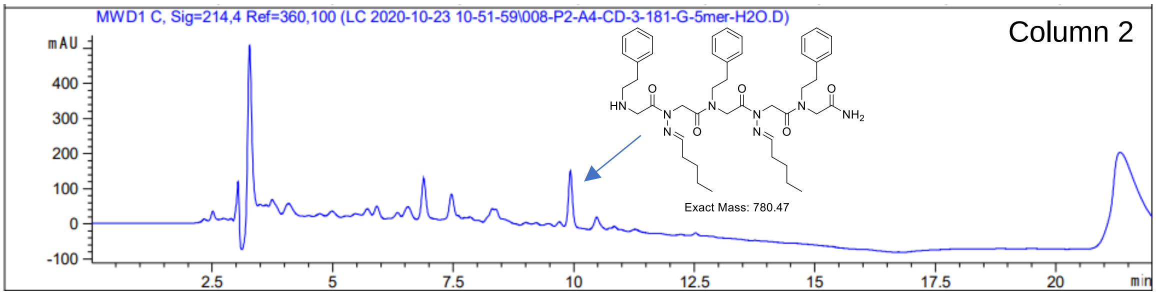
MWD1 C, Sig=214,4 Ref=360,100 (LC 2020-10-23 10-51-59\008-P2-A4-CD-3-181-G-5mer-H2O.D)
Column 2
Exact Mass: 780.47
mAU
min

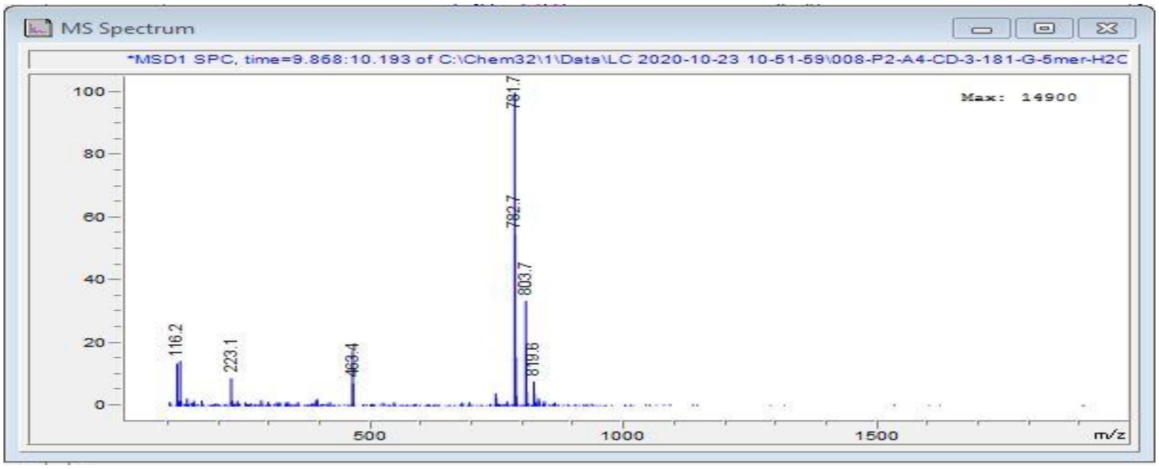
MS Spectrum
*MSD1 SPC, time=9.868:10.193 of C:\Chem32\1\Data\LC 2020-10-23 10-51-59\008-P2-A4-CD-3-181-G-5mer-H2C
Max: 14900
781.7
782.7
803.7
819.6
116.2
223.1
463.4
m/z

12j

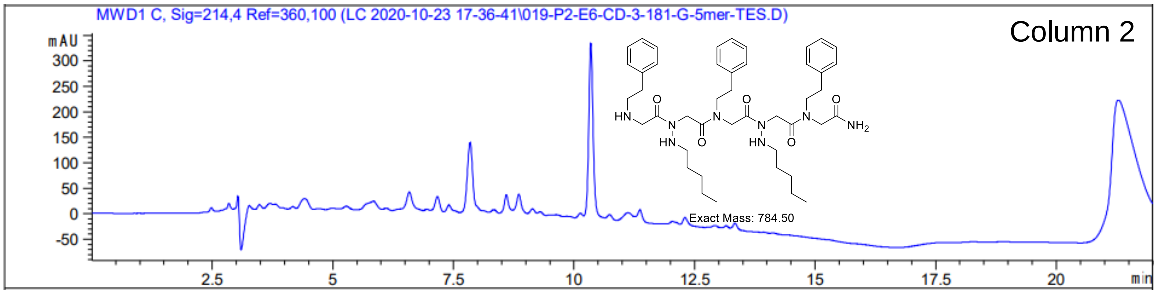
MWD1 C, Sig=214,4 Ref=360,100 (LC 2020-10-23 17-36-41\019-P2-E6-CD-3-181-G-5mer-TES.D)
Column 2
mAU
Exact Mass: 784.50
min

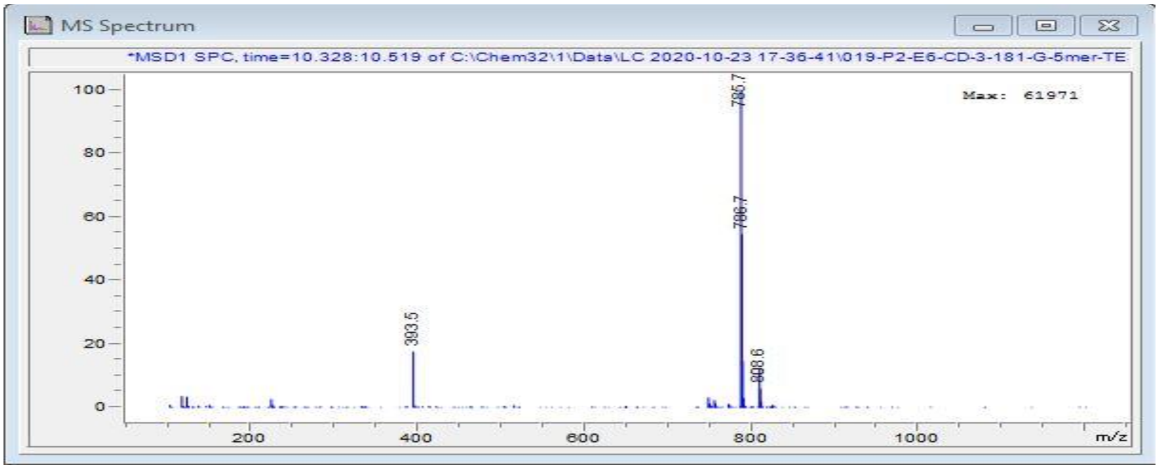
MS Spectrum
*MSD1 SPC, time=10.328:10.519 of C:\Chem32\1\Data\LC 2020-10-23 17-36-41\019-P2-E6-CD-3-181-G-5mer-TE
Max: 61971
785.7
786.7
393.5
808.6
m/z

11k

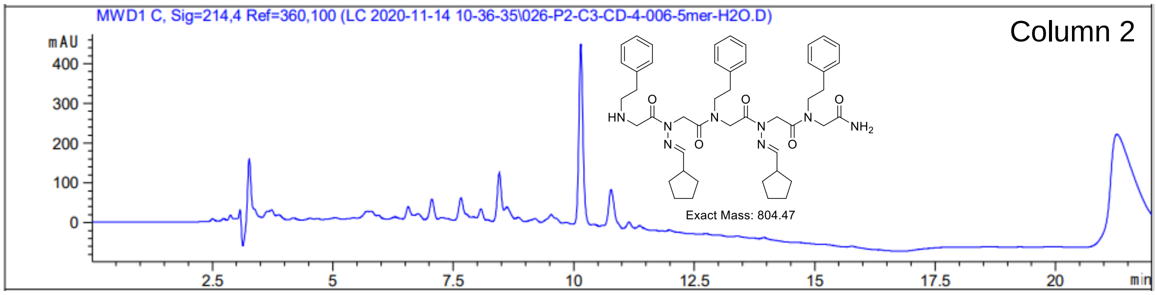
MWD1 C, Sig=214,4 Ref=360,100 (LC 2020-11-14 10-36-35\026-P2-C3-CD-4-006-5mer-H2O.D)
Column 2
mAU
Exact Mass: 804.47
min

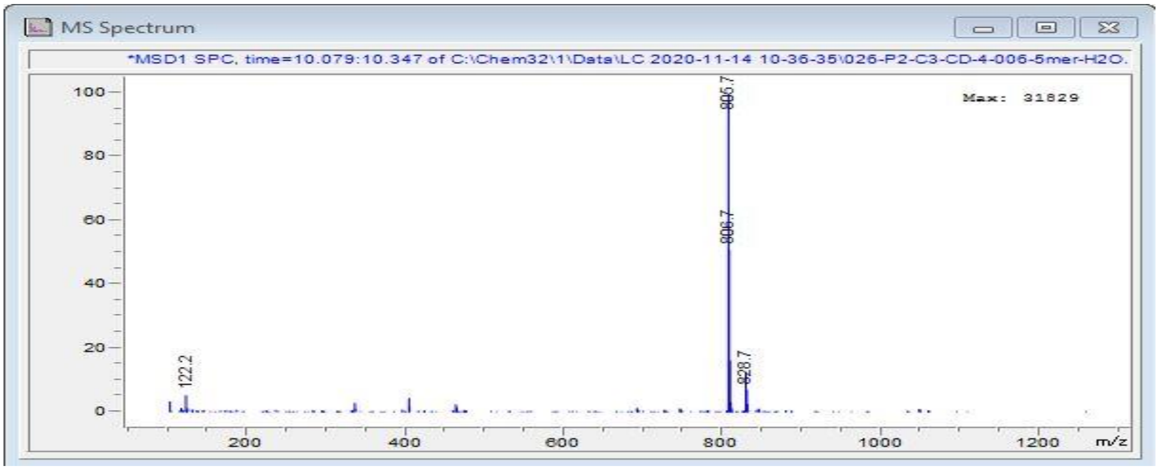
MS Spectrum
*MSD1 SPC, time=10.079:10.347 of C:\Chem32\1\Data\LC 2020-11-14 10-36-35\026-P2-C3-CD-4-006-5mer-H2O.
Max: 31829
805.7
806.7
122.2
828.7
m/z

12k

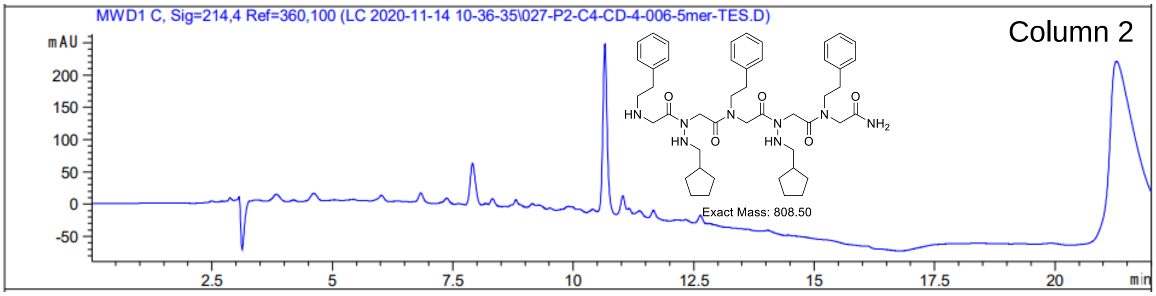
MWD1 C, Sig=214,4 Ref=360,100 (LC 2020-11-14 10-36-35\027-P2-C4-CD-4-006-5mer-TES.D)
Column 2
mAU
Exact Mass: 808.50
min

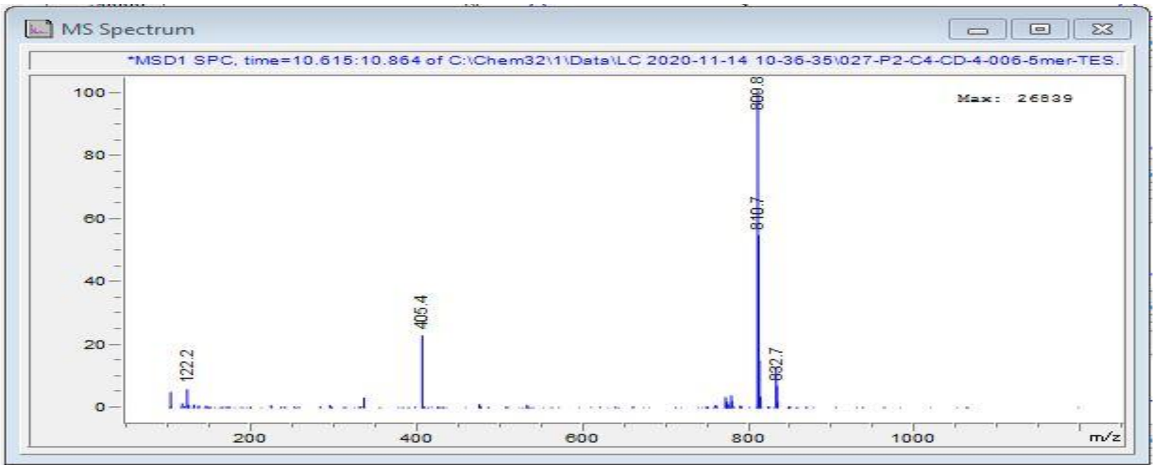
MS Spectrum
*MSD1 SPC, time=10.615:10.864 of C:\Chem32\1\Data\LC 2020-11-14 10-36-35\027-P2-C4-CD-4-006-5mer-TES.
Max: 26839
809.8
810.7
405.4
122.2
832.7
m/z

11l

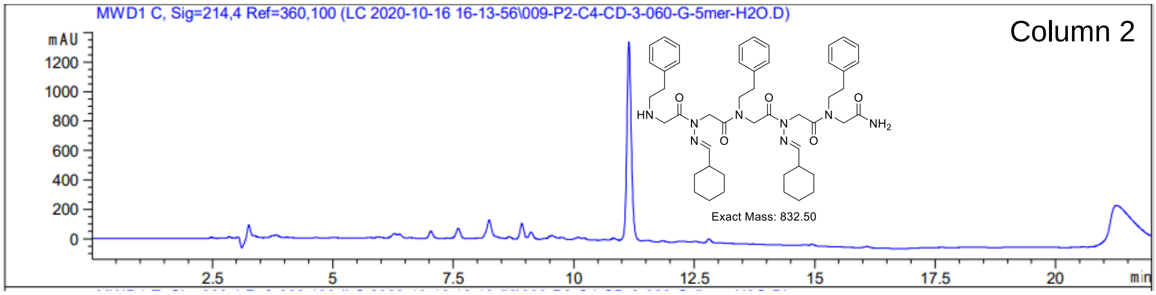
MWD1 C, Sig=214,4 Ref=360,100 (LC 2020-10-16 16-13-56\009-P2-C4-CD-3-060-G-5mer-H2O.D)
Column 2
mAU
Exact Mass: 832.50
min

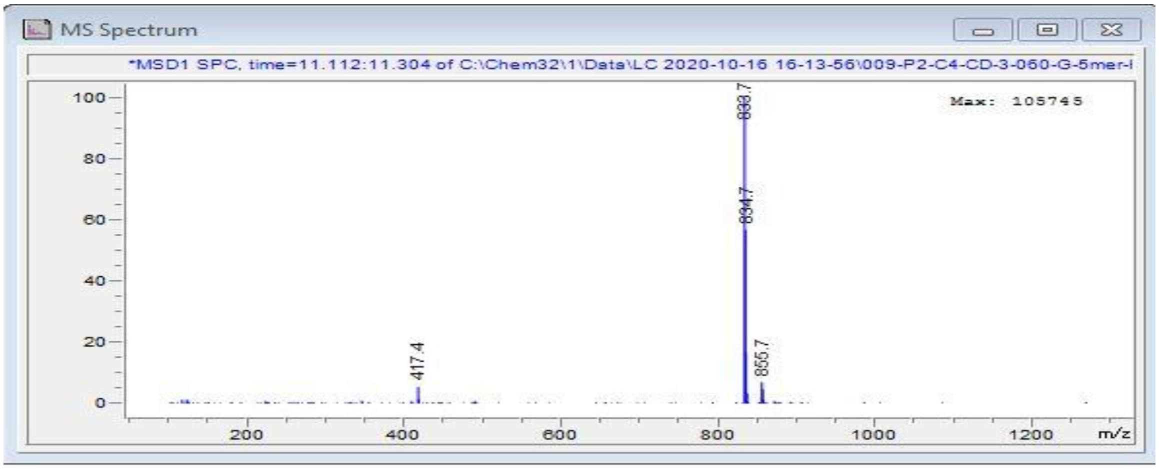
MS Spectrum
*MSD1 SPC, time=11.112:11.304 of C:\Chem32\1\Data\LC 2020-10-16 16-13-56\009-P2-C4-CD-3-060-G-5mer-I
Max: 105745
833.7
834.7
417.4
855.7
m/z

12l

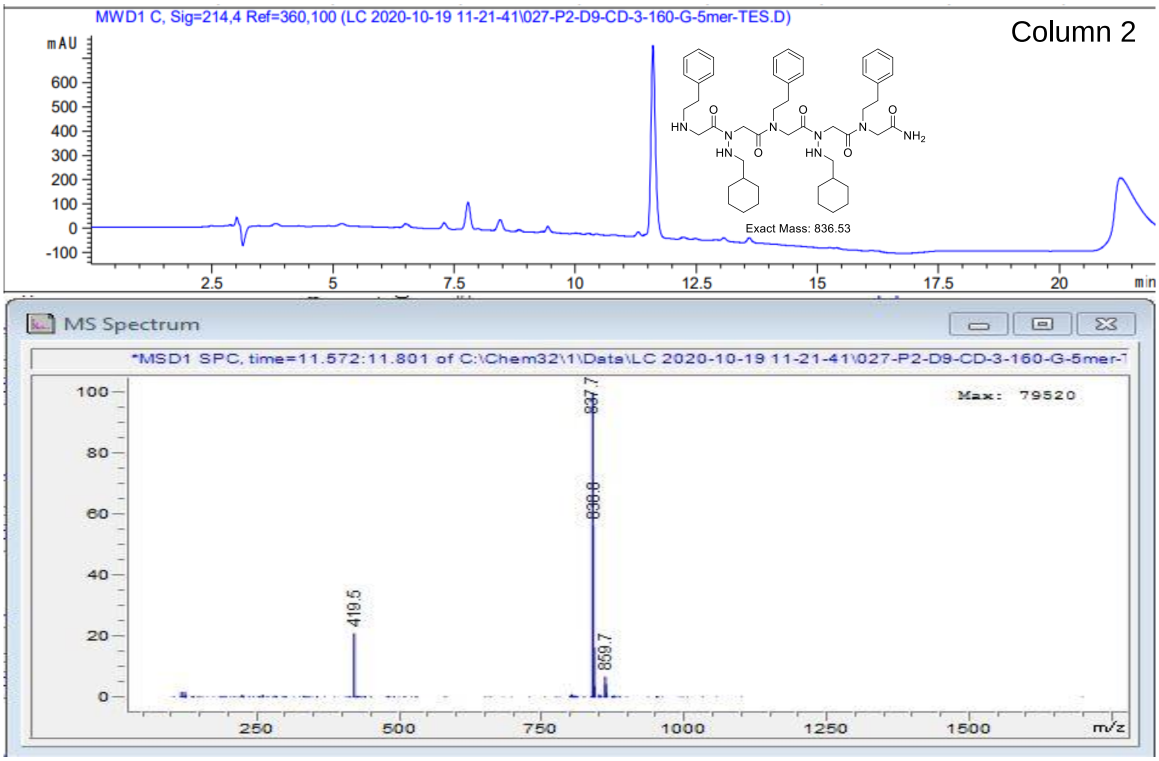
MWD1 C, Sig=214,4 Ref=360,100 (LC 2020-10-19 11-21-41\027-P2-D9-CD-3-160-G-5mer-TES.D)
Column 2
mAU
min
Exact Mass: 836.53
MS Spectrum
*MSD1 SPC, time=11.572:11.801 of C:\Chem32\1\Data\LC 2020-10-19 11-21-41\027-P2-D9-CD-3-160-G-5mer-T
Max: 79520
837.7
838.8
419.5
859.7
m/z

Characterization for stability studies performed on tripeptoids 13a-d at 1:1 0.1% FA in MeCN: 0.1% FA in H_2O

13a – 0 h

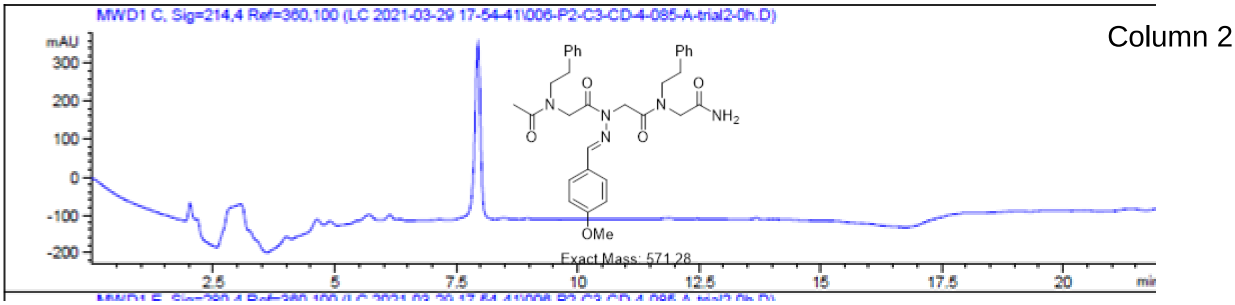

13a – 8 h

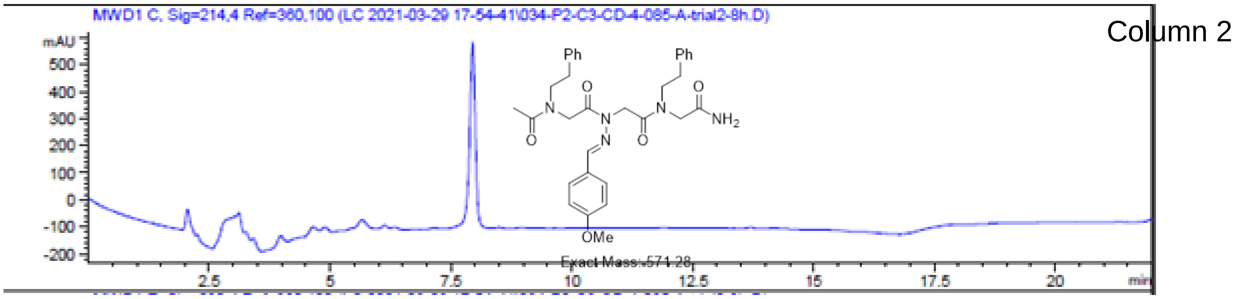

13b – 0 h

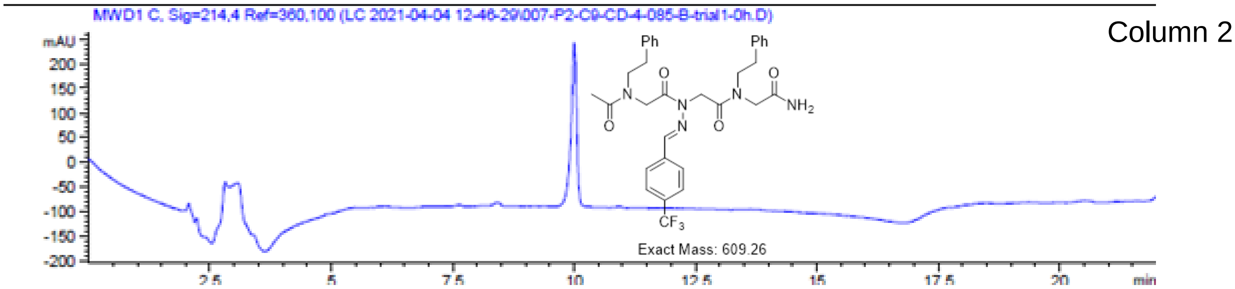

13b – 8 h

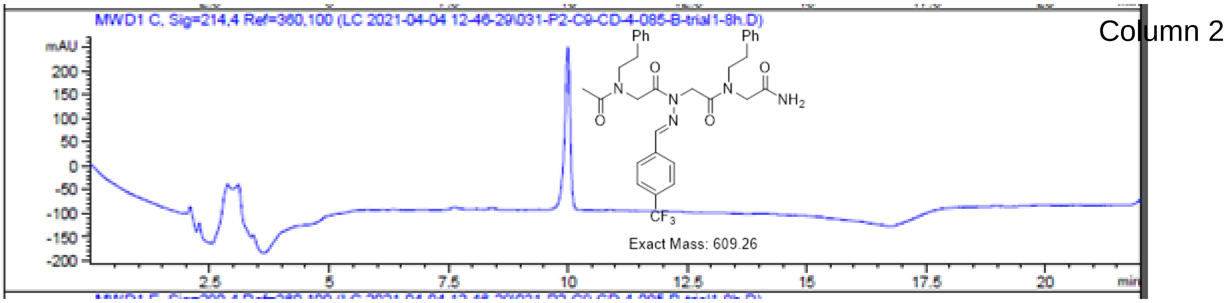

13c – 0 h

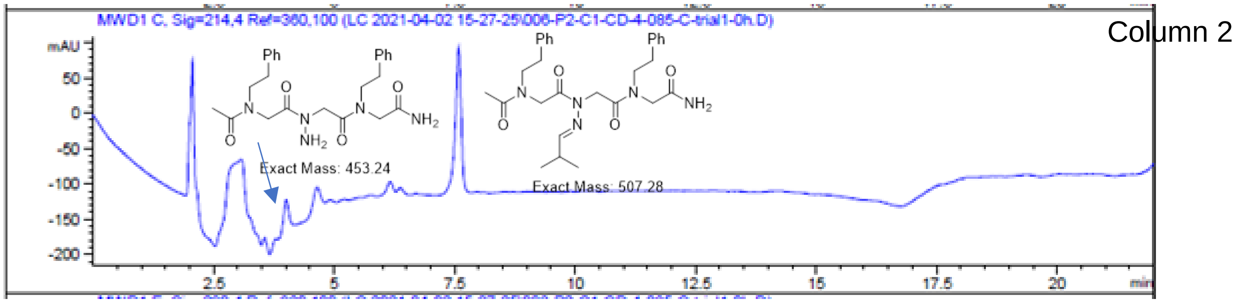

13c – 8 h

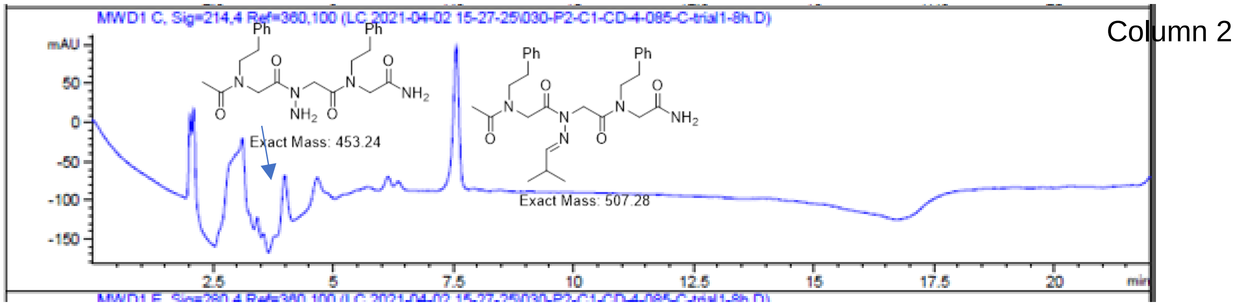

13d – 0 h

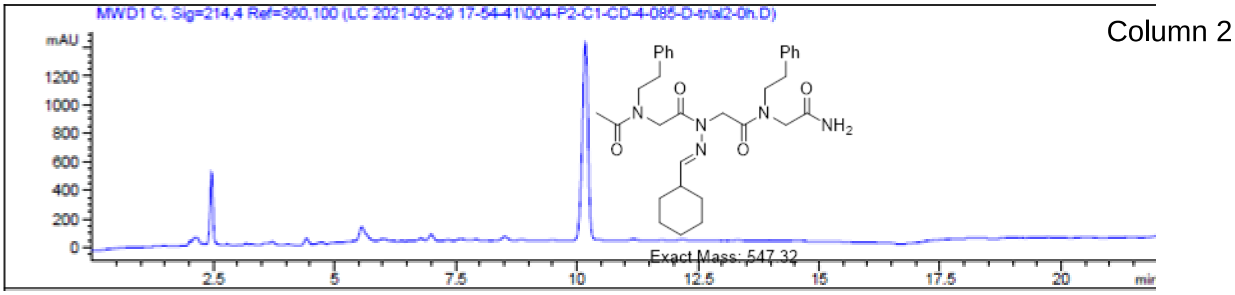

13d – 8 h

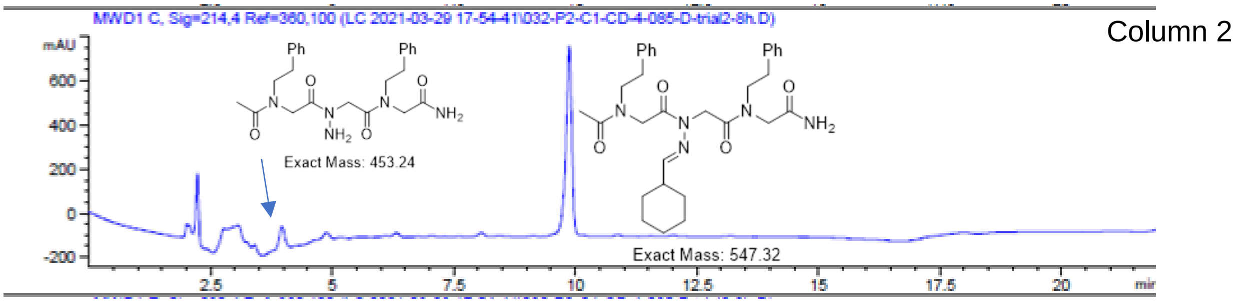

Characterization for stability studies performed on tripeptoids 13a-d at pH 7

13a – 0 h

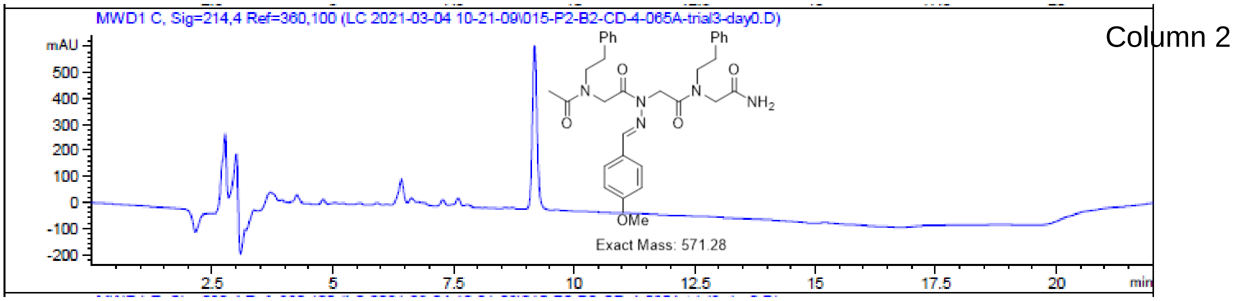

13a – 107 h

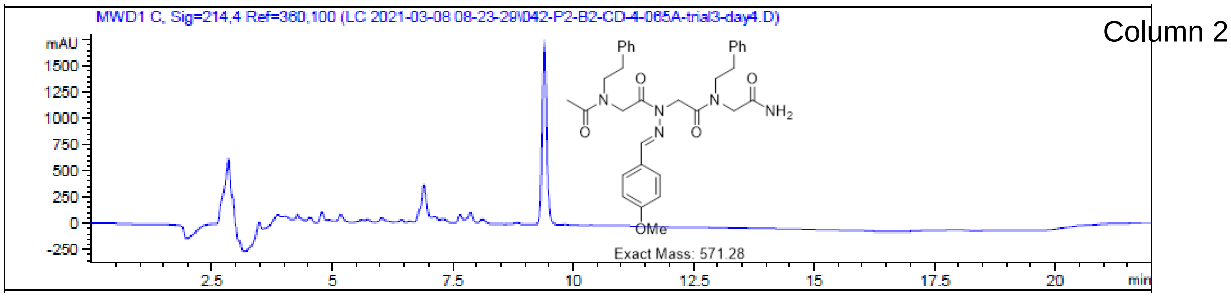

13b – 0 h

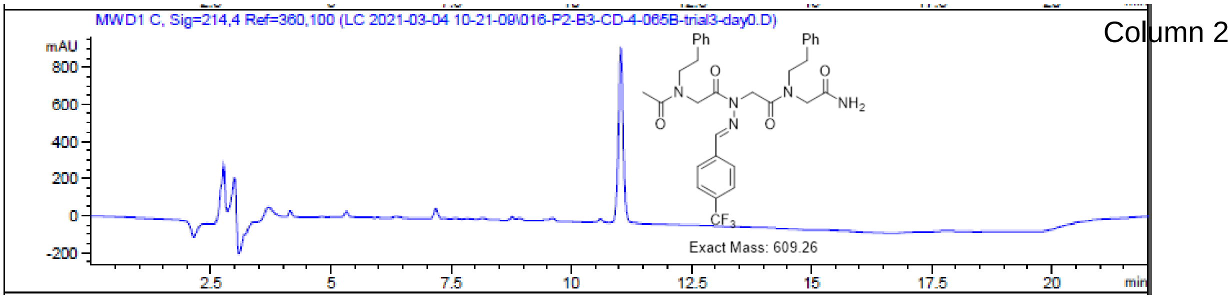

13b – 107 h

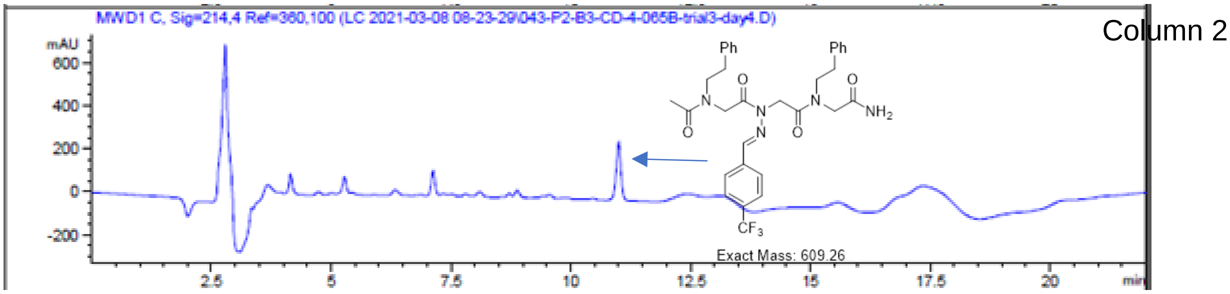

13c – 0 h

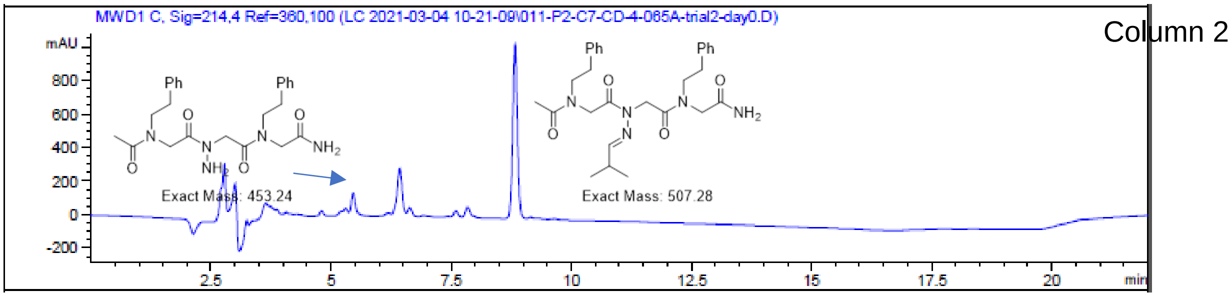

13c – 107 h

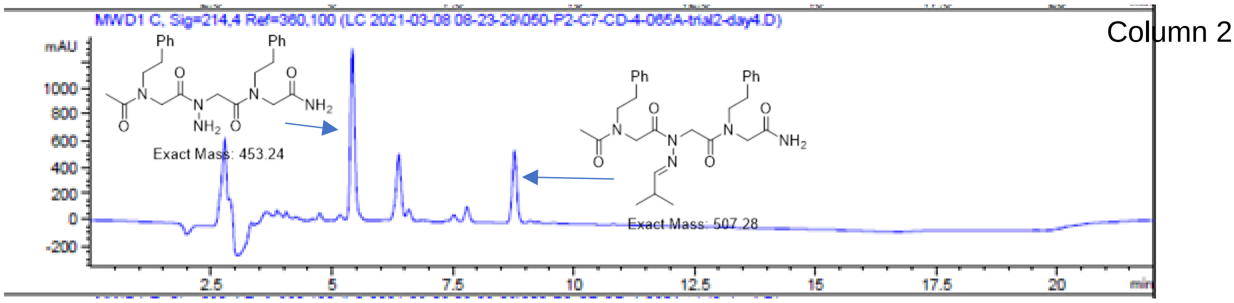

13d – 0 h

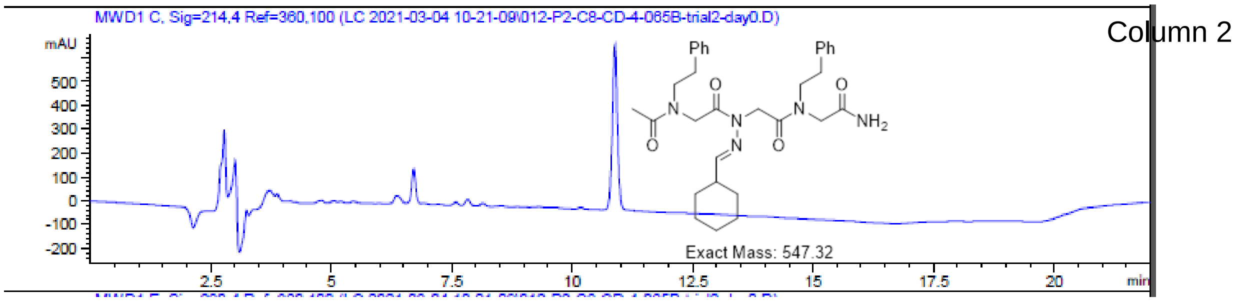

13d – 107 h

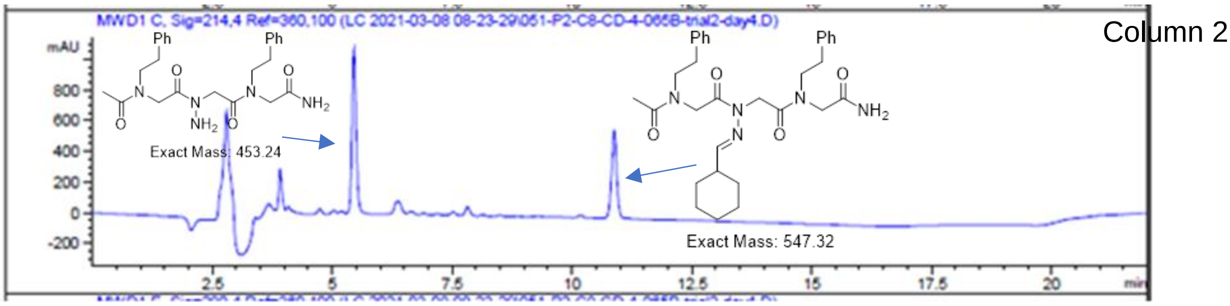

15

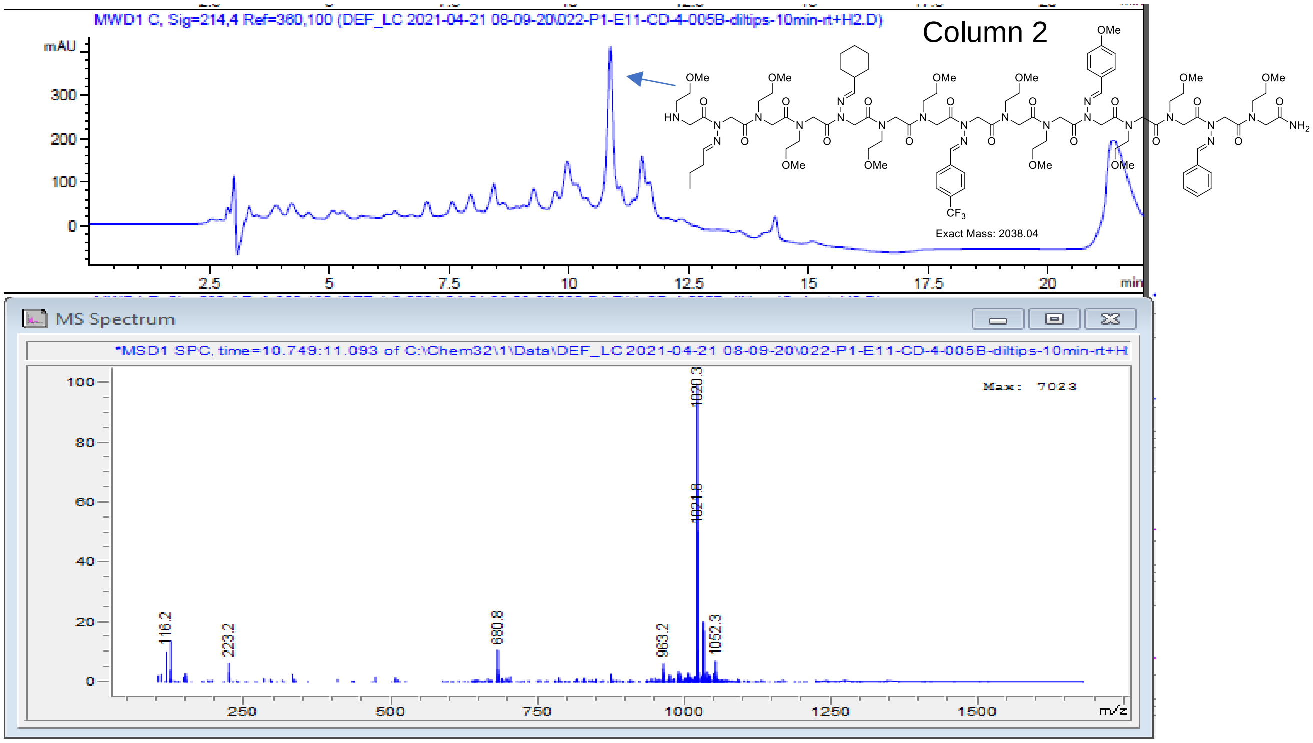
MWD1 C, Sig=214,4 Ref=360,100 (DEF_LC 2021-04-21 08-09-20\022-P1-E11-CD-4-005B-diltips-10min-rt+H2.D)
Column 2
Exact Mass: 2038.04
MS Spectrum
*MSD1 SPC, time=10.749:11.093 of C:\Chem32\1\Data\DEF_LC 2021-04-21 08-09-20\022-P1-E11-CD-4-005B-diltips-10min-rt+H
Max: 7023
1020.3
1021.8
116.2
223.2
680.8
963.2
1052.3

16

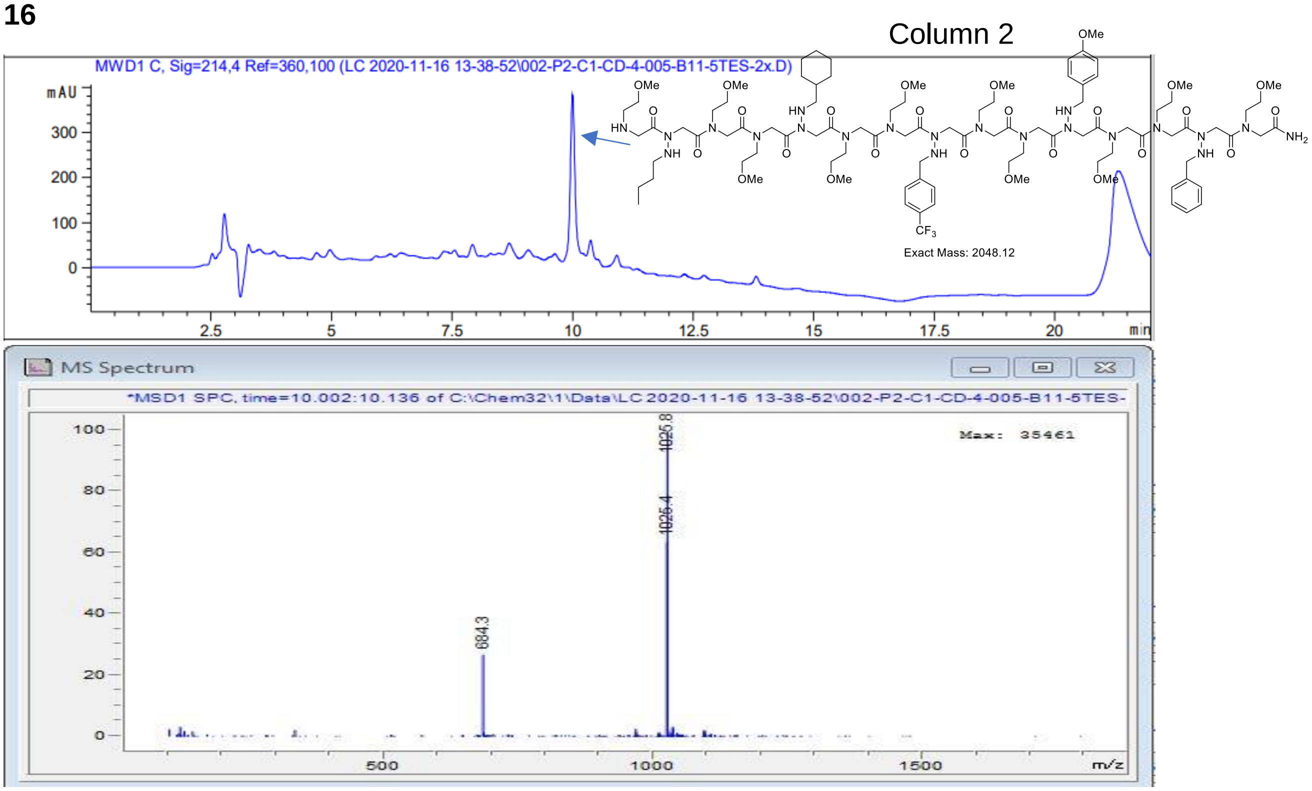
Column 2
MWD1 C, Sig=214,4 Ref=360,100 (LC 2020-11-16 13-38-52\002-P2-C1-CD-4-005-B11-5TES-2x.D)
Exact Mass: 2048.12
MS Spectrum
*MSD1 SPC, time=10.002:10.136 of C:\Chem32\1\Data\LC 2020-11-16 13-38-52\002-P2-C1-CD-4-005-B11-5TES-
Max: 35461
1025.8
1025.4
684.3

F. References

1. R. N. Zuckermann, J. M. Kerr, S. B. H. Kent and W. H. Moos, *J. Am. Chem. Soc.*, 1992, **114**, 10646–10647.
2. D. Sabatino, C. Proulx, S. Klocek, C. B. Bourguet, D. Boeglin, H. Ong and W. D. Lubell, *Org. Lett.*, 2009, **11**, 3650–3653.
3. L.-M. Zhao, H.-S. Jin, J. Liu, T. C. Skaar, J. Ipe, W. Lv, D. A. Flockhart and M. Cushman, *Bioorg. Med. Chem.*, 2016, **24**, 5400–5409.
4. N. Chen, X.-J. Dai, H. Wang and C.-J. Li, *Angew. Chem. Int. Ed.*, 2017, **56**, 6260–6263.
5. T. S. Burkoth, A. T. Fafarman, D. H. Charych, M. D. Connolly and R. N. Zuckermann, *J. Am. Chem. Soc.*, 2003, **125**, 8841–8845.
6. J. A. Crapster, J. R. Stringer, I. A. Guzei and H. E. Blackwell, *Pept. Sci.*, 2011, **96**, 604–616.
7. B. C. Gorske, B. L. Bastian, G. D. Geske and H. E. Blackwell, *J. Am. Chem. Soc.*, 2007, **129**, 8928–8929.
8. M. J. Frisch, G. W. Trucks, H. B. Schlegel, G. E. Scuseria, M. A. Robb, J. R. Cheeseman, G. Scalmani, V. Barone, G. A. Petersson, H. Nakatsuji, X. Li, M. Caricato, A. V. Marenich, J. Bloino, B. G. Janesko, R. Gomperts, B. Mennucci, H. P. Hratchian, J. V. Ortiz, A. F. Izmaylov, J. L. Sonnenberg, D. Williams-Young, F. Ding, F. Lipparini, F. Egidi, J. Goings, B. Peng, A. Petrone, T. Henderson, D. Ranasinghe, V. G. Zakrzewski, J. Gao, N. Rega, G. Zheng, W. Liang, M. Hada, M. Ehara, K. Toyota, R. Fukuda, J. Hasegawa, M. Ishida, T. Nakajima, Y. Honda, O. Kitao, H. Nakai, T. Vreven, K. Throssell, J. A. Montgomery Jr., J. E. Peralta, F. Ogliaro, M. J. Bearpark, J. J. Heyd, E. N. Brothers, K. N. Kudin, V. N. Staroverov, T. A. Keith, R. Kobayashi, J. Normand, K. Raghavachari, A. P. Rendell, J. C. Burant, S. S. Iyengar, J. Tomasi, M. Cossi, J. M. Millam, M. Klene, C. Adamo, R. Cammi, J. W. Ochterski, R. L. Martin, K. Morokuma, O. Farkas, J. B. Foresman and D. J. Fox, *Gaussian 16, Revision C.01*, Gaussian, Inc., Wallingford, CT, 2016.
9. A. D. Becke, *J. Chem. Phys.*, 1993, **98**, 5648–5652.
10. C. Lee, W. Yang and R. G. Parr, *Phys. Rev. B*, 1988, **37**, 785–789.
11. S. H. Vosko, L. Wilk and M. Nusair, *Can. J. Phys.*, 1980, **58**, 1200–1211.
12. P. J. Stephens, F. J. Devlin, C. F. Chabalowski and M. J. Frisch, *J. Phys. Chem.*, 1994, **98**, 11623–11627.
13. W. J. Hehre, R. Ditchfield and J. A. Pople, *J. Chem. Phys.*, 1972, **56**, 2257–2261.
14. R. Krishnan, J. S. Binkley, R. Seeger and J. A. Pople, *J. Chem. Phys.*, 1980, **72**, 650–654.
15. S. Grimme, J. Antony, S. Ehrlich and H. Krieg, *J. Chem. Phys.*, 2010, **132**, 154104.
16. M. Cossi, V. Barone, R. Cammi and J. Tomasi, *Chem. Phys. Lett.*, 1996, **255**, 327–335.
17. J. P. Foster and F. Weinhold, *J. Am. Chem. Soc.*, 1980, **102**, 7211–7218.
18. E. D. Glendening, C. R. Landis and F. Weinhold, *J. Comput. Chem.*, 2013, **34**, 1429–1437.
